# Transcriptome Analysis of Platelet-Rich Plasma–Treated Osteoarthritic Chondrocyte

**DOI:** 10.1155/2024/7680736

**Published:** 2024-11-21

**Authors:** Dae Keun Suh, Woo Jin Yeo, Kuhoang Cheong, Jae-Won Heo, Dong Hyeon Kim, Soo Mi Lee, Yong-Soo Lee, Dong Won Suh

**Affiliations:** ^1^Department of Orthopaedic Surgery, Kyung Hee University Hospital, Seoul, Republic of Korea; ^2^Joint Center, Barunsesang Hospital, Yatap-ro 75-5, Seongnam-si, Gyeonggi-do, Republic of Korea; ^3^Research Center for Cartilage Regeneration, Barunsesang Hospital, Yatap-ro 75-5, Seongnam-si, Gyeonggi-do, Republic of Korea

## Abstract

As a blood-derived biomaterial, platelet-rich plasma (PRP) has been considered a potential therapy and tried in knee and hip osteoarthritis with beneficial effects as an anti-inflammatory and potent regenerative agent. To better understand the substantial effect of PRP on chondrocytes in an inflammatory environment, we analyzed the transcriptome profile by RNA sequencing (RNA-seq) after PRP administration in IL-1*β*-treated osteoarthritic chondrocytes which were isolated from human knee articular cartilage tissue. A total of 24,424 genes were analyzed, and significant changes in expression were observed for 226 genes in the control (CTL) versus IL-1*β* group and 300 genes in the IL-1*β* versus IL-1*β*+PRP group. The Top 20 significantly upregulated and downregulated genes and the major altered genes in nine categories that are closely related to chondrocyte physiology were analyzed, and the expression of several important genes in each category was evaluated by qRT-PCR and western blot analysis. Our study revealed that the PRP, at the gene expression level, has apparent anti-inflammatory, cell proliferative, and regenerative activities in chondrocytes in the presence of IL-1*β*, which mimic an osteoarthritic environment. Identifying potent molecules that regulate cartilage physiology represents a promising therapeutic approach for suppressing cartilage degeneration, especially that caused by inflammation-induced osteoarthritis.

## 1. Introduction

Osteoarthritis (OA) is a degenerative disease that results in the irreversible, progressive destruction of the articular cartilage, causing chronic pain, movement restriction, and eventually reduced quality of life. Recently, the prevalence of OA has steadily increased due to aging, obesity, and sports injuries, placing considerable social burden on public health [1, 2]. Despite its high prevalence, symptomatic relief is attained with painkillers for patients with relatively mild symptoms, while surgery is inevitable for patients with more severe symptoms [3]. Therefore, developing drugs that can effectively and fundamentally treat OA is urgently needed. Drug development or regenerative approaches are currently being attempted in various fields. However, promising pharmacological therapy that exhibits convincing disease-modifying efficacy is not discovered yet [4, 5].

Articular cartilage primarily consists of a dense extracellular matrix (ECM) with a sparse population of highly specialized chondrocytes. As a unique cell type in articular cartilage, chondrocytes are essential for ECM formation and the maintenance of cartilage homeostasis [6]. Chondrocytes synthesize various anabolic and catabolic factors to maintain cartilage homeostasis, including growth factors, enzymes, and cell signaling mediators [7]. Since articular cartilage lacks blood vessels, lymphatics, and nerves, it has a limited capacity for intrinsic healing and repair [8]. Therefore, if a potent bioactive substance capable of maintaining the normal function of chondrocytes can be directly delivered to the cartilage, it will be helpful for the recovery or regeneration of damaged cartilage and maintenance of ECM homeostasis.

Because the molecular mechanisms of OA are complex and involve various factors, including genetic predisposition, acute injury, and chronic inflammation, it is challenging to control OA by regulating a single factor only [9, 10]. However, as the term “arthritis” implies, inflammatory signaling is well known to contribute the most to cartilage degradation in OA. Since mechanical stresses or various degenerative environments eventually result in the inflammation of chondrocytes, degeneration of the articular cartilage occurs, ultimately resulting in OA [11, 12]. Therefore, identifying bioactive substances that can control chondrocyte inflammation triggered by a range of factors may be essential for treating OA.

Platelet-rich plasma (PRP) is a blood product consisting of a high concentration of platelets in a small volume of plasma with a variety of growth factors, cytokines, and mediators in their alpha granules [13]. PRP has been attracting attention and has been applied for cartilage regeneration owing to its regenerative properties. However, there are some controversial opinions with respect to its efficacy due to the lack of standardization of the PRP preparation method and the number, timing, and volume of applications [14–18]. PRP exhibits anti-inflammatory properties in various cell types, including synoviocytes, macrophages, and chondrocytes [19]. More noteworthy is the fact that effective and beneficial results have recently been verified in numerous clinical trials, and more standardized PRP preparation methods are being developed [20]. To better understand the substantial effect of PRP on chondrocytes in an inflammatory environment, we analyzed the transcriptome profile after PRP administration in IL-1 *β*-treated osteoarthritic human chondrocytes by RNA sequencing (RNA-seq).

## 2. Materials and Methods

This study was approved by the Public Institutional Review Board of the Ministry of Health and Welfare (IRB no. P01-202012-31-002). Written informed consent was obtained from all patients who agreed to participate in this study. We complied with all the relevant ethical regulations for working with human participants in accordance with the National Institutes of Health Guidelines.

### 2.1. Subjects and Chondrocyte Isolation

Among the patients in our institution who were surgically treated for OA of the knee by total knee replacement (TKR) surgery, we selected 37 patients (mean age, 70.3 ± 6.52 years; 9 males and 28 females) who had no steroid injection within 3 months before the surgery and had no previous history of surgery on the knees. Relatively healthy cartilage tissues were obtained by direct dissection of the cartilage tissues using a surgical blade following TKR under sterile conditions. The cartilage tissue was minced into 1–2-mm^3^ pieces, washed with PBS 3–4 times, and sequentially digested with 0.2% type II collagenase for 4 h in an incubator (37°C, 5% CO_2_). After filtration with a 70 *μ*m syringe filter, the filtrated cells were centrifuged at 300 g for 10 min and washed twice with Dulbecco's modified Eagle's medium (DMEM, Corning, Corning, NY, USA). The chondrocytes were resuspended in DMEM supplemented with 10% fetal bovine serum (FBS; Gibco, Thermo Fisher Scientific, USA) containing antibiotics and incubated at 37°C in a humidified atmosphere containing 5% CO_2_.

### 2.2. Preparation of PRP

Whole blood (~40 mL) was collected from the median cubital vein by venipuncture into a sterile 50-mL tube containing 6 mL anticoagulant citrate dextrose solution, Solution A (ACD-A, Nothrom, Aju Pharm, Republic of Korea). The first centrifugation step was performed at 2000 rpm for 10 min at room temperature. The blood sample was divided into three layers: plasma in the first layer, a buffy coat in the middle layer, and red blood cells in the third layer. The plasma and buffy layers were collected for a second centrifugation at 4000 rpm for 10 min at room temperature. A third of the supernatant was removed, and the remnant was resuspended using a syringe with a 25-G needle and then placed at −80°C in a deep freezer for 10 min. After thawing, the PRP was homogenized using a homogenizer (T10 Basic, IKA, Germany) for 10 s and centrifuged at 13,000 rpm for 20 min at room temperature. The supernatant was aliquoted into a 1.5 mL sterile tube and stored in a deep freezer for further use.

### 2.3. Primary Chondrocyte Culture, IL-1*β* Treatment, and PRP Administration

The isolated primary chondrocytes were maintained in DMEM containing 10% FBS and antibiotics. These were used for experiments at passages 3–4. The cells were plated at a density of 0.8 × 10^6^ cells in a 60 mm culture plate and incubated for 48 h to reach 80%–90% confluency. To induce osteoarthritic conditions, the cells were treated with 1 ng/ml of IL-1*β* (Cell Signaling Technology, Danvers, MA, USA). PRP (50 *μ*L/mL media) administration was performed 4 h after IL-1*β* challenge for 48 h. The optimal concentrations of IL-1*β* and PRP were selected from preliminary experiments using qRT-PCR against matrix metallopeptidase 3 (MMP3) and MMP13, the representative catabolic factors for chondrocytes, using a broad concentration range for IL-1*β* and PRP.

### 2.4. RNA Isolation, Library Preparation, and Sequencing

For RNA-seq, we randomly selected six different batches of chondrocytes per group which were isolated from 37 patients' cartilage tissues as follows: control (CTL) group, IL-1*β*-treated group, IL-1*β*+PRP-treated group, and PRP-treated group (*n* = 6/group). Total RNA was isolated from chondrocytes using TRIzol reagent (Invitrogen, Carlsbad, CA, USA). RNA quality was assessed using an Agilent 2100 Bioanalyzer (Agilent Technologies, Amstelveen, The Netherlands), and RNA quantification was performed using an ND-2000 Spectrophotometer (Thermo Inc., DE, USA). The libraries were prepared from total RNA using the NEBNext Ultra II Directional RNA-Seq Kit (NEW ENGLAND BioLabs, Inc., UK). mRNA was isolated using a Poly(A) RNA Selection Kit (LEXOGEN, Inc., Austria). The isolated mRNAs were used for cDNA synthesis and shearing, following the manufacturer's instructions. Indexing was performed using Illumina indexes 1–12. Enrichment was performed using PCR. Subsequently, the libraries were checked using a TapeStation HS D1000 Screen Tape (Agilent Technologies, Amstelveen, Netherlands) to evaluate the mean fragment size. Quantification was performed using a library quantification kit and StepOne Real-Time PCR System (Life Technologies, Inc., USA). High-throughput sequencing was performed as paired-end 100 sequencing using NovaSeq 6000 (Illumina, Inc., USA).

### 2.5. RNA-seq Data Analysis

Quality control of the raw sequencing data was performed using FastQC (https://www.bioinformatics.babraham.ac.uk/projects/fastqc/). Adapter and low-quality reads (< Q20) were removed using FASTX Trimmer (http://hannonlab.cshl.edu/fastx_toolkit/) and BBMap (https://sourceforge.net/projects/bbmap/). Trimmed reads were mapped to the reference genome using TopHat [21]. Every gene expression value was measured, and those with a fold change greater than 2 or less than 0.5 between groups, along with normalized data (log2) values above 4, were defined as significant genes and selected for further analysis. Gene expression levels were estimated using FPKM (fragments per kilobase of exon per million fragments mapped) by the Cufflinks software [22]. The FPKM values were normalized based on TMM + CPM method using EdgeR [23]. Data mining and graphic visualization were performed using ExDEGA (Ebiogen Inc., Republic of Korea).

### 2.6. STRING Network

Genes showing significant changes in expression were selected and used as inputs for STRING (Search Tool for the Retrieval of Interacting Genes/Proteins; https://string-db.org/). Protein network analysis was performed. The STRING database and web tool STRING are meta-resources that integrate most of the available information on protein–protein associations and scores, weigh, and augment it with predicted interactions, as well as with the results of automatic literature mining searches.

### 2.7. Quantitative Real-Time PCR (qRT-PCR)

Total RNA was extracted using the TRIzol reagent and used for cDNA synthesis. qPCR was performed on a LightCycler 480 System (Roche Diagnostics, Switzerland) using 2× qPCRBIO SyGreen Mix Lo-ROX (PCR Biosystems, London, UK). All data were normalized to *β*-actin expression and analyzed quantitatively. All experiments were repeated at least thrice.

### 2.8. Western Blot Analysis

The cells were lysed using radioimmunoprecipitation assay (RIPA) lysis buffer, separated by 10%–15% sodium dodecyl sulfate-polyacrylamide gel electrophoresis (SDS-PAGE), and transferred to a polyvinylidene fluoride (PVDF) membrane. The membranes were probed with anti-MMP3 (sc21732, Santa Cruz Biotechnology, Dallas, TX, USA), anti-11*β*-HSD1 (AF3397, R&D system, Minneapolis, MN, USA), anti-Ki-67 (MA5-14520, Invitrogen), anti-CDK1/CDK2 (sc53219, Santa Cruz), and anti-GAPDH (sc32233, Santa Cruz) antibodies. Immunoreactive proteins were assessed using a Uvitec Alliance Q9 Micro Image Analyzer (Cambridge CB4 OWS, England, UK). The proteins were quantified by densitometry using the ImageJ software (National Institutes of Health, Bethesda, MD, USA).

### 2.9. Statistical Analyses

RNA-seq data differences between comparison groups were assessed using two-sample *t*-tests.

The qRT-PCR data are expressed as the mean ± standard error. Differences between groups were assessed by *t*-tests (*α* = 0.05) using GraphPad Prism 5.01 (GraphPad Software, La Jolla, CA, USA). Differences were considered significant when *p* values were < 0.05.

## 3. Results

### 3.1. Induction of Cell Differentiation and Proliferation by PRP in Primary Chondrocytes

**Figure 1:**
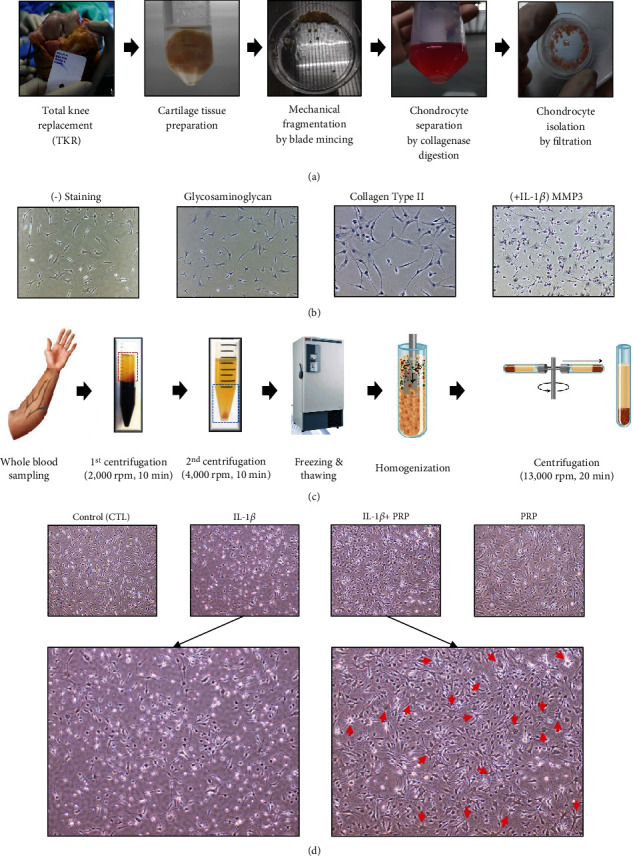
Isolation of human chondrocytes and their differentiation and proliferation by PRP. (a) Overview of chondrocytes isolation procedure. (b) Evaluation of isolated chondrocyte by staining with glycosaminoglycan, anti-collagen type II antibody, and anti-MMP3 antibody in the presence of IL-1*β*. (c) Overview of PRP isolation procedure. (d) Microscopic morphology of chondrocytes after PRP administration in the presence of IL-1*β*.

To evaluate the effect of PRP on chondrocyte physiology, we used human primary chondrocytes isolated from patients who were surgically treated for knee OA by TKR surgery (Figure 1(a)). The isolated chondrocytes revealed the typical phenotype characterized by fibroblast-like morphology and glycosaminoglycan expression as well as type II collagen. The catabolic factor MMP3 was also significantly upregulated by the IL-1*β* challenge leading to cellular damage, which is a well-known response of chondrocytes to the cytokine (Figure 1(b)). As shown in Figure 1(c), PRP was extracted using previously reported methods with slight modifications in a series of preliminary experiments to increase its functional effect on cells. In the presence of PRP, the damaged chondrocytes by IL-1*β* were dramatically recovered, showing significant cell differentiation and proliferation (Figure 1(d)). This suggests that PRP may substantially affect chondrocyte regeneration.

### 3.2. Transcriptome Analysis after PRP Administration in IL-1*β*-Challenged Chondrocytes

**Figure 2:**
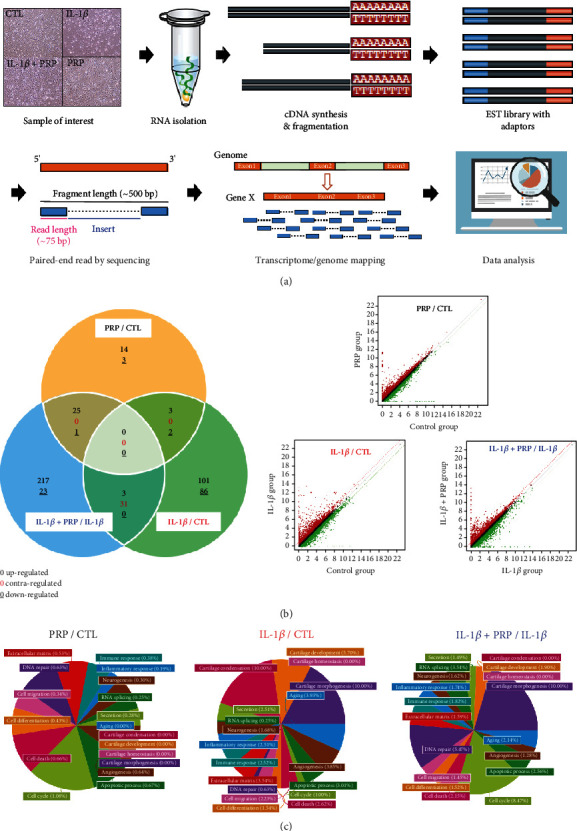
Transcriptome analysis by RNA sequencing (RNA-seq). (a) Overview of RNA-seq process (*n* = 6/group). (b) Left panel: Venn diagram depicting the number of genes up, down, and contraregulated among the significant genes for each group; PRP/control (CTL), IL-1*β*/CTL, and IL-1*β*+PRP/IL-1*β*. Right panel: scatter plot showing the distribution of log2 (normalized data) for each gene between two groups (red: upregulation, green: downregulation) (*n* = 6/group). (c) Percentages of total significant genes according to 18 categories for each group; PRP/control (CTL), IL-1*β*/CTL, and IL-1*β*+PRP/IL-1*β* (*n* = 6/group).

To understand the effect of PRP on chondrocyte physiology, we comprehensively analyzed the transcriptome profile by RNA-seq using total RNA from the CTL group, IL-1*β*-treated group, IL-1*β*+PRP-treated group, and PRP-treated group (*n* = 6/group) and also compared the altered gene expressions between the groups as follows: IL-1*β*/CTL, IL-1*β*+PRP/IL-1*β*, and PRP/CTL (Figure 2(a)). A total of 24,424 genes were analyzed, and significant changes in gene expression were observed for the 226 genes in the IL-1*β*/CTL group, 300 genes in the IL-1*β*+PRP/IL-1*β* group, and 48 genes in the PRP/CTL group (Figure 2(b)). The Venn diagram of 18 gene categories showing significantly different expression in each group revealed that PRP had a substantial and beneficial effect on defective chondrocytes compared to control chondrocytes (Figure 2(c)). Thus, our analysis was focused on the effect of PRP on the IL-1*β* damaged chondrocyte, and we were interested specifically in gene expression of the potent molecules for chondrocyte physiology. Concerning chondrocyte physiology, MMP3 was the most highly induced gene by IL-1*β* (3416.10-fold), while aggrecan (ACAN), the representative anabolic factor for ECM formation in the cartilage, was significantly downregulated (0.24) (Table 1(a)). Obviously, inflammatory cytokine-induced genes such as hydroxysteroid 11-beta dehydrogenase 1 (HSD11B1; 134.45), superoxide dismutase 2 (SOD2: 17.51), and nitric oxide synthase 2 (NOS2: 17.34) were remarkably increased by IL-1*β*. However, the expressions of these IL-1*β*-induced genes, HSD11B1 (134.45–0.19), SOD2 (17.51–0.43), NOS2 (17.34–0.06), and MMP3 (3416.10–0.31) were significantly decreased by PRP in IL-1*β*-treated chondrocytes (Table 1(b), right and Supporting Information 3). Interestingly, PRP administration led to significant upregulation of cell proliferation or differentiation-related genes, such as cell division cycle 20 (CDC20: 0.67–42.83), cyclin B1, -2 (CCNB1: 0.64–35.75; CCNB2: 0.52–24.51), marker of proliferation Ki-67 (MKI67: 0.64–22.73), and cyclin-dependent kinase 1 (CDK1: 0.77–20.53) even in the presence of IL-1*β*, revealing consistency with the result of Figure 1(d) (Table 1(b), left). The Top 20 (*p* < 0.05) upregulated and downregulated genes and their fold-change values in each group (IL-1*β*/CTL and IL-1*β*+PRP/IL-1*β*) are listed in Table 1.

### 3.3. Gene Expression Patterns and Its Correlation Between the Regulated Genes by IL-1*β* and PRP

**Figure 3:**
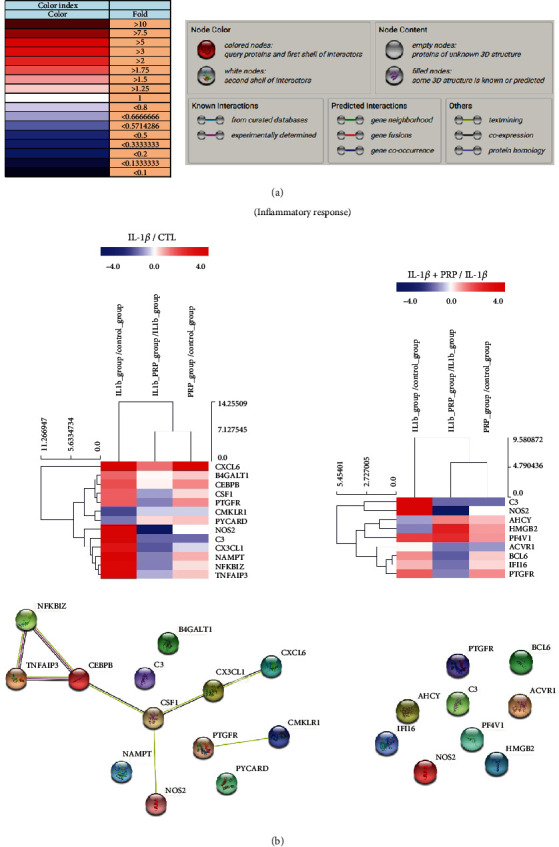
Differential expression patterns in hierarchical clustering and the STRING network for the inflammatory response category. (a) Left panel: different color index showing gene expression fold between two groups. Right panel: information for interpreting the STRING network. (b) Upper panels: differential expression patterns in hierarchical clustering of the altered genes in the inflammatory response category for IL-1*β*/CTL and IL-1*β*+PRP/IL-1*β* group. Lower panel: the STRING network for each altered gene (*n* = 6/group).

To examine the major altered genes which are closely related to chondrocyte physiology in nine categories including inflammatory response, cartilage development, cell proliferation, cell differentiation, cell cycle, cell migration, apoptosis, immune response, and aging, we observed the expression patterns of genes and the protein interaction network, referring to the hierarchical clustering and the STRING (Search Tool for the Retrieval of Interacting Genes/Proteins) network, respectively (Figure 3(a)). In the inflammatory response category, C-X-C motif chemokine ligand 6 (CX3CL1: 7.42), complement component 3 (C3: 23.33), NOS2 (17.34), and TNF alpha–induced protein 3 (TNFAIP3: 12.83) showed a relatively higher expression level by IL-1*β* compared to control (Figure 3(b) left panel and Table 2). Interestingly, these genes were significantly decreased by PRP administration, revealing that PRP has anti-inflammatory effect on the cytokine-damaged chondrocytes (CX3CL1: 7.42 to 0.36; C3: 23.33 to 0.40; NOS2: 17.34 to 0.06; TNFAIP3: 12.83 to 0.66) (Figure 3(b) and Supporting Information 3). In the STRING network depicting the interactions between genes, we observed that CXCL6, CX3CL1, TNFAIP3, and NOS2, known to be related to inflammation, closely interacted with each other (Figure 3(b) lower panel).

**Figure 4:**
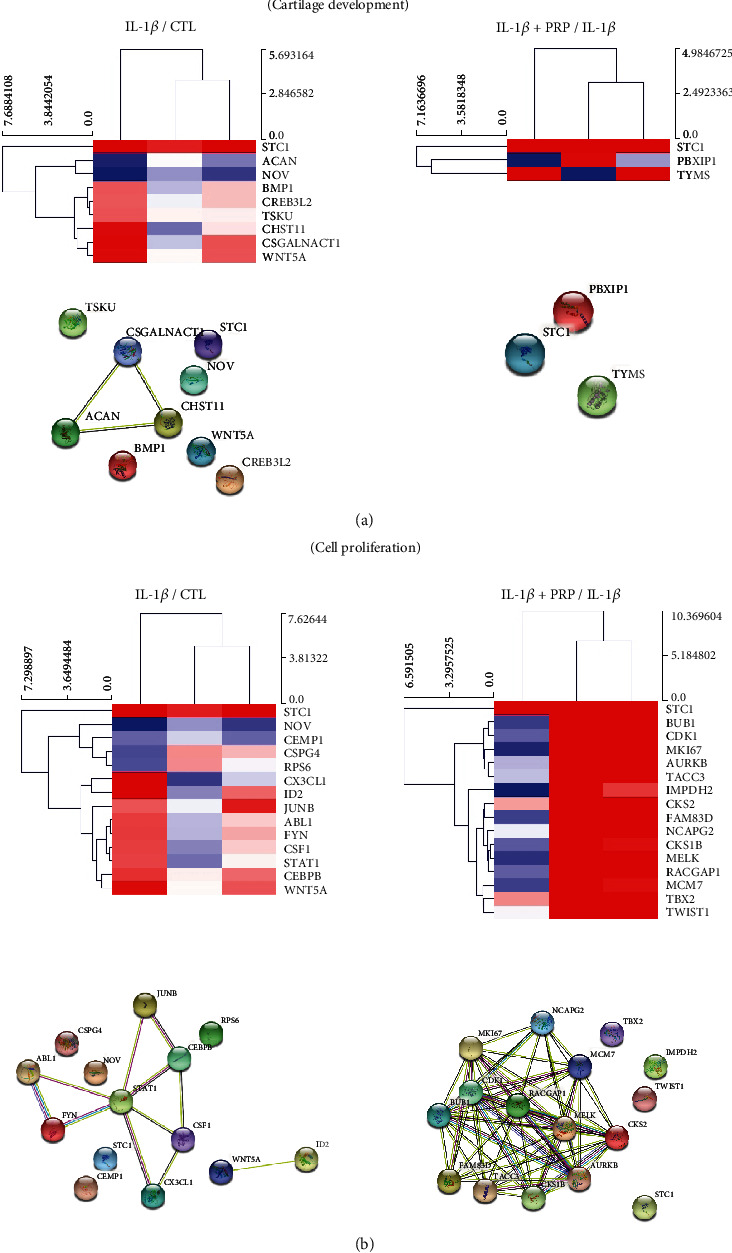
Differential expression patterns in hierarchical clustering and the STRING network for the cartilage development and cell proliferation categories. (a) Differential expression patterns in hierarchical clustering of the altered genes in the cartilage development category for IL-1*β*/CTL and IL-1*β*+PRP/IL-1*β* group. The lower panel shows the STRING network for each altered gene (*n* = 6/group). (b) Differential expression patterns in hierarchical clustering of the altered genes in the cell proliferation category for IL-1*β*/CTL and IL-1*β*+PRP/IL-1*β* group. Lower panel: the STRING network for each altered gene (*n* = 6/group).

In the cartilage development category, stanniocalcin 1 (STC1), carbohydrate (chondroitin 4) sulfotransferase 11 (CHST11), chondroitin sulfate N-acetylgalactosaminyltransferase 1 (CSGALNACT1), and Wnt family member 5A (WNT5A) were remarkably increased by the inflammatory condition, while PRP administration led to significant downregulation of these genes (STC1: 30.79 to 3.92; CHST11: 5.82 to 0.5; CSGALNACT1: 6.48 to 0.77; WNT5A: 5.62 to 1.03) (Figure 4(a) and Supporting Information 3). The string network showing the interactions between decreased ACAN and increased CSGALNACT1 and CHST11 by IL-1 *β* was noticeable (Figure 4(a), lower panel).

Regarding cell proliferation, inflammatory conditions induced the expression of the inhibitor of DNA binding 2 (ID2: 12.51), whereas PRP downregulated ID2 mRNA expression (to 0.58) (Supporting Information 3). In addition, PRP led to significant upregulation of cell cycle-related genes such as MKI67 (22.73), CDK1 (20.53), aurora kinase B (AURKB: 18.26), BUB1 mitotic checkpoint serine/threonine kinase (BUB1: 12.48), and transforming acidic coiled-coil protein 3 (TACC3: 14.38), revealing that PRP has apparent cell proliferative potential (Figure 4(b), right panel and Table 2). These genes also showed a close relationship in the STRING network (Figure 4(b), lower panel).

**Figure 5:**
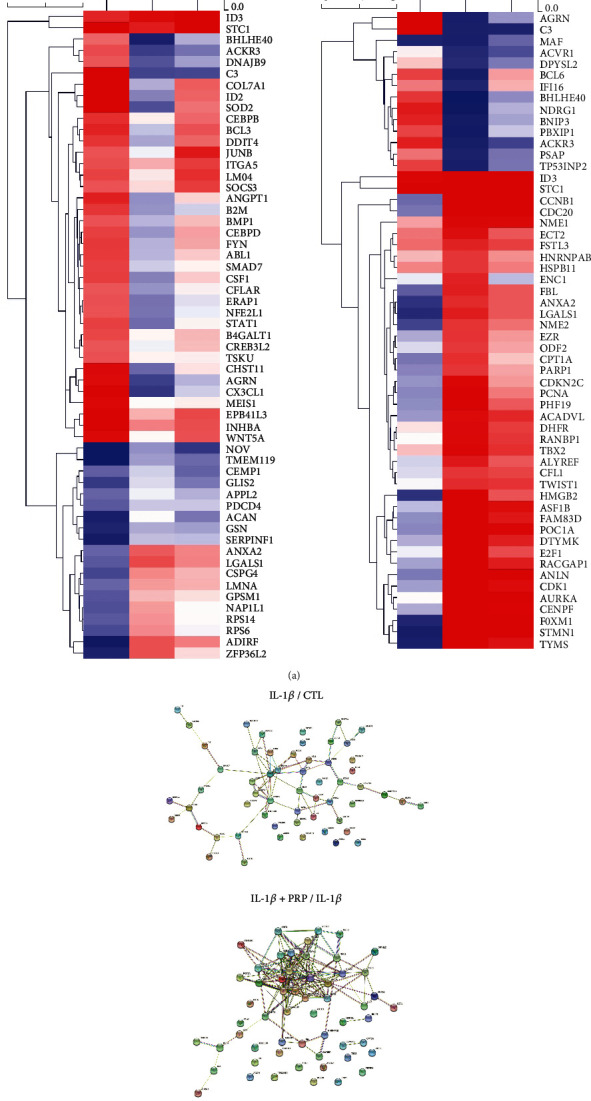
Differential expression patterns in hierarchical clustering and the STRING network for the cell differentiation category. (a) Differential expression patterns in hierarchical clustering of the altered genes in the cell differentiation category for IL-1*β*/CTL and IL-1*β*+PRP/IL-1*β* group. (b) The STRING network for each altered gene (*n* = 6/group).

In the cell differentiation category, agrin (AGRN), collagen type VII alpha 1 (COL7A1), inhibin beta A (INHBA), and meis homeobox 1 (MEIS1) were remarkably increased by the inflammatory condition, while PRP administration led to significant downregulation of these genes (AGRN: 5.45–0.38; COL7A1: 20.26–0.72; INHBA: 8.26–1.74 and MEIS1: 6.22–0.99) (Figure 5(a), Table 2, and Supporting Information 3). In this category, anillin actin–binding protein (ANLN: 0.68–22.37), CCNB1 (0.64–35.75), CDC20 (0.67–42.83), centromere protein F (CENPF: 0.80–15.99), forkhead box M1 (FOXM1: 0.44–17.35), stathmin 1 (STMN1: 0.37 to 18.98), and thymidylate synthetase (TYMS: 0.39–12.98) were significantly induced by PRP administration even in the presence of IL-1 *β*, suggesting that PRP promotes chondrocyte differentiation even in the cell-damaging condition. As shown in Figure 5(b), the string network revealed a close relationship between the upregulated genes. The cell cycle, cell migration, apoptosis, immune response, and aging categories are shown in the Supporting Information 1 and Supporting Information 2, respectively.

### 3.4. Validation of Gene Expression Using Real-Time PCR and Western Blot Analyses

**Figure 6:**
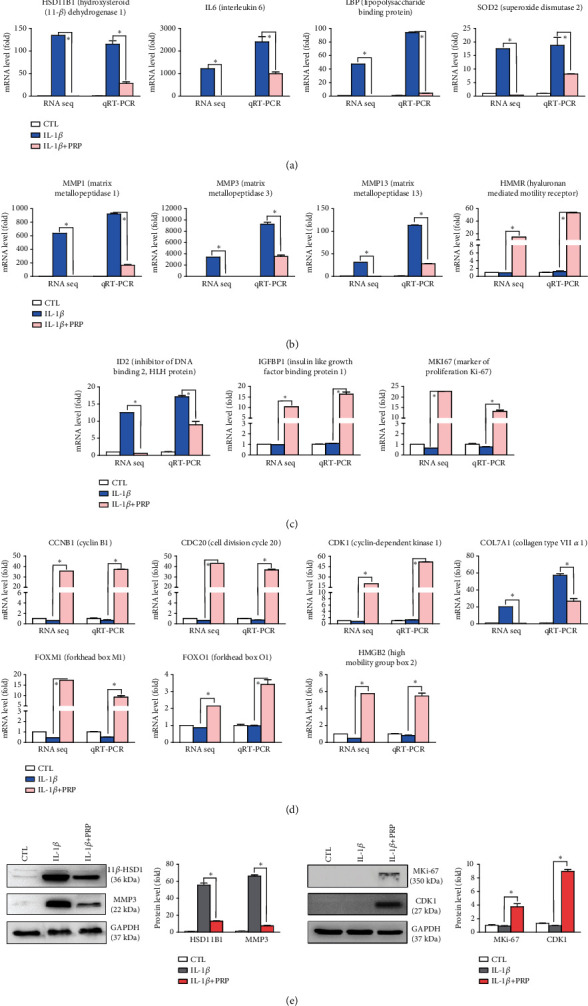
Validation of gene expression using real-time qPCR and western blot analyses. mRNA expression levels of the representative altered genes in the (a) inflammatory response, (b) chondrocyte homeostasis, (c) cell proliferation, and (d) cell differentiation categories for the validation of gene expression using quantitative real-time PCR analysis. The *p* values between two compared groups (IL-1*β*/CTL and IL-1*β*+PRP/IL-1*β*) in (a)–(d) were all < 0.05. (e) Western blot analysis for the representatively selected genes from the results of qRT-PCR analysis. Densitometric analyses of the western blots are shown on the right. Data represent means ± standard errors of the means. ⁣^∗^*p* < 0.05.

To verify the substantial gene expression, we compared the mRNA expression of representative altered genes that are speculated to be closely related to chondrocyte physiology using qRT-PCR analysis. As shown in Figure 6(a), HSD11B1, interleukin 6 (IL6), lipopolysaccharide-binding protein (LBP), and SOD2 in the inflammatory response category were significantly upregulated by IL-1*β* and downregulated by PRP, although the expression levels determined by qRT-PCR were slightly different from the RNA-seq data (Supporting Information 3). With respect to chondrocyte homeostasis, the mRNA expressions of MMP1, MMP3, and MMP13 were remarkably increased by the inflammatory condition with IL-1*β*, while PRP administration led to significant downregulation of these genes. However, in case of hyaluronan–mediated motility receptor (HMMR), its expression was significantly upregulated by PRP administration (Figure 6(b) and Supporting Information 3). The results of the qRT-PCR analyses for ID2, insulin-like growth factor–binding protein 1 (IGF–BP1), and MKI67 from the cell proliferation category and CCNB1, CDC20, CDK1, COL7A1, FOXM1, forkhead box O1 (FOXO1), and high mobility group box 2 (HMGB2) from the cell differentiation category were similar to the results of the RNA-seq analysis (Figures 6(c) and 6(d) and Supporting Information 3). As shown in Figure 6(e), the results of western blot analysis for the selected genes were consistent with those of the RNA-seq and qRT-PCR analyses. Taken together, these results suggested that various genes which are significantly altered in osteoarthritic conditions with IL-1*β* are regulated by PRP administration.

## 4. Discussion

OA, a degenerative disease caused by chronic biomechanical stress, is now recognized as an inflammatory disorder based on recent studies demonstrating that various inflammatory mediators are the ultimate regulators in the pathophysiology of OA [24, 25]. IL-1*β*, a representative OA inducer, is known to be produced by mechanical stress in chondrocytes [26, 27]. IL-1*β* upregulates the expression of aggrecanases and matrix metalloproteinases (MMPs). It induces further inflammatory mediators and the downregulation of chondrogenic ECM synthesis, leading to cartilage ECM degradation [28]. Recently, intra-articular administration of PRP has been considered a potential therapy and tried in knee and hip OA with beneficial effects as an anti-inflammatory and potent regenerative agent [29]. To investigate the effect of PRP on osteoarthritic chondrocytes, we comprehensively analyzed the genetic changes in human chondrocytes following PRP administration in the presence of IL-1*β*. Identifying gene expression patterns of the osteoarthritic chondrocytes after PRP administration in the presence of IL-1*β* would be the first step and a vital determinant in understanding the inflammation–mediated chondrocyte degeneration and improving outcomes of PRP administration.

In this study, IL-1*β*-treated chondrocytes showed a significant increase in the gene expression of various inflammation-related molecules, including CXCL6, CX3CL1, TNFAIP3, NOS2, IL6, and LBP. This inflammatory condition also resulted in an increased expression of the representative catabolic factors MMP3 and MMP13, whereas the expression of the anabolic factor ACAN was significantly decreased, suggesting that inflammation is a direct detrimental cause of chondrocyte damage and cartilage degeneration. Interestingly, PRP revealed noticeable anti-inflammatory effects on arthritic chondrocytes through inverse regulation of the altered genes, even in the presence of IL-1*β*. IL-1*β* exerts its effects through a variety of signaling pathways, including mitogen-activated protein kinase (MAPK), extracellular signal-regulated kinase (ERK), c-Jun N-terminal kinase (JNK), and p38 MAPK and nuclear factor kappa B (NF*κ*B) activation, leading to activation of the transcription factor activator protein 1 (AP-1). These signaling pathways act on various regulatory genes involved in apoptosis, inflammation, other immune responses, and ECM-degrading enzymes [28]. It seems obvious that PRP can directly or indirectly affect the gene expression of anabolic and catabolic factors through the regulation of MAPK or NF*κ*B pathways in chondrocytes. This supports a previous study result demonstrating that PRP inhibits the translocation of NF*κ*B to the nucleus causing inhibition of NF*κ*B-target gene expression, including MMPs [30]. Therefore, since various NF*κ*B inhibitors, such as nonsteroidal anti-inflammatory drugs, are used as pharmacologic agents to treat OA, identifying a potent molecule for inhibition of MAPK or NF*κ*B with PRP would be a promising therapeutic approach for OA.

Most importantly, PRP induced remarkable changes in cell proliferation and differentiation-associated genes, promoting the regeneration of osteoarthritic chondrocytes. Expressions of the classical cell proliferation and differentiation-related genes, such as CDC20, CCNB1, -2, MKI67, AURKB, BUB1, TACC3, CDK1, ANLN, CCNB1, CDC20, CENPF, FOXM1, STMN1, and TYMS were significantly upregulated by PRP even in the presence of IL-1*β*. Unexpectedly, it was interesting to note that the cartilage development-related genes STC1 [31], CHST11 [32], CSGALNACT1 [33], and WNT5A [34] were remarkably increased by IL-1*β*, while PRP administration led to significant downregulation of these genes. However, it seems clear that PRP may be a substantial regulator of cartilage development-associated genes, and it would be meaningful to investigate the molecular mechanisms underlying the regulation of these genes by PRP, especially in the context of inflammatory conditions.

It has been known that PRP secretes various growth factors at high concentrations [35]. With respect to the effector molecule(s) in PRP, it is presumed that various growth factors, including transforming growth factor-*β* (TGF-*β*), platelet-derived growth factor (PDGF), fibroblast growth factor (FGF), insulin-like growth factor (IGF), epidermal growth factor (EGF), and vascular endothelial growth factor (VEGF) play pivotal roles in cell proliferation and cell differentiation of chondrocytes. A previous study demonstrated that concentrated growth factors increase the proliferation and promote the osteogenic differentiation of periosteum-derived cells [36]. Ma et al. reported that TGF*β*1 signaling could induce the proliferation and differentiation of antler chondrocytes through the Notch-Shh-Foxa pathway [37]. Therefore, in future studies, it will be imperative to elucidate the correlation between growth factors and altered genes and to identify the molecular mechanisms underlying the regenerative efficacy of PRP in an inflammatory environment.

Our present study indicates that the PRP, at the gene expression level, has apparent anti-inflammatory, cell proliferative, and regenerative activities in human chondrocytes in the presence of IL-1*β*, which mimics an osteoarthritic environment. As an autogenous biomaterial, PRP is considered free from the risk of various viruses such as HIV and hepatitis. Additionally, immediate preoperative blood collection for PRP preparation is a convenient and time-saving option for patients compared to other therapeutic options for OA. However, despite these beneficial effects, there is no standardized and optimized protocol for PRP preparation, especially regarding the volume of blood processed and the centrifugation time or duration. Thus, more extensive studies are needed to identify and standardize PRP preparation protocols to obtain reproducible and favorable results.

Our study is the first to comprehensively analyze the transcriptome of human chondrocytes in an osteoarthritic environment using blood-derived biomaterials. This finding may help understand gene regulation by inflammatory circumstances and PRP administration, and may further improve various therapeutic approaches for OA by regulating the potent key molecule or its signaling pathway. Nevertheless, this study has several limitations that require consideration. First, pure normal chondrocytes were not used. Because it was difficult to obtain pure normal knee articular cartilage, relatively healthy cartilage tissues were acquired by direct dissection using a surgical blade following TKR surgery. However, we are confident that the healthy cartilage tissues were appropriately defined at our institution by sufficiently skilled and experienced physicians. Second, we used only IL-1*β* as an OA-inducing inflammatory cytokine to observe the effect of PRP. Because IL-1*β* is not the only single critical molecule for OA, we should have investigated the effect of PRP in the presence of other proinflammatory cytokines or adipokines such as TNF*α*, IL-6, leptin, or visfatin. Finally, we did not identify the potent effector molecule(s) regulated by PRP and could not elucidate the molecular mechanisms underlying the regenerative efficacy of PRP in an inflammatory environment. However, because of the study limitations, we only analyzed the transcriptome by PRP and categorized the altered genes into different categories associated with chondrocyte physiology. In-depth research of the potential molecular mechanisms and identification of critical molecules or signal pathways that mediate cartilage regeneration by PRP is needed, and it will represent a promising therapeutic approach for OA.

## 5. Conclusions

▪ Several genes are significantly altered after PRP administration in the damaged chondrocytes by IL-1*β*, and PRP leads to noticeable cellular changes in chondrocytes in the context of cell differentiation and proliferation.
▪ Altered PRP-dependent genes may be important in controlling chondrocyte regeneration following cellular damage.
▪ Identifying potent molecules that regulate cartilage physiology represents a promising therapeutic approach for suppressing cartilage degeneration, especially that caused by inflammation-induced OA.

## Figures and Tables

**(a):** 

**(IL-1*β*/CTL)**					
**Upregulation**			**Downregulation**		
**Gene symbol**	**Description**	**Fold change**	**Gene symbol**	**Description**	**Fold change**
MMP3	Matrix metallopeptidase 3	3416.102	TMEM119	Transmembrane protein 119	0.078
CXCL6	C-X-C motif chemokine ligand 6	1760.002	HSPB6	Heat shock protein family B (small) member 6	0.083
HSD11B1	Hydroxysteroid (11-beta) dehydrogenase 1	134.453	LSP1	Lymphocyte-specific protein 1	0.096
G0S2	G0/G1 switch 2	81.918	ADRA2A	Adrenoceptor alpha 2A	0.099
IFI6	Interferon alpha inducible protein 6	35.987	NOV	Nephroblastoma overexpressed	0.101
CFB	Complement factor B	35.001	LOC730101	Uncharacterized LOC730101	0.120
STC1	Stanniocalcin 1	30.798	KCNK15	Potassium two pore domain channel subfamily K member 15	0.134
C3	Complement component 3	23.334	OMD	Osteomodulin	0.142
NAMPT	Nicotinamide phosphoribosyltransferase	22.429	ADIRF	Adipogenesis regulatory factor	0.165
COL7A1	Collagen type VII alpha 1	20.262	LRRN4CL	LRRN4 C-terminal like	0.203
NDP	Norrie disease (pseudoglioma)	18.324	SERPINF1	Serpin family F member 1	0.217
SOD2	Superoxide dismutase 2, mitochondrial	17.511	GPX3	Glutathione peroxidase 3	0.227
NOS2	Nitric oxide synthase 2	17.339	ACAN	Aggrecan	0.243
IFITM1	Interferon induced transmembrane protein 1	16.433	THBS2	Thrombospondin 2	0.250
TNFAIP3	TNF alpha–induced protein 3	12.833	STK17B	Serine/threonine kinase 17b	0.265
ID2	Inhibitor of DNA binding 2, HLH protein	12.514	ZFP36L2	ZFP36 ring finger protein-like 2	0.265
MT2A	Metallothionein 2A	12.229	ZMAT3	Zinc finger matrin-type 3	0.267
NFKBIZ	NFKB inhibitor zeta	11.508	IFFO1	Intermediate filament family orphan 1	0.270
MT1F	Metallothionein 1F	11.192	GSN	Gelsolin	0.284
PDE4B	Phosphodiesterase 4B	10.953	TPD52L1	Tumor protein D52-like 1	0.291

**(b):** 

**(IL-1*β*+PRP/IL-1*β*)**					
**Upregulation**			**Downregulation**		
**Gene symbol**	**Description**	**Fold change**	**Gene symbol**	**Description**	**Fold change**
CDC20	Cell division cycle 20	42.828	NOS2	Nitric oxide synthase 2	0.062
UBE2C	Ubiquitin-conjugating enzyme E2 C	42.338	CFB	Complement factor B	0.135
CCNB1	cyclin B1	35.750	SNORD10	Small nucleolar RNA, C/D box 10	0.149
BIRC5	Baculoviral IAP repeat containing 5	32.789	HSD11B1	Hydroxysteroid (11-beta) dehydrogenase 1	0.198
CCNB2	Cyclin B2	24.514	SLC2A1	Solute carrier family 2 member 1	0.205
SNORA52	Small nucleolar RNA, H/ACA box 52	23.597	CFD	Complement factor D (adipsin)	0.210
TROAP	Trophinin-associated protein	23.305	BHLHE40	Basic helix-loop-helix family member e40	0.229
PLK1	Polo-like kinase 1	22.988	FBLN5	Fibulin 5	0.243
RRM2	Ribonucleotide reductase regulatory subunit M2	22.979	PRUNE2	Prune homolog 2 (Drosophila)	0.279
SNORA7B	Small nucleolar RNA, H/ACA box 7B	22.826	TRIM22	Tripartite motif containing 22	0.280
MKI67	Marker of proliferation Ki-67	22.728	MXI1	MAX interactor 1, dimerization protein	0.285
ANLN	Anillin actin-binding protein	22.374	NDRG1	N-myc downstream regulated 1	0.285
TK1	Thymidine kinase 1	21.058	CYP7B1	Cytochrome P450 family 7 subfamily B member 1	0.305
DLGAP5	Discs large homolog–associated protein 5	20.736	PFKFB3	6-phosphofructo-2-kinase/fructose-2,6-biphosphatase 3	0.311
CDK1	Cyclin-dependent kinase 1	20.525	C2	Complement component 2	0.316
KIF2C	Kinesin family member 2C	19.568	SLC5A3	Solute carrier family 5 member 3	0.320
TPX2	TPX2, microtubule-associated	19.164	HK2	hexokinase 2	0.327
STMN1	Stathmin 1	18.981	PBXIP1	Pre-B-cell leukemia homeobox interacting protein 1	0.345
PTTG1	Pituitary tumor-transforming 1	18.853	CA12	Carbonic anhydrase 12	0.353
NUSAP1	Nucleolar and spindle-associated protein 1	18.307	BNIP3	BCL2/adenovirus E1B 19 kDa interacting protein 3	0.357

**(a):** 

**Inflammatory response**		
(IL-1*β*/CTL)		
Upregulation		
Gene symbol	Description	Fold change
B4GALT1	Beta-1,4-galactosyltransferase 1	2.524
C3	Complement component 3	23.334
CEBPB	CCAAT/enhancer binding protein beta	3.111
CSF1	Colony-stimulating factor 1	2.684
CX3CL1	C-X3-C motif chemokine ligand 1	7.417
CXCL6	C-X-C motif chemokine ligand 6	1760.002
NAMPT	Nicotinamide phosphoribosyltransferase	22.429
NFKBIZ	NFKB inhibitor zeta	11.508
NOS2	Nitric oxide synthase 2	17.339
PTGFR	Prostaglandin F receptor	2.776
TNFAIP3	TNF alpha-induced protein 3	12.833

Downregulation		
Gene symbol	Description	Fold change
CMKLR1	Chemerin chemokine-like receptor 1	0.310
PYCARD	PYD and CARD domain containing	0.456

(IL-1*β* + PRP/IL-1*β*)		
Upregulation		
Gene symbol	Description	Fold change
AHCY	Adenosylhomocysteinase	2.029
HMGB2	High mobility group box 2	5.778
PF4V1	Platelet factor 4 variant 1	4.747

Downregulation		
Gene symbol	Description	Fold change
ACVR1	Activin A receptor type 1	0.452
BCL6	B-cell CLL/lymphoma 6	0.366
C3	Complement component 3	0.399
IFI16	Interferon gamma inducible protein 16	0.435
NOS2	Nitric oxide synthase 2	0.062
PTGFR	Prostaglandin F receptor	0.488

**(b):** 

**Cartilage development**		
(IL-1*β*/CTL)		
Upregulation		
Gene symbol	Description	Fold change
BMP1	Bone morphogenetic protein 1	2.277
CHST11	Carbohydrate (chondroitin 4) sulfotransferase 11	5.822
CREB3L2	cAMP responsive element binding protein 3-like 2	2.253
CSGALNACT1	Chondroitin sulfate N-acetylgalactosaminyltransferase 1	6.486
STC1	Stanniocalcin 1	30.798
TSKU	Tsukushi, small leucine–rich proteoglycan	2.307
WNT5A	Wnt family member 5A	5.624

Downregulation		
Gene symbol	Description	Fold change
ACAN	Aggrecan	0.243
NOV	Nephroblastoma overexpressed	0.101

(IL-1*β* + PRP/IL-1*β*)		
Upregulation		
Gene symbol	Description	Fold change
STC1	Stanniocalcin 1	3.925
TYMS	Thymidylate synthetase	12.980

Downregulation		
Gene symbol	Description	Fold change
PBXIP1	Pre-B-cell leukemia homeobox interacting protein 1	0.345

**(c):** 

**Cell proliferation**		
(IL-1*β*/CTL)		
Upregulation		
Gene symbol	Description	Fold change
ABL1	ABL proto-oncogene 1, nonreceptor tyrosine kinase	2.781
CEBPB	CCAAT/enhancer binding protein beta	3.111
CSF1	colony stimulating factor 1	2.684
CX3CL1	C-X3-C motif chemokine ligand 1	7.417
FYN	FYN proto-oncogene, Src family tyrosine kinase	2.816
ID2	Inhibitor of DNA binding 2, HLH protein	12.514
JUNB	JunB proto-oncogene, AP-1 transcription factor subunit	2.284
STAT1	Signal transducer and activator of transcription 1	2.629
STC1	Stanniocalcin 1	30.798
WNT5A	Wnt family member 5A	5.624

Downregulation		
Gene symbol	Description	Fold change
CEMP1	Cementum protein 1	0.480
CSPG4	Chondroitin sulfate proteoglycan 4	0.410
NOV	Nephroblastoma overexpressed	0.101
RPS6	Ribosomal protein S6	0.424

(IL-1*β*+PRP/IL-1*β*)		
Upregulation		
Gene symbol	Description	Fold change
AURKB	Aurora kinase B	18.261
BUB1	BUB1 mitotic checkpoint serine/threonine kinase	14.994
CDK1	Cyclin-dependent kinase 1	20.525
CKS1B	CDC28 protein kinase regulatory subunit 1B	6.024
CKS2	CDC28 protein kinase regulatory subunit 2	8.154
FAM83D	Family with sequence similarity 83 member D	9.940
IMPDH2	IMP (inosine 5'-monophosphate) dehydrogenase 2	2.201
MCM7	Minichromosome maintenance complex component 7	3.830
MELK	Maternal embryonic leucine zipper kinase	6.915
MKI67	Marker of proliferation Ki-67	22.728
NCAPG2	Non-SMC condensin II complex subunit G2	5.674
RACGAP1	Rac GTPase activating protein 1	7.297
STC1	Stanniocalcin 1	3.925
TACC3	Transforming acidic coiled-coil–containing protein 3	14.376
TBX2	T-box 2	3.576
TWIST1	Twist family bHLH transcription factor 1	2.155

Downregulation		
None		

**Table:** (d) The major altered genes in the cell differentiation category

**Cell differentiation**					
**(IL-1*β*/CTL)**			**(IL-1*β*+PRP/IL-1*β*)**		
Upregulation			Upregulation		
Gene symbol	Description	Fold change	Gene symbol	Description	Fold change
ABL1	ABL proto-oncogene 1, nonreceptor tyrosine kinase	2.781	ACADVL	Acyl-CoA dehydrogenase, very long chain	3.102
ACKR3	Atypical chemokine receptor 3	2.429	ALYREF	Aly/REF export factor	2.582
AGRN	Agrin	5.454	ANLN	Anillin actin-binding protein	22.374
ANGPT1	Angiopoietin 1	3.685	ANXA2	Annexin A2	2.160
B2M	Beta-2-microglobulin	2.841	ASF1B	Anti-silencing function 1B histone chaperone	9.485
B4GALT1	Beta-1,4-galactosyltransferase 1	2.524	AURKA	Aurora kinase A	15.539
BCL3	B-cell CLL/lymphoma 3	3.584	CCNB1	cyclin B1	35.750
BHLHE40	Basic helix-loop-helix family member e40	2.019	CDC20	Cell division cycle 20	42.828
BMP1	Bone morphogenetic protein 1	2.277	CDK1	Cyclin-dependent kinase 1	20.525
C3	Complement component 3	23.334	CDKN2C	Cyclin-dependent kinase inhibitor 2C	3.452
CEBPB	CCAAT/enhancer binding protein beta	3.111	CENPF	Centromere protein F	15.986
CEBPD	CCAAT/enhancer binding protein delta	2.646	CFL1	Cofilin 1	2.021
CFLAR	CASP8 and FADD-like apoptosis regulator	2.256	CPT1A	Carnitine palmitoyltransferase 1A	2.112
CHST11	Carbohydrate (chondroitin 4) sulfotransferase 11	5.822	DHFR	Dihydrofolate reductase	3.238
COL7A1	Collagen type VII alpha 1	20.262	DTYMK	Deoxythymidylate kinase	4.705
CREB3L2	cAMP responsive element binding protein 3-like 2	2.253	E2F1	E2F transcription factor 1	5.955
CSF1	Colony-stimulating factor 1	2.684	ECT2	Epithelial cell transforming 2	2.920
CX3CL1	C-X3-C motif chemokine ligand 1	7.417	ENC1	Ectodermal-neural cortex 1	2.324
DDIT4	DNA damage–inducible transcript 4	2.920	EZR	Ezrin	2.072
DNAJB9	DnaJ heat shock protein family (Hsp40) member B9	2.229	FAM83D	Family with sequence similarity 83 member D	9.940
EPB41L3	Erythrocyte membrane protein band 4.1-like 3	9.257	FBL	Fibrillarin	2.403
ERAP1	Endoplasmic reticulum aminopeptidase 1	2.309	FOXM1	Forkhead box M1	17.345
FYN	FYN proto-oncogene, Src family tyrosine kinase	2.816	FSTL3	Follistatin-like 3	2.314
ID2	Inhibitor of DNA-binding 2, HLH protein	12.514	HMGB2	High mobility group box 2	5.778
ID3	Inhibitor of DNA-binding 3, HLH protein	3.437	HNRNPAB	Heterogeneous nuclear ribonucleoprotein A/B	2.020
INHBA	Inhibin beta A	8.268	HSPB11	Heat shock protein family B (small) member 11	2.011
ITGA5	Integrin subunit alpha 5	2.367	ID3	Inhibitor of DNA binding 3, HLH protein	5.720
JUNB	JunB proto-oncogene, AP-1 transcription factor subunit	2.284	LGALS1	Lectin, galactoside binding soluble 1	2.463
LMO4	LIM domain only 4	2.616	NME1	NME/NM23 nucleoside diphosphate kinase 1	3.310
MEIS1	Meis homeobox 1	6.228	NME2	NME/NM23 nucleoside diphosphate kinase 2	2.048
NFE2L1	Nuclear factor, erythroid 2 like 1	2.280	ODF2	Outer dense fiber of sperm tails 2	2.000
SMAD7	SMAD family member 7	2.645	PARP1	Poly (ADP-ribose) polymerase 1	2.018
SOCS3	Suppressor of cytokine signaling 3	2.276	PCNA	Proliferating cell nuclear antigen	3.833
SOD2	Superoxide dismutase 2, mitochondrial	17.511	PHF19	PHD finger protein 19	4.142
STAT1	Signal transducer and activator of transcription 1	2.629	POC1A	POC1 centriolar protein A	10.832
STC1	Stanniocalcin 1	30.798	RACGAP1	Rac GTPase activating protein 1	7.297
TSKU	Tsukushi, small leucine–rich proteoglycan	2.307	RANBP1	RAN binding protein 1	3.277
WNT5A	Wnt family member 5A	5.624	STC1	Stanniocalcin 1	3.925
Downregulation			STMN1	Stathmin 1	18.981
Gene symbol	Description	Fold change	TBX2	T-box 2	3.576
ACAN	Aggrecan	0.243	TWIST1	Twist family bHLH transcription factor 1	2.155
ADIRF	Adipogenesis regulatory factor	0.165	TYMS	Thymidylate synthetase	12.980
ANXA2	Annexin A2	0.496	Downregulation		
APPL2	Adaptor protein, phosphotyrosine interacting with pH domain and leucine zipper 2	0.491	Gene symbol	Description	Fold change
CEMP1	Cementum protein 1	0.480	ACKR3	Atypical chemokine receptor 3	0.380
CSPG4	Chondroitin sulfate proteoglycan 4	0.410	ACVR1	Activin A receptor type 1	0.452
GLIS2	GLIS family zinc finger 2	0.368	AGRN	Agrin	0.385
GPSM1	G-protein signaling modulator 1	0.452	BCL6	B-cell CLL/lymphoma 6	0.366
GSN	Gelsolin	0.284	BHLHE40	Basic helix-loop-helix family member e40	0.229
LGALS1	Lectin, galactoside-binding soluble 1	0.464	BNIP3	BCL2/adenovirus E1B 19 kDa interacting protein 3	0.357
LMNA	Lamin A/C	0.499	C3	Complement component 3	0.399
NAP1L1	Nucleosome assembly protein 1-like 1	0.437	DPYSL2	Dihydropyrimidinase like 2	0.469
NOV	Nephroblastoma overexpressed	0.101	IFI16	Interferon gamma inducible protein 16	0.435
PDCD4	Programmed cell death 4 (neoplastic transformation inhibitor)	0.463	MAF	V-maf avian musculoaponeurotic fibrosarcoma oncogene homolog	0.389
RPS14	Ribosomal protein S14	0.461	NDRG1	N-myc downstream regulated 1	0.285
RPS6	Ribosomal protein S6	0.424	PBXIP1	Pre-B-cell leukemia homeobox interacting protein 1	0.345
SERPINF1	Serpin family F member 1	0.217	PSAP	Prosaposin	0.414
TMEM119	Transmembrane protein 119	0.078	TP53INP2	Tumor protein p53 inducible nuclear protein 2	0.390
ZFP36L2	ZFP36 ring finger protein-like 2	0.265			

## Data Availability

The data used to support the findings of this study are available from the corresponding author upon request.

## Supporting Information

Supporting Information 1 Supporting Figures: hierarchical clustering and the STRING network of the altered genes between each comparing group in the cell cycle, cell migration, apoptosis, immune response, and aging categories.

Supporting Information 2 Supporting Tables: the major altered genes between each comparing group in the cell cycle, cell migration, apoptosis, immune response, and aging categories.

Supporting Information 3 Supporting Dataset File: the transcriptome profile by RNA sequencing (RNA-seq).
