# Supplementary material for: Transcriptome Analysis of Platelet-Rich Plasma–Treated Osteoarthritic Chondrocyte
Source: Biomed Res Int. 2024 Nov 21;2024:7680736. doi: 10.1155/2024/7680736 (PMC11604281; doi:10.1155/2024/7680736)
Supplement: Supporting Information 1 — Supporting Figures: hierarchical clustering and the STRING network of the altered genes between each comparing group in the cell cycle, cell migration, apoptosis, immune response, and aging categories. [file 7680736.f1.pdf]

Supplementary Fig. 1

a

IL-1β / CTL (Cell cycle)

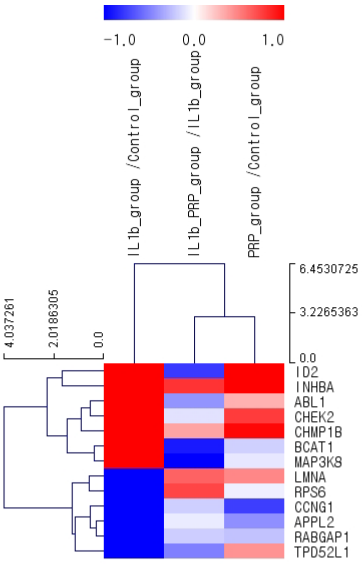

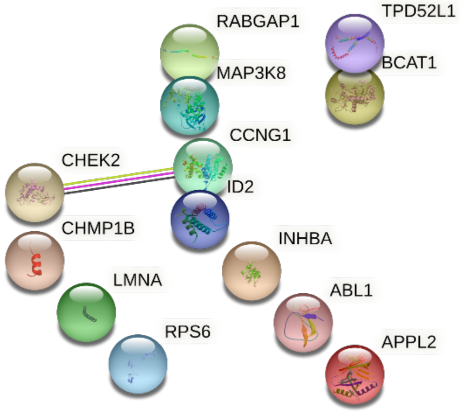

b

IL-1β + PRP / IL-1β (Cell cycle)

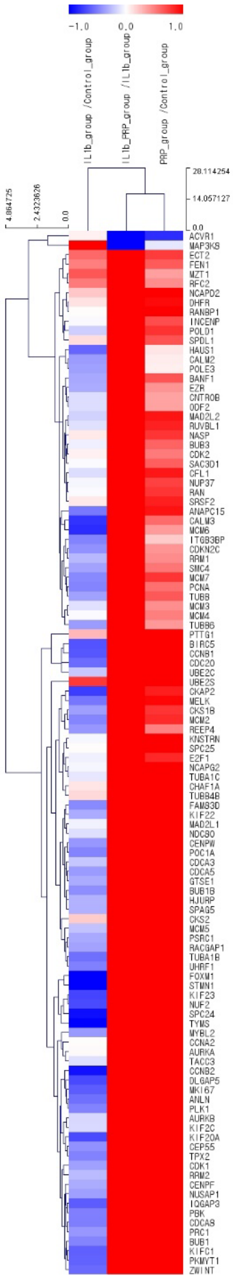

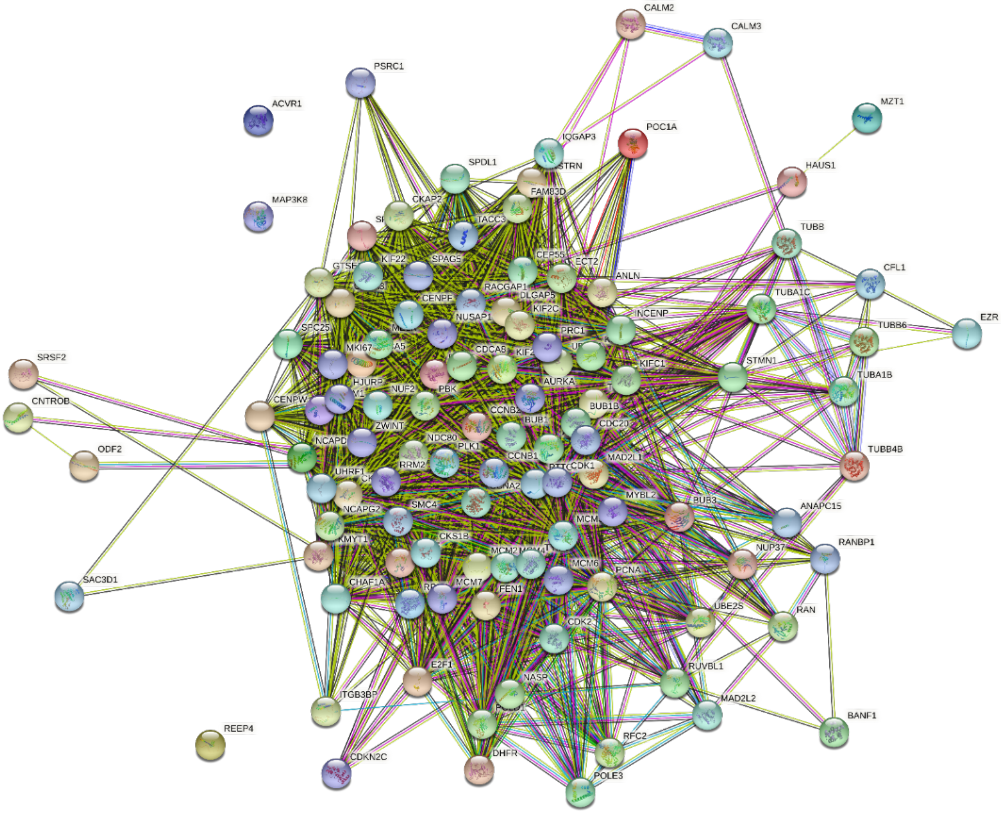

Supplementary Fig. 2

a

IL-1β / CTL (Cell migration)

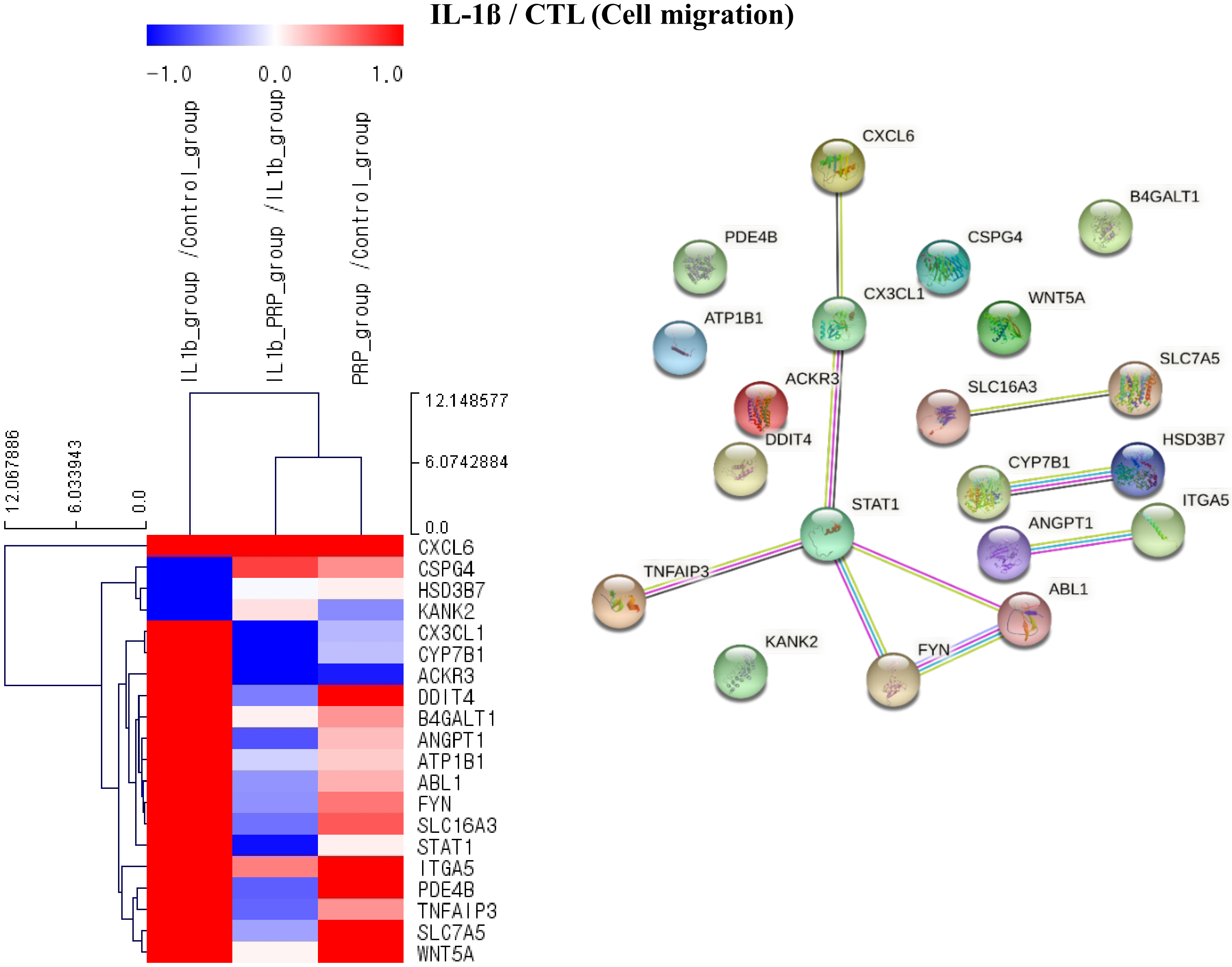

b

IL-1β + PRP / IL-1β (Cell migration)

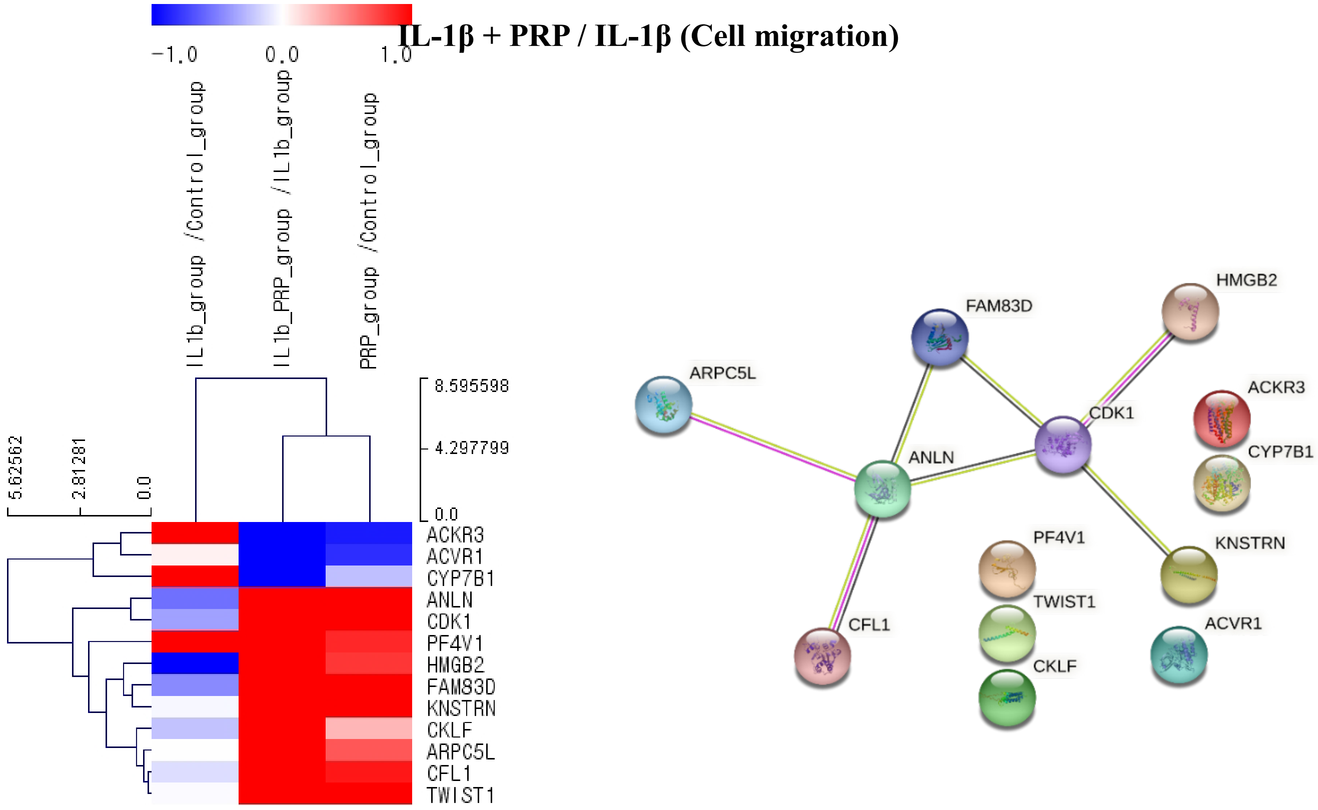

Supplementary Fig. 3

a

IL-1β / CTL (Apoptosis)

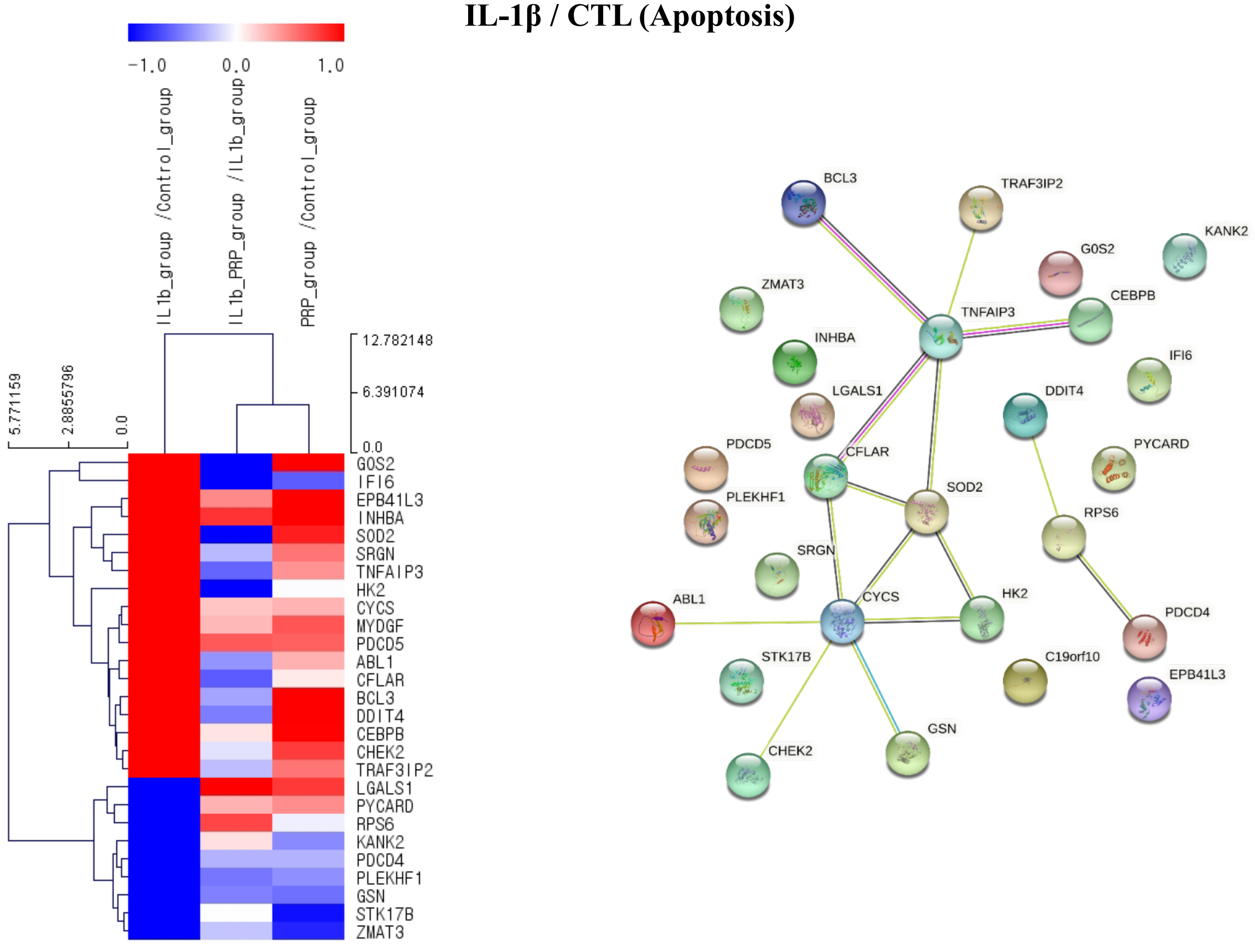

b

IL-1β + PRP / IL-1β (Apoptosis)

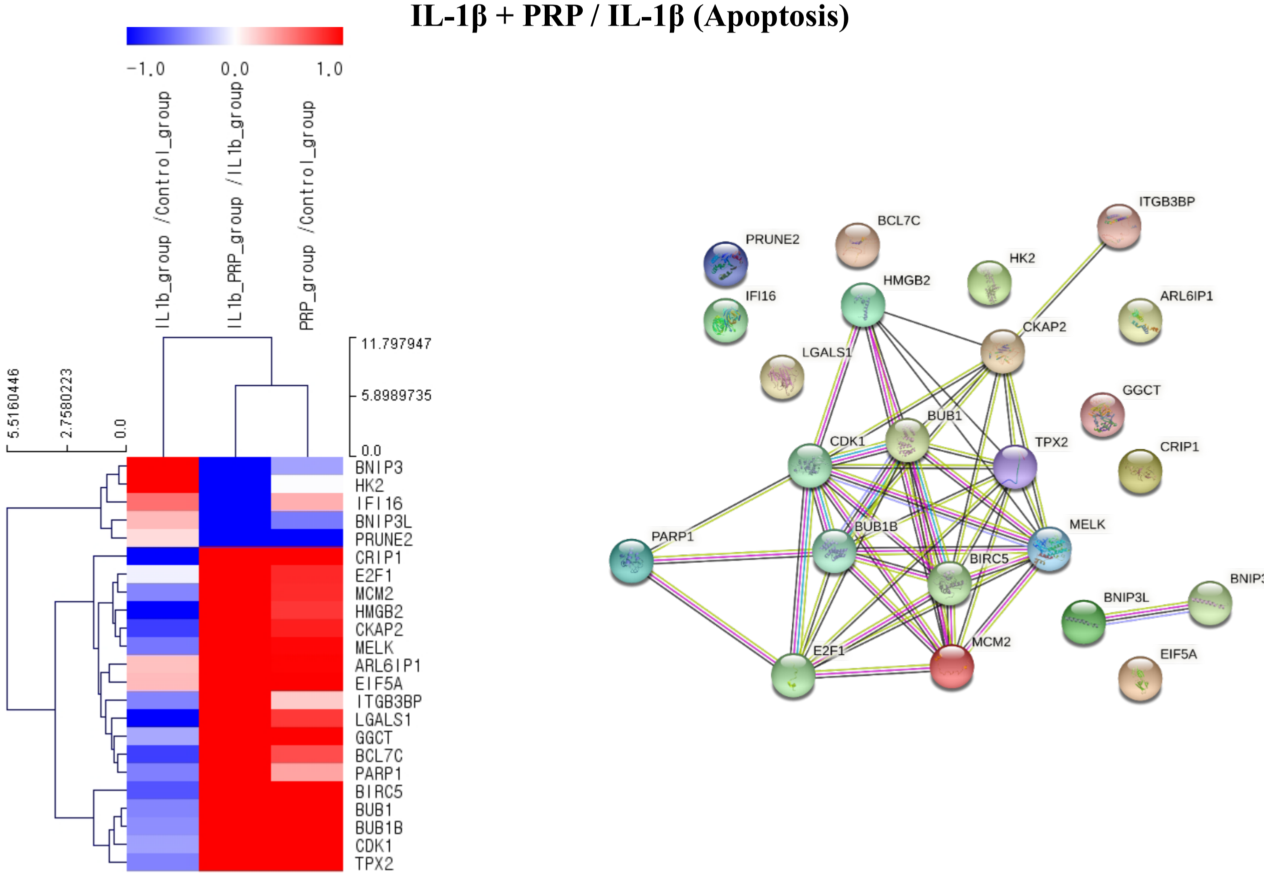

Supplementary Fig. 4

a

IL-1β / CTL (Immune response)

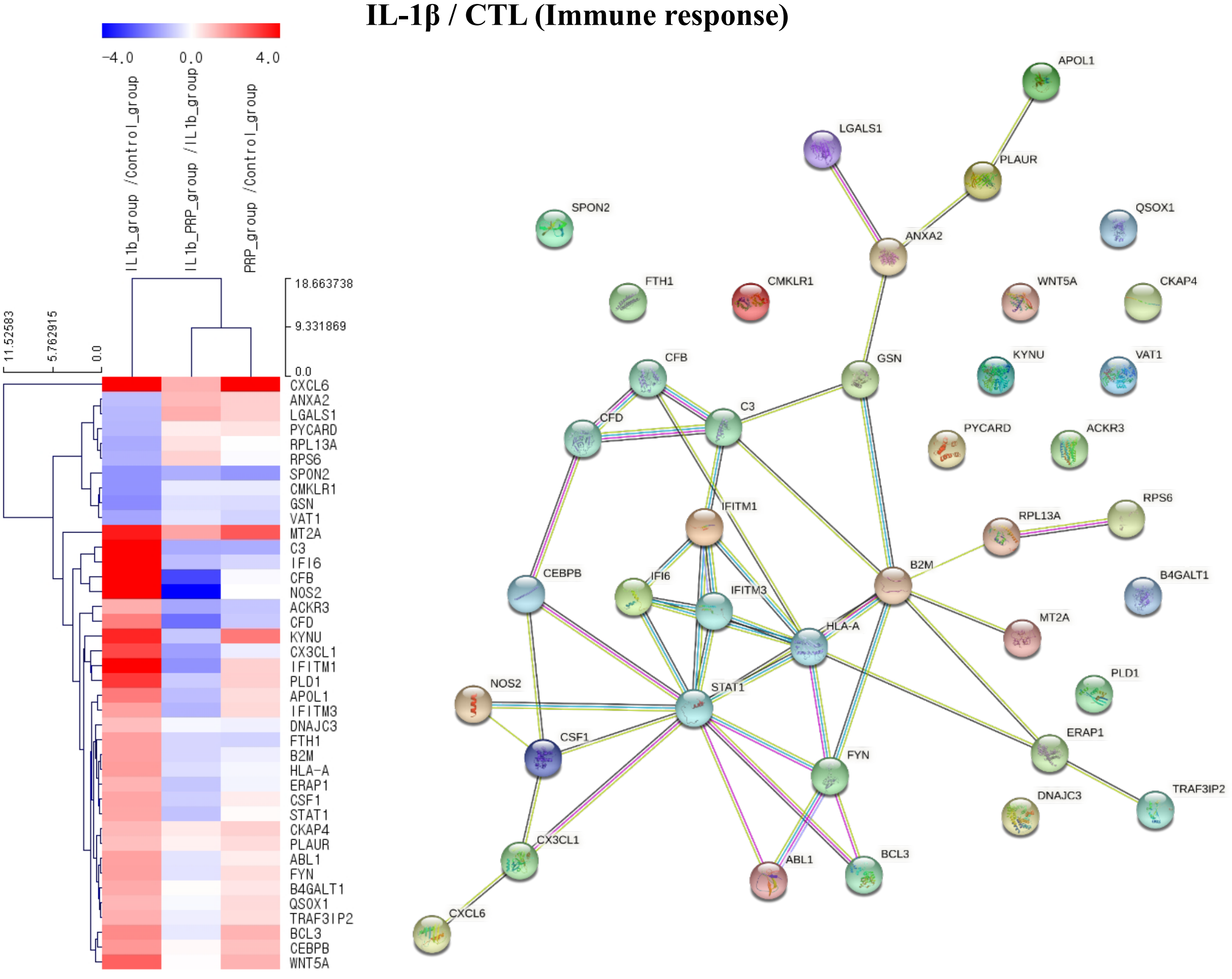

b

IL-1β + PRP / IL-1β (Immune response)

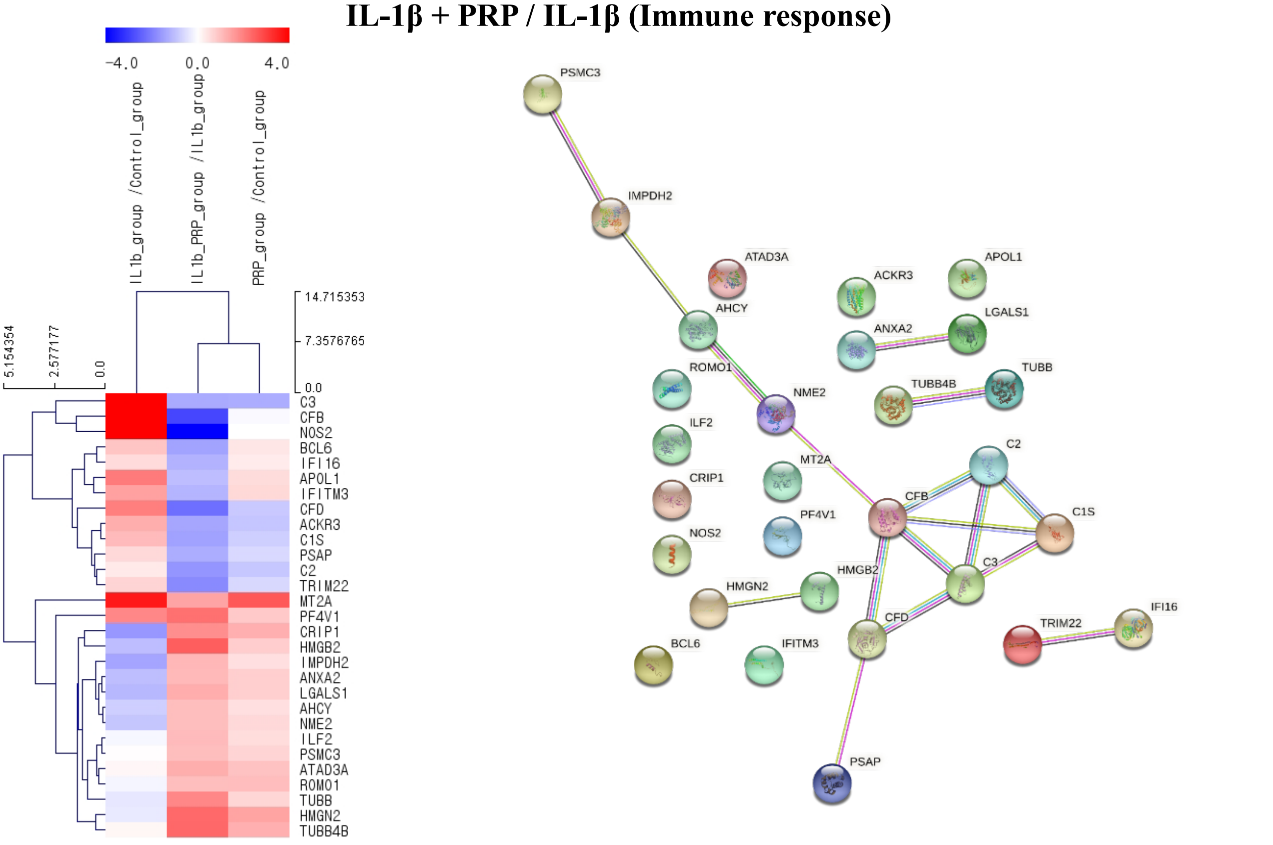

Supplementary Fig. 5

a

IL-1β / CTL (Aging)

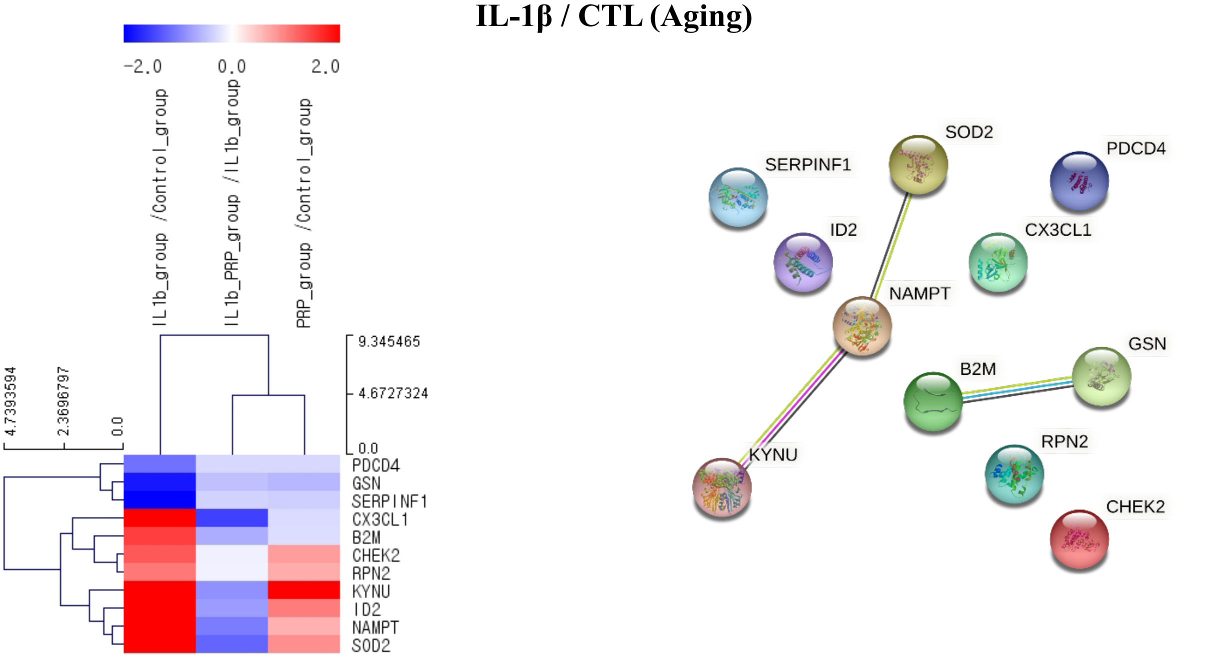

b

IL-1β + PRP / IL-1β (Aging)

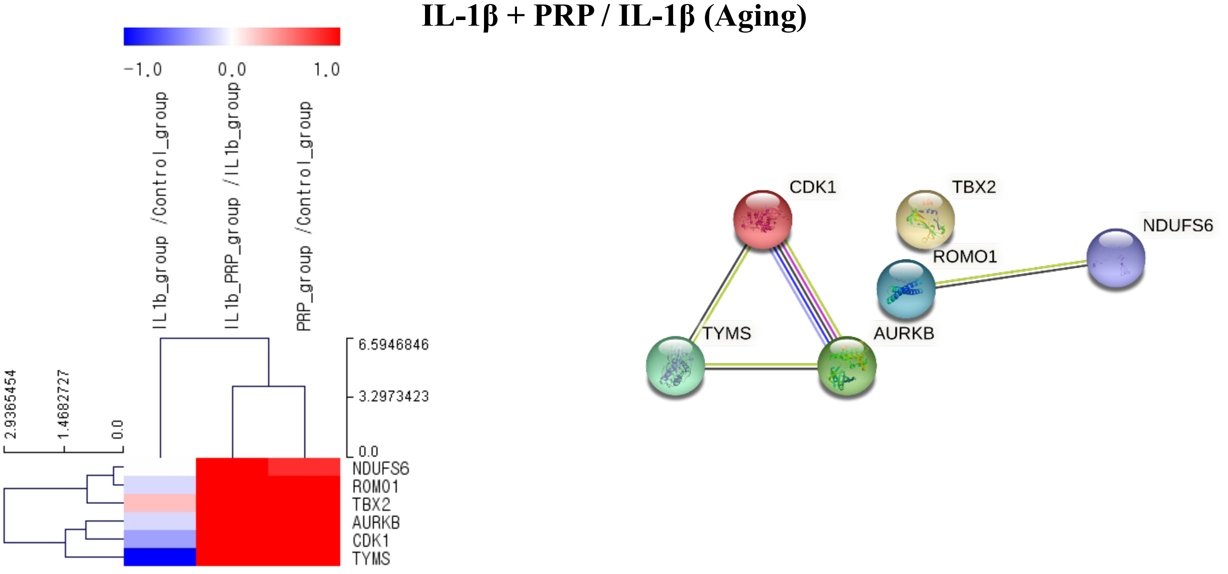

Supplementary Fig. 1. Differential expression patterns in hierarchical clustering and the STRING network for the cell cycle category. Left panels: Differential expression patterns in hierarchical clustering of the altered genes in the cell cycle category for IL-1β/CTL (a) and IL-1β+PRP/ IL-1β (b) group. Right panels: the STRING network for each altered gene.

Supplementary Fig. 2. Differential expression patterns in hierarchical clustering and the STRING network for the cell migration category. Left panels: Differential expression patterns in hierarchical clustering of the altered genes in the cell migration category for IL-1β/CTL (a) and IL-1β+PRP/ IL-1β (b) group. Right panels: the STRING network for each altered gene.

Supplementary Fig. 3. Differential expression patterns in hierarchical clustering and the STRING network for the apoptosis category. Left panels: Differential expression patterns in hierarchical clustering of the altered genes in the apoptosis category for IL-1β/CTL (a) and IL-1β+PRP/ IL-1β (b) group. Right panels: the STRING network for each altered gene.

Supplementary Fig. 4. Differential expression patterns in hierarchical clustering and the STRING network for the immune response category. Left panels: Differential expression patterns in hierarchical clustering of the altered genes in the immune response category for IL-1β/CTL (a) and IL-1β+PRP/ IL-1β (b) group. Right panels: the STRING network for each altered gene.

Supplementary Fig. 5. Differential expression patterns in hierarchical clustering and the STRING network for the aging category. Left panels: Differential expression patterns in hierarchical clustering of the altered genes in the aging category for IL-1β/CTL (a) and IL-1β+PRP/ IL-1β (b) group. Right panels: the STRING network for each altered gene.
