# Supplementary material for: Transcriptome Analysis of Platelet-Rich Plasma–Treated Osteoarthritic Chondrocyte
Source: Biomed Res Int. 2024 Nov 21;2024:7680736. doi: 10.1155/2024/7680736 (PMC11604281; doi:10.1155/2024/7680736)
Supplement: Supporting Information 2 — Supporting Tables: the major altered genes between each comparing group in the cell cycle, cell migration, apoptosis, immune response, and aging categories. [file 7680736.f2.pdf]

# Supplementary Table 1-1. The major altered genes in cell cycle category.

## Cell cycle

### IL-1β / CTL

**Up regulation**

| Gene symbol | Description | Fold change |
|---|---|---|
| ABL1 | ABL proto-oncogene 1, non-receptor tyrosine kinase | 2.781 |
| BCAT1 | branched chain amino acid transaminase 1 | 2.712 |
| CHEK2 | checkpoint kinase 2 | 2.484 |
| CHMP1B | charged multivesicular body protein 1B | 3.673 |
| ID2 | inhibitor of DNA binding 2, HLH protein | 12.514 |
| INHBA | inhibin beta A | 8.268 |
| MAP3K8 | mitogen-activated protein kinase kinase kinase 8 | 2.950 |

**Down regulation**

| Gene symbol | Description | Fold change |
|---|---|---|
| APPL2 | adaptor protein, phosphotyrosine interacting with PH domain and leucine zipper 2 | 0.491 |
| CCNG1 | cyclin G1 | 0.367 |
| LMNA | lamin A/C | 0.499 |
| RABGAP1 | RAB GTPase activating protein 1 | 0.421 |
| RPS6 | ribosomal protein S6 | 0.424 |
| TPD52L1 | tumor protein D52-like 1 | 0.291 |

### IL-1β + PRP / IL-1β

**Up regulation**

| Gene symbol | Description | Fold change |
|---|---|---|
| ANAPC15 | anaphase promoting complex subunit 15 | 2.757 |
| ANLN | anillin actin binding protein | 22.374 |
| AURKA | aurora kinase A | 15.539 |
| AURKB | aurora kinase B | 18.261 |
| BANF1 | barrier to autointegration factor 1 | 2.070 |
| BIRC5 | baculoviral IAP repeat containing 5 | 32.789 |
| BUB1 | BUB1 mitotic checkpoint serine/threonine kinase | 14.994 |
| BUB1B | BUB1 mitotic checkpoint serine/threonine kinase B | 12.479 |
| BUB3 | BUB3, mitotic checkpoint protein | 2.666 |
| CALM2 | calmodulin 2 (phosphorylase kinase, delta) | 2.161 |
| CALM3 | calmodulin 3 (phosphorylase kinase, delta) | 2.354 |
| CCNA2 | cyclin A2 | 10.966 |
| CCNB1 | cyclin B1 | 35.750 |
| CCNB2 | cyclin B2 | 24.514 |
| CDC20 | cell division cycle 20 | 42.828 |
| CDCA3 | cell division cycle associated 3 | 11.860 |
| CDCA5 | cell division cycle associated 5 | 12.528 |
| CDCA8 | cell division cycle associated 8 | 15.391 |
| CDK1 | cyclin-dependent kinase 1 | 20.525 |
| CDK2 | cyclin-dependent kinase 2 | 2.575 |
| CDKN2C | cyclin-dependent kinase inhibitor 2C | 3.452 |
| CENPF | centromere protein F | 15.986 |
| CENPW | centromere protein W | 9.943 |
| CEP55 | centrosomal protein 55 | 17.471 |
| CFL1 | cofilin 1 | 2.021 |
| CHAF1A | chromatin assembly factor 1 subunit A | 4.492 |
| CKAP2 | cytoskeleton associated protein 2 | 7.835 |
| CKS1B | CDC28 protein kinase regulatory subunit 1B | 6.024 |
| CKS2 | CDC28 protein kinase regulatory subunit 2 | 8.154 |
| CNTROB | centrobin, centriole duplication and spindle assembly protein | 2.133 |
| DHFR | dihydrofolate reductase | 3.238 |
| DLGAP5 | discs large homolog associated protein 5 | 20.736 |
| E2F1 | E2F transcription factor 1 | 5.955 |
| ECT2 | epithelial cell transforming 2 | 2.920 |
| EZR | ezrin | 2.072 |
| FAM83D | family with sequence similarity 83 member D | 9.940 |
| FEN1 | flap structure-specific endonuclease 1 | 3.375 |
| FOXM1 | forkhead box M1 | 17.345 |
| GTSE1 | G2 and S-phase expressed 1 | 13.452 |
| HAUS1 | HAUS augmin like complex subunit 1 | 2.363 |
| HJURP | Holliday junction recognition protein | 12.395 |
| INCENP | inner centromere protein | 4.424 |
| IQGAP3 | IQ motif containing GTPase activating protein 3 | 17.654 |
| ITGB3BP | integrin subunit beta 3 binding protein | 3.597 |
| KIF20A | kinesin family member 20A | 17.216 |
| KIF22 | kinesin family member 22 | 8.982 |
| KIF23 | kinesin family member 23 | 13.233 |
| KIF2C | kinesin family member 2C | 19.568 |
| KIFC1 | kinesin family member C1 | 15.394 |
| KNSTRN | kinetochore-localized astrin/SPAG5 binding protein | 6.595 |
| MAD2L1 | MAD2 mitotic arrest deficient-like 1 (yeast) | 9.230 |
| MAD2L2 | MAD2 mitotic arrest deficient-like 2 (yeast) | 2.417 |
| MCM2 | minichromosome maintenance complex component 2 | 5.517 |
| MCM3 | minichromosome maintenance complex component 3 | 3.002 |
| MCM4 | minichromosome maintenance complex component 4 | 2.888 |
| MCM5 | minichromosome maintenance complex component 5 | 6.804 |
| MCM6 | minichromosome maintenance complex component 6 | 2.868 |
| MCM7 | minichromosome maintenance complex component 7 | 3.830 |
| MELK | maternal embryonic leucine zipper kinase | 6.915 |
| MKI67 | marker of proliferation Ki-67 | 22.728 |
| MYBL2 | MYB proto-oncogene like 2 | 12.748 |
| MZT1 | mitotic spindle organizing protein 1 | 2.439 |
| NASP | nuclear autoantigenic sperm protein | 2.641 |
| NCAPD2 | non-SMC condensin I complex subunit D2 | 3.035 |
| NCAPG2 | non-SMC condensin II complex subunit G2 | 5.674 |
| NDC80 | NDC80 kinetochore complex component | 10.474 |
| NUF2 | NUF2, NDC80 kinetochore complex component | 14.080 |
| NUP37 | nucleoporin 37kDa | 2.027 |
| NUSAP1 | nucleolar and spindle associated protein 1 | 18.307 |
| ODF2 | outer dense fiber of sperm tails 2 | 2.000 |
| PBK | PDZ binding kinase | 17.774 |
| PCNA | proliferating cell nuclear antigen | 3.833 |
| PKMYT1 | protein kinase, membrane associated tyrosine/threonine 1 | 13.923 |
| PLK1 | polo-like kinase 1 | 22.988 |
| POC1A | POC1 centriolar protein A | 10.832 |
| POLD1 | polymerase (DNA) delta 1, catalytic subunit | 3.712 |
| POLE3 | polymerase (DNA) epsilon 3, accessory subunit | 2.029 |
| PRC1 | protein regulator of cytokinesis 1 | 16.320 |
| PSRC1 | proline and serine rich coiled-coil 1 | 6.698 |
| PTTG1 | pituitary tumor-transforming 1 | 18.853 |
| RACGAP1 | Rac GTPase activating protein 1 | 7.297 |
| RAN | RAN, member RAS oncogene family | 2.251 |
| RANBP1 | RAN binding protein 1 | 3.277 |
| REEP4 | receptor accessory protein 4 | 5.411 |
| RFC2 | replication factor C subunit 2 | 2.511 |
| RRM1 | ribonucleotide reductase catalytic subunit M1 | 3.542 |
| RRM2 | ribonucleotide reductase regulatory subunit M2 | 22.979 |
| RUVBL1 | RuvB like AAA ATPase 1 | 2.529 |
| SAC3D1 | SAC3 domain containing 1 | 2.326 |
| SMC4 | structural maintenance of chromosomes 4 | 4.299 |
| SPAG5 | sperm associated antigen 5 | 13.874 |
| SPC24 | SPC24, NDC80 kinetochore complex component | 10.524 |
| SPC25 | SPC25, NDC80 kinetochore complex component | 7.373 |
| SPDL1 | spindle apparatus coiled-coil protein 1 | 3.499 |
| SRSF2 | serine/arginine-rich splicing factor 2 | 2.032 |
| STMN1 | stathmin 1 | 18.981 |
| TACC3 | transforming acidic coiled-coil containing protein 3 | 14.376 |
| TPX2 | TPX2, microtubule-associated | 19.164 |
| TUBA1B | tubulin alpha 1b | 7.135 |
| TUBA1C | tubulin alpha 1c | 5.487 |
| TUBB | tubulin beta class I | 3.710 |
| TUBB4B | tubulin beta 4B class IVb | 5.213 |
| TUBB6 | tubulin beta 6 class V | 2.747 |
| TYMS | thymidylate synthetase | 12.980 |
| UBE2C | ubiquitin conjugating enzyme E2 C | 42.338 |
| UBE2S | ubiquitin conjugating enzyme E2 S | 9.079 |
| UHRF1 | ubiquitin like with PHD and ring finger domains 1 | 7.873 |
| ZWINT | ZW10 interacting kinetochore protein | 17.345 |

**Down regulation**

| Gene symbol | Description | Fold change |
|---|---|---|
| ACVR1 | activin A receptor type 1 | 0.452 |
| MAP3K8 | mitogen-activated protein kinase kinase kinase 8 | 0.413 |

# Supplementary Table 1-2. The major altered genes in cell migration and apoptosis categories.

## Cell migration

| Comparison | Gene symbol | Description | Fold change |
|---|---|---|---|
| IL-1β / CTL | **Up regulation** | | |
| | ABL1 | ABL proto-oncogene 1, non-receptor tyrosine kinase | 2.781 |
| | ACKR3 | atypical chemokine receptor 3 | 2.429 |
| | ANGPT1 | angiopoietin 1 | 3.685 |
| | ATP1B1 | ATPase Na+/K+ transporting subunit beta 1 | 3.611 |
| | B4GALT1 | beta-1,4-galactosyltransferase 1 | 2.524 |
| | CX3CL1 | C-X3-C motif chemokine ligand 1 | 7.417 |
| | CXCL6 | C-X-C motif chemokine ligand 6 | 1760.002 |
| | CYP7B1 | cytochrome P450 family 7 subfamily B member 1 | 7.213 |
| | DDIT4 | DNA damage inducible transcript 4 | 2.920 |
| | FYN | FYN proto-oncogene, Src family tyrosine kinase | 2.816 |
| | ITGA5 | integrin subunit alpha 5 | 2.367 |
| | PDE4B | phosphodiesterase 4B | 10.953 |
| | SLC16A3 | solute carrier family 16 member 3 | 2.925 |
| | SLC7A5 | solute carrier family 7 member 5 | 6.821 |
| | STAT1 | signal transducer and activator of transcription 1 | 2.629 |
| | TNFAIP3 | TNF alpha induced protein 3 | 12.833 |
| | WNT5A | Wnt family member 5A | 5.624 |
| | **Down regulation** | | |
| | CSPG4 | chondroitin sulfate proteoglycan 4 | 0.410 |
| | HSD3B7 | hydroxy-delta-5-steroid dehydrogenase, 3 beta- and steroid delta-isomerase 7 | 0.397 |
| | KANK2 | KN motif and ankyrin repeat domains 2 | 0.384 |
| IL-1β + PRP / IL-1β | **Up regulation** | | |
| | ANLN | anillin actin binding protein | 22.374 |
| | ARPC5L | actin related protein 2/3 complex subunit 5-like | 2.099 |
| | CDK1 | cyclin-dependent kinase 1 | 20.525 |
| | CFL1 | cofilin 1 | 2.021 |
| | CKLF | chemokine-like factor | 2.533 |
| | FAM83D | family with sequence similarity 83 member D | 9.940 |
| | HMGB2 | high mobility group box 2 | 5.778 |
| | KNSTRN | kinetochore-localized astrin/SPAG5 binding protein | 6.595 |
| | PF4V1 | platelet factor 4 variant 1 | 4.747 |
| | TWIST1 | twist family bHLH transcription factor 1 | 2.155 |
| | **Down regulation** | | |
| | ACKR3 | atypical chemokine receptor 3 | 0.380 |
| | ACVR1 | activin A receptor type 1 | 0.452 |
| | CYP7B1 | cytochrome P450 family 7 subfamily B member 1 | 0.305 |

## Apoptosis

| Comparison | Gene symbol | Description | Fold change |
|---|---|---|---|
| IL-1β / CTL | **Up regulation** | | |
| | ABL1 | ABL proto-oncogene 1, non-receptor tyrosine kinase | 2.781 |
| | BCL3 | B-cell CLL/lymphoma 3 | 3.584 |
| | CEBPB | CCAAT/enhancer binding protein beta | 3.111 |
| | CFLAR | CASP8 and FADD like apoptosis regulator | 2.256 |
| | CHEK2 | checkpoint kinase 2 | 2.484 |
| | CYCS | cytochrome c, somatic | 2.366 |
| | DDIT4 | DNA damage inducible transcript 4 | 2.920 |
| | EPB41L3 | erythrocyte membrane protein band 4.1 like 3 | 9.257 |
| | G0S2 | G0/G1 switch 2 | 81.918 |
| | HK2 | hexokinase 2 | 2.424 |
| | IFI6 | interferon alpha inducible protein 6 | 35.987 |
| | INHBA | inhibin beta A | 8.268 |
| | MYDGF | myeloid-derived growth factor | 2.319 |
| | PDCD5 | programmed cell death 5 | 2.441 |
| | SOD2 | superoxide dismutase 2, mitochondrial | 17.511 |
| | SRGN | serglycin | 7.926 |
| | TNFAIP3 | TNF alpha induced protein 3 | 12.833 |
| | TRAF3IP2 | TRAF3 interacting protein 2 | 2.390 |
| | **Down regulation** | | |
| | GSN | gelsolin | 0.284 |
| | KANK2 | KN motif and ankyrin repeat domains 2 | 0.384 |
| | LGALS1 | lectin, galactoside binding soluble 1 | 0.464 |
| | PDCD4 | programmed cell death 4 (neoplastic transformation inhibitor) | 0.463 |
| | PLEKHF1 | pleckstrin homology and FYVE domain containing 1 | 0.404 |
| | PYCARD | PYD and CARD domain containing | 0.456 |
| | RPS6 | ribosomal protein S6 | 0.424 |
| | STK17B | serine/threonine kinase 17b | 0.265 |
| | ZMAT3 | zinc finger matrin-type 3 | 0.267 |
| IL-1β + PRP / IL-1β | **Up regulation** | | |
| | ARL6IP1 | ADP ribosylation factor like GTPase 6 interacting protein 1 | 3.890 |
| | BCL7C | B-cell CLL/lymphoma 7C | 2.072 |
| | BIRC5 | baculoviral IAP repeat containing 5 | 32.789 |
| | BUB1 | BUB1 mitotic checkpoint serine/threonine kinase | 14.994 |
| | BUB1B | BUB1 mitotic checkpoint serine/threonine kinase B | 12.479 |
| | CDK1 | cyclin-dependent kinase 1 | 20.525 |
| | CKAP2 | cytoskeleton associated protein 2 | 7.835 |
| | CRIP1 | cysteine rich protein 1 | 3.451 |
| | E2F1 | E2F transcription factor 1 | 5.955 |
| | EIF5A | eukaryotic translation initiation factor 5A | 2.228 |
| | GGCT | gamma-glutamylcyclotransferase | 2.214 |
| | HMGB2 | high mobility group box 2 | 5.778 |
| | ITGB3BP | integrin subunit beta 3 binding protein | 3.597 |
| | LGALS1 | lectin, galactoside binding soluble 1 | 2.463 |
| | MCM2 | minichromosome maintenance complex component 2 | 5.517 |
| | MELK | maternal embryonic leucine zipper kinase | 6.915 |
| | PARP1 | poly (ADP-ribose) polymerase 1 | 2.018 |
| | TPX2 | TPX2, microtubule-associated | 19.164 |
| | **Down regulation** | | |
| | BNIP3 | BCL2/adenovirus E1B 19kDa interacting protein 3 | 0.357 |
| | BNIP3L | BCL2/adenovirus E1B 19kDa interacting protein 3-like | 0.402 |
| | HK2 | hexokinase 2 | 0.327 |
| | IFI16 | interferon gamma inducible protein 16 | 0.435 |
| | PRUNE2 | prune homolog 2 (Drosophila) | 0.279 |

# Supplementary Table 1-3. The major altered genes in immune response and aging categories.

## Immune response

| | Gene symbol | Description | Fold change |
|---|---|---|---|
| IL-1β / CTL | **Up regulation** | | |
| | ABL1 | ABL proto-oncogene 1, non-receptor tyrosine kinase | 2.781 |
| | ACKR3 | atypical chemokine receptor 3 | 2.429 |
| | APOL1 | apolipoprotein L1 | 4.233 |
| | B2M | beta-2-microglobulin | 2.841 |
| | B4GALT1 | beta-1,4-galactosyltransferase 1 | 2.524 |
| | BCL3 | B-cell CLL/lymphoma 3 | 3.584 |
| | C3 | complement component 3 | 23.334 |
| | CEBPB | CCAAT/enhancer binding protein beta | 3.111 |
| | CFB | complement factor B | 35.001 |
| | CFD | complement factor D (adipsin) | 4.108 |
| | CKAP4 | cytoskeleton-associated protein 4 | 2.165 |
| | CSF1 | colony stimulating factor 1 | 2.684 |
| | CX3CL1 | C-X3-C motif chemokine ligand 1 | 7.417 |
| | CXCL6 | C-X-C motif chemokine ligand 6 | 1760.002 |
| | DNAJC3 | DnaJ heat shock protein family (Hsp40) member C3 | 2.059 |
| | ERAP1 | endoplasmic reticulum aminopeptidase 1 | 2.309 |
| | FTH1 | ferritin, heavy polypeptide 1 | 2.842 |
| | FYN | FYN proto-oncogene, Src family tyrosine kinase | 2.816 |
| | HLA-A | major histocompatibility complex, class I, A | 2.932 |
| | IFI6 | interferon alpha inducible protein 6 | 35.987 |
| | IFITM1 | interferon induced transmembrane protein 1 | 16.433 |
| | IFITM3 | interferon induced transmembrane protein 3 | 2.783 |
| | KYNU | kynureninase | 10.734 |
| | MT2A | metallothionein 2A | 12.229 |
| | NOS2 | nitric oxide synthase 2 | 17.339 |
| | PLAUR | plasminogen activator, urokinase receptor | 2.016 |
| | PLD1 | phospholipase D1 | 8.819 |
| | QSOX1 | quiescin sulfhydryl oxidase 1 | 2.233 |
| | STAT1 | signal transducer and activator of transcription 1 | 2.629 |
| | TRAF3IP2 | TRAF3 interacting protein 2 | 2.390 |
| | WNT5A | Wnt family member 5A | 5.624 |
| | **Down regulation** | | |
| | Gene symbol | Description | Fold change |
| | ANXA2 | annexin A2 | 0.496 |
| | CMKLR1 | chemerin chemokine-like receptor 1 | 0.310 |
| | GSN | gelsolin | 0.284 |
| | LGALS1 | lectin, galactoside binding soluble 1 | 0.464 |
| | PYCARD | PYD and CARD domain containing | 0.456 |
| | RPL13A | ribosomal protein L13a | 0.401 |
| | RPS6 | ribosomal protein S6 | 0.424 |
| | SPON2 | spondin 2 | 0.311 |
| | VAT1 | vesicle amine transport 1 | 0.378 |

| | Gene symbol | Description | Fold change |
|---|---|---|---|
| IL-1β + PRP / IL-1β | **Up regulation** | | |
| | AHCY | adenosylhomocysteinase | 2.029 |
| | ANXA2 | annexin A2 | 2.160 |
| | ATAD3A | ATPase family, AAA domain containing 3A | 2.421 |
| | CRIP1 | cysteine rich protein 1 | 3.451 |
| | HMGB2 | high mobility group box 2 | 5.778 |
| | HMGN2 | high mobility group nucleosomal binding domain 2 | 5.158 |
| | ILF2 | interleukin enhancer binding factor 2 | 2.117 |
| | IMPDH2 | IMP (inosine 5'-monophosphate) dehydrogenase 2 | 2.201 |
| | LGALS1 | lectin, galactoside binding soluble 1 | 2.463 |
| | MT2A | metallothionein 2A | 2.693 |
| | NME2 | NME/NM23 nucleoside diphosphate kinase 2 | 2.048 |
| | PF4V1 | platelet factor 4 variant 1 | 4.747 |
| | PSMC3 | proteasome 26S subunit, ATPase 3 | 2.036 |
| | ROMO1 | reactive oxygen species modulator 1 | 2.037 |
| | TUBB | tubulin beta class I | 3.710 |
| | TUBB4B | tubulin beta 4B class IVb | 5.213 |
| | **Down regulation** | | |
| | Gene symbol | Description | Fold change |
| | ACKR3 | atypical chemokine receptor 3 | 0.380 |
| | APOL1 | apolipoprotein L1 | 0.492 |
| | BCL6 | B-cell CLL/lymphoma 6 | 0.366 |
| | C1S | complement component 1, s subcomponent | 0.362 |
| | C2 | complement component 2 | 0.316 |
| | C3 | complement component 3 | 0.399 |
| | CFB | complement factor B | 0.135 |
| | CFD | complement factor D (adipsin) | 0.210 |
| | IFI16 | interferon gamma inducible protein 16 | 0.435 |
| | IFITM3 | interferon induced transmembrane protein 3 | 0.447 |
| | NOS2 | nitric oxide synthase 2 | 0.062 |
| | PSAP | prosaposin | 0.414 |
| | TRIM22 | tripartite motif containing 22 | 0.280 |

## Aging

| | Gene symbol | Description | Fold change |
|---|---|---|---|
| IL-1β / CTL | **Up regulation** | | |
| | B2M | beta-2-microglobulin | 2.841 |
| | CHEK2 | checkpoint kinase 2 | 2.484 |
| | CX3CL1 | C-X3-C motif chemokine ligand 1 | 7.417 |
| | ID2 | inhibitor of DNA binding 2, HLH protein | 12.514 |
| | KYNU | kynureninase | 10.734 |
| | NAMPT | nicotinamide phosphoribosyltransferase | 22.429 |
| | RPN2 | ribophorin II | 2.084 |
| | SOD2 | superoxide dismutase 2, mitochondrial | 17.511 |
| | **Down regulation** | | |
| | Gene symbol | Description | Fold change |
| | GSN | gelsolin | 0.284 |
| | PDCD4 | programmed cell death 4 (neoplastic transformation inhibitor) | 0.463 |
| | SERPINF1 | serpin family F member 1 | 0.217 |

| | Gene symbol | Description | Fold change |
|---|---|---|---|
| IL-1β + PRP / IL-1β | **Up regulation** | | |
| | AURKB | aurora kinase B | 18.261 |
| | CDK1 | cyclin-dependent kinase 1 | 20.525 |
| | NDUFS6 | NADH:ubiquinone oxidoreductase subunit S6 | 2.022 |
| | ROMO1 | reactive oxygen species modulator 1 | 2.037 |
| | TBX2 | T-box 2 | 3.576 |
| | TYMS | thymidylate synthetase | 12.980 |
| | **Down regulation** | | |
| | **None** | | |
