# Supplementary material for: Transcriptome Analysis of Platelet-Rich Plasma–Treated Osteoarthritic Chondrocyte
Source: Biomed Res Int. 2024 Nov 21;2024:7680736. doi: 10.1155/2024/7680736 (PMC11604281; doi:10.1155/2024/7680736)
Supplement: Supporting Information 3 — Supporting Dataset File: the transcriptome profile by RNA sequencing (RNA-seq). [file 7680736.f3.pdf]

Filter: 24424		Fold change			p-value				Gene information
ID	Gene symbol	IL1b_group /Contorl_group	IL1b_PRP_group /IL1b_group	PRP_group /Contorl_group	IL1b_group /Contorl_group	IL1b_PRP_group /IL1b_group	PRP_group /Contorl_group	Description	NCBI search
13598	MMP3	3416.102	0.317	21.470	0.017	0.084	0.348	matrix metallopeptidase 3	MMP3
4403	CXCL8	2544.882	0.607	3.666	0.267	0.610	0.306	C-X-C motif chemokine ligand 8	CXCL8
4391	CXCL1	2395.465	1.520	25.809	0.224	0.489	0.361	C-X-C motif chemokine ligand 1	CXCL1
4402	CXCL6	1760.002	2.361	71.808	0.014	0.181	0.415	C-X-C motif chemokine ligand 6	CXCL6
8840	IL6	1221.366	0.443	6.085	0.129	0.317	0.428	interleukin 6	IL6
13576	MMP1	637.866	0.222	8.632	0.079	0.121	0.430	matrix metallopeptidase 1	MMP1
2042	C15orf48	383.522	0.820	1.379	0.115	0.677	0.141	chromosome 15 open reading frame 48	C15orf48
4400	CXCL3	246.027	2.330	5.134	0.285	0.196	0.400	C-X-C motif chemokine ligand 3	CXCL3
4399	CXCL2	167.875	0.746	2.948	0.211	0.688	0.393	C-X-C motif chemokine ligand 2	CXCL2
21996	TNFAIP6	142.158	0.215	3.892	0.221	0.304	0.335	TNF alpha induced protein 6	TNFAIP6
8461	HSD11B1	134.453	0.198	1.360	0.031	0.046	0.589	hydroxysteroid (11-beta) dehydrogenase 1	HSD11B1
19848	SNHG25	126.767	0.519	115.576	0.423	0.709	0.423	small nucleolar RNA host gene 25	SNHG25
4401	CXCL5	105.394	5.025	1.918	0.348	0.091	0.346	C-X-C motif chemokine ligand 5	CXCL5
2944	CCL20	103.737	0.450	1.077	0.307	0.558	0.228	C-C motif chemokine ligand 20	CCL20
21427	TFPI2	101.175	2.417	11.850	0.389	0.262	0.201	tissue factor pathway inhibitor 2	TFPI2
1873	BST2	96.191	0.431	0.934	0.084	0.285	0.893	bone marrow stromal cell antigen 2	BST2
8801	IL1RN	94.532	0.035	1.015	0.416	0.426	0.521	interleukin 1 receptor antagonist	IL1RN
2961	CCL8	93.522	0.094	1.868	0.286	0.318	0.166	C-C motif chemokine ligand 8	CCL8
8830	IL36B	83.050	0.075	1.876	0.401	0.426	0.109	interleukin 36, beta	IL36B
6949	G0S2	81.918	0.425	2.671	0.028	0.078	0.392	G0/G1 switch 2	G0S2
18553	SAA1	75.271	0.352	1.373	0.332	0.507	0.345	serum amyloid A1	SAA1
17282	PTGS2	74.944	0.293	0.816	0.306	0.434	0.596	prostaglandin-endoperoxide synthase 2	PTGS2
19587	SLC7A2	70.421	0.512	2.098	0.180	0.422	0.312	solute carrier family 7 member 2	SLC7A2
3466	CH25H	69.429	0.864	4.171	0.182	0.809	0.048	cholesterol 25-hydroxylase	CH25H
2959	CCL5	69.362	0.321	1.203	0.224	0.355	0.721	C-C motif chemokine ligand 5	CCL5
8632	IFI27	65.409	0.523	0.884	0.051	0.293	0.841	interferon alpha inducible protein 27	IFI27
2960	CCL7	55.147	0.603	3.342	0.209	0.536	0.039	C-C motif chemokine ligand 7	CCL7
8762	IL11	54.109	1.988	2.169	0.340	0.519	0.427	interleukin 11	IL11
4195	CSF3	51.702	0.848	0.964	0.319	0.880	0.542	colony stimulating factor 3	CSF3
9917	LBP	47.460	0.045	1.299	0.322	0.331	0.722	lipopolysaccharide binding protein	LBP
1990	C11orf96	42.843	0.167	7.326	0.153	0.200	0.221	chromosome 11 open reading frame 96	C11orf96
17269	PTGES	42.277	1.758	9.200	0.163	0.255	0.393	prostaglandin E synthase	PTGES
8625	IER3	38.803	0.666	3.266	0.117	0.458	0.291	immediate early response 3	IER3
16267	PILRA	37.291	0.165	0.805	0.200	0.248	0.120	paired immunoglobin like type 2 receptor alpha	PILRA
8639	IFI6	35.987	0.492	0.645	0.006	0.094	0.047	interferon alpha inducible protein 6	IFI6
3432	CFB	35.001	0.135	0.943	0.001	0.004	0.835	complement factor B	CFB
4080	CPXM1	31.990	0.111	1.081	0.334	0.367	0.842	carboxypeptidase X (M14 family), member 1	CPXM1
8638	IFI44L	31.655	0.143	0.935	0.167	0.202	0.851	interferon induced protein 44 like	IFI44L
13580	MMP13	31.299	0.221	1.883	0.160	0.235	0.438	matrix metallopeptidase 13	MMP13
20769	STC1	30.798	3.925	22.505	0.001	0.016	0.378	stanniocalcin 1	STC1
4150	CRTAC1	30.708	0.204	4.288	0.285	0.358	0.514	cartilage acidic protein 1	CRTAC1
19505	SLC39A8	30.168	0.202	0.769	0.170	0.238	0.530	solute carrier family 39 member 8	SLC39A8
9872	LAMB3	29.361	0.233	1.989	0.134	0.191	0.399	laminin subunit beta 3	LAMB3
2943	CCL2	28.517	0.740	2.199	0.159	0.652	0.063	C-C motif chemokine ligand 2	CCL2
8792	IL1B	28.340	0.195	1.036	0.380	0.453	0.454	interleukin 1, beta	IL1B
9955	LCN2	27.434	0.460	0.963	0.146	0.355	0.458	lipocalin 2	LCN2
10062	LIF	26.421	0.715	1.363	0.288	0.726	0.133	leukemia inhibitory factor	LIF
18440	RSAD2	24.730	0.079	0.807	0.344	0.360	0.236	radical S-adenosyl methionine domain containing 2	RSAD2
8832	IL36RN	24.248	0.053	1.009	0.411	0.416	0.685	interleukin 36 receptor antagonist	IL36RN
413	ADORA2A	24.025	0.120	1.361	0.406	0.440	0.303	adenosine A2a receptor	ADORA2A
2302	C3	23.334	0.399	0.414	0.008	0.045	0.490	complement component 3	C3
19962	SNORA84	23.119	0.043	1.000	0.423	0.423	0.943	small nucleolar RNA, H/ACA box 84	SNORA84
14179	NAMPT	22.429	0.493	1.533	0.025	0.083	0.281	nicotinamide phosphoribosyltransferase	NAMPT
23619	ZC3H12A	22.308	0.443	1.160	0.189	0.374	0.584	zinc finger CCCH-type containing 12A	ZC3H12A
4351	CTSS	22.040	0.466	1.147	0.208	0.414	0.459	cathepsin S	CTSS
5101	DNER	20.846	0.791	2.097	0.233	0.756	0.359	delta/notch like EGF repeat containing	DNER
20205	SNORD89	20.276	0.049	1.000	0.423	0.423	0.943	small nucleolar RNA, C/D box 89	SNORD89
3931	COL7A1	20.262	0.723	2.137	0.017	0.279	0.366	collagen type VII alpha 1	COL7A1
9017	ISG15	19.912	0.290	1.097	0.122	0.201	0.547	ISG15 ubiquitin-like modifier	ISG15
13997	MX1	19.253	0.121	0.514	0.229	0.255	0.415	MX dynamin like GTPase 1	MX1
19952	SNORA77	18.921	0.053	1.000	0.423	0.423	0.943	small nucleolar RNA, H/ACA box 77	SNORA77
8831	IL36G	18.625	0.063	1.000	0.408	0.412	0.943	interleukin 36, gamma	IL36G
14314	NDP	18.324	0.426	1.151	0.021	0.130	0.697	Norrie disease (pseudoglioma)	NDP
2268	C2CD4B	18.037	0.169	0.952	0.364	0.415	0.845	C2 calcium-dependent domain containing 4B	C2CD4B
8237	HLA-F	18.014	0.587	1.170	0.191	0.500	0.556	major histocompatibility complex, class I, F	HLA-F
8813	IL23A	17.884	0.114	0.959	0.408	0.433	0.908	interleukin 23, alpha subunit p19	IL23A
5571	ELF3	17.820	0.264	0.987	0.176	0.253	0.355	E74 like ETS transcription factor 3	ELF3
20295	SOD2	17.511	0.432	1.850	0.033	0.082	0.221	superoxide dismutase 2, mitochondrial	SOD2
7981	HCK	17.347	0.089	1.000	0.389	0.402	0.943	HCK proto-oncogene, Src family tyrosine kinase	HCK
14655	NOS2	17.339	0.062	1.000	0.012	0.012	0.943	nitric oxide synthase 2	NOS2
16216	PID1	17.294	0.958	1.468	0.063	0.889	0.640	phosphotyrosine interaction domain containing 1	PID1
19861	SNORA11B	16.988	0.059	7.248	0.423	0.423	0.422	small nucleolar RNA, H/ACA box 11B	SNORA11B
19498	SLC39A14	16.530	0.532	2.226	0.060	0.191	0.137	solute carrier family 39 member 14	SLC39A14
8646	IFITM1	16.433	0.308	1.685	0.039	0.086	0.591	interferon induced transmembrane protein 1	IFITM1
16201	PI3	16.050	0.665	1.846	0.235	0.671	0.286	peptidase inhibitor 3	PI3
14987	OAS2	15.487	0.153	1.187	0.253	0.287	0.801	2'-5'-oligoadenylate synthetase 2	OAS2
7199	GFPT2	15.466	0.877	2.255	0.206	0.831	0.232	glutamine-fructose-6-phosphate transaminase 2	GFPT2
12822	LUCAT1	15.169	0.194	1.210	0.217	0.265	0.365	lung cancer associated transcript 1 (non-protein coding)	LUCAT1
3785	CMPK2	15.142	0.138	0.859	0.297	0.326	0.584	cytidine/uridine monophosphate kinase 2	CMPK2
14986	OAS1	14.474	0.171	0.913	0.278	0.320	0.901	2'-5'-oligoadenylate synthetase 1	OAS1
3542	CHRDL2	13.941	0.090	0.987	0.426	0.434	0.971	chordin-like 2	CHRDL2
8836	IL4I1	13.830	0.305	0.900	0.327	0.451	0.844	interleukin 4 induced 1	IL4I1
2154	C1QTNF1	13.824	0.738	1.459	0.106	0.615	0.545	C1q and tumor necrosis factor related protein 1	C1QTNF1
14989	OASL	13.791	0.293	1.077	0.245	0.349	0.366	2'-5'-oligoadenylate synthetase like	OASL

1641	BCL2A1	13.601	0.400	1.569	0.292	0.466	0.298	BCL2 related protein A1	BCL2A1
18955	SERPINE2	13.384	0.971	5.168	0.088	0.932	0.413	serpin family E member 2	SERPINE2
13874	MT1G	13.207	0.816	1.501	0.107	0.817	0.416	metallothionein 1G	MT1G
2953	CCL3	12.914	0.126	1.000	0.313	0.334	0.943	C-C motif chemokine ligand 3	CCL3
21995	TNFAIP3	12.833	0.657	1.343	0.000	0.027	0.245	TNF alpha induced protein 3	TNFAIP3
14626	NOD2	12.537	0.169	0.982	0.104	0.124	0.871	nucleotide binding oligomerization domain containing 2	NOD2
8605	ID2	12.514	0.585	2.047	0.002	0.008	0.371	inhibitor of DNA binding 2, HLH protein	ID2
18677	SCARNA22	12.342	0.935	1.000	0.422	0.964	0.943	small Cajal body-specific RNA 22	SCARNA22
13882	MT2A	12.229	2.693	6.170	0.044	0.018	0.201	metallothionein 2A	MT2A
19881	SNORA26	11.766	0.085	1.000	0.423	0.423	0.943	small nucleolar RNA, H/ACA box 26	SNORA26
21707	TMEM132A	11.666	0.691	1.442	0.064	0.391	0.391	transmembrane protein 132A	TMEM132A
14471	NFKBIZ	11.508	0.487	1.188	0.020	0.063	0.466	NFKB inhibitor zeta	NFKBIZ
7136	GCH1	11.281	0.315	1.156	0.216	0.314	0.333	GTP cyclohydrolase 1	GCH1
8060	HERC6	11.200	0.193	0.768	0.165	0.198	0.168	HECT and RLD domain containing E3 ubiquitin protein ligase family m	HERC6
13873	MT1F	11.192	0.533	1.361	0.030	0.309	0.464	metallothionein 1F	MT1F
13012	MAOB	11.061	0.228	0.836	0.153	0.200	0.786	monoamine oxidase B	MAOB
18554	SAA2	11.013	0.384	0.963	0.252	0.406	0.444	serum amyloid A2	SAA2
15952	PDE4B	10.953	0.647	2.425	0.029	0.156	0.515	phosphodiesterase 4B	PDE4B
9839	KYNU	10.734	0.549	4.333	0.003	0.258	0.467	kynureninase	KYNU
8637	IFI44	10.347	0.223	0.994	0.121	0.155	0.989	interferon induced protein 44	IFI44
5825	ESM1	9.844	0.599	1.413	0.338	0.674	0.098	endothelial cell specific molecule 1	ESM1
21602	TLR2	9.828	0.129	0.988	0.320	0.331	0.813	toll like receptor 2	TLR2
13833	MSC	9.810	0.304	0.986	0.293	0.399	0.972	musculin	MSC
14988	OAS3	9.734	0.339	1.264	0.253	0.362	0.646	2'-5'-oligoadenylate synthetase 3	OAS3
4192	CSF2	9.621	0.406	0.917	0.364	0.527	0.416	colony stimulating factor 2	CSF2
19888	SNORA31	9.596	0.104	1.000	0.422	0.422	0.943	small nucleolar RNA, H/ACA box 31	SNORA31
19924	SNORA61	9.571	1.763	1.000	0.422	0.725	0.943	small nucleolar RNA, H/ACA box 61	SNORA61
2507	CA9	9.495	0.093	1.213	0.397	0.391	0.657	carbonic anhydrase 9	CA9
20117	SNORD22	9.471	1.928	15.005	0.423	0.425	0.423	small nucleolar RNA, C/D box 22	SNORD22
16676	POU2F2	9.464	0.894	0.981	0.101	0.767	0.968	POU class 2 homeobox 2	POU2F2
204	ACSL4	9.363	1.173	2.266	0.172	0.831	0.423	acyl-CoA synthetase long-chain family member 4	ACSL4
5699	EPB41L3	9.257	1.385	2.454	0.036	0.160	0.347	erythrocyte membrane protein band 4.1 like 3	EPB41L3
1021	AQP9	9.155	0.309	1.437	0.177	0.262	0.406	aquaporin 9	AQP9
16409	PLD1	8.819	0.572	1.720	0.002	0.007	0.369	phospholipase D1	PLD1
14466	NFKBIA	8.760	0.668	1.749	0.170	0.517	0.066	NFKB inhibitor alpha	NFKBIA
19897	SNORA38	8.754	0.114	1.000	0.422	0.422	0.943	small nucleolar RNA, H/ACA box 38	SNORA38
19945	SNORA72	8.754	0.114	1.000	0.422	0.422	0.943	small nucleolar RNA, H/ACA box 72	SNORA72
8588	IBSP	8.693	0.903	1.036	0.200	0.860	0.396	integrin binding sialoprotein	IBSP
13379	MFSD2A	8.638	0.786	1.593	0.214	0.712	0.108	major facilitator superfamily domain containing 2A	MFSD2A
6467	FCAMR	8.591	0.128	1.000	0.215	0.219	0.943	Fc fragment of IgA and IgM receptor	FCAMR
19009	SFRP1	8.563	0.829	4.517	0.434	0.879	0.502	secreted frizzled-related protein 1	SFRP1
18457	RSPO3	8.503	0.834	1.604	0.191	0.766	0.328	R-spondin 3	RSPO3
19922	SNORA6	8.463	0.118	1.000	0.422	0.422	0.943	small nucleolar RNA, H/ACA box 6	SNORA6
8884	INHBA	8.268	1.743	2.364	0.020	0.068	0.327	inhibin beta A	INHBA
8602	ICOSLG	8.264	0.282	0.911	0.320	0.403	0.123	inducible T-cell co-stimulator ligand	ICOSLG
2267	C2CD4A	8.229	0.173	1.018	0.378	0.401	0.882	C2 calcium-dependent domain containing 4A	C2CD4A
16270	PIM2	8.048	0.303	0.871	0.308	0.394	0.712	Pim-2 proto-oncogene, serine/threonine kinase	PIM2
8643	IFIT2	8.047	0.128	0.713	0.412	0.415	0.511	interferon induced protein with tetratricopeptide repeats 2	IFIT2
19870	SNORA16A	8.025	0.125	1.000	0.422	0.422	0.943	small nucleolar RNA, H/ACA box 16A	SNORA16A
8985	IRAK2	8.004	0.350	1.220	0.293	0.405	0.695	interleukin 1 receptor associated kinase 2	IRAK2
20594	SRGN	7.926	0.828	1.462	0.008	0.459	0.527	serglycin	SRGN
22822	UCN2	7.904	0.788	1.199	0.099	0.550	0.097	urocortin 2	UCN2
16484	PLTP	7.901	0.133	0.675	0.127	0.129	0.037	phospholipid transfer protein	PLTP
6546	FGF10	7.884	0.447	2.872	0.442	0.632	0.570	fibroblast growth factor 10	FGF10
7930	HAS3	7.868	0.350	1.456	0.266	0.401	0.583	hyaluronan synthase 3	HAS3
23433	XAF1	7.808	0.178	1.035	0.090	0.101	0.918	XIAP associated factor 1	XAF1
1726	BIRC3	7.772	0.522	1.183	0.155	0.349	0.774	baculoviral IAP repeat containing 3	BIRC3
8273	HMGN2P46	7.733	0.702	0.973	0.210	0.605	0.311	high mobility group nucleosomal binding domain 2 pseudogene 46	HMGN2P46
19937	SNORA70E	7.696	0.130	1.000	0.422	0.422	0.943	small nucleolar RNA, H/ACA box 70E	SNORA70E
4469	CYP24A1	7.635	1.341	2.847	0.084	0.772	0.416	cytochrome P450 family 24 subfamily A member 1	CYP24A1
709	AMTN	7.558	0.382	1.357	0.415	0.544	0.300	amelotin	AMTN
21453	TGM2	7.475	2.122	4.522	0.193	0.332	0.346	transglutaminase 2	TGM2
16023	PDZK1IP1	7.460	0.395	0.655	0.004	0.058	0.493	PDZK1 interacting protein 1	PDZK1IP1
4386	CX3CL1	7.417	0.355	0.824	0.038	0.063	0.715	C-X3-C motif chemokine ligand 1	CX3CL1
18926	SERPINA1	7.411	1.156	6.615	0.073	0.759	0.368	serpin family A member 1	SERPINA1
16380	PLAT	7.308	0.445	1.406	0.317	0.485	0.398	plasminogen activator, tissue type	PLAT
20773	STEAP2	7.297	0.436	1.297	0.011	0.083	0.223	STEAP2 metalloreductase	STEAP2
23418	WTAPP1	7.282	0.337	1.047	0.020	0.054	0.146	Wilms tumor 1 associated protein pseudogene 1	WTAPP1
8641	IFIT1	7.260	0.122	0.450	0.257	0.252	0.056	interferon induced protein with tetratricopeptide repeats 1	IFIT1
23417	WTAP	7.232	0.425	1.306	0.164	0.292	0.190	Wilms tumor 1 associated protein	WTAP
4523	CYP7B1	7.213	0.305	0.841	0.018	0.032	0.398	cytochrome P450 family 7 subfamily B member 1	CYP7B1
706	AMPD3	7.148	0.446	1.235	0.157	0.292	0.412	adenosine monophosphate deaminase 3	AMPD3
19898	SNORA38B	7.141	0.140	1.000	0.423	0.423	0.943	small nucleolar RNA, H/ACA box 38B	SNORA38B
13577	MMP10	7.099	4.225	9.648	0.000	0.372	0.408	matrix metallopeptidase 10	MMP10
6497	FCRLA	7.033	1.538	2.393	0.385	0.585	0.001	Fc receptor like A	FCRLA
13883	MT3	6.949	0.175	0.933	0.026	0.029	0.336	metallothionein 3	MT3
3075	CD38	6.865	0.504	1.119	0.093	0.288	0.134	CD38 molecule	CD38
937	APCDD1	6.855	0.674	5.736	0.212	0.570	0.345	adenomatosis polyposis coli down-regulated 1	APCDD1
22239	TREM1	6.848	0.367	1.058	0.298	0.418	0.413	triggering receptor expressed on myeloid cells 1	TREM1
19590	SLC7A5	6.821	0.776	2.177	0.026	0.272	0.031	solute carrier family 7 member 5	SLC7A5
574	AKR1B1	6.774	0.726	1.230	0.380	0.775	0.462	aldo-keto reductase family 1, member B1 (aldose reductase)	AKR1B1
1672	BDKRB1	6.771	1.039	2.002	0.069	0.950	0.201	bradykinin receptor B1	BDKRB1
8640	IFIH1	6.745	0.159	0.739	0.296	0.301	0.331	interferon induced with helicase C domain 1	IFIH1
9071	ITGB8	6.738	0.150	0.493	0.245	0.244	0.354	integrin subunit beta 8	ITGB8
2494	CA12	6.677	0.353	0.938	0.008	0.033	0.866	carbonic anhydrase 12	CA12
13579	MMP12	6.670	1.392	1.449	0.043	0.725	0.421	matrix metallopeptidase 12	MMP12
21994	TNFAIP2	6.646	0.212	0.764	0.215	0.240	0.671	TNF alpha induced protein 2	TNFAIP2
19919	SNORA5A	6.574	0.152	1.000	0.423	0.423	0.943	small nucleolar RNA, H/ACA box 5A	SNORA5A
4197	CSGALNACT1	6.486	0.778	2.341	0.021	0.257	0.345	chondroitin sulfate N-acetylgalactosaminyltransferase 1	CSGALNACT1
4498	CYP3A5	6.476	0.137	0.865	0.384	0.376	0.658	cytochrome P450 family 3 subfamily A member 5	CYP3A5
8644	IFIT3	6.473	0.166	0.778	0.307	0.313	0.181	interferon induced protein with tetratricopeptide repeats 3	IFIT3

14808	NRP2	6.412	1.091	1.913	0.090	0.810	0.419	neuropilin 2	NRP2
3720	CLIC6	6.337	1.322	3.119	0.014	0.457	0.368	chloride intracellular channel 6	CLIC6
20771	STEAP1	6.324	0.830	1.782	0.117	0.698	0.294	six transmembrane epithelial antigen of the prostate 1	STEAP1
22679	TYMP	6.274	0.409	2.663	0.070	0.116	0.454	thymidine phosphorylase	TYMP
13298	MEIS1	6.228	0.998	1.078	0.003	0.995	0.791	Meis homeobox 1	MEIS1
22992	USP18	6.166	0.301	1.213	0.150	0.198	0.295	ubiquitin specific peptidase 18	USP18
16471	PLOD2	6.159	0.692	1.765	0.217	0.592	0.220	procollagen-lysine,2-oxoglutarate 5-dioxygenase 2	PLOD2
13998	MX2	6.157	0.246	1.133	0.288	0.326	0.267	MX dynamin like GTPase 2	MX2
8773	IL15RA	6.138	0.307	1.561	0.301	0.375	0.366	interleukin 15 receptor, alpha	IL15RA
2956	CCL4	6.080	0.164	1.000	0.309	0.309	0.943	C-C motif chemokine ligand 4	CCL4
4023	CP	6.003	0.125	0.550	0.372	0.350	0.636	ceruloplasmin (ferroxidase)	CP
7273	GJB2	5.955	0.254	1.023	0.417	0.460	0.440	gap junction protein beta 2	GJB2
1759	BMP2	5.931	0.915	1.793	0.113	0.808	0.556	bone morphogenetic protein 2	BMP2
22016	TNFRSF1B	5.924	0.594	1.832	0.054	0.178	0.411	tumor necrosis factor receptor superfamily member 1B	TNFRSF1B
3569	CHST11	5.822	0.501	1.122	0.004	0.034	0.510	carbohydrate (chondroitin 4) sulfotransferase 11	CHST11
5751	EPSTI1	5.812	0.407	1.872	0.021	0.040	0.452	epithelial stromal interaction 1 (breast)	EPSTI1
20760	STAT4	5.795	1.480	1.910	0.006	0.178	0.417	signal transducer and activator of transcription 4	STAT4
15954	PDE4D	5.783	1.040	1.900	0.176	0.932	0.445	phosphodiesterase 4D	PDE4D
14484	NGF	5.777	0.320	1.090	0.248	0.315	0.782	nerve growth factor	NGF
119	ABI3BP	5.777	0.275	1.266	0.102	0.127	0.749	ABI family member 3 binding protein	ABI3BP
8986	IRAK3	5.769	0.636	0.955	0.114	0.356	0.851	interleukin 1 receptor associated kinase 3	IRAK3
8592	ICAM1	5.753	0.354	1.097	0.271	0.379	0.748	intercellular adhesion molecule 1	ICAM1
8702	IGF1	5.751	0.845	2.607	0.008	0.192	0.435	insulin like growth factor 1	IGF1
18618	SAT1	5.749	0.801	1.952	0.017	0.341	0.459	spermidine/spermine N1-acetyltransferase 1	SAT1
735	ANGPTL1	5.690	0.351	1.056	0.100	0.180	0.475	angiopoietin like 1	ANGPTL1
19942	SNORA71C	5.654	1.126	1.000	0.423	0.929	0.943	small nucleolar RNA, H/ACA box 71C	SNORA71C
23396	WNT5A	5.624	1.032	2.330	0.006	0.917	0.381	Wnt family member 5A	WNT5A
8217	HLA-B	5.610	0.622	0.834	0.101	0.329	0.285	major histocompatibility complex, class I, B	HLA-B
8826	IL32	5.609	0.470	1.054	0.270	0.454	0.958	interleukin 32	IL32
6608	FILIP1	5.604	0.346	1.058	0.256	0.337	0.697	filamin A interacting protein 1	FILIP1
19583	SLC7A11	5.594	0.577	0.848	0.364	0.610	0.810	solute carrier family 7 member 11	SLC7A11
23354	WFDC21P	5.575	0.616	0.841	0.286	0.635	0.510	WAP four-disulfide core domain 21, pseudogene	WFDC21P
8635	IFI30	5.553	0.644	1.510	0.072	0.312	0.596	IFI30, lysosomal thiol reductase	IFI30
8998	IRF7	5.536	0.199	0.965	0.129	0.134	0.795	interferon regulatory factor 7	IRF7
18409	RRAD	5.516	0.854	0.751	0.112	0.702	0.679	Ras-related associated with diabetes	RRAD
1712	BHLHE41	5.485	0.278	0.741	0.056	0.074	0.104	basic helix-loop-helix family member e41	BHLHE41
22023	TNFSF10	5.480	0.368	2.386	0.240	0.329	0.332	tumor necrosis factor superfamily member 10	TNFSF10
500	AGRN	5.454	0.385	0.743	0.024	0.035	0.518	agrin	AGRN
19765	SMOC1	5.446	1.259	3.991	0.178	0.694	0.509	SPARC related modular calcium binding 1	SMOC1
8844	IL7R	5.402	2.077	2.303	0.018	0.222	0.102	interleukin 7 receptor	IL7R
1674	BDNF	5.305	0.902	1.403	0.052	0.713	0.353	brain-derived neurotrophic factor	BDNF
4339	CTSC	5.270	0.444	2.283	0.436	0.591	0.577	cathepsin C	CTSC
3713	CLGN	5.265	0.510	1.008	0.304	0.531	0.981	calmegin	CLGN
16479	PLSCR1	5.246	0.318	1.575	0.114	0.151	0.369	phospholipid scramblase 1	PLSCR1
8196	HIST2H2BE	5.238	0.385	1.107	0.348	0.456	0.761	histone cluster 2, H2be	HIST2H2BE
22007	TNFRSF11B	5.222	1.045	1.610	0.154	0.915	0.523	tumor necrosis factor receptor superfamily member 11b	TNFRSF11B
14588	NMB	5.157	0.183	1.555	0.435	0.431	0.457	neuromedin B	NMB
18660	SCARB1	5.153	0.282	0.694	0.395	0.443	0.719	scavenger receptor class B member 1	SCARB1
6561	FGF2	5.133	1.193	0.996	0.112	0.677	0.916	fibroblast growth factor 2	FGF2
21737	TMEM158	5.123	1.598	1.597	0.016	0.149	0.133	transmembrane protein 158 (gene/pseudogene)	TMEM158
4392	CXCL10	5.095	0.256	1.000	0.407	0.438	0.943	C-X-C motif chemokine ligand 10	CXCL10
9041	ITGA1	5.088	0.616	0.953	0.255	0.529	0.543	integrin subunit alpha 1	ITGA1
19164	SIK1	5.044	0.747	1.440	0.013	0.249	0.414	salt inducible kinase 1	SIK1
15016	ODF3B	5.007	0.431	3.133	0.114	0.183	0.404	outer dense fiber of sperm tails 3B	ODF3B
3502	CHI3L2	4.996	0.265	1.835	0.325	0.354	0.550	chitinase 3 like 2	CHI3L2
8156	HIST1H2BD	4.967	0.533	1.681	0.343	0.559	0.349	histone cluster 1, H2bd	HIST1H2BD
16491	PLXNA4	4.937	0.983	2.643	0.245	0.976	0.513	plexin A4	PLXNA4
15515	OSGIN2	4.918	0.556	0.853	0.287	0.507	0.613	oxidative stress induced growth inhibitor family member 2	OSGIN2
19777	SMS	4.917	0.729	1.984	0.248	0.640	0.004	spermine synthase	SMS
21991	TNC	4.913	0.776	7.592	0.143	0.664	0.349	tenascin C	TNC
7118	GBP1P1	4.912	0.287	1.173	0.187	0.220	0.057	guanylate binding protein 1 pseudogene 1	GBP1P1
11257	LOC10050562	4.900	0.697	1.110	0.165	0.511	0.686	uncharacterized LOC100505622	LOC100505622
19964	SNORD10	4.900	0.149	2.161	0.042	0.018	0.454	small nucleolar RNA, C/D box 10	SNORD10
7285	GK	4.888	0.698	1.615	0.283	0.635	0.448	glycerol kinase	GK
695	AMIGO2	4.886	0.817	0.808	0.355	0.812	0.529	adhesion molecule with Ig-like domain 2	AMIGO2
72	ABCC3	4.866	0.591	1.676	0.013	0.072	0.328	ATP binding cassette subfamily C member 3	ABCC3
8142	HIST1H2AC	4.834	0.432	1.463	0.340	0.475	0.276	histone cluster 1, H2ac	HIST1H2AC
13872	MT1E	4.832	1.547	3.013	0.112	0.242	0.015	metallothionein 1E	MT1E
5724	EPHB2	4.761	0.606	1.019	0.329	0.591	0.943	EPH receptor B2	EPHB2
19254	SLC15A3	4.741	0.323	1.077	0.143	0.179	0.865	solute carrier family 15 member 3	SLC15A3
8913	INSIG2	4.703	0.380	0.804	0.021	0.031	0.427	insulin induced gene 2	INSIG2
18948	SERPINB7	4.692	0.248	1.012	0.420	0.437	0.446	serpin family B member 7	SERPINB7
17155	PSD4	4.689	0.637	1.271	0.431	0.704	0.549	pleckstrin and Sec7 domain containing 4	PSD4
2303	C3AR1	4.685	0.289	1.140	0.324	0.363	0.249	complement component 3a receptor 1	C3AR1
8059	HERC5	4.679	0.417	0.913	0.281	0.398	0.397	HECT and RLD domain containing E3 ubiquitin protein ligase 5	HERC5
6487	FCMR	4.665	0.314	1.037	0.187	0.227	0.624	Fc fragment of IgM receptor	FCMR
19515	SLC43A3	4.638	0.517	0.993	0.111	0.285	0.979	solute carrier family 43 member 3	SLC43A3
1764	BMP6	4.623	1.148	3.445	0.034	0.419	0.473	bone morphogenetic protein 6	BMP6
577	AKR1C1	4.623	0.616	1.107	0.447	0.709	0.861	aldo-keto reductase family 1, member C1	AKR1C1
3099	CD70	4.596	0.826	1.353	0.421	0.865	0.709	CD70 molecule	CD70
19595	SLC7A7	4.563	0.337	1.141	0.134	0.181	0.504	solute carrier family 7 member 7	SLC7A7
16112	PGF	4.544	2.544	12.639	0.384	0.517	0.427	placental growth factor	PGF
14907	NUDT6	4.520	0.979	1.166	0.168	0.965	0.463	nudix (nucleoside diphosphate linked moiety X)-type motif 6	NUDT6
23150	VNN1	4.510	0.522	1.014	0.260	0.455	0.673	vanin 1	VNN1
17771	RCAN1	4.477	2.585	2.271	0.185	0.129	0.326	regulator of calcineurin 1	RCAN1
4729	DDX58	4.456	0.254	0.793	0.187	0.198	0.146	DEXD/H-box helicase 58	DDX58
16464	PLK2	4.430	0.750	2.096	0.289	0.720	0.198	polo like kinase 2	PLK2
19514	SLC43A2	4.394	0.486	1.387	0.161	0.284	0.138	solute carrier family 43 member 2	SLC43A2
12993	MAN1A1	4.385	0.535	0.965	0.014	0.036	0.857	mannosidase alpha class 1A member 1	MAN1A1
8541	HTR2A	4.384	0.349	1.138	0.384	0.453	0.668	5-hydroxytryptamine (serotonin) receptor 2A, G protein-coupled	HTR2A
12837	LY6E	4.381	0.509	1.145	0.009	0.003	0.784	lymphocyte antigen 6 complex, locus E	LY6E

8155	HIST1H2BC	4.354	0.309	1.018	0.424	0.466	0.930	histone cluster 1, H2bc	HIST1H2BC
13875	MT1H	4.353	0.460	1.000	0.309	0.472	0.943	metallothionein 1H	MT1H
1393	ATP2B1	4.332	0.466	0.962	0.315	0.455	0.827	ATPase plasma membrane Ca2+ transporting 1	ATP2B1
8119	HILPDA	4.328	0.245	1.106	0.376	0.382	0.726	hypoxia inducible lipid droplet associated	HILPDA
22022	TNFRSF9	4.310	0.228	0.958	0.294	0.292	0.521	tumor necrosis factor receptor superfamily member 9	TNFRSF9
8040	HELZ2	4.272	0.251	0.941	0.174	0.180	0.794	helicase with zinc finger 2	HELZ2
23110	VEGFA	4.261	0.247	0.827	0.214	0.217	0.686	vascular endothelial growth factor A	VEGFA
7928	HAS2	4.254	5.115	3.921	0.004	0.130	0.161	hyaluronan synthase 2	HAS2
313	ADAMTS6	4.251	0.856	2.912	0.047	0.547	0.386	ADAM metallopeptidase with thrombospondin type 1 motif 6	ADAMTS6
22038	TNIP3	4.250	0.377	1.061	0.291	0.366	0.082	TNFAIP3 interacting protein 3	TNIP3
15907	PCSK1	4.235	1.429	7.637	0.012	0.584	0.404	proprotein convertase subtilisin/kexin type 1	PCSK1
987	APOL1	4.233	0.492	1.466	0.011	0.011	0.289	apolipoprotein L1	APOL1
3107	CD82	4.219	1.323	2.795	0.066	0.302	0.359	CD82 molecule	CD82
9287	KCNMB1	4.212	0.267	0.925	0.092	0.099	0.494	potassium calcium-activated channel subfamily M regulatory beta su	KCNMB1
13834	MSC-AS1	4.203	0.300	0.762	0.122	0.143	0.287	MSC antisense RNA 1	MSC-AS1
14465	NFKB2	4.201	0.646	1.263	0.150	0.438	0.353	nuclear factor of kappa light polypeptide gene enhancer in B-cells 2	NFKB2
14770	NR4A2	4.195	0.953	1.443	0.043	0.800	0.385	nuclear receptor subfamily 4 group A member 2	NR4A2
6025	FAM132B	4.169	1.359	1.570	0.013	0.405	0.319	family with sequence similarity 132 member B	FAM132B
16991	PRL	4.158	3.083	1.434	0.297	0.452	0.420	prolactin	PRL
7950	HBEGF	4.147	0.936	1.161	0.183	0.896	0.523	heparin binding EGF like growth factor	HBEGF
4908	DIO2	4.139	0.335	4.632	0.432	0.490	0.464	deiodinase, iodothyronine, type II	DIO2
19234	SLC11A2	4.123	0.449	0.705	0.142	0.229	0.054	solute carrier family 11 member 2	SLC11A2
3435	CFD	4.108	0.210	0.558	0.027	0.009	0.423	complement factor D (adipsin)	CFD
10021	LGALS3BP	4.023	0.405	0.860	0.031	0.024	0.821	lectin, galactoside binding soluble 3 binding protein	LGALS3BP
16360	PLA2G4A	3.991	0.665	0.676	0.310	0.595	0.660	phospholipase A2 group IVA	PLA2G4A
21195	TBC1D9	3.988	0.453	0.887	0.242	0.351	0.719	TBC1 domain family member 9	TBC1D9
19288	SLC19A3	3.971	0.577	1.376	0.030	0.129	0.459	solute carrier family 19 member 3	SLC19A3
13096	MARCH3	3.946	0.400	1.194	0.016	0.026	0.248	membrane associated ring-CH-type finger 3	MARCH3
17840	RERG	3.928	0.807	0.890	0.193	0.708	0.813	RAS like estrogen regulated growth inhibitor	RERG
15726	PARP14	3.918	0.237	0.886	0.152	0.146	0.397	poly(ADP-ribose) polymerase family member 14	PARP14
7871	H1F0	3.917	0.461	1.464	0.140	0.226	0.206	H1 histone family member 0	H1F0
18006	RIPK2	3.909	0.614	1.225	0.274	0.519	0.103	receptor interacting serine/threonine kinase 2	RIPK2
8636	IFI35	3.907	0.362	1.749	0.155	0.193	0.199	interferon induced protein 35	IFI35
19752	SMIM3	3.882	0.689	1.400	0.005	0.092	0.175	small integral membrane protein 3	SMIM3
19421	SLC2A6	3.878	0.459	0.991	0.402	0.522	0.989	solute carrier family 2 member 6	SLC2A6
22034	TNFSF9	3.858	0.309	0.519	0.315	0.355	0.099	tumor necrosis factor superfamily member 9	TNFSF9
13276	MEDAG	3.851	0.626	3.439	0.107	0.284	0.413	mesenteric estrogen dependent adipogenesis	MEDAG
1362	ATOH8	3.845	3.816	7.168	0.002	0.086	0.367	atonal bHLH transcription factor 8	ATOH8
15725	PARP12	3.828	0.444	1.267	0.221	0.317	0.294	poly(ADP-ribose) polymerase family member 12	PARP12
10999	LIPG	3.827	0.223	0.871	0.437	0.417	0.542	lipase G, endothelial type	LIPG
13879	MT1L	3.810	3.627	4.100	0.015	0.125	0.057	metallothionein 1L (gene/pseudogene)	MT1L
17467	RAB27B	3.809	0.544	1.323	0.070	0.301	0.643	RAB27B, member RAS oncogene family	RAB27B
21997	TNFAIP8	3.763	0.505	0.990	0.118	0.216	0.980	TNF alpha induced protein 8	TNFAIP8
16703	PPAP2B	3.755	0.278	1.406	0.255	0.267	0.500	.	PPAP2B
5777	EREG	3.733	0.723	0.951	0.434	0.779	0.695	epiregulin	EREG
16074	PF4V1	3.714	4.747	1.802	0.088	0.007	0.378	platelet factor 4 variant 1	PF4V1
21528	TIAM2	3.705	0.548	1.188	0.125	0.265	0.631	T-cell lymphoma invasion and metastasis 2	TIAM2
8791	IL1A	3.698	0.360	1.018	0.377	0.429	0.323	interleukin 1 alpha	IL1A
19352	SLC25A28	3.693	0.801	1.704	0.037	0.317	0.469	solute carrier family 25 member 28	SLC25A28
18591	SAMHD1	3.686	0.425	0.974	0.258	0.343	0.884	SAM and HD domain containing deoxynucleoside triphosphate triphc	SAMHD1
16844	PPP4R4	3.685	0.519	1.109	0.096	0.273	0.833	protein phosphatase 4, regulatory subunit 4	PPP4R4
732	ANGPT1	3.685	0.623	1.203	0.026	0.237	0.753	angiopoietin 1	ANGPT1
18089	RNF144B	3.673	0.481	0.983	0.164	0.265	0.959	ring finger protein 144B	RNF144B
3518	CHMP1B	3.673	1.284	1.967	0.020	0.048	0.466	charged multivesicular body protein 1B	CHMP1B
17639	RASD1	3.673	0.761	2.611	0.115	0.452	0.368	ras related dexamethasone induced 1	RASD1
4034	CPD	3.672	0.367	0.875	0.002	0.010	0.350	carboxypeptidase D	CPD
16025	PDZRN3	3.653	0.447	0.705	0.238	0.326	0.522	PDZ domain containing ring finger 3	PDZRN3
376	ADGRG1	3.635	0.550	1.660	0.363	0.547	0.530	adhesion G protein-coupled receptor G1	ADGRG1
17422	QPCT	3.628	0.948	0.572	0.002	0.483	0.131	glutaminyl-peptide cyclotransferase	QPCT
3005	CCR7	3.618	1.077	2.219	0.313	0.910	0.485	C-C motif chemokine receptor 7	CCR7
15668	PALM2-AKAP2	3.614	0.874	1.251	0.002	0.620	0.643	PALM2-AKAP2 readthrough	PALM2-AKAP2
1385	ATP1B1	3.611	0.883	1.158	0.006	0.663	0.434	ATPase Na+/K+ transporting subunit beta 1	ATP1B1
19316	SLC22A4	3.609	1.379	1.348	0.202	0.561	0.350	solute carrier family 22 member 4	SLC22A4
8604	ID1	3.604	24.939	75.872	0.112	0.182	0.405	inhibitor of DNA binding 1, HLH protein	ID1
13880	MT1M	3.604	1.390	2.244	0.318	0.675	0.272	metallothionein 1M	MT1M
8136	HIST1H1C	3.598	1.753	2.234	0.113	0.404	0.100	histone cluster 1, H1c	HIST1H1C
7530	GPD2	3.593	0.662	1.236	0.081	0.306	0.245	glycerol-3-phosphate dehydrogenase 2	GPD2
1651	BCL3	3.584	0.781	2.299	0.015	0.249	0.029	B-cell CLL/lymphoma 3	BCL3
23143	VMO1	3.578	1.896	21.835	0.315	0.437	0.427	vitelline membrane outer layer 1 homolog	VMO1
14771	NR4A3	3.578	0.498	1.012	0.019	0.035	0.923	nuclear receptor subfamily 4 group A member 3	NR4A3
19778	SMTN	3.575	0.868	1.351	0.017	0.318	0.316	smoothelin	SMTN
8218	HLA-C	3.554	0.646	0.810	0.134	0.402	0.311	major histocompatibility complex, class I, C	HLA-C
19553	SLC5A3	3.551	0.320	0.836	0.032	0.027	0.622	solute carrier family 5 member 3	SLC5A3
6545	FGF1	3.513	0.350	1.934	0.492	0.526	0.612	fibroblast growth factor 1	FGF1
8163	HIST1H2BK	3.513	0.829	1.314	0.406	0.856	0.659	histone cluster 1, H2bk	HIST1H2BK
18487	RTP4	3.499	0.270	1.052	0.194	0.184	0.866	receptor (chemosensory) transporter protein 4	RTP4
6551	FGF13	3.491	0.410	0.749	0.395	0.470	0.639	fibroblast growth factor 13	FGF13
7733	GSAP	3.486	0.588	1.139	0.138	0.300	0.728	gamma-secretase activating protein	GSAP
7619	GPR84	3.485	0.418	1.000	0.428	0.512	0.992	G protein-coupled receptor 84	GPR84
23421	WWC1	3.484	0.652	1.578	0.004	0.018	0.157	WW and C2 domain containing 1	WWC1
22263	TRIM14	3.459	0.489	1.635	0.305	0.423	0.519	tripartite motif containing 14	TRIM14
8607	ID3	3.437	5.720	13.304	0.047	0.043	0.346	inhibitor of DNA binding 3, HLH protein	ID3
3119	CDA	3.437	0.717	3.474	0.131	0.499	0.026	cytidine deaminase	CDA
19577	SLC6A6	3.424	0.273	0.963	0.071	0.056	0.916	solute carrier family 6 member 6	SLC6A6
9097	ITPRIP	3.408	1.788	2.969	0.195	0.177	0.272	inositol 1,4,5-trisphosphate receptor interacting protein	ITPRIP
20511	SPON1	3.404	0.815	4.241	0.497	0.870	0.463	spondin 1	SPON1
4753	DEFB1	3.393	0.652	0.650	0.026	0.093	0.468	defensin beta 1	DEFB1
3096	CD68	3.391	0.615	1.346	0.125	0.315	0.231	CD68 molecule	CD68
14267	NCEH1	3.386	1.251	1.432	0.322	0.733	0.615	neutral cholesterol ester hydrolase 1	NCEH1
578	AKR1C2	3.358	0.478	1.112	0.325	0.439	0.894	aldo-keto reductase family 1, member C2	AKR1C2
5270	DTX2	3.356	0.452	1.071	0.322	0.419	0.531	deltex 2, E3 ubiquitin ligase	DTX2

1597	BATF2	3.344	0.395	1.069	0.395	0.450	0.867	basic leucine zipper ATF-like transcription factor 2	BATF2
1596	BATF	3.339	1.440	1.229	0.174	0.492	0.737	basic leucine zipper ATF-like transcription factor	BATF
4732	DDX60	3.337	0.255	0.834	0.094	0.085	0.428	DEXD/H-box helicase 60	DDX60
16271	PIM3	3.325	0.392	1.154	0.143	0.174	0.532	Pim-3 proto-oncogene, serine/threonine kinase	PIM3
6618	FKBP11	3.324	1.097	2.544	0.016	0.464	0.194	FK506 binding protein 11	FKBP11
1212	ARRDC3	3.319	0.381	1.435	0.006	0.011	0.320	arrestin domain containing 3	ARRDC3
11297	LOC10050617	3.318	0.651	1.009	0.025	0.101	0.842	uncharacterized LOC100506178	LOC100506178
23216	VTN	3.306	0.369	0.953	0.442	0.484	0.461	vitronectin	VTN
21102	TAP1	3.299	0.647	1.293	0.226	0.479	0.436	transporter 1, ATP-binding cassette, sub-family B (MDR/TAP)	TAP1
2497	CA2	3.292	5.200	6.420	0.351	0.101	0.382	carbonic anhydrase 2	CA2
6390	FBLN5	3.279	0.243	0.622	0.047	0.041	0.007	fibulin 5	FBLN5
7167	GDF6	3.278	0.721	1.310	0.387	0.701	0.545	growth differentiation factor 6	GDF6
5601	ELOVL7	3.274	0.481	1.048	0.320	0.433	0.035	ELOVL fatty acid elongase 7	ELOVL7
14800	NRIR	3.264	0.330	1.000	0.303	0.315	0.943	negative regulator of interferon response (non-protein coding)	NRIR
13484	MIR155HG	3.258	0.350	0.817	0.149	0.172	0.463	MIR155 host gene	MIR155HG
20691	ST3GAL1	3.253	0.832	1.428	0.022	0.601	0.482	ST3 beta-galactoside alpha-2,3-sialyltransferase 1	ST3GAL1
5264	DTNA	3.245	0.618	1.168	0.086	0.224	0.556	dystrobrevin alpha	DTNA
9018	ISG20	3.236	0.332	0.765	0.291	0.309	0.328	interferon stimulated exonuclease gene 20kDa	ISG20
5506	EIF1B	3.229	0.437	0.878	0.316	0.400	0.749	eukaryotic translation initiation factor 1B	EIF1B
1719	BID	3.228	0.573	1.212	0.327	0.511	0.604	BH3 interacting domain death agonist	BID
8989	IRF1	3.227	0.473	1.329	0.188	0.265	0.499	interferon regulatory factor 1	IRF1
11397	LOC10050763	3.217	0.441	1.055	0.007	0.021	0.692	uncharacterized LOC100507639	LOC100507639
3427	CFAP69	3.205	0.361	1.381	0.175	0.195	0.345	cilia and flagella associated protein 69	CFAP69
7601	GPR37L1	3.193	0.380	1.033	0.302	0.339	0.553	G protein-coupled receptor 37 like 1	GPR37L1
7121	GBP4	3.187	0.419	1.136	0.375	0.439	0.101	guanylate binding protein 4	GBP4
989	APOL3	3.179	0.457	1.612	0.269	0.364	0.069	apolipoprotein L3	APOL3
19409	SLC2A1	3.177	0.205	0.613	0.044	0.035	0.507	solute carrier family 2 member 1	SLC2A1
9965	LCTL	3.177	0.543	1.087	0.004	0.002	0.639	lactase like	LCTL
18932	SERPINA3	3.172	0.695	1.010	0.222	0.566	0.988	serpin family A member 3	SERPINA3
13321	MET	3.167	0.739	0.957	0.420	0.732	0.960	MET proto-oncogene, receptor tyrosine kinase	MET
23385	WNK4	3.167	0.728	1.047	0.109	0.414	0.181	WNK lysine deficient protein kinase 4	WNK4
21753	TMEM171	3.163	0.195	0.692	0.476	0.414	0.626	transmembrane protein 171	TMEM171
4619	DCBLD1	3.149	0.920	1.047	0.104	0.784	0.807	discoidin, CUB and LCCL domain containing 1	DCBLD1
19767	SMOX	3.132	4.223	6.400	0.031	0.081	0.415	spermine oxidase	SMOX
8300	HNMT	3.132	0.870	1.522	0.003	0.605	0.566	histamine N-methyltransferase	HNMT
15734	PARP9	3.120	0.258	1.022	0.187	0.164	0.934	poly(ADP-ribose) polymerase family member 9	PARP9
7075	GAS1	3.119	1.324	2.661	0.458	0.803	0.417	growth arrest specific 1	GAS1
14110	MYPN	3.115	1.649	2.432	0.015	0.516	0.055	myopalladin	MYPN
16927	PREX1	3.112	1.325	0.957	0.065	0.498	0.947	phosphatidylinositol-3,4,5-trisphosphate dependent Rac exchange fa	PREX1
3287	CEBPB	3.111	1.077	1.999	0.022	0.762	0.340	CCAAT/enhancer binding protein beta	CEBPB
3397	CES1	3.108	0.244	0.624	0.234	0.204	0.060	carboxylesterase 1	CES1
7460	GOLGA7B	3.096	0.497	1.159	0.208	0.325	0.552	golgin A7 family member B	GOLGA7B
18590	SAMD9L	3.094	0.252	0.811	0.320	0.280	0.711	sterile alpha motif domain containing 9 like	SAMD9L
15488	ORM1	3.084	0.324	1.000	0.001	0.001	0.943	orosomucoid 1	ORM1
14873	NUAK2	3.079	1.215	1.288	0.025	0.143	0.386	NUAK family kinase 2	NUAK2
17179	PSMA6	3.071	0.909	1.337	0.214	0.855	0.395	proteasome subunit alpha 6	PSMA6
23379	WISP3	3.070	0.707	1.034	0.118	0.369	0.885	WNT1 inducible signaling pathway protein 3	WISP3
16026	PDZRN3-AS1	3.059	0.532	0.904	0.004	0.019	0.623	PDZRN3 antisense RNA 1	PDZRN3-AS1
6736	FNDC3B	3.049	0.528	0.964	0.047	0.089	0.766	fibronectin type III domain containing 3B	FNDC3B
7041	GALNT15	3.049	0.926	2.069	0.360	0.923	0.499	polypeptide N-acetylgalactosaminyltransferase 15	GALNT15
5306	DUSP6	3.038	0.457	0.713	0.390	0.445	0.716	dual specificity phosphatase 6	DUSP6
15604	P4HA3	3.034	1.607	2.222	0.268	0.412	0.536	prolyl 4-hydroxylase subunit alpha 3	P4HA3
14642	NOMO2	3.024	1.106	1.953	0.005	0.292	0.253	NODAL modulator 2	NOMO2
6155	FAM20A	3.023	0.515	1.640	0.337	0.460	0.106	family with sequence similarity 20 member A	FAM20A
8240	HLA-H	3.019	0.467	0.825	0.060	0.068	0.777	major histocompatibility complex, class I, H (pseudogene)	HLA-H
5993	FAM110B	3.012	0.470	1.131	0.048	0.068	0.689	family with sequence similarity 110 member B	FAM110B
18589	SAMD9	3.007	0.269	0.772	0.364	0.324	0.646	sterile alpha motif domain containing 9	SAMD9
20542	SPRY4	3.007	0.913	2.418	0.123	0.767	0.382	sprouty RTK signaling antagonist 4	SPRY4
15995	PDLIM4	3.003	0.780	1.581	0.141	0.535	0.200	PDZ and LIM domain 4	PDLIM4
3460	CGNL1	3.000	0.324	0.572	0.229	0.214	0.468	cingulin-like 1	CGNL1
17265	PTGER2	3.000	0.803	1.691	0.354	0.764	0.404	prostaglandin E receptor 2	PTGER2
7987	HCP5	2.997	0.514	0.767	0.459	0.577	0.378	HLA complex P5 (non-protein coding)	HCP5
3057	CD274	2.996	0.455	0.968	0.297	0.370	0.758	CD274 molecule	CD274
21103	TAP2	2.994	0.765	1.478	0.221	0.600	0.040	transporter 2, ATP-binding cassette, sub-family B (MDR/TAP)	TAP2
202	ACSL1	2.994	0.609	0.830	0.224	0.426	0.163	acyl-CoA synthetase long-chain family member 1	ACSL1
19094	SH3RF2	2.994	0.984	1.083	0.005	0.914	0.691	SH3 domain containing ring finger 2	SH3RF2
10273	LINC00473	2.978	0.755	1.044	0.309	0.721	0.416	long intergenic non-protein coding RNA 473	LINC00473
506	AGTRAP	2.968	1.003	1.847	0.060	0.989	0.343	angiotensin II receptor associated protein	AGTRAP
1326	ATF3	2.964	0.273	0.861	0.188	0.166	0.407	activating transcription factor 3	ATF3
8895	INO80C	2.961	0.913	1.387	0.237	0.845	0.344	INO80 complex subunit C	INO80C
19363	SLC25A37	2.956	0.733	2.189	0.086	0.432	0.468	solute carrier family 25 member 37	SLC25A37
13046	MAP3K8	2.950	0.413	0.937	0.034	0.032	0.825	mitogen-activated protein kinase kinase kinase 8	MAP3K8
13214	MCTP2	2.948	1.489	1.923	0.054	0.572	0.494	multiple C2 domains, transmembrane 2	MCTP2
7097	GATA6	2.945	0.735	2.134	0.266	0.620	0.260	GATA binding protein 6	GATA6
7117	GBP1	2.943	0.360	0.780	0.147	0.149	0.648	guanylate binding protein 1	GBP1
8216	HLA-A	2.932	0.693	0.917	0.034	0.065	0.816	major histocompatibility complex, class I, A	HLA-A
6709	FLVCR2	2.931	0.675	1.014	0.006	0.033	0.957	feline leukemia virus subgroup C cellular receptor family member 2	FLVCR2
15949	PDE3A	2.927	0.900	1.445	0.285	0.844	0.346	phosphodiesterase 3A	PDE3A
19266	SLC16A3	2.925	0.680	1.577	0.006	0.081	0.157	solute carrier family 16 member 3	SLC16A3
20352	SP6	2.925	0.405	1.071	0.220	0.252	0.361	Sp6 transcription factor	SP6
15994	PDLIM3	2.924	0.361	1.024	0.372	0.382	0.945	PDZ and LIM domain 3	PDLIM3
4675	DDIT4	2.920	0.704	2.019	0.013	0.104	0.257	DNA damage inducible transcript 4	DDIT4
21675	TMEM100	2.914	1.072	2.358	0.035	0.701	0.373	transmembrane protein 100	TMEM100
9850	LACC1	2.909	0.523	1.001	0.204	0.309	0.997	laccase domain containing 1	LACC1
9230	KCNE4	2.908	0.346	0.910	0.194	0.188	0.738	potassium voltage-gated channel subfamily E regulatory subunit 4	KCNE4
13299	MEIS1-AS2	2.900	0.631	1.053	0.017	0.207	0.837	MEIS1 antisense RNA 2	MEIS1-AS2
1375	ATP13A3	2.893	0.484	0.746	0.031	0.052	0.488	ATPase 13A3	ATP13A3
1896	BTG3	2.888	0.973	0.879	0.157	0.935	0.658	BTG family member 3	BTG3
15670	PALMD	2.885	0.939	1.249	0.086	0.809	0.382	palmdelphin	PALMD
9237	KCNH1	2.873	0.631	1.252	0.208	0.408	0.626	potassium voltage-gated channel subfamily H member 1	KCNH1
23772	ZMIZ1-AS1	2.869	0.470	1.119	0.206	0.270	0.820	ZMIZ1 antisense RNA 1	ZMIZ1-AS1

8667	IFNAR2	2.862	0.730	1.319	0.195	0.504	0.351	interferon alpha and beta receptor subunit 2	IFNAR2
12214	LOC149684	2.862	0.481	1.645	0.015	0.021	0.406	uncharacterized LOC149684	LOC149684
5805	ERN1	2.861	0.415	0.976	0.196	0.228	0.933	endoplasmic reticulum to nucleus signaling 1	ERN1
14265	NCCRP1	2.851	0.415	0.987	0.275	0.312	0.426	non-specific cytotoxic cell receptor protein 1 homolog (zebrafish)	NCCRP1
6885	FTH1	2.842	0.641	0.620	0.012	0.094	0.155	ferritin, heavy polypeptide 1	FTH1
938	APCDD1L	2.842	0.537	1.214	0.285	0.395	0.719	adenomatosis polyposis coli down-regulated 1 like	APCDD1L
1514	B2M	2.841	0.642	0.834	0.035	0.098	0.181	beta-2-microglobulin	B2M
20936	SUSD6	2.824	0.606	1.208	0.089	0.236	0.153	sushi domain containing 6	SUSD6
9446	KIAA1755	2.818	0.521	1.640	0.179	0.270	0.019	KIAA1755	KIAA1755
6935	FYN	2.816	0.741	1.453	0.023	0.154	0.405	FYN proto-oncogene, Src family tyrosine kinase	FYN
21426	TFPI	2.805	0.974	2.019	0.129	0.940	0.019	tissue factor pathway inhibitor	TFPI
10741	LINC01272	2.794	0.429	0.753	0.487	0.539	0.348	long intergenic non-protein coding RNA 1272	LINC01272
8701	IGDCC4	2.792	0.369	0.708	0.035	0.026	0.314	immunoglobulin superfamily DCC subclass member 4	IGDCC4
13175	MCAM	2.788	1.161	1.604	0.376	0.860	0.608	melanoma cell adhesion molecule	MCAM
8649	IFITM3	2.783	0.447	1.539	0.032	0.016	0.497	interferon induced transmembrane protein 3	IFITM3
14643	NOMO3	2.782	1.121	1.851	0.021	0.572	0.229	NODAL modulator 3	NOMO3
120	ABL1	2.781	0.748	1.238	0.003	0.286	0.674	ABL proto-oncogene 1, non-receptor tyrosine kinase	ABL1
17275	PTGFR	2.776	0.488	1.931	0.023	0.038	0.403	prostaglandin F receptor	PTGFR
8815	IL24	2.774	0.429	1.016	0.318	0.360	0.432	interleukin 24	IL24
5017	DMXL2	2.761	0.475	1.030	0.230	0.295	0.780	Dmx like 2	DMXL2
1771	BMPR1B	2.754	0.563	1.131	0.417	0.575	0.560	bone morphogenetic protein receptor type 1B	BMPR1B
4580	DAZL	2.751	0.472	1.040	0.385	0.459	0.504	deleted in azoospermia like	DAZL
14601	NMI	2.750	0.596	1.311	0.181	0.331	0.132	N-myc and STAT interactor	NMI
16083	PFKFB3	2.746	0.311	1.003	0.021	0.006	0.992	6-phosphofructo-2-kinase/fructose-2,6-biphosphatase 3	PFKFB3
5273	DTX3L	2.743	0.297	1.013	0.120	0.101	0.929	deltex 3 like, E3 ubiquitin ligase	DTX3L
13881	MT1X	2.740	2.972	2.372	0.021	0.188	0.095	metallothionein 1X	MT1X
12856	LYN	2.736	0.435	0.617	0.041	0.054	0.100	LYN proto-oncogene, Src family tyrosine kinase	LYN
21442	TGFBR1	2.728	0.579	0.892	0.371	0.538	0.823	transforming growth factor beta receptor 1	TGFBR1
21585	TLCD1	2.724	0.635	1.248	0.195	0.411	0.451	TLC domain containing 1	TLCD1
19660	SLPI	2.721	0.431	0.476	0.452	0.483	0.604	secretory leukocyte peptidase inhibitor	SLPI
5963	FADS1	2.720	0.194	0.286	0.525	0.413	0.439	fatty acid desaturase 1	FADS1
1628	BCAT1	2.712	0.536	0.881	0.008	0.057	0.297	branched chain amino acid transaminase 1	BCAT1
4733	DDX60L	2.711	0.277	0.700	0.205	0.168	0.069	DEAD-box helicase 60-like	DDX60L
23152	VNN3	2.708	1.068	1.000	0.020	0.852	0.943	vanin 3	VNN3
23332	WDR86	2.703	0.347	2.145	0.430	0.418	0.493	WD repeat domain 86	WDR86
19207	SKIL	2.701	0.723	1.171	0.076	0.349	0.541	SKI-like proto-oncogene	SKIL
19487	SLC38A5	2.698	1.326	2.258	0.005	0.617	0.030	solute carrier family 38 member 5	SLC38A5
20888	SULF1	2.694	1.255	1.535	0.333	0.649	0.301	sulfatase 1	SULF1
21104	TAPBP	2.690	0.654	1.224	0.196	0.410	0.201	TAP binding protein (tapasin)	TAPBP
5128	DOCK10	2.688	0.806	1.745	0.449	0.807	0.354	dedicator of cytokinesis 10	DOCK10
4190	CSF1	2.684	0.588	1.236	0.037	0.084	0.687	colony stimulating factor 1	CSF1
3782	CMIP	2.684	1.185	1.723	0.086	0.708	0.324	c-Maf inducing protein	CMIP
20694	ST3GAL4	2.674	0.858	1.161	0.276	0.790	0.490	ST3 beta-galactoside alpha-2,3-sialyltransferase 4	ST3GAL4
13177	MCC	2.674	0.566	1.477	0.098	0.179	0.250	mutated in colorectal cancers	MCC
13655	MOXD1	2.673	1.067	1.371	0.045	0.768	0.460	monooxygenase DBH like 1	MOXD1
20336	SOX5	2.667	0.476	1.153	0.164	0.238	0.681	SRY-box 5	SOX5
15597	P3H2	2.662	1.830	2.582	0.009	0.148	0.245	prolyl 3-hydroxylase 2	P3H2
19178	SIRPA	2.662	0.648	0.787	0.170	0.423	0.622	signal regulatory protein alpha	SIRPA
19899	SNORA4	2.659	0.492	0.138	0.597	0.675	0.423	small nucleolar RNA, H/ACA box 4	SNORA4
14464	NFKB1	2.658	0.559	1.138	0.217	0.337	0.007	nuclear factor of kappa light polypeptide gene enhancer in B-cells 1	NFKB1
17824	RELB	2.654	0.787	1.630	0.081	0.298	0.251	RELB proto-oncogene, NF-kB subunit	RELB
22036	TNIP1	2.654	0.657	1.161	0.218	0.432	0.204	TNFAIP3 interacting protein 1	TNIP1
16084	PFKFB4	2.653	0.443	0.725	0.015	0.012	0.228	6-phosphofructo-2-kinase/fructose-2,6-biphosphatase 4	PFKFB4
5510	EIF2AK2	2.652	0.343	0.719	0.142	0.128	0.254	eukaryotic translation initiation factor 2 alpha kinase 2	EIF2AK2
3242	CDKN3	2.647	7.375	6.385	0.367	0.159	0.112	cyclin-dependent kinase inhibitor 3	CDKN3
3289	CEBPD	2.646	0.645	1.491	0.028	0.074	0.295	CCAAT/enhancer binding protein delta	CEBPD
19681	SMAD7	2.645	0.825	1.054	0.034	0.341	0.861	SMAD family member 7	SMAD7
15517	OSMR	2.638	0.365	1.112	0.123	0.119	0.351	oncostatin M receptor	OSMR
20757	STAT1	2.629	0.519	1.046	0.041	0.025	0.917	signal transducer and activator of transcription 1	STAT1
4548	DACT1	2.618	0.927	0.825	0.135	0.777	0.694	dishevelled binding antagonist of beta catenin 1	DACT1
11042	LMO4	2.616	1.094	3.081	0.000	0.705	0.162	LIM domain only 4	LMO4
22265	TRIM16	2.615	0.545	1.176	0.080	0.145	0.578	tripartite motif containing 16	TRIM16
8787	IL18BP	2.607	0.894	1.595	0.104	0.724	0.268	interleukin 18 binding protein	IL18BP
8843	IL7	2.607	0.537	1.040	0.251	0.352	0.724	interleukin 7	IL7
4540	DAAM1	2.603	0.621	1.056	0.089	0.192	0.739	dishevelled associated activator of morphogenesis 1	DAAM1
9528	KITLG	2.602	0.569	1.233	0.325	0.521	0.710	KIT ligand	KITLG
22198	TRAF1	2.593	0.422	0.999	0.376	0.403	0.998	TNF receptor associated factor 1	TRAF1
4030	CPAMD8	2.589	0.410	0.954	0.057	0.062	0.383	C3 and PZP like, alpha-2-macroglobulin domain containing 8	CPAMD8
1689	BEST1	2.586	0.552	1.039	0.120	0.197	0.611	bestrophin 1	BEST1
16712	PPARGC1A	2.584	0.524	0.828	0.131	0.198	0.713	PPARG coactivator 1 alpha	PPARGC1A
3912	COL3A1	2.583	0.465	1.272	0.421	0.475	0.703	collagen type III alpha 1	COL3A1
8191	HIST2H2AA4	2.577	0.869	1.346	0.278	0.783	0.218	histone cluster 2, H2aa4	HIST2H2AA4
1806	BPI	2.575	0.374	0.888	0.007	0.012	0.319	bactericidal/permeability-increasing protein	BPI
18325	RPLP0P2	2.573	0.817	1.118	0.496	0.834	0.675	ribosomal protein lateral stalk subunit P0 pseudogene 2	RPLP0P2
16178	PHLDA2	2.570	0.530	1.111	0.424	0.522	0.845	pleckstrin homology like domain family A member 2	PHLDA2
20340	SOX9	2.565	0.354	0.759	0.245	0.229	0.424	SRY-box 9	SOX9
6212	FAM26F	2.563	0.596	1.642	0.096	0.311	0.360	family with sequence similarity 26 member F	FAM26F
14315	NDRG1	2.560	0.285	0.816	0.069	0.050	0.534	N-myc downstream regulated 1	NDRG1
16087	PFKP	2.556	1.496	1.555	0.024	0.127	0.168	phosphofructokinase, platelet	PFKP
14555	NKX3-1	2.551	2.822	2.253	0.133	0.319	0.364	NK3 homeobox 1	NKX3-1
20370	SPAG4	2.545	0.449	1.400	0.228	0.258	0.036	sperm associated antigen 4	SPAG4
15927	PDCD1LG2	2.540	0.788	0.841	0.024	0.500	0.773	programmed cell death 1 ligand 2	PDCD1LG2
10088	LIMK2	2.539	0.555	1.151	0.231	0.352	0.222	LIM domain kinase 2	LIMK2
1042	ARFGEF3	2.539	0.260	0.704	0.505	0.424	0.415	ARFGEF family member 3	ARFGEF3
6232	FAM46A	2.536	0.456	0.804	0.062	0.058	0.661	family with sequence similarity 46 member A	FAM46A
10646	LINC01137	2.532	0.675	1.172	0.222	0.443	0.776	long intergenic non-protein coding RNA 1137	LINC01137
22266	TRIM16L	2.532	0.516	0.910	0.297	0.382	0.746	tripartite motif containing 16-like	TRIM16L
9257	KCNJ15	2.528	0.601	1.539	0.200	0.361	0.581	potassium voltage-gated channel subfamily J member 15	KCNJ15
1540	B4GALT1	2.524	1.041	1.340	0.002	0.136	0.417	beta-1,4-galactosyltransferase 1	B4GALT1
14430	NEURL2	2.524	0.298	0.962	0.260	0.210	0.896	neuralized E3 ubiquitin protein ligase 2	NEURL2
7627	GPRC5A	2.524	0.751	1.826	0.131	0.642	0.049	G protein-coupled receptor class C group 5 member A	GPRC5A

13426	MGST1	2.523	0.786	0.895	0.265	0.674	0.640	microsomal glutathione S-transferase 1	MGST1
5132	DOCK4	2.523	0.440	0.584	0.206	0.196	0.390	dedicator of cytokinesis 4	DOCK4
11099	LOC10012951	2.517	0.369	0.791	0.073	0.060	0.372	uncharacterized LOC100129518	LOC100129518
8645	IFIT5	2.514	0.397	0.772	0.122	0.121	0.255	interferon induced protein with tetratricopeptide repeats 5	IFIT5
414	ADORA2A-AS1	2.509	0.624	1.079	0.353	0.534	0.592	ADORA2A antisense RNA 1	ADORA2A-AS1
17335	PTPRN	2.506	1.034	1.341	0.065	0.909	0.472	protein tyrosine phosphatase, receptor type N	PTPRN
4827	DFNA5	2.505	0.336	0.746	0.455	0.413	0.608	DFNA5, deafness associated tumor suppressor	DFNA5
42	ABCA1	2.502	0.589	1.737	0.306	0.445	0.405	ATP binding cassette subfamily A member 1	ABCA1
19311	SLC22A23	2.500	0.593	0.698	0.155	0.391	0.298	solute carrier family 22 member 23	SLC22A23
20725	STAC2	2.500	0.497	1.703	0.390	0.459	0.492	SH3 and cysteine rich domain 2	STAC2
6599	FIBIN	2.497	0.413	0.782	0.147	0.086	0.775	fin bud initiation factor homolog (zebrafish)	FIBIN
4676	DDIT4L	2.494	0.237	2.037	0.219	0.077	0.635	DNA damage inducible transcript 4 like	DDIT4L
5367	EBI3	2.494	0.452	1.049	0.320	0.353	0.425	Epstein-Barr virus induced 3	EBI3
6013	FAM126A	2.488	0.701	0.941	0.053	0.256	0.792	family with sequence similarity 126 member A	FAM126A
3495	CHEK2	2.484	0.925	1.705	0.014	0.290	0.294	checkpoint kinase 2	CHEK2
19627	SLCO3A1	2.481	0.424	0.923	0.002	0.002	0.314	solute carrier organic anion transporter family member 3A1	SLCO3A1
666	ALPK3	2.481	0.691	1.084	0.239	0.476	0.648	alpha kinase 3	ALPK3
11944	LOC10192931	2.478	0.553	1.088	0.031	0.053	0.459	uncharacterized LOC101929319	LOC101929319
4844	DGKD	2.475	0.896	1.498	0.187	0.763	0.425	diacylglycerol kinase delta	DGKD
8145	HIST1H2AG	2.472	0.769	1.829	0.161	0.535	0.147	histone cluster 1, H2ag	HIST1H2AG
15601	P4HA1	2.472	0.229	0.600	0.364	0.265	0.273	prolyl 4-hydroxylase subunit alpha 1	P4HA1
9843	L3HYPDH	2.470	0.648	1.035	0.036	0.154	0.926	trans-L-3-hydroxyproline dehydratase	L3HYPDH
10203	LINC00342	2.467	0.245	0.551	0.302	0.221	0.118	long intergenic non-protein coding RNA 342	LINC00342
16319	PKD1P1	2.461	1.028	1.291	0.332	0.959	0.645	polycystin 1, transient receptor potential channel interacting pseudo	PKD1P1
20740	STAMBPL1	2.461	3.936	2.444	0.024	0.057	0.211	STAM binding protein like 1	STAMBPL1
5285	DUSP1	2.459	2.225	1.466	0.080	0.070	0.327	dual specificity phosphatase 1	DUSP1
4397	CXCL16	2.455	0.729	1.945	0.498	0.770	0.668	C-X-C motif chemokine ligand 16	CXCL16
14037	MYH11	2.455	0.408	0.964	0.218	0.219	0.018	myosin, heavy chain 11, smooth muscle	MYH11
8498	HSP90B1	2.453	1.145	1.643	0.192	0.679	0.437	heat shock protein 90kDa beta family member 1	HSP90B1
7119	GBP2	2.453	0.482	1.111	0.170	0.213	0.471	guanylate binding protein 2	GBP2
21478	THBS1	2.448	1.842	1.509	0.187	0.211	0.355	thrombospondin 1	THBS1
8195	HIST2H2BC	2.447	0.621	1.602	0.097	0.260	0.071	histone cluster 2, H2bc (pseudogene)	HIST2H2BC
21441	TGFBI	2.446	1.044	2.070	0.467	0.952	0.381	transforming growth factor beta induced	TGFBI
8718	IGFBP7	2.443	0.611	0.609	0.317	0.464	0.499	insulin like growth factor binding protein 7	IGFBP7
15932	PDCD5	2.441	1.571	1.542	0.034	0.108	0.001	programmed cell death 5	PDCD5
1901	BTN2A1	2.440	0.850	1.155	0.009	0.112	0.328	butyrophilin subfamily 2 member A1	BTN2A1
22024	TNFSF11	2.438	1.442	3.409	0.340	0.651	0.422	tumor necrosis factor superfamily member 11	TNFSF11
21888	TMEM51	2.438	0.990	1.160	0.429	0.988	0.353	transmembrane protein 51	TMEM51
18935	SERPINA6	2.432	0.476	1.017	0.058	0.079	0.827	serpin family A member 6	SERPINA6
21491	THEMIS2	2.429	0.435	1.441	0.123	0.130	0.416	thymocyte selection associated family member 2	THEMIS2
168	ACKR3	2.429	0.380	0.537	0.040	0.003	0.275	atypical chemokine receptor 3	ACKR3
8212	HK2	2.424	0.327	0.993	0.010	0.006	0.946	hexokinase 2	HK2
2144	C1GALT1C1	2.423	1.152	1.702	0.071	0.574	0.290	C1GALT1 specific chaperone 1	C1GALT1C1
15684	PANX2	2.422	0.573	1.038	0.298	0.443	0.881	pannexin 2	PANX2
19628	SLCO4A1	2.421	0.458	0.904	0.445	0.477	0.119	solute carrier organic anion transporter family member 4A1	SLCO4A1
18840	SEL1L3	2.420	0.419	1.159	0.087	0.102	0.073	SEL1L family member 3	SEL1L3
9569	KLHL13	2.418	1.696	1.713	0.410	0.358	0.289	kelch like family member 13	KLHL13
5214	DRAM1	2.410	0.540	0.775	0.224	0.308	0.601	DNA damage regulated autophagy modulator 1	DRAM1
13586	MMP2	2.407	0.476	0.709	0.075	0.076	0.461	matrix metallopeptidase 2	MMP2
12989	MAML2	2.403	0.604	0.906	0.186	0.285	0.805	mastermind like transcriptional coactivator 2	MAML2
12608	LOXL3	2.400	1.378	1.679	0.399	0.562	0.362	lysyl oxidase like 3	LOXL3
8715	IGFBP4	2.400	1.139	2.865	0.497	0.867	0.521	insulin like growth factor binding protein 4	IGFBP4
19639	SLFN5	2.399	0.398	0.889	0.336	0.280	0.856	schlafen family member 5	SLFN5
20992	SYNJ2	2.398	0.481	1.141	0.049	0.062	0.366	synaptojanin 2	SYNJ2
10350	LINC00602	2.394	0.634	1.280	0.269	0.454	0.422	long intergenic non-protein coding RNA 602	LINC00602
22202	TRAF3IP2	2.390	0.837	1.461	0.012	0.097	0.035	TRAF3 interacting protein 2	TRAF3IP2
22964	USP13	2.386	1.086	1.090	0.270	0.848	0.705	ubiquitin specific peptidase 13 (isopeptidase T-3)	USP13
2318	C3orf58	2.383	0.311	0.604	0.271	0.209	0.313	chromosome 3 open reading frame 58	C3orf58
8912	INSIG1	2.377	0.107	0.293	0.494	0.291	0.434	insulin induced gene 1	INSIG1
17425	QRFP	2.374	0.941	1.243	0.147	0.907	0.567	pyroglutamylated RFamide peptide	QRFP
131	ABTB2	2.370	0.778	1.376	0.146	0.455	0.556	ankyrin repeat and BTB domain containing 2	ABTB2
3164	CDCP1	2.369	1.460	1.654	0.114	0.167	0.246	CUB domain containing protein 1	CDCP1
21734	TMEM154	2.368	0.213	0.485	0.574	0.435	0.555	transmembrane protein 154	TMEM154
9048	ITGA5	2.367	1.417	2.734	0.029	0.079	0.005	integrin subunit alpha 5	ITGA5
18115	RNF19B	2.367	0.564	0.912	0.283	0.391	0.742	ring finger protein 19B	RNF19B
14311	NDNF	2.367	0.257	0.541	0.135	0.083	0.164	neuron-derived neurotrophic factor	NDNF
4447	CYCS	2.366	1.175	1.230	0.040	0.394	0.060	cytochrome c, somatic	CYCS
15737	PART1	2.359	1.225	1.076	0.001	0.646	0.676	prostate androgen-regulated transcript 1 (non-protein coding)	PART1
10858	LINC01465	2.357	0.530	1.129	0.020	0.010	0.787	long intergenic non-protein coding RNA 1465	LINC01465
12802	LSS	2.356	0.209	0.485	0.471	0.340	0.298	lanosterol synthase (2,3-oxidosqualene-lanosterol cyclase)	LSS
810	ANKRD36	2.355	0.324	0.672	0.230	0.183	0.064	ankyrin repeat domain 36	ANKRD36
14331	NDUFA4L2	2.353	0.100	0.545	0.556	0.371	0.501	NADH dehydrogenase (ubiquinone) 1 alpha subcomplex, 4-like 2	NDUFA4L2
5977	FAM101A	2.353	0.544	0.676	0.409	0.493	0.488	family with sequence similarity 101 member A	FAM101A
19106	SHB	2.349	0.648	1.459	0.296	0.469	0.284	SH2 domain containing adaptor protein B	SHB
4229	CSRNP1	2.347	0.866	1.363	0.102	0.535	0.432	cysteine and serine rich nuclear protein 1	CSRNP1
12673	LRP8	2.345	0.798	0.982	0.315	0.708	0.885	LDL receptor related protein 8	LRP8
9892	LAP3	2.344	0.633	1.250	0.339	0.510	0.120	leucine aminopeptidase 3	LAP3
1787	BNIP3	2.336	0.357	0.780	0.055	0.049	0.469	BCL2/adenovirus E1B 19kDa interacting protein 3	BNIP3
8185	HIST1H4H	2.335	0.952	1.829	0.489	0.952	0.039	histone cluster 1, H4h	HIST1H4H
16114	PGK1	2.334	0.683	1.170	0.083	0.211	0.400	phosphoglycerate kinase 1	PGK1
24407	ZSWIM4	2.332	0.524	0.992	0.057	0.076	0.973	zinc finger SWIM-type containing 4	ZSWIM4
6840	FRK	2.322	0.392	0.977	0.226	0.206	0.863	fyn related Src family tyrosine kinase	FRK
16741	PPIF	2.321	1.172	1.516	0.053	0.568	0.189	peptidylprolyl isomerase F	PPIF
4317	CTHRC1	2.321	1.332	1.185	0.325	0.499	0.773	collagen triple helix repeat containing 1	CTHRC1
17287	PTHLH	2.320	0.988	2.634	0.300	0.980	0.452	parathyroid hormone-like hormone	PTHLH
14029	MYDGF	2.319	1.214	1.588	0.007	0.259	0.260	myeloid-derived growth factor	MYDGF
8761	IL10RB-AS1	2.318	0.568	1.202	0.374	0.479	0.543	IL10RB antisense RNA 1 (head to head)	IL10RB-AS1
9976	LDLR	2.316	0.139	0.351	0.496	0.300	0.456	low density lipoprotein receptor	LDLR
13449	MID1	2.312	0.919	0.949	0.036	0.759	0.895	midline 1	MID1
9970	LDHA	2.312	0.776	1.759	0.203	0.538	0.029	lactate dehydrogenase A	LDHA
15983	PDIA4	2.310	1.247	1.533	0.036	0.280	0.329	protein disulfide isomerase family A member 4	PDIA4

5757	ERAP1	2.309	0.539	0.890	0.008	0.006	0.618	endoplasmic reticulum aminopeptidase 1	ERAP1
22431	TSKU	2.307	1.053	1.075	0.002	0.683	0.669	tsukushi, small leucine rich proteoglycan	TSKU
8502	HSPA13	2.305	0.645	0.812	0.024	0.045	0.333	heat shock protein family A (Hsp70) member 13	HSPA13
21235	TC2N	2.304	0.446	0.950	0.252	0.263	0.431	tandem C2 domains, nuclear	TC2N
3008	CCRL2	2.303	0.376	1.232	0.357	0.324	0.516	C-C motif chemokine receptor like 2	CCRL2
21430	TFRC	2.302	0.705	0.664	0.110	0.283	0.404	transferrin receptor	TFRC
15683	PANX1	2.298	0.719	1.171	0.027	0.112	0.166	pannexin 1	PANX1
12769	LRRN3	2.297	0.625	0.806	0.460	0.626	0.410	leucine rich repeat neuronal 3	LRRN3
18250	RPH3AL	2.296	0.911	1.219	0.077	0.843	0.618	rabphilin 3A-like (without C2 domains)	RPH3AL
9000	IRF9	2.294	0.372	0.923	0.106	0.087	0.754	interferon regulatory factor 9	IRF9
18769	SDC4	2.293	0.562	0.908	0.107	0.101	0.809	syndecan 4	SDC4
387	ADGRL4	2.291	0.803	1.034	0.501	0.824	0.947	adhesion G protein-coupled receptor L4	ADGRL4
15028	OGFRL1	2.291	0.617	0.722	0.120	0.269	0.077	opioid growth factor receptor-like 1	OGFRL1
17925	RGS5	2.290	0.349	0.975	0.452	0.396	0.928	regulator of G-protein signaling 5	RGS5
3644	CLCF1	2.288	1.224	1.668	0.061	0.551	0.167	cardiotrophin-like cytokine factor 1	CLCF1
328	ADAR	2.285	0.468	0.887	0.155	0.166	0.517	adenosine deaminase, RNA-specific	ADAR
21797	TMEM205	2.285	0.988	1.405	0.083	0.966	0.309	transmembrane protein 205	TMEM205
9156	JUNB	2.284	0.946	4.231	0.045	0.798	0.194	JunB proto-oncogene, AP-1 transcription factor subunit	JUNB
19425	SLC30A1	2.284	0.589	1.344	0.045	0.058	0.448	solute carrier family 30 member 1	SLC30A1
4892	DHX58	2.282	0.396	1.005	0.287	0.262	0.970	DEXH-box helicase 58	DHX58
4002	COX17	2.282	1.289	0.939	0.346	0.593	0.661	COX17 cytochrome c oxidase copper chaperone	COX17
14454	NFE2L1	2.280	0.549	0.922	0.013	0.019	0.461	nuclear factor, erythroid 2 like 1	NFE2L1
1756	BMP1	2.277	0.751	1.310	0.011	0.106	0.419	bone morphogenetic protein 1	BMP1
20289	SOCS3	2.276	1.158	2.729	0.001	0.680	0.115	suppressor of cytokine signaling 3	SOCS3
17015	PROCR	2.275	1.187	2.184	0.075	0.390	0.212	protein C receptor	PROCR
21744	TMEM164	2.273	1.227	1.709	0.118	0.524	0.306	transmembrane protein 164	TMEM164
3679	CLDND1	2.271	0.781	0.972	0.016	0.053	0.860	claudin domain containing 1	CLDND1
18059	RND3	2.266	0.824	1.323	0.421	0.796	0.615	Rho family GTPase 3	RND3
17423	QPCTL	2.265	1.073	1.226	0.203	0.852	0.468	glutaminyl-peptide cyclotransferase-like	QPCTL
6157	FAM20C	2.264	1.461	2.813	0.061	0.184	0.219	family with sequence similarity 20 member C	FAM20C
16341	PKNOX2	2.263	0.548	1.384	0.300	0.409	0.356	PBX/knotted 1 homeobox 2	PKNOX2
19645	SLIT2	2.262	1.361	1.167	0.107	0.414	0.612	slit guidance ligand 2	SLIT2
8197	HIST2H2BF	2.262	1.013	1.397	0.277	0.979	0.544	histone cluster 2, H2bf	HIST2H2BF
15947	PDE1C	2.258	0.546	0.808	0.138	0.155	0.548	phosphodiesterase 1C	PDE1C
15623	PACERR	2.256	0.758	1.236	0.002	0.324	0.436	PTGS2 antisense NFKB1 complex-mediated expression regulator RNA	PACERR
3447	CFLAR	2.256	0.642	1.061	0.031	0.090	0.816	CASP8 and FADD like apoptosis regulator	CFLAR
4105	CREB3L2	2.253	0.949	1.312	0.039	0.797	0.233	cAMP responsive element binding protein 3-like 2	CREB3L2
8673	IFNGR2	2.251	0.755	1.435	0.104	0.366	0.092	interferon gamma receptor 2 (interferon gamma transducer 1)	IFNGR2
24212	ZNF697	2.251	0.883	1.251	0.157	0.691	0.344	zinc finger protein 697	ZNF697
13676	MPP4	2.248	0.960	1.000	0.162	0.895	0.999	membrane palmitoylated protein 4	MPP4
189	ACP2	2.248	1.079	1.655	0.138	0.805	0.332	acid phosphatase 2, lysosomal	ACP2
2089	C17orf58	2.246	0.602	1.136	0.041	0.155	0.171	chromosome 17 open reading frame 58	C17orf58
2463	C9orf16	2.245	0.610	1.207	0.405	0.545	0.476	chromosome 9 open reading frame 16	C9orf16
9301	KCNQ3	2.240	0.441	0.915	0.071	0.061	0.702	potassium voltage-gated channel subfamily Q member 3	KCNQ3
5477	EGLN3	2.236	0.394	0.946	0.373	0.338	0.684	egl-9 family hypoxia inducible factor 3	EGLN3
1662	BCOR	2.235	0.774	1.098	0.044	0.244	0.586	BCL6 corepressor	BCOR
5629	EML1	2.233	0.738	0.945	0.252	0.541	0.879	echinoderm microtubule associated protein like 1	EML1
17431	QSOX1	2.233	0.930	1.500	0.026	0.727	0.025	quiescin sulfhydryl oxidase 1	QSOX1
7219	GGT5	2.231	2.029	3.825	0.366	0.120	0.417	gamma-glutamyltransferase 5	GGT5
5058	DNAJB9	2.229	0.447	0.675	0.037	0.072	0.090	DnaJ heat shock protein family (Hsp40) member B9	DNAJB9
16123	PGM2L1	2.228	0.603	0.852	0.399	0.517	0.795	phosphoglucomutase 2-like 1	PGM2L1
17586	RAMP1	2.227	2.144	2.160	0.091	0.056	0.559	receptor activity modifying protein 1	RAMP1
1155	ARL4A	2.227	1.107	1.439	0.282	0.805	0.130	ADP ribosylation factor like GTPase 4A	ARL4A
13600	MMP8	2.226	0.786	0.952	0.009	0.066	0.321	matrix metallopeptidase 8	MMP8
22273	TRIM25	2.225	0.385	0.940	0.325	0.277	0.863	tripartite motif containing 25	TRIM25
9441	KIAA1644	2.223	1.057	2.246	0.025	0.869	0.383	KIAA1644	KIAA1644
19790	SNAI1	2.221	0.839	1.967	0.033	0.504	0.051	snail family zinc finger 1	SNAI1
4409	CXCR4	2.217	0.770	1.000	0.029	0.547	0.943	C-X-C motif chemokine receptor 4	CXCR4
4831	DGAT2	2.216	1.213	2.390	0.023	0.130	0.355	diacylglycerol O-acyltransferase 2	DGAT2
22150	TPI1	2.212	0.959	1.914	0.224	0.907	0.214	triosephosphate isomerase 1	TPI1
205	ACSL5	2.206	0.429	0.994	0.348	0.352	0.990	acyl-CoA synthetase long-chain family member 5	ACSL5
7613	GPR68	2.205	0.414	0.906	0.403	0.387	0.370	G protein-coupled receptor 68	GPR68
13653	MOV10	2.204	0.742	1.700	0.082	0.242	0.070	Mov10 RISC complex RNA helicase	MOV10
17218	PSME2	2.202	1.378	2.030	0.068	0.154	0.175	proteasome activator subunit 2	PSME2
8531	HTATIP2	2.199	0.769	1.256	0.265	0.610	0.132	HIV-1 Tat interactive protein 2	HTATIP2
20843	STX11	2.198	0.492	0.939	0.330	0.357	0.482	syntaxin 11	STX11
19281	SLC17A9	2.196	1.811	2.080	0.038	0.224	0.135	solute carrier family 17 member 9	SLC17A9
3322	CEND1	2.193	1.654	1.899	0.070	0.509	0.171	cell cycle exit and neuronal differentiation 1	CEND1
849	ANO10	2.193	0.782	1.213	0.247	0.563	0.560	anoctamin 10	ANO10
13606	MN1	2.191	0.372	0.806	0.050	0.044	0.356	meningioma (disrupted in balanced translocation) 1	MN1
14615	NNMT	2.188	0.997	2.956	0.168	0.990	0.441	nicotinamide N-methyltransferase	NNMT
17193	PSMB9	2.187	0.800	1.571	0.318	0.660	0.203	proteasome subunit beta 9	PSMB9
15049	OLMALINC	2.185	0.215	0.454	0.446	0.302	0.121	oligodendrocyte maturation-associated long intergenic non-coding R	OLMALINC
17625	RARRES2	2.184	0.067	0.227	0.622	0.382	0.498	retinoic acid receptor responder 2	RARRES2
21589	TLE1	2.183	0.573	0.831	0.042	0.066	0.207	transducin like enhancer of split 1	TLE1
995	APOO	2.181	1.369	1.429	0.305	0.498	0.205	apolipoprotein O	APOO
10036	LGR4	2.178	0.549	1.058	0.083	0.053	0.913	leucine-rich repeat containing G protein-coupled receptor 4	LGR4
811	ANKRD36B	2.176	0.336	0.688	0.263	0.199	0.090	ankyrin repeat domain 36B	ANKRD36B
19222	SLAMF7	2.176	0.475	1.000	0.311	0.322	0.943	SLAM family member 7	SLAMF7
18867	SEMA6A	2.173	1.022	0.977	0.018	0.964	0.631	semaphorin 6A	SEMA6A
22835	UGCG	2.170	0.567	1.005	0.065	0.096	0.988	UDP-glucose ceramide glucosyltransferase	UGCG
18534	S100A3	2.170	0.966	2.757	0.246	0.942	0.162	S100 calcium binding protein A3	S100A3
19581	SLC7A1	2.170	0.850	1.318	0.252	0.693	0.411	solute carrier family 7 member 1	SLC7A1
5617	EMC7	2.168	0.984	1.039	0.007	0.917	0.790	ER membrane protein complex subunit 7	EMC7
11242	LOC10041958	2.168	0.479	0.826	0.269	0.280	0.548	ring finger protein 4 pseudogene	LOC100419583
17192	PSMB8-AS1	2.167	0.623	1.148	0.315	0.451	0.145	PSMB8 antisense RNA 1 (head to head)	PSMB8-AS1
3623	CKAP4	2.165	1.272	1.669	0.021	0.086	0.130	cytoskeleton-associated protein 4	CKAP4
21603	TLR3	2.163	0.362	0.950	0.184	0.136	0.921	toll like receptor 3	TLR3
18862	SEMA4D	2.163	0.642	0.968	0.057	0.239	0.924	semaphorin 4D	SEMA4D
3654	CLDN1	2.159	0.629	0.928	0.082	0.143	0.719	claudin 1	CLDN1
1129	ARID3A	2.157	1.067	1.352	0.009	0.248	0.488	AT-rich interaction domain 3A	ARID3A

20405	SPATA25	2.156	0.483	0.738	0.263	0.263	0.311	spermatogenesis associated 25	SPATA25
9260	KCNJ2	2.156	0.907	0.842	0.460	0.884	0.734	potassium voltage-gated channel subfamily J member 2	KCNJ2
23241	WARS	2.155	0.437	1.124	0.384	0.365	0.736	tryptophanyl-tRNA synthetase	WARS
22644	TVP23B	2.155	0.744	1.092	0.049	0.229	0.275	trans-golgi network vesicle protein 23 homolog B (S. cerevisiae)	TVP23B
264	ACYP1	2.155	1.932	1.816	0.018	0.103	0.462	acylphosphatase 1	ACYP1
14469	NFKBIE	2.155	0.607	1.129	0.327	0.446	0.473	NFKB inhibitor epsilon	NFKBIE
15525	OSTM1	2.151	0.852	0.728	0.099	0.478	0.375	osteopetrosis associated transmembrane protein 1	OSTM1
17664	RASSF5	2.151	0.391	1.006	0.287	0.248	0.985	Ras association domain family member 5	RASSF5
10508	LINC00936	2.150	0.599	0.978	0.249	0.342	0.935	long intergenic non-protein coding RNA 936	LINC00936
19511	SLC41A2	2.149	0.368	0.777	0.263	0.210	0.395	solute carrier family 41 member 2	SLC41A2
18090	RNF145	2.148	0.668	0.898	0.055	0.127	0.579	ring finger protein 145	RNF145
858	ANOS1	2.148	0.523	0.620	0.572	0.620	0.454	anosmin 1	ANOS1
11061	LOC10010114	2.145	0.715	1.244	0.020	0.388	0.422	FK506 binding protein 6, 36kDa pseudogene	LOC100101148
7044	GALNT2	2.142	0.961	1.863	0.176	0.893	0.417	polypeptide N-acetylgalactosaminyltransferase 2	GALNT2
8809	IL21R-AS1	2.142	0.623	1.039	0.455	0.579	0.872	IL21R antisense RNA 1	IL21R-AS1
12612	LPAR1	2.141	0.844	1.496	0.126	0.552	0.061	lysophosphatidic acid receptor 1	LPAR1
14509	NIM1K	2.139	0.608	1.332	0.008	0.080	0.146	NIM1 serine/threonine protein kinase	NIM1K
18219	RORA	2.138	0.331	0.761	0.022	0.006	0.249	RAR related orphan receptor A	RORA
22439	TSPAN1	2.138	0.676	1.203	0.271	0.488	0.573	tetraspanin 1	TSPAN1
19702	SMC2	2.132	1.396	0.965	0.326	0.405	0.941	structural maintenance of chromosomes 2	SMC2
7701	GRK5	2.132	0.825	1.308	0.395	0.762	0.123	G protein-coupled receptor kinase 5	GRK5
12607	LOXL2	2.131	0.444	1.062	0.598	0.566	0.943	lysyl oxidase like 2	LOXL2
17765	RBPMS-AS1	2.130	0.735	1.327	0.051	0.230	0.190	RBPMS antisense RNA 1	RBPMS-AS1
6257	FAM65B	2.129	1.845	3.131	0.222	0.562	0.466	family with sequence similarity 65 member B	FAM65B
8710	IGF2R	2.128	0.515	0.685	0.050	0.029	0.271	insulin like growth factor 2 receptor	IGF2R
5627	EMILIN2	2.128	1.080	1.757	0.309	0.903	0.351	elastin microfibril interfacer 2	EMILIN2
16971	PRKCH	2.126	1.375	1.285	0.018	0.113	0.439	protein kinase C eta	PRKCH
13491	MIR210HG	2.126	0.217	0.495	0.266	0.142	0.228	MIR210 host gene	MIR210HG
21810	TMEM217	2.122	0.472	1.066	0.154	0.159	0.767	transmembrane protein 217	TMEM217
21745	TMEM165	2.122	0.720	0.866	0.027	0.127	0.529	transmembrane protein 165	TMEM165
12755	LRRC8D	2.121	0.911	1.085	0.147	0.729	0.671	leucine-rich repeat containing 8 family member D	LRRC8D
10123	LINC00160	2.119	1.539	1.312	0.288	0.527	0.422	long intergenic non-protein coding RNA 160	LINC00160
3779	CMC1	2.117	1.237	1.293	0.101	0.296	0.569	C-x9-C motif containing 1	CMC1
18871	SEMA6D	2.116	0.393	0.737	0.183	0.148	0.065	semaphorin 6D	SEMA6D
1904	BTN3A1	2.115	0.518	1.011	0.270	0.288	0.965	butyrophilin subfamily 3 member A1	BTN3A1
16269	PIM1	2.114	0.567	1.445	0.018	0.048	0.258	Pim-1 proto-oncogene, serine/threonine kinase	PIM1
20565	SPX	2.112	0.380	1.306	0.368	0.303	0.564	spexin hormone	SPX
1790	BOC	2.110	0.743	1.305	0.387	0.649	0.717	BOC cell adhesion associated, oncogene regulated	BOC
21447	TGIF1	2.109	0.962	0.929	0.227	0.892	0.872	TGFB induced factor homeobox 1	TGIF1
891	AOC2	2.105	0.656	0.853	0.063	0.067	0.662	amine oxidase, copper containing 2	AOC2
2170	C1S	2.105	0.362	0.590	0.092	0.021	0.338	complement component 1, s subcomponent	C1S
21903	TMEM62	2.100	0.716	1.081	0.203	0.407	0.823	transmembrane protein 62	TMEM62
8109	HIF1A	2.100	1.748	2.317	0.321	0.260	0.119	hypoxia inducible factor 1 alpha subunit	HIF1A
19580	SLC6A9	2.097	0.577	1.113	0.065	0.154	0.633	solute carrier family 6 member 9	SLC6A9
18640	SBSN	2.097	0.579	1.295	0.339	0.378	0.622	suprabasin	SBSN
22243	TREML3P	2.096	0.649	0.914	0.378	0.555	0.590	triggering receptor expressed on myeloid cells like 3, pseudogene	TREML3P
13163	MBOAT2	2.095	0.890	1.466	0.372	0.832	0.179	membrane bound O-acyltransferase domain containing 2	MBOAT2
21881	TMEM45A	2.093	0.587	0.953	0.103	0.181	0.747	transmembrane protein 45A	TMEM45A
22013	TNFRSF18	2.089	0.666	0.798	0.144	0.229	0.511	tumor necrosis factor receptor superfamily member 18	TNFRSF18
17432	QSOX2	2.088	1.337	1.338	0.020	0.037	0.150	quiescin sulfhydryl oxidase 2	QSOX2
18329	RPN2	2.084	0.931	1.570	0.045	0.583	0.162	ribophorin II	RPN2
23621	ZC3H12C	2.082	0.519	0.717	0.327	0.329	0.495	zinc finger CCCH-type containing 12C	ZC3H12C
20540	SPRY2	2.082	0.663	0.535	0.131	0.149	0.342	sprouty RTK signaling antagonist 2	SPRY2
952	APLN	2.081	0.155	0.401	0.580	0.392	0.254	apelin	APLN
5686	ENTPD7	2.080	0.554	0.795	0.140	0.111	0.568	ectonucleoside triphosphate diphosphohydrolase 7	ENTPD7
23036	USP54	2.078	0.866	1.002	0.165	0.669	0.988	ubiquitin specific peptidase 54	USP54
3574	CHST2	2.077	0.360	0.980	0.540	0.456	0.966	carbohydrate sulfotransferase 2	CHST2
13479	MIR100HG	2.077	0.619	1.507	0.017	0.067	0.444	mir-100-let-7a-2 cluster host gene	MIR100HG
23276	WDR11	2.077	0.524	0.864	0.264	0.289	0.558	WD repeat domain 11	WDR11
8620	IDO1	2.074	0.451	1.047	0.359	0.336	0.680	indoleamine 2,3-dioxygenase 1	IDO1
19287	SLC19A2	2.070	0.760	0.881	0.129	0.328	0.671	solute carrier family 19 member 2	SLC19A2
355	ADD3	2.070	0.726	1.037	0.166	0.431	0.814	adducin 3	ADD3
8209	HIVEP3	2.066	1.071	1.148	0.078	0.757	0.231	human immunodeficiency virus type I enhancer binding protein 3	HIVEP3
5079	DNAJC3	2.059	0.926	0.856	0.023	0.639	0.246	DnaJ heat shock protein family (Hsp40) member C3	DNAJC3
19270	SLC16A7	2.058	0.689	0.905	0.129	0.186	0.811	solute carrier family 16 member 7	SLC16A7
19010	SFRP2	2.057	0.335	0.505	0.116	0.008	0.336	secreted frizzled-related protein 2	SFRP2
296	ADAMDEC1	2.057	0.561	1.000	0.413	0.474	0.943	ADAM-like, decysin 1	ADAMDEC1
5475	EGLN1	2.054	0.539	0.803	0.012	0.018	0.285	egl-9 family hypoxia inducible factor 1	EGLN1
3818	CNN1	2.051	1.321	0.804	0.464	0.637	0.631	calponin 1	CNN1
8808	IL21R	2.050	0.608	1.019	0.473	0.569	0.926	interleukin 21 receptor	IL21R
417	ADPGK	2.049	0.920	1.310	0.000	0.202	0.057	ADP-dependent glucokinase	ADPGK
9158	JUP	2.049	0.294	0.792	0.273	0.167	0.456	junction plakoglobin	JUP
5807	ERO1A	2.044	0.582	0.839	0.056	0.018	0.622	endoplasmic reticulum oxidoreductase alpha	ERO1A
13315	MESDC1	2.042	0.764	1.013	0.203	0.499	0.900	mesoderm development candidate 1	MESDC1
17641	RASEF	2.041	0.458	0.903	0.453	0.428	0.469	RAS and EF-hand domain containing	RASEF
19166	SIK3	2.041	0.839	1.207	0.007	0.168	0.536	SIK family kinase 3	SIK3
22744	UBE2L6	2.041	0.467	1.160	0.203	0.175	0.549	ubiquitin conjugating enzyme E2 L6	UBE2L6
4761	DEFB109P1	2.038	1.026	1.828	0.423	0.975	0.423	defensin beta 109 pseudogene 1	DEFB109P1
1673	BDKRB2	2.036	0.488	0.781	0.244	0.119	0.722	bradykinin receptor B2	BDKRB2
9896	LARGE	2.034	0.507	0.663	0.140	0.145	0.058	like-glycosyltransferase	LARGE
8669	IFNE	2.028	0.819	1.227	0.005	0.132	0.548	interferon, epsilon	IFNE
21660	TMED1	2.026	0.996	1.552	0.025	0.976	0.408	transmembrane p24 trafficking protein 1	TMED1
5597	ELOVL3	2.025	0.759	0.937	0.137	0.303	0.856	ELOVL fatty acid elongase 3	ELOVL3
5308	DUSP8	2.024	0.562	0.882	0.065	0.096	0.527	dual specificity phosphatase 8	DUSP8
5179	DPP4	2.024	0.622	0.535	0.583	0.665	0.520	dipeptidyl peptidase 4	DPP4
12652	LRIG1	2.023	0.721	0.925	0.077	0.199	0.877	leucine-rich repeats and immunoglobulin like domains 1	LRIG1
1710	BHLHE40	2.019	0.229	0.722	0.048	0.009	0.361	basic helix-loop-helix family member e40	BHLHE40
16382	PLAUR	2.016	1.173	1.493	0.003	0.410	0.331	plasminogen activator, urokinase receptor	PLAUR
3716	CLIC2	2.015	0.308	0.810	0.509	0.385	0.613	chloride intracellular channel 2	CLIC2
8454	HS6ST1	2.015	0.514	0.935	0.492	0.486	0.895	heparan sulfate 6-O-sulfotransferase 1	HS6ST1
8579	HYOU1	2.015	1.270	1.504	0.071	0.318	0.545	hypoxia up-regulated 1	HYOU1

8751	IKBKE	2.014	0.844	1.847	0.317	0.726	0.040	inhibitor of kappa light polypeptide gene enhancer in B-cells, kinase e	IKBKE
5773	ERCC6-PGBD3	2.011	0.546	0.768	0.074	0.027	0.522	.	ERCC6-PGBD3
18803	SEC11C	2.007	1.548	0.940	0.187	0.518	0.853	SEC11 homolog C, signal peptidase complex subunit	SEC11C
13169	MBTPS2	2.007	0.825	1.071	0.013	0.107	0.625	membrane bound transcription factor peptidase, site 2	MBTPS2
18937	SERPINA9	2.006	4.059	1.338	0.409	0.400	0.334	serpin family A member 9	SERPINA9
20105	SNORD17	2.005	1.418	1.414	0.102	0.738	0.667	small nucleolar RNA, C/D box 17	SNORD17
17424	QPRT	2.004	0.118	0.415	0.640	0.419	0.423	quinolinate phosphoribosyltransferase	QPRT
5765	ERC2	2.004	0.759	1.000	0.358	0.637	0.943	ELKS/RAB6-interacting/CAST family member 2	ERC2
12878	LYSMD2	2.003	0.797	1.131	0.119	0.505	0.735	LysM domain containing 2	LYSMD2
14704	NPIPA5	2.001	1.124	1.135	0.269	0.796	0.398	nuclear pore complex interacting protein family member A5	NPIPA5
4803	DENND5A	1.999	0.629	1.000	0.129	0.177	0.999	DENN domain containing 5A	DENND5A
13450	MID1IP1	1.998	0.481	0.923	0.367	0.333	0.824	MID1 interacting protein 1	MID1IP1
1224	ARSJ	1.998	0.731	1.108	0.075	0.202	0.844	arylsulfatase family member J	ARSJ
2950	CCL26	1.996	0.862	1.497	0.344	0.780	0.184	C-C motif chemokine ligand 26	CCL26
15695	PAPPA	1.996	0.817	1.137	0.503	0.798	0.922	pappalysin 1	PAPPA
18137	RNF24	1.991	0.706	1.402	0.006	0.023	0.446	ring finger protein 24	RNF24
21778	TMEM189-UB	1.990	0.554	1.000	0.000	0.013	0.943	TMEM189-UBE2V1 readthrough	TMEM189-UBE2V1
18399	RPSAP52	1.990	1.094	1.571	0.504	0.896	0.491	ribosomal protein SA pseudogene 52	RPSAP52
6843	FRMD4A	1.989	0.551	0.998	0.052	0.089	0.991	FERM domain containing 4A	FRMD4A
20543	SPRY4-IT1	1.989	0.946	1.885	0.062	0.823	0.378	SPRY4 intronic transcript 1	SPRY4-IT1
3090	CD55	1.985	1.067	0.753	0.202	0.822	0.565	CD55 molecule (Cromer blood group)	CD55
18218	ROR2	1.984	0.344	1.057	0.102	0.023	0.943	receptor tyrosine kinase like orphan receptor 2	ROR2
21533	TIFA	1.983	0.746	0.858	0.277	0.531	0.498	TRAF interacting protein with forkhead associated domain	TIFA
1732	BISPR	1.983	0.569	1.034	0.199	0.245	0.632	BST2 interferon stimulated positive regulator (non-protein coding)	BISPR
4817	DERL1	1.982	0.822	1.050	0.143	0.492	0.727	derlin 1	DERL1
19800	SNAPC1	1.979	1.545	2.055	0.159	0.437	0.403	small nuclear RNA activating complex polypeptide 1	SNAPC1
8817	IL26	1.978	1.058	2.525	0.197	0.940	0.499	interleukin 26	IL26
22200	TRAF3	1.976	0.773	1.114	0.056	0.072	0.657	TNF receptor associated factor 3	TRAF3
14566	NLGN4Y	1.975	0.539	0.922	0.524	0.580	0.918	neuroligin 4, Y-linked	NLGN4Y
3238	CDKN2B	1.973	0.240	0.405	0.499	0.307	0.331	cyclin-dependent kinase inhibitor 2B	CDKN2B
8239	HLA-G	1.971	0.855	1.094	0.429	0.850	0.880	major histocompatibility complex, class I, G	HLA-G
5075	DNAJC25-GNG	1.971	0.507	1.000	0.118	0.118	0.943	DNAJC25-GNG10 readthrough	DNAJC25-GNG10
546	AK4	1.970	0.222	0.618	0.197	0.079	0.239	adenylate kinase 4	AK4
6959	GAB2	1.969	0.812	1.179	0.044	0.399	0.755	GRB2 associated binding protein 2	GAB2
13464	MILR1	1.969	1.680	2.682	0.324	0.222	0.266	mast cell immunoglobulin like receptor 1	MILR1
18821	SEC24A	1.968	0.633	0.932	0.063	0.104	0.816	SEC24 homolog A, COPII coat complex component	SEC24A
4570	DARS-AS1	1.966	0.419	0.942	0.033	0.030	0.722	DARS antisense RNA 1	DARS-AS1
7046	GALNT4	1.966	0.542	1.050	0.039	0.032	0.691	polypeptide N-acetylgalactosaminyltransferase 4	GALNT4
21411	TFB1M	1.965	0.799	1.147	0.202	0.573	0.267	transcription factor B1, mitochondrial	TFB1M
16001	PDPN	1.965	0.399	0.637	0.278	0.188	0.416	podoplanin	PDPN
22961	USP12	1.964	0.648	0.850	0.083	0.176	0.309	ubiquitin specific peptidase 12	USP12
21563	TIMP3	1.964	0.480	0.841	0.481	0.457	0.574	TIMP metallopeptidase inhibitor 3	TIMP3
7315	GLIS3	1.964	0.641	0.771	0.199	0.203	0.667	GLIS family zinc finger 3	GLIS3
5304	DUSP5	1.960	0.944	2.080	0.189	0.799	0.134	dual specificity phosphatase 5	DUSP5
15911	PCSK5	1.959	0.393	0.574	0.579	0.487	0.462	proprotein convertase subtilisin/kexin type 5	PCSK5
19179	SIRPB1	1.958	0.658	1.113	0.180	0.339	0.507	signal regulatory protein beta 1	SIRPB1
7212	GGH	1.957	3.094	4.622	0.053	0.121	0.145	gamma-glutamyl hydrolase	GGH
23645	ZCCHC2	1.957	0.492	0.747	0.132	0.119	0.130	zinc finger CCHC-type containing 2	ZCCHC2
7553	GPR135	1.956	0.712	1.009	0.191	0.320	0.976	G protein-coupled receptor 135	GPR135
2739	CBR3-AS1	1.956	0.764	1.353	0.057	0.284	0.128	CBR3 antisense RNA 1	CBR3-AS1
76	ABCC6	1.954	0.973	1.341	0.354	0.956	0.540	ATP binding cassette subfamily C member 6	ABCC6
1738	BLID	1.953	0.424	0.848	0.015	0.004	0.316	BH3-like motif containing, cell death inducer	BLID
18122	RNF213	1.951	0.440	0.938	0.334	0.274	0.829	ring finger protein 213	RNF213
9502	KIFC3	1.951	1.464	1.376	0.073	0.128	0.414	kinesin family member C3	KIFC3
1211	ARRDC2	1.950	0.989	1.230	0.022	0.973	0.723	arrestin domain containing 2	ARRDC2
23492	XYLT1	1.950	1.008	1.253	0.271	0.976	0.685	xylosyltransferase 1	XYLT1
17327	PTPRE	1.949	0.970	1.098	0.281	0.931	0.776	protein tyrosine phosphatase, receptor type E	PTPRE
24354	ZNFX1	1.945	0.443	0.724	0.225	0.182	0.234	zinc finger NFX1-type containing 1	ZNFX1
10027	LGALS9	1.944	0.586	1.006	0.245	0.314	0.969	lectin, galactoside binding soluble 9	LGALS9
15988	PDK1	1.943	0.240	0.638	0.198	0.097	0.143	pyruvate dehydrogenase kinase 1	PDK1
458	AGAP1	1.942	0.536	0.945	0.296	0.311	0.521	ArfGAP with GTPase domain, ankyrin repeat and PH domain 1	AGAP1
20775	STEAP3	1.940	0.337	0.784	0.467	0.336	0.575	STEAP3 metalloreductase	STEAP3
11019	LMAN1	1.937	0.826	1.133	0.004	0.166	0.191	lectin, mannose binding 1	LMAN1
16254	PIK3CD	1.937	0.609	0.994	0.263	0.341	0.965	phosphatidylinositol-4,5-bisphosphate 3-kinase catalytic subunit delt	PIK3CD
15795	PCBP3	1.936	0.869	1.956	0.112	0.544	0.312	poly(rC) binding protein 3	PCBP3
18577	SAMD11	1.935	2.514	6.935	0.106	0.404	0.422	sterile alpha motif domain containing 11	SAMD11
9093	ITPR1	1.934	0.527	1.012	0.314	0.287	0.986	inositol 1,4,5-trisphosphate receptor type 1	ITPR1
9113	JADE2	1.934	0.591	0.951	0.160	0.215	0.743	jade family PHD finger 2	JADE2
4042	CPED1	1.933	0.410	0.581	0.431	0.336	0.389	cadherin like and PC-esterase domain containing 1	CPED1
19257	SLC16A1	1.932	0.656	0.910	0.168	0.307	0.700	solute carrier family 16 member 1	SLC16A1
24376	ZRANB1	1.932	0.834	1.106	0.345	0.716	0.782	zinc finger RANBP2-type containing 1	ZRANB1
1905	BTN3A2	1.929	0.835	1.315	0.173	0.332	0.684	butyrophilin subfamily 3 member A2	BTN3A2
9988	LEF1	1.928	0.868	1.336	0.280	0.803	0.594	lymphoid enhancer binding factor 1	LEF1
14126	N4BP1	1.928	0.416	0.738	0.121	0.091	0.056	NEDD4 binding protein 1	N4BP1
21777	TMEM189	1.926	0.766	1.010	0.068	0.215	0.915	transmembrane protein 189	TMEM189
3101	CD74	1.926	2.423	1.060	0.213	0.568	0.922	CD74 molecule	CD74
14511	NINJ1	1.926	0.717	0.973	0.474	0.658	0.778	ninjurin 1	NINJ1
21404	TFAP2A	1.924	0.689	0.917	0.067	0.221	0.708	transcription factor AP-2 alpha	TFAP2A
18550	S1PR3	1.923	1.964	1.975	0.176	0.054	0.247	sphingosine-1-phosphate receptor 3	S1PR3
6367	FAS	1.923	1.092	1.045	0.377	0.857	0.946	Fas cell surface death receptor	FAS
18661	SCARB2	1.922	0.611	0.835	0.045	0.035	0.402	scavenger receptor class B, member 2	SCARB2
22127	TP53INP2	1.920	0.390	0.670	0.036	0.026	0.113	tumor protein p53 inducible nuclear protein 2	TP53INP2
6907	FURIN	1.920	1.183	1.918	0.013	0.586	0.371	furin, paired basic amino acid cleaving enzyme	FURIN
20612	SRPX2	1.920	1.165	1.401	0.296	0.671	0.685	sushi repeat containing protein, X-linked 2	SRPX2
9538	KLF10	1.920	1.235	1.880	0.140	0.543	0.098	Kruppel-like factor 10	KLF10
17667	RASSF8	1.919	1.014	1.099	0.085	0.967	0.849	Ras association domain family member 8	RASSF8
14392	NEGR1	1.917	1.311	1.361	0.400	0.570	0.678	neuronal growth regulator 1	NEGR1
21718	TMEM140	1.915	0.349	0.733	0.422	0.296	0.341	transmembrane protein 140	TMEM140
20665	SSTR2	1.915	0.541	0.979	0.127	0.137	0.889	somatostatin receptor 2	SSTR2
13689	MPZL1	1.914	0.671	1.037	0.172	0.286	0.782	myelin protein zero like 1	MPZL1
8568	HYAL1	1.913	0.453	1.154	0.008	0.006	0.589	hyaluronoglucosaminidase 1	HYAL1

12823	LUM	1.913	0.391	0.668	0.019	0.022	0.008	lumican	LUM
113	ABHD5	1.911	1.220	1.381	0.055	0.587	0.314	abhydrolase domain containing 5	ABHD5
22154	TPM1	1.911	1.563	1.458	0.284	0.309	0.256	tropomyosin 1 (alpha)	TPM1
10087	LIMK1	1.911	1.020	1.302	0.317	0.961	0.089	LIM domain kinase 1	LIMK1
13188	MCHR1	1.910	0.473	1.168	0.007	0.003	0.646	melanin concentrating hormone receptor 1	MCHR1
17951	RHOB	1.910	0.819	2.049	0.535	0.810	0.277	ras homolog family member B	RHOB
66	ABCC1	1.910	0.533	1.006	0.221	0.229	0.973	ATP binding cassette subfamily C member 1	ABCC1
5421	EEF1E1	1.909	1.348	1.681	0.186	0.291	0.054	eukaryotic translation elongation factor 1 epsilon 1	EEF1E1
5656	ENO1	1.909	1.227	2.600	0.046	0.249	0.052	enolase 1	ENO1
3735	CLMP	1.907	0.626	0.822	0.247	0.233	0.705	CXADR-like membrane protein	CLMP
443	AFAP1	1.906	0.530	0.921	0.024	0.005	0.710	actin filament associated protein 1	AFAP1
1652	BCL6	1.905	0.366	1.317	0.085	0.044	0.467	B-cell CLL/lymphoma 6	BCL6
13899	MTF1	1.905	0.675	1.023	0.218	0.343	0.893	metal-regulatory transcription factor 1	MTF1
19267	SLC16A4	1.904	0.695	1.333	0.016	0.033	0.100	solute carrier family 16 member 4	SLC16A4
5772	ERCC6	1.902	0.489	0.636	0.115	0.110	0.192	excision repair cross-complementation group 6	ERCC6
5798	ERLEC1	1.902	0.903	1.030	0.005	0.422	0.660	endoplasmic reticulum lectin 1	ERLEC1
17303	PTPN1	1.900	0.829	1.199	0.143	0.484	0.272	protein tyrosine phosphatase, non-receptor type 1	PTPN1
18068	RNF114	1.898	0.568	0.946	0.255	0.288	0.577	ring finger protein 114	RNF114
13288	MEGF10	1.898	0.718	1.278	0.189	0.469	0.508	multiple EGF like domains 10	MEGF10
22839	UGGT2	1.897	0.844	0.941	0.039	0.468	0.501	UDP-glucose glycoprotein glucosyltransferase 2	UGGT2
14456	NFE2L3	1.896	0.452	0.874	0.067	0.052	0.602	nuclear factor, erythroid 2 like 3	NFE2L3
6310	FAM86DP	1.894	0.976	1.363	0.084	0.922	0.113	family with sequence similarity 86 member D, pseudogene	FAM86DP
13022	MAP2K1	1.893	0.611	0.824	0.030	0.030	0.569	mitogen-activated protein kinase kinase 1	MAP2K1
7370	GMNN	1.892	4.171	4.469	0.055	0.066	0.299	geminin, DNA replication inhibitor	GMNN
20343	SP100	1.892	0.377	0.884	0.244	0.156	0.537	SP100 nuclear antigen	SP100
20286	SOCS1	1.892	1.076	1.623	0.037	0.629	0.073	suppressor of cytokine signaling 1	SOCS1
21008	SYS1-DBNDD2	1.892	0.934	1.837	0.121	0.798	0.283	SYS1-DBNDD2 readthrough (NMD candidate)	SYS1-DBNDD2
17354	PTX3	1.892	0.331	0.672	0.239	0.100	0.583	pentraxin 3	PTX3
1532	B3GNT7	1.891	0.724	0.881	0.490	0.681	0.030	UDP-GlcNAc:betaGal beta-1,3-N-acetylglucosaminyltransferase 7	B3GNT7
5662	ENOSF1	1.890	1.105	1.380	0.086	0.671	0.070	enolase superfamily member 1	ENOSF1
365	ADGRD1	1.884	0.233	0.443	0.630	0.426	0.438	adhesion G protein-coupled receptor D1	ADGRD1
7330	GLRX2	1.883	1.136	1.357	0.240	0.728	0.375	glutaredoxin 2	GLRX2
8672	IFNGR1	1.883	0.642	0.863	0.005	0.007	0.456	interferon gamma receptor 1	IFNGR1
18690	SCD	1.882	0.025	0.108	0.698	0.399	0.446	stearoyl-CoA desaturase (delta-9-desaturase)	SCD
5910	EXT2	1.881	0.876	1.249	0.042	0.293	0.154	exostosin glycosyltransferase 2	EXT2
22217	TRANK1	1.881	0.592	0.835	0.002	0.004	0.019	tetratricopeptide repeat and ankyrin repeat containing 1	TRANK1
5245	DSE	1.880	0.739	1.134	0.100	0.356	0.730	dermatan sulfate epimerase	DSE
16956	PRKAG2	1.880	1.150	1.349	0.048	0.348	0.174	protein kinase AMP-activated non-catalytic subunit gamma 2	PRKAG2
15777	PBXIP1	1.878	0.345	0.864	0.092	0.046	0.775	pre-B-cell leukemia homeobox interacting protein 1	PBXIP1
2167	C1R	1.878	0.378	0.768	0.232	0.108	0.587	complement C1r subcomponent	C1R
12025	LOC10192970	1.877	0.496	0.833	0.142	0.113	0.326	uncharacterized LOC101929709	LOC101929709
22375	TRPA1	1.877	0.579	1.012	0.227	0.262	0.682	transient receptor potential cation channel subfamily A member 1	TRPA1
894	AOX1	1.876	0.830	1.678	0.361	0.668	0.570	aldehyde oxidase 1	AOX1
23439	XBP1	1.873	0.720	1.037	0.102	0.271	0.820	X-box binding protein 1	XBP1
16042	PELO	1.872	0.790	0.908	0.301	0.605	0.620	pelota homolog (Drosophila)	PELO
18225	ROS1	1.869	0.893	0.937	0.005	0.009	0.417	ROS proto-oncogene 1, receptor tyrosine kinase	ROS1
17263	PTGDS	1.869	0.190	0.283	0.545	0.292	0.328	prostaglandin D2 synthase	PTGDS
23139	VLDLR	1.868	0.580	0.864	0.067	0.060	0.588	very low density lipoprotein receptor	VLDLR
23515	YIPF6	1.867	0.734	0.909	0.042	0.118	0.200	Yip1 domain family member 6	YIPF6
21229	TBX4	1.867	0.603	1.165	0.543	0.614	0.856	T-box 4	TBX4
124	ABLIM3	1.864	0.781	1.037	0.525	0.747	0.952	actin binding LIM protein family member 3	ABLIM3
17299	PTP4A2	1.862	0.707	1.400	0.108	0.217	0.095	protein tyrosine phosphatase type IVA, member 2	PTP4A2
13042	MAP3K5	1.862	1.313	1.159	0.199	0.585	0.514	mitogen-activated protein kinase kinase kinase 5	MAP3K5
17828	RELT	1.861	1.165	1.175	0.032	0.312	0.286	RELT tumor necrosis factor receptor	RELT
15697	PAPPA2	1.861	0.499	1.009	0.013	0.007	0.937	pappalysin 2	PAPPA2
16701	PPAN-P2RY11	1.860	1.170	1.622	0.421	0.848	0.422	PPAN-P2RY11 readthrough	PPAN-P2RY11
17183	PSMB10	1.860	0.731	1.669	0.180	0.320	0.157	proteasome subunit beta 10	PSMB10
16504	PMEPA1	1.858	1.295	1.578	0.305	0.597	0.495	prostate transmembrane protein, androgen induced 1	PMEPA1
14641	NOMO1	1.856	1.160	1.703	0.003	0.036	0.205	NODAL modulator 1	NOMO1
6947	FZD9	1.855	0.620	0.919	0.115	0.197	0.201	frizzled class receptor 9	FZD9
16833	PPP3CC	1.854	0.791	1.100	0.126	0.369	0.499	protein phosphatase 3 catalytic subunit gamma	PPP3CC
16234	PIGP	1.854	0.797	0.860	0.203	0.482	0.808	phosphatidylinositol glycan anchor biosynthesis class P	PIGP
73	ABCC4	1.853	0.656	1.100	0.214	0.285	0.756	ATP binding cassette subfamily C member 4	ABCC4
8207	HIVEP1	1.852	0.460	0.765	0.198	0.151	0.276	human immunodeficiency virus type I enhancer binding protein 1	HIVEP1
21205	TBK1	1.852	0.639	0.887	0.213	0.290	0.419	TANK binding kinase 1	TBK1
401	ADIPOR1	1.852	0.901	0.914	0.188	0.705	0.816	adiponectin receptor 1	ADIPOR1
7661	GRB14	1.851	1.175	1.167	0.258	0.683	0.233	growth factor receptor bound protein 14	GRB14
16557	PNRC1	1.849	0.315	0.886	0.261	0.141	0.477	proline rich nuclear receptor coactivator 1	PNRC1
203	ACSL3	1.848	0.316	0.540	0.582	0.386	0.546	acyl-CoA synthetase long-chain family member 3	ACSL3
2052	C15orf65	1.848	0.748	0.972	0.036	0.095	0.826	chromosome 15 open reading frame 65	C15orf65
2364	C5orf56	1.845	0.547	0.888	0.267	0.239	0.783	chromosome 5 open reading frame 56	C5orf56
19468	SLC35G2	1.845	1.057	0.961	0.223	0.802	0.931	solute carrier family 35 member G2	SLC35G2
19392	SLC26A6	1.844	0.861	1.576	0.083	0.188	0.550	solute carrier family 26 member 6	SLC26A6
20344	SP110	1.842	0.427	1.069	0.249	0.181	0.778	SP110 nuclear body protein	SP110
1715	BHMT2	1.841	0.613	0.902	0.336	0.370	0.844	betaine--homocysteine S-methyltransferase 2	BHMT2
19947	SNORA74A	1.840	0.543	1.000	0.423	0.423	0.943	small nucleolar RNA, H/ACA box 74A	SNORA74A
866	ANPEP	1.838	1.795	2.921	0.231	0.104	0.015	alanyl aminopeptidase, membrane	ANPEP
23581	ZBTB21	1.838	0.621	0.825	0.119	0.138	0.434	zinc finger and BTB domain containing 21	ZBTB21
16278	PINLYP	1.837	1.201	1.694	0.384	0.663	0.117	phospholipase A2 inhibitor and LY6/PLAUR domain containing	PINLYP
8206	HIST4H4	1.836	0.453	0.819	0.198	0.124	0.507	histone cluster 4, H4	HIST4H4
5039	DNAJA1	1.836	0.823	0.986	0.127	0.492	0.936	DnaJ heat shock protein family (Hsp40) member A1	DNAJA1
18819	SEC23B	1.834	0.945	1.260	0.376	0.907	0.226	Sec23 homolog B, coat complex II component	SEC23B
6436	FBXO32	1.832	0.583	0.485	0.308	0.371	0.307	F-box protein 32	FBXO32
6763	FOXC1	1.830	0.789	1.172	0.008	0.007	0.363	forkhead box C1	FOXC1
23684	ZEB2	1.829	0.442	0.876	0.301	0.225	0.685	zinc finger E-box binding homeobox 2	ZEB2
6000	FAM117A	1.828	0.356	0.755	0.441	0.305	0.204	family with sequence similarity 117 member A	FAM117A
17728	RBM47	1.825	0.745	1.225	0.308	0.530	0.226	RNA binding motif protein 47	RBM47
988	APOL2	1.825	0.597	1.193	0.202	0.193	0.500	apolipoprotein L2	APOL2
6334	FAM96B	1.824	1.334	1.583	0.015	0.237	0.138	family with sequence similarity 96 member B	FAM96B
2358	C5orf45	1.824	0.876	1.262	0.368	0.801	0.424	chromosome 5 open reading frame 45	C5orf45
17110	PRRX2	1.823	1.377	2.902	0.230	0.329	0.405	paired related homeobox 2	PRRX2

18504	RUSC1-AS1	1.823	0.394	0.589	0.607	0.496	0.285	RUSC1 antisense RNA 1	RUSC1-AS1
11849	LOC10192879	1.823	1.159	2.089	0.081	0.423	0.057	uncharacterized LOC101928796	LOC101928796
22028	TNFSF13B	1.821	0.448	0.856	0.247	0.133	0.689	tumor necrosis factor superfamily member 13b	TNFSF13B
13659	MPC2	1.820	0.813	1.131	0.193	0.506	0.263	mitochondrial pyruvate carrier 2	MPC2
9231	KCNE5	1.816	1.251	1.129	0.127	0.385	0.222	potassium voltage-gated channel subfamily E regulatory subunit 5	KCNE5
20365	SPAG1	1.815	0.928	0.950	0.292	0.849	0.135	sperm associated antigen 1	SPAG1
2558	CACUL1	1.815	0.641	0.956	0.334	0.413	0.819	CDK2 associated cullin domain 1	CACUL1
14874	NUB1	1.814	0.610	1.011	0.216	0.262	0.573	negative regulator of ubiquitin-like proteins 1	NUB1
15979	PDHX	1.814	0.799	1.268	0.122	0.425	0.234	pyruvate dehydrogenase complex component X	PDHX
5001	DMKN	1.814	0.279	0.623	0.303	0.156	0.309	dermokine	DMKN
2143	C1GALT1	1.812	0.558	1.109	0.271	0.275	0.350	core 1 synthase, glycoprotein-N-acetylgalactosamine 3-beta-galactos	C1GALT1
9	A4GALT	1.811	0.869	1.475	0.171	0.344	0.515	alpha 1,4-galactosyltransferase	A4GALT
992	APOL6	1.811	0.468	0.995	0.239	0.083	0.992	apolipoprotein L6	APOL6
6963	GABARAPL1	1.808	0.935	1.403	0.036	0.729	0.702	GABA type A receptor associated protein like 1	GABARAPL1
22394	TRPS1	1.807	0.510	0.916	0.104	0.046	0.722	transcriptional repressor GATA binding 1	TRPS1
7329	GLRX	1.807	0.761	1.380	0.106	0.272	0.417	glutaredoxin (thioltransferase)	GLRX
493	AGPAT4-IT1	1.806	0.806	1.514	0.235	0.568	0.570	AGPAT4 intronic transcript 1	AGPAT4-IT1
8406	HPD	1.805	1.413	2.227	0.588	0.704	0.461	4-hydroxyphenylpyruvate dioxygenase	HPD
16508	PML	1.805	0.497	1.027	0.325	0.284	0.425	promyelocytic leukemia	PML
13804	MRPS6	1.803	0.824	1.178	0.088	0.358	0.174	mitochondrial ribosomal protein S6	MRPS6
5059	DNAJC1	1.802	1.186	1.190	0.002	0.532	0.673	DnaJ heat shock protein family (Hsp40) member C1	DNAJC1
20931	SUSD1	1.802	0.820	1.133	0.025	0.142	0.566	sushi domain containing 1	SUSD1
8116	HIGD1C	1.802	0.555	1.000	0.423	0.423	0.943	HIG1 hypoxia inducible domain family member 1C	HIGD1C
22901	UNC93B1	1.800	0.471	0.866	0.120	0.087	0.201	unc-93 homolog B1 (C. elegans)	UNC93B1
16262	PIK3R3	1.800	0.621	0.958	0.438	0.513	0.927	phosphoinositide-3-kinase regulatory subunit 3	PIK3R3
13453	MIDN	1.798	0.896	1.665	0.153	0.647	0.226	midnolin	MIDN
6098	FAM177A1	1.798	0.789	0.937	0.039	0.075	0.652	family with sequence similarity 177 member A1	FAM177A1
13869	MT1A	1.797	2.368	4.038	0.346	0.327	0.149	metallothionein 1A	MT1A
5511	EIF2AK3	1.796	0.509	0.743	0.095	0.071	0.205	eukaryotic translation initiation factor 2 alpha kinase 3	EIF2AK3
1906	BTN3A3	1.795	0.467	0.773	0.316	0.221	0.464	butyrophilin subfamily 3 member A3	BTN3A3
7141	GCLM	1.794	0.357	0.758	0.370	0.254	0.546	glutamate-cysteine ligase modifier subunit	GCLM
23887	ZNF267	1.794	0.885	0.795	0.071	0.612	0.307	zinc finger protein 267	ZNF267
4949	DLAT	1.793	0.853	0.981	0.176	0.547	0.950	dihydrolipoamide S-acetyltransferase	DLAT
529	AIFM2	1.792	0.913	0.934	0.085	0.596	0.760	apoptosis inducing factor, mitochondria associated 2	AIFM2
12921	MAFF	1.791	0.674	0.566	0.254	0.239	0.375	v-maf avian musculoaponeurotic fibrosarcoma oncogene homolog F	MAFF
5473	EGFR	1.791	0.618	1.162	0.116	0.054	0.679	epidermal growth factor receptor	EGFR
8863	IMPAD1	1.790	0.998	1.167	0.070	0.993	0.218	inositol monophosphatase domain containing 1	IMPAD1
23130	VIMP	1.789	0.990	1.172	0.008	0.890	0.376	VCP interacting membrane selenoprotein	VIMP
2396	C6orf62	1.789	0.789	1.336	0.018	0.172	0.262	chromosome 6 open reading frame 62	C6orf62
8939	IP6K3	1.788	0.565	0.946	0.388	0.392	0.424	inositol hexakisphosphate kinase 3	IP6K3
5937	F3	1.788	0.770	2.095	0.197	0.546	0.193	coagulation factor III, tissue factor	F3
9104	IVNS1ABP	1.788	1.518	1.279	0.271	0.392	0.314	influenza virus NS1A binding protein	IVNS1ABP
20404	SPATA24	1.787	0.924	1.287	0.005	0.148	0.425	spermatogenesis associated 24	SPATA24
8194	HIST2H2BA	1.787	1.147	0.843	0.557	0.838	0.697	histone cluster 2, H2ba (pseudogene)	HIST2H2BA
23247	WASF3	1.787	0.440	0.640	0.107	0.036	0.268	WAS protein family member 3	WASF3
20306	SORCS2	1.786	0.570	1.017	0.303	0.307	0.941	sortilin related VPS10 domain containing receptor 2	SORCS2
9119	JAK2	1.786	0.565	1.043	0.155	0.140	0.854	Janus kinase 2	JAK2
15985	PDIA6	1.786	1.139	1.464	0.139	0.519	0.278	protein disulfide isomerase family A member 6	PDIA6
5909	EXT1	1.786	0.876	1.118	0.050	0.151	0.784	exostosin glycosyltransferase 1	EXT1
8772	IL15	1.786	0.790	1.162	0.021	0.073	0.195	interleukin 15	IL15
5052	DNAJB5	1.785	0.629	0.928	0.306	0.332	0.819	DnaJ heat shock protein family (Hsp40) member B5	DNAJB5
16077	PFDN2	1.784	1.451	1.757	0.007	0.041	0.123	prefoldin subunit 2	PFDN2
4455	CYLD	1.784	0.542	0.802	0.264	0.229	0.505	CYLD lysine 63 deubiquitinase	CYLD
4821	DESI1	1.782	0.836	1.230	0.077	0.485	0.054	desumoylating isopeptidase 1	DESI1
4969	DLGAP1-AS2	1.782	0.783	0.966	0.137	0.353	0.900	DLGAP1 antisense RNA 2	DLGAP1-AS2
22285	TRIM36	1.779	0.637	0.987	0.301	0.371	0.418	tripartite motif containing 36	TRIM36
14733	NPTN	1.779	0.888	1.118	0.055	0.460	0.075	neuroplastin	NPTN
22450	TSPAN2	1.779	0.891	1.081	0.094	0.668	0.404	tetraspanin 2	TSPAN2
7051	GALNT9	1.779	0.774	1.254	0.461	0.686	0.212	polypeptide N-acetylgalactosaminyltransferase 9	GALNT9
20787	STK17A	1.777	1.454	1.332	0.059	0.172	0.257	serine/threonine kinase 17a	STK17A
20929	SURF4	1.776	1.610	1.816	0.011	0.093	0.223	surfeit 4	SURF4
15667	PALM2	1.776	1.159	1.161	0.005	0.139	0.548	paralemmin 2	PALM2
15981	PDIA3	1.776	0.927	1.415	0.292	0.834	0.460	protein disulfide isomerase family A member 3	PDIA3
7403	GNG11	1.775	2.720	2.655	0.378	0.172	0.408	G protein subunit gamma 11	GNG11
15720	PARM1	1.773	1.825	1.042	0.333	0.531	0.752	prostate androgen-regulated mucin-like protein 1	PARM1
13460	MIF	1.772	0.913	1.915	0.304	0.800	0.162	macrophage migration inhibitory factor (glycosylation-inhibiting facto	MIF
19417	SLC2A3	1.772	0.364	1.066	0.297	0.178	0.647	solute carrier family 2 member 3	SLC2A3
4113	CREG1	1.772	0.574	0.604	0.013	0.002	0.040	cellular repressor of E1A stimulated genes 1	CREG1
20466	SPESP1	1.770	0.782	1.006	0.411	0.709	0.986	sperm equatorial segment protein 1	SPESP1
20991	SYNJ1	1.770	0.618	0.905	0.081	0.195	0.780	synaptojanin 1	SYNJ1
569	AKIRIN1	1.767	0.810	1.004	0.141	0.437	0.989	akirin 1	AKIRIN1
15580	P2RX4	1.765	0.727	0.876	0.197	0.342	0.636	purinergic receptor P2X 4	P2RX4
7122	GBP5	1.764	0.684	0.999	0.228	0.339	0.956	guanylate binding protein 5	GBP5
6393	FBN2	1.764	1.548	3.203	0.561	0.584	0.132	fibrillin 2	FBN2
23734	ZFYVE16	1.764	0.565	0.693	0.322	0.297	0.325	zinc finger FYVE-type containing 16	ZFYVE16
6173	FAM219A	1.762	0.526	0.841	0.012	0.027	0.433	family with sequence similarity 219 member A	FAM219A
5437	EFCAB3	1.760	0.530	0.950	0.151	0.130	0.364	EF-hand calcium binding domain 3	EFCAB3
5409	EDNRA	1.760	0.888	1.199	0.163	0.734	0.010	endothelin receptor type A	EDNRA
172	ACO1	1.759	0.763	1.223	0.167	0.357	0.205	aconitase 1	ACO1
1355	ATL2	1.758	1.002	1.002	0.177	0.993	0.982	atlastin GTPase 2	ATL2
15699	PAPSS2	1.758	0.779	0.990	0.127	0.233	0.981	3'-phosphoadenosine 5'-phosphosulfate synthase 2	PAPSS2
3247	CDR1	1.757	0.874	1.507	0.566	0.877	0.591	cerebellar degeneration related protein 1	CDR1
7086	GAS7	1.757	0.484	1.215	0.446	0.375	0.368	growth arrest specific 7	GAS7
2108	C18orf32	1.756	0.911	1.051	0.001	0.495	0.605	chromosome 18 open reading frame 32	C18orf32
14302	NCSTN	1.756	0.657	0.806	0.017	0.010	0.319	nicastrin	NCSTN
6701	FLRT2	1.756	0.894	0.843	0.155	0.311	0.667	fibronectin leucine rich transmembrane protein 2	FLRT2
22840	UGP2	1.756	0.863	1.046	0.029	0.262	0.099	UDP-glucose pyrophosphorylase 2	UGP2
5964	FADS2	1.756	0.113	0.251	0.707	0.420	0.465	fatty acid desaturase 2	FADS2
1497	AVPI1	1.755	0.665	0.778	0.144	0.339	0.369	arginine vasopressin induced 1	AVPI1
14978	NXT2	1.754	0.934	1.196	0.281	0.840	0.575	nuclear transport factor 2 like export factor 2	NXT2
15596	P3H1	1.753	0.964	1.426	0.125	0.831	0.271	prolyl 3-hydroxylase 1	P3H1

18639	SBNO2	1.753	0.737	2.059	0.142	0.215	0.267	strawberry notch homolog 2 (Drosophila)	SBNO2
21667	TMED5	1.753	0.857	0.938	0.090	0.189	0.806	transmembrane p24 trafficking protein 5	TMED5
17391	PXDN	1.751	1.277	1.970	0.412	0.647	0.440	peroxidasin	PXDN
19246	SLC13A3	1.749	0.543	1.006	0.308	0.282	0.950	solute carrier family 13 member 3	SLC13A3
2347	C5AR1	1.749	0.468	0.667	0.515	0.400	0.483	complement component 5a receptor 1	C5AR1
682	AMD1	1.749	1.201	1.270	0.057	0.070	0.268	adenosylmethionine decarboxylase 1	AMD1
15605	P4HB	1.748	0.998	1.481	0.159	0.990	0.243	prolyl 4-hydroxylase subunit beta	P4HB
21228	TBX3	1.748	0.507	1.163	0.191	0.172	0.388	T-box 3	TBX3
12200	LOC10574897	1.745	0.735	1.860	0.371	0.582	0.422	uncharacterized LOC105748977	LOC105748977
23739	ZFYVE28	1.744	0.906	1.323	0.397	0.849	0.486	zinc finger FYVE-type containing 28	ZFYVE28
20745	STARD13	1.744	0.775	0.854	0.021	0.170	0.346	StAR related lipid transfer domain containing 13	STARD13
18735	SCN8A	1.742	0.792	0.862	0.036	0.071	0.425	sodium voltage-gated channel alpha subunit 8	SCN8A
9554	KLF9	1.742	0.929	2.032	0.081	0.604	0.346	Kruppel-like factor 9	KLF9
14572	NLRC5	1.742	0.575	0.936	0.290	0.298	0.470	NLR family, CARD domain containing 5	NLRC5
22441	TSPAN11	1.741	0.260	1.228	0.111	0.010	0.540	tetraspanin 11	TSPAN11
8613	IDH3A	1.741	1.238	1.266	0.019	0.160	0.198	isocitrate dehydrogenase 3 (NAD(+)) alpha	IDH3A
18831	SEC61G	1.740	1.193	1.556	0.027	0.403	0.069	Sec61 translocon gamma subunit	SEC61G
23208	VSTM4	1.739	0.536	0.838	0.130	0.125	0.131	V-set and transmembrane domain containing 4	VSTM4
7323	GLP2R	1.738	0.710	1.095	0.261	0.400	0.013	glucagon like peptide 2 receptor	GLP2R
17191	PSMB8	1.738	0.887	1.244	0.355	0.772	0.299	proteasome subunit beta 8	PSMB8
5358	EAF1	1.738	0.923	1.169	0.319	0.835	0.579	ELL associated factor 1	EAF1
6252	FAM60A	1.736	0.834	1.304	0.091	0.428	0.118	family with sequence similarity 60 member A	FAM60A
5658	ENO2	1.736	0.389	0.550	0.135	0.042	0.255	enolase 2	ENO2
20776	STEAP3-AS1	1.736	0.393	0.808	0.444	0.295	0.461	STEAP3 antisense RNA 1	STEAP3-AS1
16013	PDXP	1.735	1.426	1.188	0.062	0.092	0.240	pyridoxal phosphatase	PDXP
7114	GBE1	1.734	0.616	0.912	0.060	0.117	0.494	glucan (1,4-alpha-), branching enzyme 1	GBE1
11469	LOC10192696	1.733	1.141	1.192	0.141	0.437	0.490	uncharacterized LOC101926963	LOC101926963
16470	PLOD1	1.732	0.772	1.296	0.401	0.624	0.373	procollagen-lysine,2-oxoglutarate 5-dioxygenase 1	PLOD1
21170	TBC1D22B	1.732	0.682	1.207	0.186	0.275	0.383	TBC1 domain family member 22B	TBC1D22B
18202	ROBO1	1.731	0.630	0.945	0.061	0.072	0.828	roundabout guidance receptor 1	ROBO1
18845	SELM	1.731	1.073	1.566	0.242	0.816	0.415	selenoprotein M	SELM
9422	KIAA1217	1.731	0.644	0.649	0.033	0.093	0.123	KIAA1217	KIAA1217
7120	GBP3	1.729	0.372	0.763	0.685	0.532	0.817	guanylate binding protein 3	GBP3
9520	KIRREL	1.729	0.571	0.932	0.007	0.009	0.437	kin of IRRE like (Drosophila)	KIRREL
3476	CHCHD10	1.728	1.172	1.121	0.077	0.750	0.704	coiled-coil-helix-coiled-coil-helix domain containing 10	CHCHD10
18489	RTTN	1.728	1.508	0.969	0.292	0.229	0.900	rotatin	RTTN
3394	CERS5	1.728	0.955	1.244	0.089	0.775	0.526	ceramide synthase 5	CERS5
8689	IFT43	1.727	1.456	1.723	0.053	0.073	0.254	intraflagellar transport 43	IFT43
1753	BLZF1	1.727	0.658	0.900	0.178	0.202	0.753	basic leucine zipper nuclear factor 1	BLZF1
17946	RHEB	1.727	1.203	1.091	0.024	0.061	0.425	Ras homolog enriched in brain	RHEB
3977	COQ10B	1.725	0.757	0.908	0.248	0.433	0.547	coenzyme Q10B	COQ10B
814	ANKRD36C	1.724	0.372	0.682	0.368	0.216	0.258	ankyrin repeat domain 36C	ANKRD36C
14290	NCOA7	1.722	0.414	0.779	0.333	0.200	0.493	nuclear receptor coactivator 7	NCOA7
22745	UBE2M	1.721	1.447	1.509	0.058	0.235	0.123	ubiquitin conjugating enzyme E2 M	UBE2M
9552	KLF7	1.720	0.517	1.121	0.046	0.034	0.129	Kruppel-like factor 7 (ubiquitous)	KLF7
24370	ZP3	1.720	0.753	1.294	0.374	0.600	0.532	zona pellucida glycoprotein 3 (sperm receptor)	ZP3
5402	EDEM2	1.720	1.170	1.434	0.239	0.684	0.127	ER degradation enhancing alpha-mannosidase like protein 2	EDEM2
16457	PLGRKT	1.720	1.165	1.219	0.248	0.555	0.427	plasminogen receptor with a C-terminal lysine	PLGRKT
23375	WIPI1	1.720	0.587	1.050	0.210	0.094	0.901	WD repeat domain, phosphoinositide interacting 1	WIPI1
7674	GRIA3	1.720	1.120	1.177	0.001	0.491	0.387	glutamate ionotropic receptor AMPA type subunit 3	GRIA3
22758	UBE2S	1.719	9.079	4.027	0.088	0.010	0.116	ubiquitin conjugating enzyme E2 S	UBE2S
16556	PNPT1	1.719	0.746	0.888	0.244	0.433	0.559	polyribonucleotide nucleotidyltransferase 1	PNPT1
14512	NINJ2	1.719	1.289	1.789	0.101	0.258	0.011	ninjurin 2	NINJ2
11168	LOC10013309	1.719	0.553	0.810	0.201	0.177	0.456	uncharacterized LOC100133091	LOC100133091
19259	SLC16A10	1.717	0.702	1.096	0.131	0.237	0.346	solute carrier family 16 member 10	SLC16A10
14727	NPR3	1.717	0.627	1.807	0.701	0.719	0.598	natriuretic peptide receptor 3	NPR3
20230	SNRPA1	1.713	2.013	2.182	0.033	0.057	0.010	small nuclear ribonucleoprotein polypeptide A'	SNRPA1
20879	SUCO	1.713	0.720	0.868	0.087	0.167	0.213	SUN domain containing ossification factor	SUCO
11289	LOC10050612	1.712	0.584	1.000	0.201	0.201	0.910	uncharacterized LOC100506123	LOC100506123
16347	PLA1A	1.712	0.779	0.889	0.020	0.101	0.059	phospholipase A1 member A	PLA1A
18696	SCG2	1.712	0.207	0.435	0.659	0.402	0.397	secretogranin II	SCG2
16240	PIGV	1.712	0.968	1.050	0.058	0.863	0.325	phosphatidylinositol glycan anchor biosynthesis class V	PIGV
6601	FICD	1.712	0.817	0.871	0.022	0.450	0.481	FIC domain containing	FICD
10826	LINC01411	1.711	0.535	1.010	0.478	0.434	0.922	long intergenic non-protein coding RNA 1411	LINC01411
14976	NXPH4	1.709	0.410	0.934	0.389	0.206	0.880	neurexophilin 4	NXPH4
10025	LGALS8	1.708	0.495	0.900	0.417	0.318	0.765	lectin, galactoside binding soluble 8	LGALS8
16362	PLA2G4C	1.708	0.511	0.644	0.445	0.401	0.477	phospholipase A2 group IVC	PLA2G4C
762	ANKRD1	1.708	1.040	0.895	0.430	0.942	0.187	ankyrin repeat domain 1	ANKRD1
17309	PTPN2	1.707	0.661	1.173	0.043	0.080	0.623	protein tyrosine phosphatase, non-receptor type 2	PTPN2
518	AHR	1.707	0.588	0.745	0.096	0.029	0.428	aryl hydrocarbon receptor	AHR
22865	UHRF1BP1	1.705	0.771	1.140	0.101	0.319	0.224	UHRF1 binding protein 1	UHRF1BP1
4855	DHCR7	1.705	0.122	0.296	0.689	0.385	0.431	7-dehydrocholesterol reductase	DHCR7
17204	PSMD12	1.704	1.269	1.375	0.057	0.108	0.043	proteasome 26S subunit, non-ATPase 12	PSMD12
23742	ZG16B	1.704	0.892	1.445	0.213	0.725	0.426	zymogen granule protein 16B	ZG16B
9571	KLHL15	1.703	0.875	0.924	0.004	0.189	0.010	kelch like family member 15	KLHL15
22269	TRIM21	1.703	0.659	1.233	0.287	0.357	0.274	tripartite motif containing 21	TRIM21
4620	DCBLD2	1.702	0.874	0.865	0.151	0.593	0.476	discoidin, CUB and LCCL domain containing 2	DCBLD2
8162	HIST1H2BJ	1.699	0.800	0.883	0.570	0.758	0.808	histone cluster 1, H2bj	HIST1H2BJ
270	ADAM10	1.698	0.799	1.073	0.303	0.464	0.881	ADAM metallopeptidase domain 10	ADAM10
10893	LINC01503	1.698	0.899	0.838	0.100	0.543	0.118	long intergenic non-protein coding RNA 1503	LINC01503
1889	BTC	1.697	0.715	1.071	0.102	0.368	0.814	betacellulin	BTC
17850	REV3L	1.695	0.437	1.023	0.413	0.290	0.965	REV3 like, DNA directed polymerase zeta catalytic subunit	REV3L
7862	GYS1	1.695	0.836	1.312	0.271	0.644	0.148	glycogen synthase 1 (muscle)	GYS1
7879	H2AFJ	1.694	1.372	2.201	0.174	0.246	0.233	H2A histone family member J	H2AFJ
14880	NUCB2	1.694	1.522	1.600	0.005	0.055	0.015	nucleobindin 2	NUCB2
23259	WBP2	1.693	0.476	0.882	0.418	0.322	0.406	WW domain binding protein 2	WBP2
2349	C5orf15	1.693	0.801	0.998	0.037	0.222	0.989	chromosome 5 open reading frame 15	C5orf15
9665	KREMEN1	1.693	0.585	0.987	0.436	0.455	0.962	kringle containing transmembrane protein 1	KREMEN1
23094	VCAM1	1.692	0.385	0.729	0.518	0.050	0.788	vascular cell adhesion molecule 1	VCAM1
21980	TMTC1	1.692	0.598	0.647	0.153	0.209	0.044	transmembrane and tetratricopeptide repeat containing 1	TMTC1
23622	ZC3H12D	1.692	0.759	0.884	0.132	0.275	0.182	zinc finger CCCH-type containing 12D	ZC3H12D

10458	LINC00869	1.691	0.818	1.565	0.068	0.230	0.167	long intergenic non-protein coding RNA 869	LINC00869
15723	PARP10	1.691	0.576	1.271	0.224	0.166	0.451	poly(ADP-ribose) polymerase family member 10	PARP10
13571	MMD	1.690	1.818	2.001	0.040	0.352	0.390	monocyte to macrophage differentiation associated	MMD
19028	SFXN4	1.690	1.178	1.332	0.047	0.638	0.045	sideroflexin 4	SFXN4
15696	PAPPA-AS1	1.689	0.895	1.053	0.630	0.894	0.970	PAPPA antisense RNA 1	PAPPA-AS1
19025	SFXN1	1.689	1.031	1.256	0.006	0.751	0.157	sideroflexin 1	SFXN1
21092	TALDO1	1.689	1.127	1.439	0.286	0.759	0.008	transaldolase 1	TALDO1
12894	LZTS3	1.687	0.482	0.684	0.146	0.045	0.326	leucine zipper, putative tumor suppressor family member 3	LZTS3
9027	ISOC1	1.687	0.930	1.060	0.134	0.685	0.795	isochorismatase domain containing 1	ISOC1
17502	RAB4B	1.687	1.053	1.335	0.353	0.890	0.241	RAB4B, member RAS oncogene family	RAB4B
14002	MXI1	1.686	0.285	0.576	0.120	0.030	0.114	MAX interactor 1, dimerization protein	MXI1
21105	TAPBPL	1.685	0.677	0.886	0.128	0.212	0.421	TAP binding protein like	TAPBPL
1798	BOLA3	1.685	2.273	1.747	0.022	0.109	0.465	bolA family member 3	BOLA3
17349	PTS	1.684	1.718	1.075	0.026	0.085	0.496	6-pyruvoyltetrahydropterin synthase	PTS
1530	B3GNT5	1.683	0.792	0.938	0.255	0.473	0.811	UDP-GlcNAc:betaGal beta-1,3-N-acetylglucosaminyltransferase 5	B3GNT5
13410	MGC27382	1.683	0.831	1.867	0.217	0.590	0.414	uncharacterized MGC27382	MGC27382
23129	VIM-AS1	1.683	0.572	1.072	0.201	0.187	0.129	VIM antisense RNA 1	VIM-AS1
1705	BHLHA15	1.683	0.593	1.035	0.330	0.320	0.836	basic helix-loop-helix family member a15	BHLHA15
7874	H1FX	1.683	0.997	1.514	0.146	0.990	0.288	H1 histone family member X	H1FX
10089	LIMS1	1.683	0.526	0.805	0.326	0.251	0.516	LIM zinc finger domain containing 1	LIMS1
14767	NR3C1	1.683	0.434	0.764	0.187	0.084	0.383	nuclear receptor subfamily 3 group C member 1	NR3C1
22814	UCHL3	1.683	1.093	1.645	0.032	0.344	0.024	ubiquitin C-terminal hydrolase L3	UCHL3
634	ALG3	1.682	1.452	1.458	0.188	0.390	0.007	ALG3, alpha-1,3- mannosyltransferase	ALG3
20997	SYNPO	1.682	0.661	1.644	0.280	0.352	0.053	synaptopodin	SYNPO
14946	NUS1	1.681	0.904	1.028	0.007	0.339	0.659	NUS1 dehydrodolichyl diphosphate synthase subunit	NUS1
9948	LCMT1-AS1	1.680	0.808	1.381	0.110	0.339	0.382	LCMT1 antisense RNA 1	LCMT1-AS1
14930	NUP35	1.680	1.775	1.521	0.128	0.038	0.065	nucleoporin 35kDa	NUP35
7283	GJD3	1.680	0.825	1.543	0.093	0.219	0.277	gap junction protein delta 3	GJD3
14624	NOCT	1.680	0.858	1.258	0.068	0.252	0.518	nocturnin	NOCT
23527	YRDC	1.679	1.107	1.072	0.382	0.855	0.796	yrdC N6-threonylcarbamoyltransferase domain containing	YRDC
20749	STARD4	1.679	0.172	0.390	0.659	0.351	0.473	StAR related lipid transfer domain containing 4	STARD4
17332	PTPRJ	1.679	1.714	1.614	0.105	0.143	0.007	protein tyrosine phosphatase, receptor type J	PTPRJ
6756	FOSL1	1.677	1.784	1.557	0.469	0.274	0.489	FOS like 1, AP-1 trancription factor subunit	FOSL1
13471	MINPP1	1.676	0.945	1.086	0.004	0.368	0.394	multiple inositol-polyphosphate phosphatase 1	MINPP1
20517	SPPL2A	1.676	0.710	0.840	0.114	0.213	0.064	signal peptide peptidase like 2A	SPPL2A
1223	ARSI	1.676	1.360	2.231	0.159	0.444	0.056	arylsulfatase family member I	ARSI
1026	ARAP2	1.676	0.590	0.921	0.426	0.409	0.728	ArfGAP with RhoGAP domain, ankyrin repeat and PH domain 2	ARAP2
17938	RHBDF2	1.676	0.571	1.001	0.519	0.496	0.999	rhomboid 5 homolog 2 (Drosophila)	RHBDF2
19333	SLC25A12	1.675	0.705	1.051	0.101	0.174	0.808	solute carrier family 25 member 12	SLC25A12
13911	MTHFD2L	1.674	1.197	0.910	0.182	0.419	0.479	methylenetetrahydrofolate dehydrogenase (NADP+ dependent) 2-lik	MTHFD2L
1422	ATP6AP1	1.673	0.791	0.964	0.167	0.427	0.747	ATPase H+ transporting accessory protein 1	ATP6AP1
7549	GPR108	1.673	0.814	1.037	0.290	0.601	0.847	G protein-coupled receptor 108	GPR108
19165	SIK2	1.672	0.969	1.114	0.062	0.891	0.701	salt inducible kinase 2	SIK2
6445	FBXO42	1.672	0.594	0.855	0.104	0.102	0.060	F-box protein 42	FBXO42
14815	NRXN2	1.672	0.752	2.246	0.554	0.727	0.533	neurexin 2	NRXN2
9138	JMJD6	1.670	1.205	1.299	0.050	0.133	0.454	arginine demethylase and lysine hydroxylase	JMJD6
2447	C8orf88	1.670	1.184	1.598	0.053	0.274	0.342	chromosome 8 open reading frame 88	C8orf88
17571	RAI14	1.670	1.171	0.903	0.134	0.700	0.540	retinoic acid induced 14	RAI14
22070	TNS3	1.669	0.783	1.421	0.185	0.473	0.404	tensin 3	TNS3
12809	LTBP1	1.668	0.804	1.584	0.355	0.578	0.179	latent transforming growth factor beta binding protein 1	LTBP1
7486	GOPC	1.668	0.699	0.772	0.003	0.004	0.024	golgi-associated PDZ and coiled-coil motif containing	GOPC
12732	LRRC55	1.667	0.605	1.005	0.249	0.253	0.409	leucine rich repeat containing 55	LRRC55
10843	LINC01433	1.667	1.345	1.409	0.013	0.141	0.468	long intergenic non-protein coding RNA 1433	LINC01433
16472	PLOD3	1.666	1.046	1.639	0.058	0.695	0.123	procollagen-lysine,2-oxoglutarate 5-dioxygenase 3	PLOD3
14622	NOC3L	1.666	0.946	1.192	0.024	0.573	0.136	NOC3 like DNA replication regulator	NOC3L
7244	GIMAP2	1.666	0.457	0.977	0.073	0.013	0.901	GTPase, IMAP family member 2	GIMAP2
22169	TPRG1-AS1	1.666	0.923	1.103	0.352	0.838	0.421	TPRG1 antisense RNA 1	TPRG1-AS1
19476	SLC36A4	1.665	0.872	0.980	0.205	0.568	0.931	solute carrier family 36 member 4	SLC36A4
5749	EPS8L2	1.665	0.366	0.892	0.318	0.174	0.713	EPS8 like 2	EPS8L2
2145	C1GALT1C1L	1.665	0.793	1.300	0.149	0.351	0.209	C1GALT1-specific chaperone 1 like	C1GALT1C1L
8796	IL1RAP	1.664	0.617	0.880	0.336	0.352	0.133	interleukin 1 receptor accessory protein	IL1RAP
14667	NOVA1	1.664	0.990	1.106	0.328	0.980	0.899	NOVA alternative splicing regulator 1	NOVA1
3365	CEP350	1.664	0.624	1.021	0.224	0.185	0.947	centrosomal protein 350	CEP350
13208	MCOLN2	1.663	0.846	1.042	0.058	0.261	0.851	mucolipin 2	MCOLN2
7813	GTPBP2	1.663	0.621	0.898	0.121	0.134	0.426	GTP binding protein 2	GTPBP2
15965	PDE8A	1.662	0.969	0.952	0.044	0.882	0.454	phosphodiesterase 8A	PDE8A
5403	EDEM3	1.662	0.826	0.979	0.054	0.243	0.862	ER degradation enhancing alpha-mannosidase like protein 3	EDEM3
7811	GTPBP1	1.662	0.783	1.048	0.227	0.433	0.731	GTP binding protein 1	GTPBP1
3085	CD47	1.661	0.538	0.815	0.220	0.173	0.456	CD47 molecule	CD47
22068	TNS1	1.661	0.599	1.018	0.035	0.051	0.796	tensin 1	TNS1
13234	ME1	1.661	0.353	0.643	0.520	0.316	0.430	malic enzyme 1	ME1
5436	EFCAB2	1.660	0.832	1.086	0.020	0.040	0.459	EF-hand calcium binding domain 2	EFCAB2
23009	USP30-AS1	1.660	0.584	0.843	0.144	0.127	0.314	USP30 antisense RNA 1	USP30-AS1
9719	KRT75	1.660	0.648	0.870	0.214	0.197	0.659	keratin 75	KRT75
11050	LMTK2	1.660	0.720	0.811	0.374	0.486	0.632	lemur tyrosine kinase 2	LMTK2
23608	ZBTB7B	1.660	0.740	1.026	0.273	0.442	0.874	zinc finger and BTB domain containing 7B	ZBTB7B
7401	GNE	1.660	0.622	0.775	0.264	0.273	0.424	glucosamine (UDP-N-acetyl)-2-epimerase/N-acetylmannosamine kina	GNE
22199	TRAF2	1.659	1.140	1.211	0.120	0.156	0.500	TNF receptor associated factor 2	TRAF2
5861	EVA1A	1.658	1.188	1.280	0.513	0.753	0.712	eva-1 homolog A, regulator of programmed cell death	EVA1A
15003	OCIAD2	1.657	1.037	1.275	0.115	0.780	0.677	OCIA domain containing 2	OCIAD2
24047	ZNF503-AS2	1.657	0.856	1.283	0.251	0.602	0.211	ZNF503 antisense RNA 2	ZNF503-AS2
298	ADAMTS10	1.656	0.683	0.953	0.244	0.045	0.940	ADAM metallopeptidase with thrombospondin type 1 motif 10	ADAMTS10
19591	SLC7A5P1	1.656	1.032	1.152	0.118	0.918	0.202	solute carrier family 7 member 5 pseudogene 1	SLC7A5P1
19395	SLC26A9	1.656	2.353	1.246	0.098	0.210	0.446	solute carrier family 26 member 9	SLC26A9
18915	SERF2-C15ORI	1.656	0.604	1.274	0.069	0.069	0.072	SERF2-C15orf63 readthrough	SERF2-C15ORF63
19092	SH3PXD2B	1.656	0.374	0.793	0.519	0.312	0.635	SH3 and PX domains 2B	SH3PXD2B
9713	KRT7	1.655	7.393	4.090	0.007	0.172	0.012	keratin 7	KRT7
21863	TMEM30A	1.654	0.877	0.949	0.197	0.711	0.771	transmembrane protein 30A	TMEM30A
21065	TAF13	1.654	1.084	0.957	0.047	0.562	0.801	TATA-box binding protein associated factor 13	TAF13
21352	TENM2	1.653	0.416	0.825	0.677	0.540	0.727	teneurin transmembrane protein 2	TENM2
20806	STK40	1.653	0.625	1.198	0.048	0.065	0.051	serine/threonine kinase 40	STK40

6510	FEM1C	1.653	0.540	0.754	0.074	0.055	0.231	fem-1 homolog C	FEM1C
13902	MTFP1	1.653	1.285	1.126	0.073	0.462	0.523	mitochondrial fission process 1	MTFP1
9134	JKAMP	1.651	0.972	0.938	0.050	0.623	0.688	JNK1/MAPK8-associated membrane protein	JKAMP
18328	RPN1	1.651	1.102	1.264	0.002	0.206	0.045	ribophorin I	RPN1
5045	DNAJB11	1.649	1.419	1.456	0.107	0.113	0.447	DnaJ heat shock protein family (Hsp40) member B11	DNAJB11
24191	ZNF672	1.649	0.939	1.272	0.007	0.500	0.400	zinc finger protein 672	ZNF672
24361	ZNRF1	1.648	0.942	1.286	0.249	0.857	0.286	zinc and ring finger 1, E3 ubiquitin protein ligase	ZNRF1
1349	ATG7	1.648	0.986	1.226	0.306	0.966	0.353	autophagy related 7	ATG7
20490	SPINK4	1.648	1.139	1.145	0.423	0.819	0.422	serine peptidase inhibitor, Kazal type 4	SPINK4
5965	FADS3	1.646	0.721	0.982	0.063	0.340	0.964	fatty acid desaturase 3	FADS3
21561	TIMP1	1.646	1.635	2.493	0.146	0.433	0.372	TIMP metallopeptidase inhibitor 1	TIMP1
21056	TACSTD2	1.645	0.622	1.097	0.076	0.078	0.690	tumor-associated calcium signal transducer 2	TACSTD2
4633	DCLK3	1.643	0.699	1.000	0.282	0.380	0.943	doublecortin like kinase 3	DCLK3
492	AGPAT4	1.643	0.829	1.549	0.153	0.442	0.557	1-acylglycerol-3-phosphate O-acyltransferase 4	AGPAT4
10836	LINC01426	1.643	1.226	1.818	0.310	0.486	0.504	long intergenic non-protein coding RNA 1426	LINC01426
24122	ZNF585A	1.643	0.775	0.983	0.092	0.229	0.870	zinc finger protein 585A	ZNF585A
22081	TOM1	1.643	0.756	1.088	0.169	0.394	0.798	target of myb1 membrane trafficking protein	TOM1
21899	TMEM59	1.642	0.704	1.068	0.116	0.179	0.753	transmembrane protein 59	TMEM59
6808	FOXP1	1.642	1.062	1.490	0.047	0.842	0.464	forkhead box P1	FOXP1
17782	RCL1	1.642	1.075	2.059	0.130	0.679	0.045	RNA terminal phosphate cyclase like 1	RCL1
405	ADM	1.642	0.921	1.051	0.168	0.724	0.917	adrenomedullin	ADM
22180	TPST1	1.642	0.817	1.241	0.064	0.411	0.701	tyrosylprotein sulfotransferase 1	TPST1
5808	ERO1B	1.642	0.592	0.839	0.071	0.057	0.436	endoplasmic reticulum oxidoreductase beta	ERO1B
7172	GDNF	1.641	2.104	0.953	0.523	0.178	0.782	glial cell derived neurotrophic factor	GDNF
6042	FAM13A	1.641	0.521	0.968	0.030	0.012	0.746	family with sequence similarity 13 member A	FAM13A
18954	SERPINE1	1.641	0.976	1.726	0.584	0.973	0.532	serpin family E member 1	SERPINE1
5811	ERP44	1.641	0.958	1.082	0.030	0.639	0.490	endoplasmic reticulum protein 44	ERP44
23377	WISP1	1.641	0.438	0.897	0.642	0.521	0.763	WNT1 inducible signaling pathway protein 1	WISP1
10966	LINC01588	1.641	1.180	0.930	0.072	0.703	0.748	long intergenic non-protein coding RNA 1588	LINC01588
17203	PSMD11	1.641	1.197	1.585	0.062	0.210	0.080	proteasome 26S subunit, non-ATPase 11	PSMD11
6166	FAM214B	1.640	0.793	1.272	0.367	0.560	0.457	family with sequence similarity 214 member B	FAM214B
9831	KSR1	1.640	0.793	1.112	0.123	0.377	0.501	kinase suppressor of ras 1	KSR1
23900	ZNF281	1.640	1.041	1.260	0.183	0.877	0.652	zinc finger protein 281	ZNF281
1640	BCL2	1.639	0.556	0.528	0.573	0.513	0.214	B-cell CLL/lymphoma 2	BCL2
16998	PRMT1	1.638	1.492	1.854	0.114	0.100	0.095	protein arginine methyltransferase 1	PRMT1
549	AK7	1.638	0.605	0.897	0.226	0.205	0.516	adenylate kinase 7	AK7
5835	ESRRA	1.638	0.893	1.201	0.021	0.671	0.380	estrogen related receptor alpha	ESRRA
8822	IL2RG	1.637	0.622	1.052	0.340	0.351	0.424	interleukin 2 receptor subunit gamma	IL2RG
2374	C6orf1	1.637	0.798	0.963	0.552	0.736	0.932	chromosome 6 open reading frame 1	C6orf1
6946	FZD8	1.637	0.670	0.946	0.567	0.603	0.923	frizzled class receptor 8	FZD8
5668	ENPP2	1.637	0.401	1.552	0.621	0.465	0.469	ectonucleotide pyrophosphatase/phosphodiesterase 2	ENPP2
13843	MSI2	1.636	0.775	1.051	0.439	0.676	0.891	musashi RNA binding protein 2	MSI2
62	ABCB6	1.636	0.890	1.206	0.102	0.559	0.559	ATP binding cassette subfamily B member 6 (Langereis blood group)	ABCB6
16151	PHF11	1.636	0.740	1.169	0.121	0.222	0.244	PHD finger protein 11	PHF11
10063	LIFR	1.635	0.568	1.101	0.077	0.101	0.466	leukemia inhibitory factor receptor alpha	LIFR
3064	CD300E	1.635	0.655	0.954	0.326	0.370	0.289	CD300e molecule	CD300E
9199	KCCAT211	1.635	0.577	0.716	0.175	0.033	0.440	renal clear cell carcinoma-associated transcript 211	KCCAT211
5695	EPAS1	1.635	0.810	0.908	0.268	0.509	0.709	endothelial PAS domain protein 1	EPAS1
13284	MEFV	1.635	0.698	1.060	0.311	0.418	0.009	Mediterranean fever	MEFV
18857	SEMA3F	1.635	1.185	0.938	0.401	0.772	0.823	semaphorin 3F	SEMA3F
16554	PNPLA8	1.634	0.697	0.873	0.229	0.282	0.523	patatin like phospholipase domain containing 8	PNPLA8
6879	FSTL1	1.633	0.772	1.126	0.583	0.718	0.868	follistatin like 1	FSTL1
22128	TP53RK	1.633	0.838	1.182	0.250	0.575	0.008	TP53 regulating kinase	TP53RK
19042	SGMS1	1.633	0.579	0.792	0.045	0.032	0.175	sphingomyelin synthase 1	SGMS1
21032	SYVN1	1.633	0.658	0.996	0.067	0.105	0.951	synoviolin 1	SYVN1
6094	FAM174A	1.632	0.957	0.968	0.057	0.536	0.829	family with sequence similarity 174 member A	FAM174A
20770	STC2	1.631	1.016	0.849	0.542	0.978	0.520	stanniocalcin 2	STC2
7431	GNS	1.631	0.581	0.836	0.316	0.231	0.647	glucosamine (N-acetyl)-6-sulfatase	GNS
11581	LOC10192748	1.631	1.000	1.284	0.070	1.000	0.171	uncharacterized LOC101927481	LOC101927481
7035	GALNT1	1.630	1.424	1.604	0.603	0.631	0.675	polypeptide N-acetylgalactosaminyltransferase 1	GALNT1
174	ACOT1	1.630	0.662	1.018	0.032	0.038	0.850	acyl-CoA thioesterase 1	ACOT1
7602	GPR39	1.629	0.604	0.990	0.492	0.483	0.967	G protein-coupled receptor 39	GPR39
23318	WDR66	1.629	0.644	0.948	0.133	0.140	0.782	WD repeat domain 66	WDR66
22256	TRIB1	1.629	0.915	1.310	0.046	0.743	0.223	tribbles pseudokinase 1	TRIB1
12882	LYVE1	1.628	1.063	2.827	0.473	0.909	0.321	lymphatic vessel endothelial hyaluronan receptor 1	LYVE1
23176	VPS37C	1.628	0.812	1.043	0.121	0.327	0.603	VPS37C, ESCRT-I subunit	VPS37C
1864	BSCL2	1.628	1.285	1.095	0.055	0.078	0.617	Berardinelli-Seip congenital lipodystrophy 2 (seipin)	BSCL2
16080	PFDN6	1.626	1.568	1.788	0.084	0.091	0.220	prefoldin subunit 6	PFDN6
13556	MLLT4	1.626	0.979	1.102	0.163	0.922	0.522	myeloid/lymphoid or mixed-lineage leukemia	MLLT4
14977	NXT1	1.625	1.285	1.539	0.019	0.270	0.075	nuclear transport factor 2 like export factor 1	NXT1
8111	HIF1A-AS2	1.624	2.364	2.431	0.273	0.025	0.197	HIF1A antisense RNA 2	HIF1A-AS2
21669	TMED7	1.624	0.714	0.846	0.073	0.024	0.546	transmembrane p24 trafficking protein 7	TMED7
3425	CFAP58-AS1	1.623	1.343	2.036	0.205	0.415	0.372	CFAP58 antisense RNA 1 (head to head)	CFAP58-AS1
14539	NKILA	1.622	0.789	1.487	0.106	0.301	0.049	NF-kappaB interacting long non-coding RNA	NKILA
24375	ZPR1	1.622	1.478	1.501	0.033	0.039	0.152	ZPR1 zinc finger	ZPR1
16635	POM121L9P	1.622	3.083	4.727	0.475	0.111	0.289	POM121 transmembrane nucleoporin like 9, pseudogene	POM121L9P
20606	SRPK1	1.621	1.152	1.171	0.238	0.566	0.282	SRSF protein kinase 1	SRPK1
5857	ETV4	1.621	1.564	1.048	0.044	0.281	0.753	ETS variant 4	ETV4
22832	UFSP1	1.619	0.812	1.844	0.074	0.426	0.252	UFM1-specific peptidase 1 (inactive)	UFSP1
5752	EPT1	1.619	0.948	1.048	0.109	0.699	0.782	ethanolaminephosphotransferase 1	EPT1
14342	NDUFAF4	1.619	1.047	1.213	0.229	0.878	0.053	NADH:ubiquinone oxidoreductase complex assembly factor 4	NDUFAF4
8057	HERC3	1.618	0.545	0.992	0.457	0.363	0.989	HECT and RLD domain containing E3 ubiquitin protein ligase 3	HERC3
6619	FKBP14	1.617	0.935	0.975	0.005	0.574	0.913	FK506 binding protein 14	FKBP14
11027	LMCD1	1.617	0.246	0.680	0.676	0.422	0.621	LIM and cysteine rich domains 1	LMCD1
22620	TUBE1	1.616	0.718	0.948	0.026	0.049	0.702	tubulin epsilon 1	TUBE1
22002	TNFRSF10A	1.616	0.680	1.072	0.462	0.487	0.851	tumor necrosis factor receptor superfamily member 10a	TNFRSF10A
16301	PITPNM1	1.616	1.454	1.747	0.060	0.008	0.243	phosphatidylinositol transfer protein membrane associated 1	PITPNM1
1928	C10orf11	1.616	0.804	1.465	0.191	0.421	0.161	chromosome 10 open reading frame 11	C10orf11
6570	FGF7	1.615	0.817	1.366	0.706	0.875	0.793	fibroblast growth factor 7	FGF7
9024	ISM1	1.615	0.517	1.294	0.490	0.294	0.777	isthmin 1, angiogenesis inhibitor	ISM1
12032	LOC10192972	1.615	0.665	0.999	0.360	0.421	0.995	uncharacterized LOC101929723	LOC101929723

21106	TAPT1	1.614	0.810	0.970	0.005	0.110	0.203	transmembrane anterior posterior transformation 1	TAPT1
22399	TRPV4	1.614	1.089	1.246	0.041	0.361	0.596	transient receptor potential cation channel subfamily V member 4	TRPV4
22342	TRIO	1.614	0.532	0.762	0.134	0.024	0.616	trio Rho guanine nucleotide exchange factor	TRIO
22299	TRIM47	1.614	0.381	1.114	0.111	0.017	0.852	tripartite motif containing 47	TRIM47
22096	TOP1	1.613	1.042	1.105	0.077	0.726	0.421	topoisomerase (DNA) I	TOP1
22270	TRIM22	1.613	0.280	0.656	0.074	0.006	0.174	tripartite motif containing 22	TRIM22
6901	FUCA2	1.613	1.211	1.231	0.027	0.074	0.210	fucosidase, alpha-L- 2, plasma	FUCA2
15038	OLFM1	1.613	0.349	0.417	0.551	0.193	0.491	olfactomedin 1	OLFM1
4293	CTBS	1.612	0.490	0.693	0.101	0.003	0.312	chitobiase	CTBS
14660	NOTCH2	1.612	0.646	0.914	0.106	0.130	0.716	notch 2	NOTCH2
3489	CHD6	1.610	0.629	0.924	0.045	0.067	0.678	chromodomain helicase DNA binding protein 6	CHD6
14167	NAF1	1.610	0.747	1.013	0.020	0.039	0.929	nuclear assembly factor 1 ribonucleoprotein	NAF1
1347	ATG4D	1.610	1.168	1.288	0.024	0.362	0.017	autophagy related 4D cysteine peptidase	ATG4D
4232	CSRP1	1.610	1.137	1.512	0.329	0.728	0.249	cysteine and glycine rich protein 1	CSRP1
22338	TRIM8	1.610	0.659	0.934	0.033	0.024	0.621	tripartite motif containing 8	TRIM8
22248	TREX1	1.610	0.566	0.915	0.328	0.277	0.549	three prime repair exonuclease 1	TREX1
12987	MAMDC4	1.610	0.945	1.143	0.113	0.770	0.587	MAM domain containing 4	MAMDC4
7520	GPC4	1.609	0.770	0.723	0.185	0.322	0.373	glypican 4	GPC4
6453	FBXO6	1.609	0.480	0.828	0.215	0.142	0.536	F-box protein 6	FBXO6
8260	HMGA2	1.609	1.133	1.057	0.615	0.844	0.937	high mobility group AT-hook 2	HMGA2
12962	MAGEF1	1.608	1.041	1.338	0.021	0.587	0.164	MAGE family member F1	MAGEF1
8424	HR	1.608	0.368	0.957	0.519	0.306	0.914	hair growth associated	HR
23174	VPS37A	1.607	0.761	0.923	0.210	0.321	0.761	VPS37A, ESCRT-I subunit	VPS37A
12826	LURAP1L	1.606	0.303	0.684	0.278	0.116	0.362	leucine rich adaptor protein 1 like	LURAP1L
20584	SRF	1.605	0.800	1.036	0.044	0.091	0.823	serum response factor	SRF
15654	PAIP2	1.604	0.904	1.222	0.261	0.712	0.300	poly(A) binding protein interacting protein 2	PAIP2
8100	HIAT1	1.604	0.453	0.650	0.420	0.212	0.460	.	HIAT1
14661	NOTCH2NL	1.604	0.746	1.107	0.113	0.306	0.851	notch 2 N-terminal like	NOTCH2NL
1856	BRPF3	1.604	0.666	0.840	0.170	0.143	0.508	bromodomain and PHD finger containing 3	BRPF3
22650	TWIST2	1.604	1.512	1.575	0.187	0.213	0.166	twist family bHLH transcription factor 2	TWIST2
6753	FOPNL	1.603	1.047	0.981	0.051	0.577	0.868	FGFR1OP N-terminal like	FOPNL
22697	UAP1	1.603	1.730	1.752	0.045	0.109	0.133	UDP-N-acetylglucosamine pyrophosphorylase 1	UAP1
22409	TSC22D1	1.603	0.638	0.890	0.440	0.491	0.783	TSC22 domain family member 1	TSC22D1
14153	NAB1	1.602	0.950	0.867	0.280	0.853	0.506	NGFI-A binding protein 1	NAB1
23485	XRN1	1.602	0.534	0.877	0.167	0.137	0.415	5'-3' exoribonuclease 1	XRN1
17109	PRRX1	1.601	1.293	1.606	0.247	0.367	0.277	paired related homeobox 1	PRRX1
515	AHI1	1.601	0.830	0.841	0.279	0.537	0.484	Abelson helper integration site 1	AHI1
4370	CUL4B	1.601	2.128	2.412	0.265	0.187	0.414	cullin 4B	CUL4B
19658	SLMO2-ATP5E	1.601	1.190	1.141	0.357	0.567	0.835	SLMO2-ATP5E readthrough	SLMO2-ATP5E
4439	CYB5R2	1.601	1.053	1.458	0.516	0.923	0.316	cytochrome b5 reductase 2	CYB5R2
2413	C7orf60	1.600	0.669	0.962	0.270	0.259	0.880	.	C7orf60
23022	USP40	1.600	0.897	1.177	0.074	0.434	0.032	ubiquitin specific peptidase 40	USP40
4198	CSGALNACT2	1.600	0.986	1.129	0.157	0.942	0.798	chondroitin sulfate N-acetylgalactosaminyltransferase 2	CSGALNACT2
20567	SQLE	1.599	0.220	0.449	0.633	0.333	0.347	squalene epoxidase	SQLE
16781	PPP1R15B	1.597	0.639	0.880	0.357	0.321	0.763	protein phosphatase 1 regulatory subunit 15B	PPP1R15B
6635	FKRP	1.597	0.996	1.183	0.032	0.974	0.349	fukutin related protein	FKRP
7650	GRAMD1A	1.597	0.701	0.994	0.464	0.548	0.973	GRAM domain containing 1A	GRAMD1A
6548	FGF11	1.597	0.350	0.687	0.646	0.415	0.599	fibroblast growth factor 11	FGF11
6007	FAM120C	1.596	0.821	1.051	0.172	0.422	0.517	family with sequence similarity 120C	FAM120C
18625	SAV1	1.595	0.366	0.727	0.257	0.113	0.085	salvador family WW domain containing protein 1	SAV1
20634	SRXN1	1.594	0.812	0.845	0.353	0.656	0.394	sulfiredoxin 1	SRXN1
23089	VAV3	1.594	0.650	0.963	0.344	0.376	0.712	vav guanine nucleotide exchange factor 3	VAV3
17175	PSMA3	1.594	1.226	1.466	0.138	0.279	0.056	proteasome subunit alpha 3	PSMA3
8990	IRF2	1.594	0.670	1.106	0.168	0.103	0.680	interferon regulatory factor 2	IRF2
16085	PFKL	1.594	0.749	1.110	0.013	0.012	0.269	phosphofructokinase, liver type	PFKL
14678	NPAS2	1.593	0.843	0.707	0.007	0.498	0.026	neuronal PAS domain protein 2	NPAS2
13550	MLKL	1.593	1.131	1.937	0.295	0.664	0.195	mixed lineage kinase domain-like	MLKL
6375	FASTKD5	1.593	0.832	0.899	0.090	0.252	0.447	FAST kinase domains 5	FASTKD5
1398	ATP2C1	1.593	0.705	0.882	0.091	0.036	0.568	ATPase secretory pathway Ca2+ transporting 1	ATP2C1
15761	PAX8-AS1	1.593	0.475	0.582	0.602	0.423	0.621	PAX8 antisense RNA 1	PAX8-AS1
4990	DLX4	1.592	0.770	1.166	0.294	0.509	0.811	distal-less homeobox 4	DLX4
7198	GFPT1	1.592	0.748	0.787	0.028	0.065	0.030	glutamine--fructose-6-phosphate transaminase 1	GFPT1
4701	DDX21	1.592	1.431	1.614	0.031	0.071	0.088	DEAD-box helicase 21	DDX21
21088	TAGLN2	1.591	1.632	2.269	0.020	0.023	0.042	transgelin 2	TAGLN2
8788	IL18R1	1.591	0.527	0.936	0.137	0.044	0.804	interleukin 18 receptor 1	IL18R1
15874	PCED1B	1.591	1.109	0.871	0.338	0.763	0.469	PC-esterase domain containing 1B	PCED1B
9840	L1CAM	1.590	0.569	0.889	0.070	0.056	0.319	L1 cell adhesion molecule	L1CAM
4249	CSTB	1.590	0.601	0.905	0.562	0.525	0.835	cystatin B	CSTB
18584	SAMD4A	1.589	0.605	0.810	0.215	0.304	0.214	sterile alpha motif domain containing 4A	SAMD4A
18307	RPL36A-HNRN	1.589	0.861	2.274	0.370	0.268	0.266	RPL36A-HNRNPH2 readthrough	RPL36A-HNRNPH2
1027	ARAP3	1.589	1.052	1.283	0.013	0.809	0.001	ArfGAP with RhoGAP domain, ankyrin repeat and PH domain 3	ARAP3
22660	TXNDC11	1.589	0.922	1.118	0.050	0.529	0.265	thioredoxin domain containing 11	TXNDC11
17822	REL	1.589	0.621	0.886	0.026	0.050	0.197	v-rel avian reticuloendotheliosis viral oncogene homolog	REL
12858	LYPD1	1.588	0.683	0.881	0.476	0.528	0.639	LY6/PLAUR domain containing 1	LYPD1
13187	MCFD2	1.588	0.936	0.860	0.088	0.810	0.592	multiple coagulation factor deficiency 2	MCFD2
7586	GPR183	1.588	1.679	1.126	0.102	0.096	0.392	G protein-coupled receptor 183	GPR183
6716	FMNL3	1.587	0.737	1.038	0.472	0.593	0.824	formin like 3	FMNL3
14783	NRCAM	1.587	0.779	0.945	0.324	0.574	0.090	neuronal cell adhesion molecule	NRCAM
3248	CDR2	1.587	0.809	0.837	0.217	0.486	0.394	cerebellar degeneration related protein 2	CDR2
2155	C1QTNF1-AS1	1.587	1.153	1.046	0.041	0.265	0.706	C1QTNF1 antisense RNA 1	C1QTNF1-AS1
14123	MZT1	1.587	2.439	1.301	0.070	0.007	0.513	mitotic spindle organizing protein 1	MZT1
178	ACOT2	1.586	0.763	1.136	0.081	0.186	0.102	acyl-CoA thioesterase 2	ACOT2
6407	FBXL19-AS1	1.586	0.770	1.148	0.213	0.425	0.700	FBXL19 antisense RNA 1 (head to head)	FBXL19-AS1
5613	EMC3	1.585	0.967	1.328	0.022	0.612	0.463	ER membrane protein complex subunit 3	EMC3
16778	PPP1R14C	1.584	1.436	1.721	0.219	0.397	0.459	protein phosphatase 1 regulatory inhibitor subunit 14C	PPP1R14C
17241	PTBP3	1.584	0.785	1.123	0.035	0.127	0.165	polypyrimidine tract binding protein 3	PTBP3
9403	KIAA0754	1.584	0.827	0.666	0.192	0.525	0.300	KIAA0754	KIAA0754
3601	CIDECP	1.584	1.185	1.657	0.169	0.378	0.300	cell death-inducing DFFA-like effector c pseudogene	CIDECP
23649	ZCCHC5	1.583	1.192	1.190	0.048	0.022	0.594	zinc finger CCHC-type containing 5	ZCCHC5
16124	PGM3	1.583	1.062	1.291	0.041	0.522	0.462	phosphoglucomutase 3	PGM3
23650	ZCCHC6	1.583	0.825	1.142	0.114	0.481	0.640	zinc finger CCHC-type containing 6	ZCCHC6

16414	PLD6	1.582	0.730	0.918	0.237	0.467	0.692	phospholipase D family member 6	PLD6
13850	MSMO1	1.581	0.106	0.261	0.731	0.389	0.407	methylsterol monooxygenase 1	MSMO1
14722	NPPA-AS1	1.581	0.472	0.726	0.608	0.459	0.562	NPPA antisense RNA 1	NPPA-AS1
7043	GALNT18	1.580	1.212	1.164	0.389	0.497	0.693	polypeptide N-acetylgalactosaminyltransferase 18	GALNT18
10649	LINC01140	1.580	0.606	1.084	0.017	0.012	0.788	long intergenic non-protein coding RNA 1140	LINC01140
5907	EXOSC9	1.580	1.943	1.840	0.105	0.032	0.406	exosome component 9	EXOSC9
19957	SNORA8	1.579	1.838	0.667	0.574	0.507	0.772	small nucleolar RNA, H/ACA box 8	SNORA8
3894	COL16A1	1.579	0.822	0.884	0.552	0.675	0.897	collagen type XVI alpha 1	COL16A1
11125	LOC10013047	1.578	0.875	1.231	0.013	0.086	0.206	uncharacterized LOC100130476	LOC100130476
9574	KLHL2	1.577	0.811	0.780	0.348	0.573	0.326	kelch like family member 2	KLHL2
6582	FGFRL1	1.577	1.219	1.118	0.434	0.664	0.788	fibroblast growth factor receptor-like 1	FGFRL1
11090	LOC10012914	1.577	0.784	1.332	0.298	0.484	0.111	uncharacterized LOC100129148	LOC100129148
14462	NFIL3	1.576	0.619	0.865	0.263	0.170	0.657	nuclear factor, interleukin 3 regulated	NFIL3
12922	MAFG	1.576	0.659	0.874	0.339	0.403	0.227	v-maf avian musculoaponeurotic fibrosarcoma oncogene homolog G	MAFG
3913	COL4A1	1.576	0.746	1.154	0.484	0.653	0.766	collagen type IV alpha 1	COL4A1
8510	HSPA5	1.575	1.001	1.363	0.229	0.996	0.494	heat shock protein family A (Hsp70) member 5	HSPA5
24173	ZNF653	1.575	1.026	1.171	0.061	0.831	0.562	zinc finger protein 653	ZNF653
13005	MANEA	1.575	0.702	0.741	0.041	0.118	0.181	mannosidase endo-alpha	MANEA
6392	FBN1	1.575	0.755	1.322	0.584	0.707	0.589	fibrillin 1	FBN1
6502	FDPSP2	1.574	0.582	0.766	0.276	0.171	0.455	farnesyl diphosphate synthase pseudogene 2	FDPSP2
13524	MKL1	1.574	0.828	1.139	0.032	0.039	0.313	megakaryoblastic leukemia (translocation) 1	MKL1
19438	SLC33A1	1.574	0.575	0.775	0.226	0.174	0.390	solute carrier family 33 member 1	SLC33A1
23787	ZMYND8	1.574	1.122	1.755	0.186	0.627	0.249	zinc finger MYND-type containing 8	ZMYND8
14934	NUP50-AS1	1.574	1.307	1.377	0.069	0.135	0.511	NUP50 antisense RNA 1 (head to head)	NUP50-AS1
17498	RAB42	1.573	0.652	0.772	0.249	0.304	0.623	RAB42, member RAS oncogene family	RAB42
23538	YWHAG	1.573	0.902	1.116	0.172	0.640	0.527	tyrosine 3-monooxygenase/tryptophan 5-monooxygenase activation	YWHAG
19546	SLC52A2	1.573	1.293	1.293	0.188	0.396	0.170	solute carrier family 52 member 2	SLC52A2
13978	MUC5AC	1.572	0.841	1.009	0.410	0.696	0.413	mucin 5AC, oligomeric mucus/gel-forming	MUC5AC
14371	NDUFV2	1.572	1.394	1.603	0.164	0.155	0.051	NADH:ubiquinone oxidoreductase core subunit V2	NDUFV2
5563	ELANE	1.572	1.016	1.519	0.407	0.964	0.479	elastase, neutrophil expressed	ELANE
22667	TXNDC5	1.571	1.038	1.378	0.213	0.869	0.314	thioredoxin domain containing 5	TXNDC5
2078	C17orf100	1.571	0.611	1.250	0.074	0.052	0.159	chromosome 17 open reading frame 100	C17orf100
15518	OSMR-AS1	1.570	0.678	0.995	0.043	0.037	0.979	OSMR antisense RNA 1 (head to head)	OSMR-AS1
6944	FZD6	1.568	0.878	0.900	0.163	0.445	0.824	frizzled class receptor 6	FZD6
5683	ENTPD4	1.566	0.913	0.956	0.035	0.324	0.639	ectonucleoside triphosphate diphosphohydrolase 4	ENTPD4
6898	FUBP1	1.566	1.276	1.233	0.008	0.056	0.024	far upstream element binding protein 1	FUBP1
22029	TNFSF14	1.566	0.786	0.987	0.169	0.436	0.743	tumor necrosis factor superfamily member 14	TNFSF14
620	ALDOA	1.565	0.789	1.032	0.145	0.327	0.882	aldolase, fructose-bisphosphate A	ALDOA
21634	TM9SF4	1.564	0.783	1.057	0.150	0.252	0.824	transmembrane 9 superfamily member 4	TM9SF4
20738	STAM2	1.564	0.842	0.859	0.152	0.356	0.553	signal transducing adaptor molecule 2	STAM2
12184	LOC10527430	1.563	1.427	0.971	0.423	0.594	0.894	uncharacterized LOC105274304	LOC105274304
973	APOBEC3G	1.563	0.770	1.313	0.042	0.086	0.384	apolipoprotein B mRNA editing enzyme catalytic subunit 3G	APOBEC3G
16900	PRDM1	1.563	1.796	1.131	0.144	0.033	0.263	PR domain 1	PRDM1
12615	LPAR4	1.562	1.645	1.263	0.010	0.149	0.265	lysophosphatidic acid receptor 4	LPAR4
8743	IGSF8	1.562	0.800	0.821	0.121	0.107	0.470	immunoglobulin superfamily member 8	IGSF8
14942	NUPL1	1.562	1.057	1.071	0.163	0.703	0.839	.	NUPL1
24408	ZSWIM5	1.561	0.676	0.885	0.055	0.084	0.117	zinc finger SWIM-type containing 5	ZSWIM5
2714	CATIP-AS2	1.561	0.672	1.156	0.077	0.091	0.288	CATIP antisense RNA 2	CATIP-AS2
604	ALDH1B1	1.561	0.847	1.120	0.397	0.677	0.615	aldehyde dehydrogenase 1 family member B1	ALDH1B1
6880	FSTL3	1.559	2.314	1.926	0.373	0.047	0.209	follistatin like 3	FSTL3
4662	DDA1	1.559	1.062	1.105	0.139	0.707	0.566	DET1 and DDB1 associated 1	DDA1
5989	FAM107B	1.558	1.402	1.118	0.088	0.151	0.558	family with sequence similarity 107 member B	FAM107B
22323	TRIM63	1.558	0.553	0.862	0.482	0.396	0.010	tripartite motif containing 63	TRIM63
23063	UXS1	1.557	1.187	1.043	0.034	0.050	0.806	UDP-glucuronate decarboxylase 1	UXS1
14242	NBPF25P	1.556	0.933	1.544	0.022	0.819	0.389	neuroblastoma breakpoint family member 25, pseudogene	NBPF25P
14335	NDUFA7	1.556	1.967	2.527	0.372	0.119	0.035	NADH:ubiquinone oxidoreductase subunit A7	NDUFA7
23967	ZNF385A	1.556	0.388	0.823	0.405	0.217	0.228	zinc finger protein 385A	ZNF385A
22071	TNS4	1.555	1.277	1.696	0.448	0.633	0.492	tensin 4	TNS4
3757	CLSTN1	1.555	0.734	0.929	0.091	0.150	0.635	calsyntenin 1	CLSTN1
3838	CNPY2	1.555	1.128	1.312	0.065	0.560	0.331	canopy FGF signaling regulator 2	CNPY2
1952	C10orf90	1.554	1.532	1.369	0.011	0.254	0.113	chromosome 10 open reading frame 90	C10orf90
15914	PCSK9	1.554	0.151	0.307	0.743	0.410	0.451	proprotein convertase subtilisin/kexin type 9	PCSK9
285	ADAM28	1.554	0.936	1.072	0.046	0.530	0.306	ADAM metallopeptidase domain 28	ADAM28
703	AMOTL2	1.553	0.780	0.996	0.495	0.648	0.990	angiomotin like 2	AMOTL2
17624	RARRES1	1.553	0.854	1.817	0.574	0.746	0.421	retinoic acid receptor responder 1	RARRES1
8622	IDS	1.551	0.411	0.649	0.411	0.219	0.218	iduronate 2-sulfatase	IDS
21986	TMX1	1.550	1.065	1.085	0.022	0.678	0.339	thioredoxin related transmembrane protein 1	TMX1
6501	FDPS	1.550	0.289	0.434	0.710	0.447	0.393	farnesyl diphosphate synthase	FDPS
13914	MTHFS	1.550	1.038	1.215	0.095	0.676	0.444	5,10-methenyltetrahydrofolate synthetase (5-formyltetrahydrofolate	MTHFS
3155	CDC7	1.549	1.594	1.430	0.255	0.111	0.051	cell division cycle 7	CDC7
23393	WNT3	1.549	0.877	0.992	0.064	0.182	0.978	Wnt family member 3	WNT3
9357	KDM5B	1.548	0.708	1.166	0.006	0.159	0.231	lysine demethylase 5B	KDM5B
17305	PTPN12	1.548	0.885	0.960	0.320	0.660	0.897	protein tyrosine phosphatase, non-receptor type 12	PTPN12
3824	CNNM4	1.548	0.679	0.874	0.364	0.411	0.637	cyclin and CBS domain divalent metal cation transport mediator 4	CNNM4
939	APCDD1L-AS1	1.548	0.661	1.260	0.114	0.125	0.022	APCDD1L antisense RNA 1 (head to head)	APCDD1L-AS1
5610	EMC1	1.548	1.094	1.493	0.224	0.687	0.290	ER membrane protein complex subunit 1	EMC1
15997	PDLIM7	1.548	1.133	1.503	0.169	0.536	0.191	PDZ and LIM domain 7	PDLIM7
9060	ITGB1	1.546	1.255	1.688	0.317	0.609	0.197	integrin subunit beta 1	ITGB1
12381	LOC400553	1.546	0.619	0.713	0.415	0.327	0.422	uncharacterized LOC400553	LOC400553
20569	SQSTM1	1.544	0.670	0.886	0.389	0.439	0.478	sequestosome 1	SQSTM1
15672	PAM16	1.544	2.059	1.965	0.047	0.028	0.249	presequence translocase-associated motor 16 homolog (S. cerevisiae	PAM16
5878	EWSR1	1.543	1.428	1.768	0.046	0.034	0.123	EWS RNA binding protein 1	EWSR1
2623	CAP2	1.543	0.849	0.853	0.296	0.746	0.815	CAP, adenylate cyclase-associated protein, 2 (yeast)	CAP2
8203	HIST3H2A	1.542	0.759	1.639	0.191	0.354	0.385	histone cluster 3, H2a	HIST3H2A
7929	HAS2-AS1	1.542	3.239	1.455	0.061	0.156	0.059	HAS2 antisense RNA 1	HAS2-AS1
21530	TICAM2	1.542	0.683	1.106	0.118	0.061	0.743	toll like receptor adaptor molecule 2	TICAM2
12925	MAFK	1.541	0.796	1.007	0.199	0.431	0.981	v-maf avian musculoaponeurotic fibrosarcoma oncogene homolog K	MAFK
6372	FASTKD1	1.541	1.188	1.377	0.075	0.113	0.170	FAST kinase domains 1	FASTKD1
698	AMMECR1L	1.541	0.914	1.001	0.036	0.168	0.991	AMMECR1 like	AMMECR1L
18151	RNF5P1	1.541	1.110	1.206	0.121	0.640	0.028	ring finger protein 5 pseudogene 1	RNF5P1
21012	SYT12	1.540	0.881	0.930	0.313	0.579	0.918	synaptotagmin 12	SYT12

16343	PKP1	1.540	0.722	1.245	0.468	0.571	0.583	plakophilin 1	PKP1
11078	LOC10012857	1.539	0.746	1.276	0.302	0.384	0.497	uncharacterized LOC100128573	LOC100128573
1693	BET1	1.539	1.024	1.298	0.260	0.921	0.338	Bet1 golgi vesicular membrane trafficking protein	BET1
402	ADIPOR2	1.539	0.725	0.877	0.136	0.187	0.465	adiponectin receptor 2	ADIPOR2
22124	TP53I13	1.539	1.132	1.151	0.118	0.473	0.583	tumor protein p53 inducible protein 13	TP53I13
19334	SLC25A13	1.539	1.082	1.206	0.030	0.569	0.167	solute carrier family 25 member 13	SLC25A13
19926	SNORA63	1.538	1.475	0.622	0.677	0.596	0.725	small nucleolar RNA, H/ACA box 63	SNORA63
4464	CYP1B1	1.538	0.309	0.636	0.038	0.052	0.011	cytochrome P450 family 1 subfamily B member 1	CYP1B1
5685	ENTPD6	1.538	1.324	1.568	0.098	0.447	0.011	ectonucleoside triphosphate diphosphohydrolase 6 (putative)	ENTPD6
22017	TNFRSF21	1.538	0.894	1.439	0.198	0.649	0.014	tumor necrosis factor receptor superfamily member 21	TNFRSF21
16324	PKDCC	1.537	0.874	1.537	0.152	0.708	0.178	protein kinase domain containing, cytoplasmic	PKDCC
16492	PLXNB1	1.537	0.538	0.760	0.175	0.030	0.411	plexin B1	PLXNB1
3963	COPG1	1.536	0.938	1.168	0.174	0.767	0.373	coatomer protein complex subunit gamma 1	COPG1
14689	NPEPL1	1.536	0.744	1.221	0.004	0.035	0.009	aminopeptidase-like 1	NPEPL1
22790	UBR1	1.536	0.717	0.872	0.069	0.093	0.465	ubiquitin protein ligase E3 component n-recognin 1	UBR1
9037	ITFG1	1.536	0.823	0.861	0.140	0.183	0.657	integrin alpha FG-GAP repeat containing 1	ITFG1
622	ALDOC	1.536	0.218	0.634	0.356	0.095	0.251	aldolase, fructose-bisphosphate C	ALDOC
6065	FAM160A1	1.536	0.572	0.821	0.322	0.253	0.236	family with sequence similarity 160 member A1	FAM160A1
7812	GTPBP10	1.534	0.570	0.863	0.341	0.239	0.569	GTP binding protein 10 (putative)	GTPBP10
1984	C11orf86	1.534	1.049	1.079	0.025	0.810	0.421	chromosome 11 open reading frame 86	C11orf86
22008	TNFRSF12A	1.533	1.847	1.226	0.442	0.329	0.637	tumor necrosis factor receptor superfamily member 12A	TNFRSF12A
4697	DDX18	1.533	0.998	1.156	0.014	0.986	0.106	DEAD-box helicase 18	DDX18
2805	CCDC144A	1.533	0.552	0.860	0.589	0.483	0.747	coiled-coil domain containing 144A	CCDC144A
3749	CLPTM1	1.533	1.081	1.133	0.131	0.722	0.191	cleft lip and palate associated transmembrane protein 1	CLPTM1
8767	IL12RB1	1.533	0.745	1.087	0.170	0.269	0.321	interleukin 12 receptor subunit beta 1	IL12RB1
5612	EMC2	1.532	0.749	0.982	0.015	0.020	0.701	ER membrane protein complex subunit 2	EMC2
8249	HM13	1.532	0.890	1.231	0.362	0.765	0.255	histocompatibility (minor) 13	HM13
19358	SLC25A32	1.532	0.914	1.122	0.139	0.512	0.560	solute carrier family 25 member 32	SLC25A32
23349	WFDC10B	1.531	0.688	1.118	0.092	0.168	0.615	WAP four-disulfide core domain 10B	WFDC10B
5321	DYM	1.531	0.655	0.915	0.133	0.084	0.615	dymeclin	DYM
15643	PAG1	1.531	0.628	0.927	0.211	0.212	0.793	phosphoprotein membrane anchor with glycosphingolipid microdom	PAG1
1039	ARFGAP3	1.530	0.734	1.019	0.206	0.296	0.850	ADP ribosylation factor GTPase activating protein 3	ARFGAP3
8837	IL4R	1.529	1.277	1.710	0.046	0.230	0.304	interleukin 4 receptor	IL4R
4659	DCUN1D5	1.529	1.017	1.215	0.012	0.788	0.161	defective in cullin neddylation 1 domain containing 5	DCUN1D5
22555	TTLL4	1.528	0.986	1.320	0.083	0.907	0.409	tubulin tyrosine ligase like 4	TTLL4
1824	BRCA2	1.528	1.273	1.056	0.358	0.480	0.747	breast cancer 2	BRCA2
5747	EPS8	1.528	0.634	0.905	0.277	0.298	0.287	epidermal growth factor receptor pathway substrate 8	EPS8
22712	UBAP1	1.527	0.854	1.211	0.322	0.611	0.315	ubiquitin associated protein 1	UBAP1
4018	COX7B	1.527	1.570	1.643	0.044	0.085	0.181	cytochrome c oxidase subunit 7B	COX7B
20706	ST6GALNAC4	1.527	1.324	2.037	0.129	0.346	0.282	ST6 N-acetylgalactosaminide alpha-2,6-sialyltransferase 4	ST6GALNAC4
20898	SULT1C4	1.527	0.656	0.960	0.316	0.312	0.627	sulfotransferase family 1C member 4	SULT1C4
16744	PPIL1	1.526	1.866	1.528	0.126	0.095	0.127	peptidylprolyl isomerase like 1	PPIL1
5401	EDEM1	1.526	0.795	0.791	0.180	0.124	0.497	ER degradation enhancing alpha-mannosidase like protein 1	EDEM1
1428	ATP6V0B	1.526	1.280	1.251	0.270	0.532	0.356	ATPase H+ transporting V0 subunit b	ATP6V0B
6945	FZD7	1.526	0.333	0.559	0.053	0.016	0.002	frizzled class receptor 7	FZD7
19556	SLC5A6	1.526	1.218	1.646	0.057	0.422	0.111	solute carrier family 5 member 6	SLC5A6
13762	MRPL44	1.525	0.934	1.414	0.119	0.703	0.329	mitochondrial ribosomal protein L44	MRPL44
1032	ARF1	1.525	1.134	1.340	0.176	0.516	0.018	ADP ribosylation factor 1	ARF1
19443	SLC35A2	1.524	1.106	1.519	0.075	0.658	0.092	solute carrier family 35 member A2	SLC35A2
17682	RBBP7	1.524	1.232	1.358	0.092	0.158	0.130	retinoblastoma binding protein 7	RBBP7
3744	CLPB	1.524	1.238	1.776	0.282	0.395	0.210	ClpB homolog, mitochondrial AAA ATPase chaperonin	CLPB
22812	UCHL1	1.524	1.416	1.696	0.685	0.755	0.609	ubiquitin C-terminal hydrolase L1	UCHL1
16420	PLEKHA3	1.524	0.906	0.889	0.118	0.524	0.280	pleckstrin homology domain containing A3	PLEKHA3
23508	YIF1A	1.522	1.111	1.494	0.125	0.634	0.213	Yip1 interacting factor homolog A, membrane trafficking protein	YIF1A
21207	TBL1X	1.522	0.845	1.227	0.007	0.387	0.512	transducin (beta)-like 1X-linked	TBL1X
2672	CASC15	1.522	1.207	1.282	0.267	0.578	0.442	cancer susceptibility candidate 15 (non-protein coding)	CASC15
2806	CCDC144B	1.522	0.516	0.740	0.625	0.513	0.611	coiled-coil domain containing 144B (pseudogene)	CCDC144B
20561	SPTSSA	1.521	1.001	1.047	0.140	0.991	0.846	serine palmitoyltransferase small subunit A	SPTSSA
18868	SEMA6A-AS1	1.519	0.853	1.129	0.374	0.671	0.607	SEMA6A antisense RNA 1	SEMA6A-AS1
11231	LOC10028951	1.519	0.790	1.161	0.083	0.175	0.389	uncharacterized LOC100289511	LOC100289511
16645	PON2	1.519	1.047	1.302	0.409	0.904	0.260	paraoxonase 2	PON2
21192	TBC1D7	1.519	1.172	1.740	0.154	0.375	0.012	TBC1 domain family member 7	TBC1D7
21045	TAB3	1.518	0.646	0.839	0.283	0.242	0.466	TGF-beta activated kinase 1/MAP3K7 binding protein 3	TAB3
19429	SLC30A4	1.517	0.546	0.773	0.283	0.172	0.426	solute carrier family 30 member 4	SLC30A4
1029	ARCN1	1.516	0.922	1.127	0.230	0.697	0.715	archain 1	ARCN1
18816	SEC22B	1.516	0.924	1.357	0.126	0.525	0.390	SEC22 homolog B, vesicle trafficking protein (gene/pseudogene)	SEC22B
8626	IER3IP1	1.516	1.243	1.088	0.008	0.010	0.152	immediate early response 3 interacting protein 1	IER3IP1
11579	LOC10192747	1.516	1.055	1.047	0.003	0.506	0.691	uncharacterized LOC101927476	LOC101927476
17148	PSAP	1.516	0.414	0.664	0.020	0.003	0.092	prosaposin	PSAP
18620	SATB1	1.515	0.380	0.970	0.253	0.077	0.877	SATB homeobox 1	SATB1
17825	RELL1	1.515	0.988	1.023	0.170	0.942	0.905	RELT like 1	RELL1
16549	PNPLA3	1.515	0.273	0.447	0.738	0.431	0.537	patatin like phospholipase domain containing 3	PNPLA3
19535	SLC4A2	1.515	1.339	1.351	0.176	0.357	0.164	solute carrier family 4 member 2	SLC4A2
8522	HSPB8	1.515	0.577	0.625	0.033	0.000	0.065	heat shock protein family B (small) member 8	HSPB8
13638	MORC2-AS1	1.514	0.715	1.107	0.076	0.007	0.693	MORC2 antisense RNA 1	MORC2-AS1
16202	PI4K2A	1.514	0.629	0.751	0.565	0.509	0.477	phosphatidylinositol 4-kinase type 2 alpha	PI4K2A
23562	ZBED6	1.514	0.654	1.039	0.161	0.172	0.864	zinc finger BED-type containing 6	ZBED6
15606	P4HTM	1.514	0.977	1.127	0.041	0.917	0.517	prolyl 4-hydroxylase, transmembrane	P4HTM
19014	SFT2D1	1.513	1.110	1.272	0.019	0.154	0.061	SFT2 domain containing 1	SFT2D1
22003	TNFRSF10B	1.513	0.672	0.879	0.175	0.131	0.618	tumor necrosis factor receptor superfamily member 10b	TNFRSF10B
18109	RNF182	1.513	1.072	1.025	0.244	0.789	0.339	ring finger protein 182	RNF182
5376	ECE2	1.512	2.298	2.424	0.117	0.035	0.287	endothelin converting enzyme 2	ECE2
5302	DUSP3	1.512	0.582	0.740	0.325	0.206	0.467	dual specificity phosphatase 3	DUSP3
17611	RAPGEF2	1.512	0.732	0.775	0.402	0.427	0.594	Rap guanine nucleotide exchange factor 2	RAPGEF2
7718	GRPEL1	1.511	1.200	1.313	0.082	0.435	0.015	GrpE like 1, mitochondrial	GRPEL1
14416	NEMP2	1.511	1.289	1.220	0.053	0.239	0.251	nuclear envelope integral membrane protein 2	NEMP2
6914	FUT4	1.511	0.606	0.774	0.235	0.186	0.195	fucosyltransferase 4	FUT4
5391	ECT2	1.510	2.920	1.701	0.492	0.037	0.356	epithelial cell transforming 2	ECT2
17475	RAB32	1.510	1.011	0.989	0.200	0.957	0.841	RAB32, member RAS oncogene family	RAB32
13599	MMP7	1.510	0.998	1.124	0.071	0.986	0.195	matrix metallopeptidase 7	MMP7
20943	SV2A	1.509	0.820	1.164	0.543	0.718	0.657	synaptic vesicle glycoprotein 2A	SV2A

8920	INSR	1.509	0.533	0.902	0.032	0.014	0.696	insulin receptor	INSR
21913	TMEM71	1.508	1.098	1.041	0.257	0.788	0.862	transmembrane protein 71	TMEM71
16706	PPAPDC1B	1.508	0.811	1.063	0.130	0.389	0.798	.	PPAPDC1B
19613	SLC9A8	1.507	0.756	0.978	0.386	0.518	0.909	solute carrier family 9 member A8	SLC9A8
6715	FMNL2	1.507	0.775	0.815	0.508	0.555	0.723	formin like 2	FMNL2
6424	FBXO2	1.506	0.408	0.447	0.366	0.045	0.292	F-box protein 2	FBXO2
5842	ETAA1	1.506	1.013	0.958	0.201	0.957	0.594	Ewing tumor associated antigen 1	ETAA1
21631	TM9SF1	1.506	0.828	0.930	0.017	0.140	0.551	transmembrane 9 superfamily member 1	TM9SF1
7890	H3F3B	1.506	1.079	1.352	0.010	0.156	0.032	H3 histone, family 3B (H3.3B)	H3F3B
10183	LINC00312	1.505	0.345	0.711	0.670	0.403	0.601	long intergenic non-protein coding RNA 312	LINC00312
2240	C20orf24	1.505	1.344	1.257	0.010	0.354	0.048	chromosome 20 open reading frame 24	C20orf24
6577	FGFR1OP	1.505	1.447	1.128	0.051	0.024	0.479	FGFR1 oncogene partner	FGFR1OP
6376	FAT1	1.505	0.593	0.618	0.111	0.145	0.141	FAT atypical cadherin 1	FAT1
9830	KRTDAP	1.504	0.612	0.920	0.170	0.123	0.423	keratinocyte differentiation associated protein	KRTDAP
20267	SNX25	1.504	0.804	1.033	0.076	0.045	0.852	sorting nexin 25	SNX25
5592	ELN	1.504	8.300	1.946	0.674	0.220	0.596	elastin	ELN
16233	PIGO	1.504	1.081	1.114	0.078	0.432	0.365	phosphatidylinositol glycan anchor biosynthesis class O	PIGO
18615	SASH1	1.504	0.662	1.054	0.119	0.173	0.789	SAM and SH3 domain containing 1	SASH1
14405	NEK7	1.504	0.681	0.912	0.040	0.020	0.840	NIMA related kinase 7	NEK7
3906	COL24A1	1.504	0.586	0.803	0.276	0.201	0.148	collagen type XXIV alpha 1	COL24A1
21432	TGDS	1.504	0.852	1.232	0.076	0.251	0.224	TDP-glucose 4,6-dehydratase	TGDS
12647	LRFN4	1.503	0.963	1.311	0.081	0.900	0.373	leucine rich repeat and fibronectin type III domain containing 4	LRFN4
8693	IFT74	1.503	0.929	1.006	0.061	0.526	0.951	intraflagellar transport 74	IFT74
20844	STX12	1.503	0.721	0.903	0.083	0.079	0.742	syntaxin 12	STX12
23151	VNN2	1.503	1.139	1.092	0.007	0.737	0.003	vanin 2	VNN2
17766	RBPMS2	1.502	0.625	0.747	0.518	0.448	0.455	RNA binding protein with multiple splicing 2	RBPMS2
4457	CYP11A1	1.502	1.005	1.292	0.506	0.991	0.381	cytochrome P450 family 11 subfamily A member 1	CYP11A1
1302	ASS1	1.501	0.795	1.166	0.359	0.523	0.625	argininosuccinate synthase 1	ASS1
22870	ULBP2	1.501	1.247	1.050	0.401	0.506	0.913	UL16 binding protein 2	ULBP2
9348	KDM3A	1.501	0.421	0.731	0.051	0.013	0.158	lysine demethylase 3A	KDM3A
7575	GPR161	1.501	1.291	1.102	0.168	0.090	0.741	G protein-coupled receptor 161	GPR161
21871	TMEM38B	1.500	0.831	0.645	0.500	0.727	0.049	transmembrane protein 38B	TMEM38B
5448	EFHC2	1.500	0.984	1.117	0.077	0.943	0.221	EF-hand domain containing 2	EFHC2

Filter: 24424		Fold change			p-value				Gene information
ID	Gene symbol	IL1b_group /Contorl_group	IL1b_PRP_group /IL1b_group	PRP_group /Contorl_group	IL1b_group /Contorl_group	IL1b_PRP_group /IL1b_group	PRP_group /Contorl_group	Description	NCBI search
18196	RNU86	1.005	9487.588	1.001	0.713	0.423	0.943	.	RNU86
20179	SNORD68	1.005	1337.435	1.001	0.713	0.423	0.943	small nucleolar RNA, C/D box 68	SNORD68
3127	CDC20	0.670	42.828	11.491	0.254	0.002	0.011	cell division cycle 20	CDC20
22723	UBE2C	0.862	42.338	8.463	0.699	0.000	0.001	ubiquitin conjugating enzyme E2 C	UBE2C
2966	CCNB1	0.635	35.750	7.493	0.349	0.008	0.003	cyclin B1	CCNB1
1727	BIRC5	0.627	32.789	9.871	0.198	0.042	0.134	baculoviral IAP repeat containing 5	BIRC5
22100	TOP2A	0.822	26.118	4.694	0.698	0.094	0.021	topoisomerase (DNA) II alpha 170kDa	TOP2A
15991	PDK4	0.622	25.914	5.751	0.409	0.338	0.433	pyruvate dehydrogenase kinase 4	PDK4
8604	ID1	3.604	24.939	75.872	0.112	0.182	0.405	inhibitor of DNA binding 1, HLH protein	ID1
2968	CCNB2	0.524	24.514	4.884	0.227	0.041	0.003	cyclin B2	CCNB2
19911	SNORA52	1.002	23.597	1.000	0.714	0.020	0.943	small nucleolar RNA, H/ACA box 52	SNORA52
22373	TROAP	0.856	23.305	6.197	0.501	0.018	0.036	trophinin associated protein	TROAP
16463	PLK1	0.716	22.988	5.398	0.001	0.002	0.028	polo-like kinase 1	PLK1
18420	RRM2	0.832	22.979	4.049	0.716	0.007	0.084	ribonucleotide reductase regulatory subunit M2	RRM2
19956	SNORA7B	0.150	22.826	0.677	0.423	0.004	0.778	small nucleolar RNA, H/ACA box 7B	SNORA7B
13522	MKI67	0.644	22.728	4.928	0.442	0.011	0.031	marker of proliferation Ki-67	MKI67
846	ANLN	0.679	22.374	5.347	0.449	0.017	0.150	anillin actin binding protein	ANLN
21579	TK1	0.526	21.058	8.254	0.081	0.004	0.010	thymidine kinase 1	TK1
4978	DLGAP5	0.611	20.736	4.548	0.168	0.033	0.005	discs large homolog associated protein 5	DLGAP5
3197	CDK1	0.774	20.525	3.085	0.406	0.007	0.023	cyclin-dependent kinase 1	CDK1
9486	KIF2C	0.902	19.568	5.181	0.510	0.013	0.026	kinesin family member 2C	KIF2C
22191	TPX2	0.713	19.164	5.427	0.378	0.019	0.006	TPX2, microtubule-associated	TPX2
20808	STMN1	0.372	18.981	3.813	0.190	0.010	0.015	stathmin 1	STMN1
17350	PTTG1	1.217	18.853	8.542	0.122	0.022	0.054	pituitary tumor-transforming 1	PTTG1
14947	NUSAP1	0.771	18.307	4.114	0.568	0.016	0.016	nucleolar and spindle associated protein 1	NUSAP1
1490	AURKB	0.901	18.261	5.751	0.545	0.006	0.025	aurora kinase B	AURKB
19963	SNORA9	1.002	17.951	1.000	0.714	0.038	0.943	small nucleolar RNA, H/ACA box 9	SNORA9
15769	PBK	0.707	17.774	4.470	0.323	0.005	0.022	PDZ binding kinase	PBK
8977	IQGAP3	0.644	17.654	4.499	0.276	0.003	0.004	IQ motif containing GTPase activating protein 3	IQGAP3
3368	CEP55	0.743	17.471	5.177	0.370	0.001	0.134	centrosomal protein 55	CEP55
6796	FOXM1	0.443	17.345	4.589	0.189	0.020	0.010	forkhead box M1	FOXM1
24416	ZWINT	0.668	17.345	3.436	0.365	0.002	0.013	ZW10 interacting kinetochore protein	ZWINT
9472	KIF20A	0.612	17.216	5.239	0.366	0.000	0.143	kinesin family member 20A	KIF20A
16894	PRC1	0.756	16.320	4.316	0.485	0.005	0.028	protein regulator of cytokinesis 1	PRC1
3329	CENPF	0.800	15.986	3.719	0.650	0.038	0.029	centromere protein F	CENPF
17058	PRR11	0.918	15.650	6.804	0.492	0.055	0.099	proline rich 11	PRR11
1487	AURKA	1.015	15.539	4.010	0.958	0.000	0.072	aurora kinase A	AURKA
9500	KIFC1	0.651	15.394	3.514	0.315	0.004	0.009	kinesin family member C1	KIFC1
3163	CDCA8	0.706	15.391	4.288	0.072	0.008	0.000	cell division cycle associated 8	CDCA8
14400	NEK2	0.854	15.216	4.148	0.387	0.059	0.021	NIMA related kinase 2	NEK2
19112	SHCBP1	0.695	15.172	3.769	0.353	0.006	0.033	SHC binding and spindle associated 1	SHCBP1
6255	FAM64A	0.567	15.124	3.453	0.231	0.000	0.074	family with sequence similarity 64 member A	FAM64A
1913	BUB1	0.716	14.994	3.319	0.285	0.041	0.034	BUB1 mitotic checkpoint serine/threonine kinase	BUB1
9384	KIAA0101	0.471	14.711	4.734	0.151	0.003	0.013	KIAA0101	KIAA0101
8282	HMMR	0.944	14.609	3.762	0.540	0.051	0.041	hyaluronan mediated motility receptor	HMMR
21051	TACC3	0.918	14.376	4.643	0.745	0.006	0.021	transforming acidic coiled-coil containing protein 3	TACC3
14912	NUF2	0.613	14.080	3.045	0.262	0.015	0.043	NUF2, NDC80 kinetochore complex component	NUF2
3335	CENPM	1.022	13.976	6.955	0.859	0.000	0.040	centromere protein M	CENPM
16335	PKMYT1	0.653	13.923	3.576	0.128	0.000	0.002	protein kinase, membrane associated tyrosine/threonine 1	PKMYT1
20371	SPAG5	0.810	13.874	3.077	0.376	0.000	0.053	sperm associated antigen 5	SPAG5
7819	GTSE1	0.797	13.452	3.850	0.466	0.003	0.012	G2 and S-phase expressed 1	GTSE1
18681	SCARNA4	0.097	13.436	0.097	0.423	0.423	0.423	small Cajal body-specific RNA 4	SCARNA4
9477	KIF23	0.603	13.233	2.977	0.230	0.012	0.059	kinesin family member 23	KIF23
3323	CENPA	0.719	13.098	3.676	0.043	0.031	0.024	centromere protein A	CENPA
22680	TYMS	0.385	12.980	2.825	0.058	0.000	0.108	thymidylate synthetase	TYMS
14012	MYBL2	0.759	12.748	5.743	0.136	0.000	0.000	MYB proto-oncogene like 2	MYBL2
3160	CDCA5	0.761	12.528	3.844	0.254	0.000	0.069	cell division cycle associated 5	CDCA5
1914	BUB1B	0.737	12.479	3.253	0.349	0.006	0.014	BUB1 mitotic checkpoint serine/threonine kinase B	BUB1B
8210	HJURP	0.811	12.395	2.917	0.384	0.002	0.011	Holliday junction recognition protein	HJURP
19879	SNORA24	1.001	12.224	1.000	0.714	0.423	0.943	small nucleolar RNA, H/ACA box 24	SNORA24
3158	CDCA3	0.859	11.860	3.874	0.464	0.003	0.022	cell division cycle associated 3	CDCA3
1297	ASPM	0.695	11.081	2.582	0.353	0.082	0.065	abnormal spindle microtubule assembly	ASPM
2965	CCNA2	1.010	10.966	4.531	0.967	0.024	0.049	cyclin A2	CCNA2
16559	POC1A	0.716	10.832	3.512	0.016	0.005	0.075	POC1 centriolar protein A	POC1A
20440	SPC24	0.519	10.524	3.119	0.018	0.021	0.138	SPC24, NDC80 kinetochore complex component	SPC24
14304	NDC80	0.918	10.474	2.895	0.201	0.012	0.022	NDC80 kinetochore complex component	NDC80
8712	IGFBP1	0.983	10.403	2.356	0.978	0.238	0.055	insulin like growth factor binding protein 1	IGFBP1
4809	DEPDC1	0.997	10.138	2.923	0.988	0.042	0.036	DEP domain containing 1	DEPDC1
3345	CENPW	0.755	9.943	3.403	0.072	0.018	0.030	centromere protein W	CENPW
6297	FAM83D	0.728	9.940	2.599	0.351	0.002	0.061	family with sequence similarity 83 member D	FAM83D
9467	KIF18B	0.940	9.671	3.276	0.790	0.000	0.008	kinesin family member 18B	KIF18B
968	APOBEC3B	0.891	9.607	3.300	0.607	0.031	0.063	apolipoprotein B mRNA editing enzyme catalytic subunit 3B	APOBEC3B
14258	NCAPG	0.856	9.550	2.559	0.449	0.001	0.041	non-SMC condensin I complex subunit G	NCAPG
1272	ASF1B	0.836	9.485	3.028	0.569	0.001	0.111	anti-silencing function 1B histone chaperone	ASF1B
3132	CDC25C	0.857	9.231	2.552	0.055	0.127	0.189	cell division cycle 25C	CDC25C
12909	MAD2L1	0.967	9.230	2.993	0.888	0.010	0.006	MAD2 mitotic arrest deficient-like 1 (yeast)	MAD2L1
11035	LMNB1	0.768	9.160	3.720	0.246	0.017	0.007	lamin B1	LMNB1
23315	WDR62	0.854	9.127	2.623	0.024	0.001	0.000	WD repeat domain 62	WDR62
9490	KIF4A	1.042	9.088	3.585	0.549	0.019	0.001	kinesin family member 4A	KIF4A
22758	UBE2S	1.719	9.079	4.027	0.088	0.010	0.116	ubiquitin conjugating enzyme E2 S	UBE2S
6754	FOS	0.660	9.034	4.938	0.030	0.411	0.400	Fos proto-oncogene, AP-1 trancription factor subunit	FOS
9476	KIF22	0.798	8.982	2.871	0.016	0.003	0.067	kinesin family member 22	KIF22
22546	TTK	0.979	8.959	2.905	0.921	0.018	0.035	TTK protein kinase	TTK
3152	CDC45	1.040	8.877	2.824	0.577	0.003	0.043	cell division cycle 45	CDC45

9458	KIF11	0.832	8.589	2.536	0.555	0.054	0.033	kinesin family member 11	KIF11
18604	SAPCD2	0.750	8.582	3.362	0.165	0.003	0.006	suppressor APC domain containing 2	SAPCD2
22347	TRIP13	0.793	8.502	2.994	0.431	0.002	0.065	thyroid hormone receptor interactor 13	TRIP13
18049	RNASEH2A	0.882	8.419	3.485	0.210	0.000	0.041	ribonuclease H2 subunit A	RNASEH2A
5592	ELN	1.504	8.300	1.946	0.674	0.220	0.596	elastin	ELN
19202	SKA3	0.886	8.272	2.737	0.568	0.013	0.035	spindle and kinetochore associated complex subunit 3	SKA3
19200	SKA1	0.853	8.174	2.519	0.082	0.010	0.044	spindle and kinetochore associated complex subunit 1	SKA1
19875	SNORA20	1.001	8.155	1.000	0.714	0.423	0.943	small nucleolar RNA, H/ACA box 20	SNORA20
3634	CKS2	1.151	8.154	2.893	0.754	0.001	0.149	CDC28 protein kinase regulatory subunit 2	CKS2
22759	UBE2T	0.969	8.102	2.347	0.942	0.009	0.256	ubiquitin conjugating enzyme E2 T	UBE2T
3261	CDT1	0.769	8.010	3.079	0.047	0.000	0.084	chromatin licensing and DNA replication factor 1	CDT1
3330	CENPH	0.662	7.982	2.908	0.013	0.058	0.000	centromere protein H	CENPH
22864	UHRF1	0.712	7.873	2.707	0.361	0.001	0.015	ubiquitin like with PHD and ring finger domains 1	UHRF1
3621	CKAP2	0.590	7.835	1.846	0.271	0.027	0.226	cytoskeleton associated protein 2	CKAP2
19890	SNORA33	1.002	7.785	10.229	0.714	0.423	0.423	small nucleolar RNA, H/ACA box 33	SNORA33
19869	SNORA15	0.112	7.771	1.681	0.423	0.423	0.571	small nucleolar RNA, H/ACA box 15	SNORA15
9713	KRT7	1.655	7.393	4.090	0.007	0.172	0.012	keratin 7	KRT7
3242	CDKN3	2.647	7.375	6.385	0.367	0.159	0.112	cyclin-dependent kinase inhibitor 3	CDKN3
20441	SPC25	1.009	7.373	2.279	0.962	0.004	0.001	SPC25, NDC80 kinetochore complex component	SPC25
17534	RACGAP1	0.779	7.297	2.443	0.588	0.002	0.140	Rac GTPase activating protein 1	RACGAP1
6755	FOSB	0.954	7.198	3.392	0.612	0.410	0.404	FosB proto-oncogene, AP-1 trancription factor subunit	FOSB
1053	ARHGAP11A	0.811	7.193	2.446	0.263	0.146	0.121	Rho GTPase activating protein 11A	ARHGAP11A
22599	TUBA1B	0.679	7.135	2.597	0.049	0.004	0.183	tubulin alpha 1b	TUBA1B
3584	CHTF18	0.653	7.040	2.054	0.006	0.025	0.032	chromosome transmission fidelity factor 18	CHTF18
3341	CENPU	0.826	6.996	1.968	0.258	0.023	0.009	centromere protein U	CENPU
13304	MELK	0.690	6.915	1.974	0.492	0.002	0.268	maternal embryonic leucine zipper kinase	MELK
19959	SNORA80B	1.001	6.885	1.000	0.714	0.423	0.943	small nucleolar RNA, H/ACA box 80B	SNORA80B
13608	MND1	0.896	6.881	2.614	0.132	0.001	0.000	meiotic nuclear divisions 1	MND1
9649	KPNA2	1.292	6.849	2.752	0.450	0.005	0.013	karyopherin subunit alpha 2	KPNA2
13199	MCM5	0.849	6.804	2.971	0.349	0.010	0.006	minichromosome maintenance complex component 5	MCM5
7254	GINS2	0.871	6.757	2.649	0.643	0.019	0.010	GINS complex subunit 2	GINS2
7881	H2AFX	1.082	6.739	2.832	0.419	0.001	0.017	H2A histone family member X	H2AFX
2862	CCDC34	0.717	6.730	2.493	0.081	0.009	0.150	coiled-coil domain containing 34	CCDC34
17233	PSRC1	0.793	6.698	2.426	0.184	0.018	0.038	proline and serine rich coiled-coil 1	PSRC1
6352	FANCI	0.881	6.624	2.017	0.324	0.005	0.015	Fanconi anemia complementation group I	FANCI
9646	KNSTRN	0.979	6.595	2.209	0.888	0.006	0.133	kinetochore-localized astrin/SPAG5 binding protein	KNSTRN
2683	CASC5	0.829	6.456	2.371	0.248	0.071	0.072	cancer susceptibility candidate 5	CASC5
3328	CENPE	0.905	6.349	2.232	0.684	0.028	0.064	centromere protein E	CENPE
19047	SGOL2	0.805	6.343	1.977	0.376	0.059	0.133	.	SGOL2
17808	RECQL4	0.775	6.297	2.471	0.097	0.000	0.081	RecQ like helicase 4	RECQL4
9473	KIF20B	0.871	6.260	1.752	0.432	0.026	0.039	kinesin family member 20B	KIF20B
3333	CENPK	0.993	6.205	2.553	0.969	0.028	0.099	centromere protein K	CENPK
3633	CKS1B	0.771	6.024	1.738	0.289	0.019	0.114	CDC28 protein kinase regulatory subunit 1B	CKS1B
18766	SDC1	0.315	6.011	1.729	0.233	0.139	0.476	syndecan 1	SDC1
3285	CEBPA	1.348	5.991	1.315	0.216	0.379	0.583	CCAAT/enhancer binding protein alpha	CEBPA
5349	E2F1	0.961	5.955	1.798	0.900	0.000	0.115	E2F transcription factor 1	E2F1
3622	CKAP2L	1.282	5.944	2.392	0.559	0.007	0.127	cytoskeleton associated protein 2 like	CKAP2L
21268	TCF19	0.546	5.784	1.690	0.127	0.005	0.222	transcription factor 19	TCF19
8262	HMGB2	0.490	5.778	1.731	0.147	0.022	0.149	high mobility group box 2	HMGB2
8607	ID3	3.437	5.720	13.304	0.047	0.043	0.346	inhibitor of DNA binding 3, HLH protein	ID3
14259	NCAPG2	0.978	5.674	2.146	0.919	0.043	0.052	non-SMC condensin II complex subunit G2	NCAPG2
14429	NEURL1B	0.661	5.627	2.155	0.060	0.019	0.008	neuralized E3 ubiquitin protein ligase 1B	NEURL1B
13194	MCM2	0.719	5.517	1.780	0.031	0.012	0.011	minichromosome maintenance complex component 2	MCM2
22600	TUBA1C	0.938	5.487	2.140	0.497	0.028	0.079	tubulin alpha 1c	TUBA1C
17813	REEP4	0.754	5.411	1.410	0.252	0.008	0.197	receptor accessory protein 4	REEP4
14886	NUDT1	0.493	5.403	2.621	0.112	0.087	0.249	nudix (nucleoside diphosphate linked moiety X)-type motif 1	NUDT1
9462	KIF14	0.961	5.271	2.017	0.739	0.066	0.016	kinesin family member 14	KIF14
19909	SNORA49	1.001	5.231	1.000	0.714	0.423	0.943	small nucleolar RNA, H/ACA box 49	SNORA49
22615	TUBB4B	1.111	5.213	2.405	0.144	0.030	0.084	tubulin beta 4B class IVb	TUBB4B
2497	CA2	3.292	5.200	6.420	0.351	0.101	0.382	carbonic anhydrase 2	CA2
9463	KIF15	0.758	5.194	1.830	0.233	0.026	0.034	kinesin family member 15	KIF15
22825	UCP2	0.748	5.178	2.934	0.609	0.019	0.181	uncoupling protein 2	UCP2
19878	SNORA23	0.295	5.177	0.804	0.423	0.219	0.842	small nucleolar RNA, H/ACA box 23	SNORA23
5826	ESPL1	0.959	5.160	2.200	0.784	0.012	0.019	extra spindle pole bodies like 1, separase	ESPL1
8272	HMGN2	0.793	5.158	2.721	0.413	0.013	0.121	high mobility group nucleosomal binding domain 2	HMGN2
2978	CCNF	0.941	5.128	2.418	0.690	0.008	0.020	cyclin F	CCNF
19873	SNORA18	0.767	5.119	0.650	0.853	0.089	0.769	small nucleolar RNA, H/ACA box 18	SNORA18
7928	HAS2	4.254	5.115	3.921	0.004	0.130	0.161	hyaluronan synthase 2	HAS2
17545	RAD51	0.734	5.097	1.626	0.279	0.008	0.081	RAD51 recombinase	RAD51
15033	OIP5	0.789	5.059	1.741	0.301	0.009	0.082	Opa interacting protein 5	OIP5
19940	SNORA71A	1.001	5.050	1.000	0.714	0.423	0.943	small nucleolar RNA, H/ACA box 71A	SNORA71A
3157	CDCA2	0.896	5.041	1.816	0.690	0.035	0.022	cell division cycle associated 2	CDCA2
16466	PLK4	0.847	5.033	1.637	0.415	0.012	0.127	polo like kinase 4	PLK4
4401	CXCL5	105.394	5.025	1.918	0.348	0.091	0.346	C-X-C motif chemokine ligand 5	CXCL5
11036	LMNB2	0.901	5.001	2.425	0.320	0.000	0.004	lamin B2	LMNB2
16895	PRC1-AS1	0.937	4.967	1.881	0.769	0.001	0.125	PRC1 antisense RNA 1	PRC1-AS1
17555	RAD54L	0.864	4.915	1.729	0.247	0.010	0.017	RAD54-like (S. cerevisiae)	RAD54L
2032	C14orf80	0.852	4.881	1.802	0.124	0.001	0.088	chromosome 14 open reading frame 80	C14orf80
3331	CENPI	0.794	4.880	1.535	0.256	0.023	0.113	centromere protein I	CENPI
5822	ESCO2	0.821	4.872	1.397	0.172	0.005	0.159	establishment of sister chromatid cohesion N-acetyltransferase 2	ESCO2
15487	ORC6	1.139	4.869	1.742	0.562	0.040	0.386	origin recognition complex, subunit 6	ORC6
7884	H2AFZ	1.262	4.852	2.719	0.349	0.001	0.040	H2A histone family member Z	H2AFZ
9466	KIF18A	1.220	4.837	1.741	0.200	0.020	0.054	kinesin family member 18A	KIF18A
3336	CENPN	1.059	4.821	2.383	0.604	0.011	0.007	centromere protein N	CENPN
5957	FABP5	0.169	4.780	1.340	0.390	0.131	0.799	fatty acid binding protein 5	FABP5
16074	PF4V1	3.714	4.747	1.802	0.088	0.007	0.378	platelet factor 4 variant 1	PF4V1
7256	GINS4	0.833	4.723	1.941	0.251	0.001	0.009	GINS complex subunit 4	GINS4
5275	DTYMK	0.808	4.705	2.400	0.243	0.001	0.017	deoxythymidylate kinase	DTYMK
9726	KRT81	1.080	4.643	1.152	0.220	0.100	0.420	keratin 81	KRT81
10065	LIG1	0.678	4.632	1.871	0.059	0.007	0.007	DNA ligase 1	LIG1
14260	NCAPH	0.905	4.632	1.789	0.523	0.008	0.081	non-SMC condensin I complex subunit H	NCAPH

6347	FANCD2	1.164	4.603	2.054	0.083	0.016	0.012	Fanconi anemia complementation group D2	FANCD2
9435	KIAA1524	0.828	4.509	1.482	0.319	0.022	0.253	KIAA1524	KIAA1524
3471	CHAF1A	1.076	4.492	2.075	0.280	0.000	0.157	chromatin assembly factor 1 subunit A	CHAF1A
14396	NEIL3	0.766	4.475	1.533	0.020	0.003	0.338	nei like DNA glycosylase 3	NEIL3
3616	CIT	0.807	4.446	1.758	0.585	0.066	0.144	citron rho-interacting serine/threonine kinase	CIT
17885	RFX8	0.517	4.438	1.784	0.391	0.074	0.285	RFX family member 8, lacking RFX DNA binding domain	RFX8
19939	SNORA70G	1.001	4.428	1.000	0.714	0.423	0.943	small nucleolar RNA, H/ACA box 70G	SNORA70G
8873	INCENP	0.984	4.424	1.624	0.594	0.020	0.008	inner centromere protein	INCENP
5882	EXO1	0.983	4.401	1.690	0.940	0.002	0.071	exonuclease 1	EXO1
19705	SMC4	0.779	4.299	1.452	0.401	0.046	0.190	structural maintenance of chromosomes 4	SMC4
7253	GINS1	0.864	4.243	1.564	0.331	0.029	0.044	GINS complex subunit 1	GINS1
22210	TRAIP	0.728	4.231	1.415	0.083	0.010	0.050	TRAF interacting protein	TRAIP
13577	MMP10	7.099	4.225	9.648	0.000	0.372	0.408	matrix metallopeptidase 10	MMP10
19767	SMOX	3.132	4.223	6.400	0.031	0.081	0.415	spermine oxidase	SMOX
1823	BRCA1	0.808	4.205	1.641	0.195	0.004	0.014	breast cancer 1	BRCA1
5997	FAM111B	0.970	4.187	1.395	0.890	0.042	0.308	family with sequence similarity 111 member B	FAM111B
7370	GMNN	1.892	4.171	4.469	0.055	0.066	0.299	geminin, DNA replication inhibitor	GMNN
4811	DEPDC1B	0.946	4.167	1.709	0.474	0.027	0.030	DEP domain containing 1B	DEPDC1B
21682	TMEM106C	0.479	4.148	1.109	0.077	0.001	0.702	transmembrane protein 106C	TMEM106C
16155	PHF19	0.728	4.142	1.684	0.370	0.012	0.150	PHD finger protein 19	PHF19
20937	SUV39H1	0.822	4.137	1.546	0.073	0.007	0.131	suppressor of variegation 3-9 homolog 1	SUV39H1
2127	C19orf48	0.626	4.135	1.746	0.119	0.057	0.136	chromosome 19 open reading frame 48	C19orf48
17547	RAD51AP1	0.989	4.119	1.884	0.931	0.001	0.165	RAD51 associated protein 1	RAD51AP1
13905	MTFR2	0.805	4.119	1.394	0.066	0.018	0.167	mitochondrial fission regulator 2	MTFR2
3154	CDC6	0.994	4.089	1.290	0.984	0.021	0.460	cell division cycle 6	CDC6
5622	EME1	0.959	4.083	1.834	0.560	0.007	0.007	essential meiotic structure-specific endonuclease 1	EME1
18937	SERPINA9	2.006	4.059	1.338	0.409	0.400	0.334	serpin family A member 9	SERPINA9
14796	NRGN	0.856	4.024	3.773	0.559	0.062	0.140	neurogranin	NRGN
12676	LRR1	1.022	4.006	1.713	0.930	0.028	0.067	leucine rich repeat protein 1	LRR1
8036	HELLS	0.872	3.970	1.686	0.390	0.003	0.034	helicase, lymphoid-specific	HELLS
20740	STAMBPL1	2.461	3.936	2.444	0.024	0.057	0.211	STAM binding protein like 1	STAMBPL1
5263	DTL	0.997	3.932	1.683	0.985	0.026	0.085	denticleless E3 ubiquitin protein ligase homolog	DTL
20769	STC1	30.798	3.925	22.505	0.001	0.016	0.378	stanniocalcin 1	STC1
22094	TONSL	0.774	3.920	1.426	0.011	0.008	0.098	tonsoku-like, DNA repair protein	TONSL
13202	MCM8	1.033	3.917	1.548	0.854	0.011	0.066	minichromosome maintenance 8 homologous recombination repair f	MCM8
1314	ATAD2	1.058	3.914	1.421	0.739	0.039	0.148	ATPase family, AAA domain containing 2	ATAD2
13193	MCM10	1.038	3.910	1.601	0.701	0.001	0.074	minichromosome maintenance 10 replication initiation factor	MCM10
14909	NUDT8	0.395	3.907	1.359	0.172	0.022	0.334	nudix hydrolase 8	NUDT8
23961	ZNF367	0.598	3.905	1.176	0.176	0.005	0.611	zinc finger protein 367	ZNF367
1162	ARL6IP1	1.187	3.890	1.985	0.551	0.008	0.067	ADP ribosylation factor like GTPase 6 interacting protein 1	ARL6IP1
2060	C16orf59	0.897	3.867	1.657	0.467	0.009	0.020	chromosome 16 open reading frame 59	C16orf59
21954	TMPO	0.701	3.860	1.300	0.316	0.009	0.523	thymopoietin	TMPO
17857	RFC3	1.048	3.852	1.825	0.858	0.007	0.116	replication factor C subunit 3	RFC3
15892	PCNA	0.716	3.833	1.479	0.078	0.002	0.002	proliferating cell nuclear antigen	PCNA
13201	MCM7	0.724	3.830	1.739	0.095	0.003	0.186	minichromosome maintenance complex component 7	MCM7
16220	PIF1	0.727	3.819	1.763	0.280	0.012	0.251	PIF1 5'-to-3' DNA helicase	PIF1
4583	DBF4B	0.766	3.818	1.579	0.071	0.000	0.015	DBF4 zinc finger B	DBF4B
865	ANP32E	0.963	3.817	1.926	0.781	0.047	0.188	acidic nuclear phosphoprotein 32 family member E	ANP32E
1362	ATOH8	3.845	3.816	7.168	0.002	0.086	0.367	atonal bHLH transcription factor 8	ATOH8
17599	RANGAP1	0.691	3.805	1.526	0.006	0.031	0.371	Ran GTPase activating protein 1	RANGAP1
6344	FANCA	0.925	3.780	1.356	0.505	0.020	0.154	Fanconi anemia complementation group A	FANCA
21545	TIMELESS	1.119	3.772	1.956	0.715	0.008	0.006	timeless circadian clock	TIMELESS
2249	C21orf58	0.720	3.766	1.449	0.311	0.007	0.117	chromosome 21 open reading frame 58	C21orf58
14099	MYOCD	1.125	3.748	3.566	0.591	0.228	0.014	myocardin	MYOCD
4899	DIAPH3	0.854	3.734	1.344	0.690	0.111	0.106	diaphanous related formin 3	DIAPH3
8291	HN1	0.782	3.730	2.464	0.488	0.016	0.016	hematological and neurological expressed 1	HN1
16576	POLD1	0.876	3.712	1.748	0.161	0.009	0.070	polymerase (DNA) delta 1, catalytic subunit	POLD1
18277	RPL22L1	0.711	3.711	2.353	0.549	0.094	0.215	ribosomal protein L22 like 1	RPL22L1
22609	TUBB	0.777	3.710	1.576	0.066	0.040	0.374	tubulin beta class I	TUBB
19045	SGOL1	0.957	3.676	1.590	0.771	0.000	0.104	.	SGOL1
14847	NT5DC2	0.309	3.636	1.328	0.036	0.005	0.237	5'-nucleotidase domain containing 2	NT5DC2
3756	CLSPN	1.299	3.634	1.877	0.253	0.008	0.006	claspin	CLSPN
6694	FLJ46906	0.910	3.627	1.770	0.833	0.358	0.279	uncharacterized LOC441172	FLJ46906
13879	MT1L	3.810	3.627	4.100	0.015	0.125	0.057	metallothionein 1L (gene/pseudogene)	MT1L
23136	VIT	0.713	3.626	1.733	0.048	0.214	0.441	vitrin	VIT
4047	CPM	1.443	3.611	1.379	0.341	0.345	0.610	carboxypeptidase M	CPM
9066	ITGB3BP	0.714	3.597	1.157	0.380	0.017	0.622	integrin subunit beta 3 binding protein	ITGB3BP
18313	RPL39L	1.031	3.595	2.214	0.933	0.004	0.259	ribosomal protein L39 like	RPL39L
7743	GSG2	0.892	3.591	1.280	0.337	0.002	0.195	germ cell associated 2, haspin	GSG2
21223	TBX2	1.193	3.576	2.130	0.571	0.035	0.049	T-box 2	TBX2
20229	SNRPA	0.729	3.571	3.118	0.147	0.004	0.117	small nuclear ribonucleoprotein polypeptide A	SNRPA
18419	RRM1	0.826	3.542	1.367	0.356	0.008	0.370	ribonucleotide reductase catalytic subunit M1	RRM1
4710	DDX39A	1.162	3.511	2.261	0.617	0.009	0.125	DEAD-box helicase 39A	DDX39A
20446	SPDL1	1.098	3.499	1.600	0.119	0.011	0.070	spindle apparatus coiled-coil protein 1	SPDL1
7129	GCAT	0.395	3.499	1.803	0.023	0.015	0.181	glycine C-acetyltransferase	GCAT
2088	C17orf53	0.814	3.491	1.467	0.380	0.001	0.126	chromosome 17 open reading frame 53	C17orf53
15735	PARPBP	0.968	3.490	1.476	0.643	0.039	0.010	PARP1 binding protein	PARPBP
18195	RNU6ATAC	0.927	3.464	0.635	0.959	0.326	0.768	RNA, U6atac small nuclear (U12-dependent splicing)	RNU6ATAC
3240	CDKN2C	0.742	3.452	1.341	0.081	0.020	0.239	cyclin-dependent kinase inhibitor 2C	CDKN2C
4124	CRIP1	0.325	3.451	2.412	0.070	0.027	0.016	cysteine rich protein 1	CRIP1
21976	TMSB15A	0.701	3.444	2.092	0.305	0.004	0.341	thymosin beta 15a	TMSB15A
15701	PAQR4	0.573	3.439	1.359	0.044	0.054	0.224	progestin and adipoQ receptor family member 4	PAQR4
3472	CHAF1B	0.679	3.432	1.153	0.074	0.005	0.395	chromatin assembly factor 1 subunit B	CHAF1B
8263	HMGB3	0.784	3.422	1.710	0.270	0.152	0.367	high mobility group box 3	HMGB3
7939	HAUS8	0.749	3.421	1.337	0.059	0.006	0.010	HAUS augmin like complex subunit 8	HAUS8
15482	ORC1	0.936	3.419	1.369	0.514	0.000	0.105	origin recognition complex subunit 1	ORC1
14803	NRM	0.628	3.393	1.851	0.116	0.002	0.165	nurim (nuclear envelope membrane protein)	NRM
12589	LOC81691	0.602	3.386	1.532	0.072	0.012	0.189	exonuclease NEF-sp	LOC81691
18508	RUVBL2	0.988	3.384	2.493	0.954	0.003	0.200	RuvB like AAA ATPase 2	RUVBL2
17197	PSMC3IP	1.114	3.376	1.778	0.357	0.051	0.014	PSMC3 interacting protein	PSMC3IP
6511	FEN1	1.420	3.375	1.565	0.328	0.016	0.057	flap structure-specific endonuclease 1	FEN1

17858	RFC4	0.913	3.372	1.380	0.111	0.007	0.010	replication factor C subunit 4	RFC4
13516	MIS18A	1.040	3.356	1.856	0.426	0.000	0.141	MIS18 kinetochore protein A	MIS18A
5774	ERCC6L	0.931	3.346	1.407	0.525	0.001	0.217	excision repair cross-complementation group 6 like	ERCC6L
19331	SLC25A10	0.940	3.327	1.749	0.130	0.004	0.010	solute carrier family 25 member 10	SLC25A10
14591	NME1	1.302	3.310	3.277	0.422	0.027	0.229	NME/NM23 nucleoside diphosphate kinase 1	NME1
5089	DNAJC9	0.998	3.301	1.538	0.972	0.004	0.085	DnaJ heat shock protein family (Hsp40) member C9	DNAJC9
17591	RANBP1	1.011	3.277	2.010	0.965	0.018	0.048	RAN binding protein 1	RANBP1
23192	VRK1	0.905	3.270	1.361	0.237	0.000	0.353	vaccinia related kinase 1	VRK1
7929	HAS2-AS1	1.542	3.239	1.455	0.061	0.156	0.059	HAS2 antisense RNA 1	HAS2-AS1
5921	EZH2	1.143	3.239	1.853	0.602	0.008	0.049	enhancer of zeste homolog 2 (Drosophila)	EZH2
4858	DHFR	1.084	3.238	1.948	0.636	0.005	0.017	dihydrofolate reductase	DHFR
23291	WDR34	0.707	3.213	1.902	0.130	0.011	0.196	WD repeat domain 34	WDR34
5303	DUSP4	1.467	3.184	3.033	0.666	0.416	0.500	dual specificity phosphatase 4	DUSP4
22621	TUBG1	1.456	3.175	1.784	0.181	0.080	0.283	tubulin gamma 1	TUBG1
3337	CENPO	0.891	3.174	1.600	0.013	0.000	0.029	centromere protein O	CENPO
14303	NDC1	1.064	3.169	1.331	0.444	0.002	0.262	NDC1 transmembrane nucleoporin	NDC1
24415	ZWILCH	0.989	3.168	1.498	0.718	0.032	0.106	zwilch kinetochore protein	ZWILCH
4673	DDIAS	0.951	3.167	1.276	0.458	0.041	0.147	DNA damage induced apoptosis suppressor	DDIAS
5115	DNMT1	1.048	3.166	1.994	0.815	0.005	0.125	DNA (cytosine-5-)-methyltransferase 1	DNMT1
15893	PCNA-AS1	0.799	3.133	1.383	0.521	0.044	0.222	PCNA antisense RNA 1	PCNA-AS1
20356	SPA17	1.176	3.133	1.882	0.542	0.097	0.136	sperm autoantigenic protein 17	SPA17
5978	FAM101B	0.916	3.123	1.718	0.751	0.135	0.222	family with sequence similarity 101 member B	FAM101B
4868	DHRS3	0.923	3.114	2.399	0.942	0.095	0.549	dehydrogenase/reductase (SDR family) member 3	DHRS3
144	ACADVL	0.755	3.102	2.249	0.625	0.025	0.223	acyl-CoA dehydrogenase, very long chain	ACADVL
14000	MXD3	0.926	3.102	1.947	0.682	0.001	0.058	MAX dimerization protein 3	MXD3
7212	GGH	1.957	3.094	4.622	0.053	0.121	0.145	gamma-glutamyl hydrolase	GGH
16635	POM121L9P	1.622	3.083	4.727	0.475	0.111	0.289	POM121 transmembrane nucleoporin like 9, pseudogene	POM121L9P
16991	PRL	4.158	3.083	1.434	0.297	0.452	0.420	prolactin	PRL
5479	EGR1	0.585	3.068	2.054	0.007	0.401	0.002	early growth response 1	EGR1
16594	POLQ	0.950	3.067	1.500	0.376	0.000	0.073	polymerase (DNA) theta	POLQ
15641	PAFAH1B3	0.661	3.064	1.629	0.343	0.063	0.069	platelet activating factor acetylhydrolase 1b catalytic subunit 3	PAFAH1B3
16944	PRIM1	0.735	3.062	1.267	0.198	0.011	0.269	primase (DNA) subunit 1	PRIM1
23367	WHSC1	0.922	3.062	1.552	0.098	0.030	0.061	Wolf-Hirschhorn syndrome candidate 1	WHSC1
4819	DERL3	1.008	3.057	1.418	0.953	0.193	0.075	derlin 3	DERL3
21739	TMEM160	0.491	3.053	1.769	0.087	0.009	0.120	transmembrane protein 160	TMEM160
14256	NCAPD2	1.165	3.035	2.080	0.511	0.012	0.039	non-SMC condensin I complex subunit D2	NCAPD2
2843	CCDC18	0.851	3.035	1.240	0.410	0.015	0.199	coiled-coil domain containing 18	CCDC18
6452	FBXO5	1.032	3.013	1.361	0.926	0.004	0.469	F-box protein 5	FBXO5
13195	MCM3	0.922	3.002	1.360	0.746	0.013	0.221	minichromosome maintenance complex component 3	MCM3
1740	BLM	0.927	2.991	1.274	0.347	0.032	0.003	Bloom syndrome RecQ like helicase	BLM
16582	POLE	0.714	2.983	1.134	0.140	0.002	0.516	polymerase (DNA) epsilon, catalytic subunit	POLE
13881	MT1X	2.740	2.972	2.372	0.021	0.188	0.095	metallothionein 1X	MT1X
3130	CDC25A	1.434	2.971	1.573	0.006	0.032	0.023	cell division cycle 25A	CDC25A
7888	H3F3A	0.377	2.967	0.299	0.121	0.530	0.052	H3 histone, family 3A	H3F3A
16500	PMCH	1.188	2.956	1.759	0.590	0.016	0.186	pro-melanin concentrating hormone	PMCH
2156	C1QTNF2	0.480	2.954	0.806	0.180	0.044	0.667	C1q and tumor necrosis factor related protein 2	C1QTNF2
16073	PF4	1.337	2.951	1.380	0.423	0.041	0.155	platelet factor 4	PF4
21998	TNFAIP8L1	0.648	2.948	1.795	0.296	0.067	0.266	TNF alpha induced protein 8 like 1	TNFAIP8L1
1669	BCYRN1	1.454	2.938	3.243	0.654	0.079	0.197	brain cytoplasmic RNA 1	BCYRN1
6616	FJX1	1.205	2.924	3.064	0.633	0.023	0.108	four jointed box 1	FJX1
8577	HYLS1	0.969	2.923	1.454	0.888	0.013	0.085	hydrolethalus syndrome 1	HYLS1
16574	POLA2	0.862	2.923	1.150	0.427	0.090	0.617	polymerase (DNA) alpha 2, accessory subunit	POLA2
5391	ECT2	1.510	2.920	1.701	0.492	0.037	0.356	epithelial cell transforming 2	ECT2
13198	MCM4	0.988	2.888	1.419	0.973	0.038	0.414	minichromosome maintenance complex component 4	MCM4
4695	DDX12P	0.889	2.877	1.396	0.278	0.011	0.268	DEAD/H-box helicase 12, pseudogene	DDX12P
13200	MCM6	0.564	2.868	1.314	0.148	0.001	0.238	minichromosome maintenance complex component 6	MCM6
8520	HSPB6	0.083	2.852	0.842	0.000	0.037	0.673	heat shock protein family B (small) member 6	HSPB6
5373	EBPL	0.299	2.852	1.084	0.273	0.147	0.885	emopamil binding protein like	EBPL
5350	E2F2	0.935	2.845	1.376	0.376	0.013	0.028	E2F transcription factor 2	E2F2
21502	THOP1	1.070	2.833	1.686	0.640	0.027	0.081	thimet oligopeptidase 1	THOP1
16629	POM121L10P	1.192	2.823	3.285	0.605	0.047	0.336	POM121 transmembrane nucleoporin like 10, pseudogene	POM121L10P
14555	NKX3-1	2.551	2.822	2.253	0.133	0.319	0.364	NK3 homeobox 1	NKX3-1
4239	CST2	1.001	2.817	3.704	0.714	0.402	0.395	cystatin SA	CST2
20696	ST3GAL5	0.668	2.811	1.457	0.447	0.317	0.661	ST3 beta-galactoside alpha-2,3-sialyltransferase 5	ST3GAL5
5253	DSN1	0.958	2.803	1.334	0.706	0.002	0.131	DSN1 homolog, MIS12 kinetochore complex component	DSN1
21224	TBX2-AS1	1.221	2.795	2.359	0.179	0.029	0.040	TBX2 antisense RNA 1	TBX2-AS1
12792	LSM4	0.836	2.783	1.824	0.353	0.033	0.003	LSM4 homolog, U6 small nuclear RNA and mRNA degradation associa	LSM4
1390	ATP2A1-AS1	0.958	2.779	1.333	0.897	0.060	0.490	ATP2A1 antisense RNA 1	ATP2A1-AS1
18027	RMI2	0.605	2.771	1.256	0.022	0.017	0.001	RecQ mediated genome instability 2	RMI2
8133	HIRIP3	0.841	2.769	1.362	0.306	0.004	0.181	HIRA interacting protein 3	HIRIP3
22125	TP53I3	0.281	2.764	1.552	0.082	0.008	0.197	tumor protein p53 inducible protein 3	TP53I3
1054	ARHGAP11B	0.894	2.761	1.572	0.496	0.099	0.271	Rho GTPase activating protein 11B	ARHGAP11B
722	ANAPC15	0.697	2.757	1.908	0.015	0.003	0.284	anaphase promoting complex subunit 15	ANAPC15
9647	KNTC1	0.936	2.756	1.321	0.759	0.008	0.416	kinetochore associated 1	KNTC1
21850	TMEM256	0.518	2.749	1.763	0.214	0.045	0.368	transmembrane protein 256	TMEM256
22616	TUBB6	0.766	2.747	1.322	0.179	0.046	0.568	tubulin beta 6 class V	TUBB6
10102	LIN9	0.980	2.738	1.494	0.824	0.016	0.197	lin-9 DREAM MuvB core complex component	LIN9
8283	HMMR-AS1	1.000	2.737	1.244	0.714	0.059	0.133	HMMR antisense RNA 1	HMMR-AS1
20779	STIL	1.047	2.737	1.316	0.880	0.014	0.533	SCL/TAL1 interrupting locus	STIL
23405	WRAP53	1.078	2.732	1.399	0.207	0.003	0.090	WD repeat containing antisense to TP53	WRAP53
19540	SLC4A8	0.696	2.731	1.204	0.529	0.078	0.710	solute carrier family 4 member 8	SLC4A8
7403	GNG11	1.775	2.720	2.655	0.378	0.172	0.408	G protein subunit gamma 11	GNG11
3776	CMAHP	1.061	2.719	2.006	0.870	0.415	0.445	cytidine monophospho-N-acetylneuraminic acid hydroxylase, pseudo	CMAHP
6908	FUS	1.252	2.715	2.041	0.287	0.000	0.243	FUS RNA binding protein	FUS
739	ANGPTL5	0.645	2.709	1.318	0.630	0.407	0.723	angiopoietin like 5	ANGPTL5
23325	WDR76	1.002	2.698	1.366	0.956	0.005	0.224	WD repeat domain 76	WDR76
13882	MT2A	12.229	2.693	6.170	0.044	0.018	0.201	metallothionein 2A	MT2A
13839	MSH5	0.747	2.693	1.241	0.269	0.001	0.280	mutS homolog 5	MSH5
18909	SEPW1	0.487	2.688	1.903	0.022	0.087	0.238	selenoprotein W, 1	SEPW1
13610	MNS1	0.601	2.670	1.044	0.313	0.008	0.893	meiosis specific nuclear structural 1	MNS1
1915	BUB3	0.959	2.666	1.531	0.848	0.030	0.159	BUB3, mitotic checkpoint protein	BUB3

21780	TMEM190	0.903	2.655	1.564	0.686	0.331	0.519	transmembrane protein 190	TMEM190
2977	CCNE2	1.042	2.646	1.115	0.759	0.121	0.620	cyclin E2	CCNE2
7540	GPM6B	0.407	2.645	1.564	0.174	0.022	0.199	glycoprotein M6B	GPM6B
18800	SDSL	0.772	2.645	1.827	0.603	0.045	0.249	serine dehydratase like	SDSL
14206	NASP	1.063	2.641	1.734	0.775	0.042	0.353	nuclear autoantigenic sperm protein	NASP
910	AP2S1	0.611	2.632	1.821	0.046	0.008	0.275	adaptor related protein complex 2 sigma 1 subunit	AP2S1
8378	HOXC-AS3	0.669	2.626	1.502	0.297	0.459	0.584	HOXC cluster antisense RNA 3	HOXC-AS3
4188	CSE1L	1.138	2.608	1.372	0.043	0.004	0.283	chromosome segregation 1 like	CSE1L
21440	TGFB3	0.895	2.604	2.078	0.799	0.093	0.252	transforming growth factor beta 3	TGFB3
22087	TOMM34	0.598	2.602	1.292	0.090	0.010	0.209	translocase of outer mitochondrial membrane 34	TOMM34
8230	HLA-DRA	1.479	2.592	1.190	0.042	0.499	0.161	major histocompatibility complex, class II, DR alpha	HLA-DRA
17771	RCAN1	4.477	2.585	2.271	0.185	0.129	0.326	regulator of calcineurin 1	RCAN1
12633	LPPR3	1.050	2.584	1.729	0.874	0.419	0.465	.	LPPR3
677	ALYREF	0.901	2.582	1.701	0.215	0.027	0.003	Aly/REF export factor	ALYREF
3209	CDK2	1.041	2.575	1.434	0.546	0.033	0.112	cyclin-dependent kinase 2	CDK2
6276	FAM72B	0.981	2.573	1.339	0.812	0.002	0.117	family with sequence similarity 72 member B	FAM72B
20825	STRA13	0.899	2.572	1.810	0.762	0.034	0.219	stimulated by retinoic acid 13	STRA13
19377	SLC25A5	0.957	2.566	1.720	0.508	0.001	0.072	solute carrier family 25 member 5	SLC25A5
19843	SNHG19	0.389	2.564	2.373	0.156	0.135	0.490	small nucleolar RNA host gene 19	SNHG19
6351	FANCG	0.705	2.562	0.988	0.026	0.028	0.916	Fanconi anemia complementation group G	FANCG
4998	DMC1	0.840	2.548	1.994	0.427	0.038	0.329	DNA meiotic recombinase 1	DMC1
16112	PGF	4.544	2.544	12.639	0.384	0.517	0.427	placental growth factor	PGF
3738	CLN6	1.010	2.541	1.510	0.695	0.030	0.003	ceroid-lipofuscinosis, neuronal 6, late infantile, variant	CLN6
5845	ETFB	0.740	2.539	2.308	0.315	0.044	0.355	electron transfer flavoprotein beta subunit	ETFB
16583	POLE2	0.916	2.533	1.205	0.624	0.011	0.439	polymerase (DNA) epsilon 2, accessory subunit	POLE2
3626	CKLF	0.848	2.533	1.220	0.179	0.010	0.109	chemokine-like factor	CKLF
16546	PNP	1.360	2.530	1.839	0.747	0.279	0.252	purine nucleoside phosphorylase	PNP
18506	RUVBL1	0.920	2.529	1.831	0.250	0.006	0.116	RuvB like AAA ATPase 1	RUVBL1
12044	LOC10193007	0.917	2.526	3.350	0.843	0.043	0.332	uncharacterized LOC101930071	LOC101930071
2101	C17orf89	0.480	2.516	1.589	0.128	0.025	0.154	chromosome 17 open reading frame 89	C17orf89
18577	SAMD11	1.935	2.514	6.935	0.106	0.404	0.422	sterile alpha motif domain containing 11	SAMD11
17856	RFC2	1.436	2.511	1.365	0.016	0.040	0.014	replication factor C subunit 2	RFC2
7080	GAS2L3	0.842	2.502	1.237	0.754	0.090	0.603	growth arrest specific 2 like 3	GAS2L3
181	ACOT7	1.273	2.499	2.358	0.197	0.010	0.068	acyl-CoA thioesterase 7	ACOT7
7188	GEN1	0.970	2.494	1.189	0.640	0.022	0.170	GEN1, Holliday junction 5' flap endonuclease	GEN1
18234	RPA3	0.967	2.488	2.027	0.829	0.022	0.147	replication protein A3	RPA3
738	ANGPTL4	1.385	2.483	5.807	0.810	0.414	0.421	angiopoietin like 4	ANGPTL4
21618	TM4SF1	0.347	2.482	1.066	0.200	0.010	0.868	transmembrane 4 L six family member 1	TM4SF1
17394	PXMP2	0.579	2.480	1.362	0.138	0.092	0.074	peroxisomal membrane protein 2	PXMP2
20983	SYNE2	0.617	2.479	1.092	0.099	0.011	0.738	spectrin repeat containing nuclear envelope protein 2	SYNE2
3241	CDKN2D	1.275	2.471	1.684	0.475	0.024	0.266	cyclin-dependent kinase inhibitor 2D	CDKN2D
8063	HES1	1.272	2.471	3.283	0.249	0.327	0.005	hes family bHLH transcription factor 1	HES1
16474	PLP2	0.517	2.464	1.622	0.020	0.016	0.287	proteolipid protein 2 (colonic epithelium-enriched)	PLP2
10013	LGALS1	0.464	2.463	1.720	0.021	0.036	0.069	lectin, galactoside binding soluble 1	LGALS1
3353	CEP152	0.989	2.454	1.231	0.918	0.065	0.204	centrosomal protein 152	CEP152
21955	TMPO-AS1	0.930	2.453	1.173	0.485	0.001	0.146	TMPO antisense RNA 1	TMPO-AS1
7059	GAMT	0.387	2.439	1.225	0.137	0.036	0.469	guanidinoacetate N-methyltransferase	GAMT
11217	LOC10028863	1.046	2.439	1.293	0.670	0.094	0.189	OTU deubiquitinase 7A pseudogene	LOC100288637
14123	MZT1	1.587	2.439	1.301	0.070	0.007	0.513	mitotic spindle organizing protein 1	MZT1
4133	CRISPLD2	0.764	2.433	1.159	0.683	0.379	0.715	cysteine rich secretory protein LCCL domain containing 2	CRISPLD2
5161	DPF1	1.000	2.431	1.558	0.998	0.096	0.141	double PHD fingers 1	DPF1
14030	MYEF2	0.995	2.429	1.077	0.978	0.028	0.842	myelin expression factor 2	MYEF2
3339	CENPQ	0.958	2.426	0.998	0.904	0.028	0.996	centromere protein Q	CENPQ
3101	CD74	1.926	2.423	1.060	0.213	0.568	0.922	CD74 molecule	CD74
3350	CEP128	0.865	2.422	1.113	0.262	0.030	0.115	centrosomal protein 128	CEP128
1316	ATAD3A	1.096	2.421	1.945	0.672	0.010	0.211	ATPase family, AAA domain containing 3A	ATAD3A
21427	TFPI2	101.175	2.417	11.850	0.389	0.262	0.201	tissue factor pathway inhibitor 2	TFPI2
12911	MAD2L2	0.887	2.417	1.894	0.731	0.022	0.280	MAD2 mitotic arrest deficient-like 2 (yeast)	MAD2L2
17486	RAB3B	1.078	2.413	1.961	0.750	0.234	0.088	RAB3B, member RAS oncogene family	RAB3B
430	ADRB2	0.630	2.409	1.413	0.431	0.088	0.629	adrenoceptor beta 2	ADRB2
14719	NPM3	0.534	2.404	1.943	0.288	0.042	0.369	nucleophosmin/nucleoplasmin 3	NPM3
6385	FBL	0.607	2.403	1.711	0.157	0.003	0.399	fibrillarin	FBL
1802	BORA	1.184	2.399	1.313	0.256	0.017	0.180	bora, aurora kinase A activator	BORA
16866	PRADC1	0.612	2.398	1.323	0.102	0.087	0.112	protease-associated domain containing 1	PRADC1
21276	TCF7	0.726	2.396	1.209	0.405	0.038	0.675	transcription factor 7 (T-cell specific, HMG-box)	TCF7
19286	SLC19A1	0.959	2.394	1.702	0.608	0.140	0.068	solute carrier family 19 member 1	SLC19A1
14019	MYCBP	1.089	2.393	1.775	0.783	0.035	0.009	MYC binding protein	MYCBP
17859	RFC5	1.011	2.382	1.257	0.946	0.014	0.434	replication factor C subunit 5	RFC5
3332	CENPJ	1.042	2.381	1.149	0.141	0.008	0.255	centromere protein J	CENPJ
3978	COQ2	1.444	2.378	1.997	0.048	0.012	0.063	coenzyme Q2, polyprenyltransferase	COQ2
17876	RFWD3	0.962	2.376	1.120	0.616	0.006	0.156	ring finger and WD repeat domain 3	RFWD3
13743	MRPL24	0.886	2.371	2.301	0.419	0.018	0.138	mitochondrial ribosomal protein L24	MRPL24
5151	DONSON	0.895	2.370	1.227	0.172	0.005	0.287	downstream neighbor of SON	DONSON
13869	MT1A	1.797	2.368	4.038	0.346	0.327	0.149	metallothionein 1A	MT1A
4910	DIO3	1.241	2.367	1.774	0.517	0.390	0.465	deiodinase, iodothyronine, type III	DIO3
8111	HIF1A-AS2	1.624	2.364	2.431	0.273	0.025	0.197	HIF1A antisense RNA 2	HIF1A-AS2
7932	HAUS1	0.661	2.363	1.073	0.307	0.029	0.826	HAUS augmin like complex subunit 1	HAUS1
4402	CXCL6	1760.002	2.361	71.808	0.014	0.181	0.415	C-X-C motif chemokine ligand 6	CXCL6
2585	CALM3	0.575	2.354	1.500	0.027	0.007	0.007	calmodulin 3 (phosphorylase kinase, delta)	CALM3
19395	SLC26A9	1.656	2.353	1.246	0.098	0.210	0.446	solute carrier family 26 member 9	SLC26A9
403	ADIRF	0.165	2.350	1.778	0.004	0.105	0.016	adipogenesis regulatory factor	ADIRF
9478	KIF24	0.937	2.349	1.158	0.441	0.002	0.058	kinesin family member 24	KIF24
23710	ZFP36L2	0.265	2.344	1.160	0.034	0.270	0.350	ZFP36 ring finger protein-like 2	ZFP36L2
2472	C9orf40	0.968	2.342	1.322	0.882	0.068	0.295	chromosome 9 open reading frame 40	C9orf40
17811	REEP2	0.457	2.341	1.458	0.134	0.078	0.174	receptor accessory protein 2	REEP2
1409	ATP5G1	1.169	2.338	2.079	0.098	0.031	0.067	ATP synthase, H+ transporting, mitochondrial Fo complex subunit C1	ATP5G1
18398	RPSA	0.390	2.338	1.539	0.127	0.046	0.369	ribosomal protein SA	RPSA
20223	SNRNP25	0.761	2.337	1.632	0.516	0.036	0.303	small nuclear ribonucleoprotein U11/U12 subunit 25	SNRNP25
4400	CXCL3	246.027	2.330	5.134	0.285	0.196	0.400	C-X-C motif chemokine ligand 3	CXCL3
12845	LY6K	1.490	2.328	2.997	0.570	0.208	0.283	lymphocyte antigen 6 complex, locus K	LY6K
2178	C1orf112	1.004	2.327	1.111	0.839	0.010	0.666	chromosome 1 open reading frame 112	C1orf112

18559	SAC3D1	0.989	2.326	1.545	0.887	0.028	0.018	SAC3 domain containing 1	SAC3D1
5646	ENC1	0.947	2.324	0.837	0.905	0.006	0.786	ectodermal-neural cortex 1	ENC1
12791	LSM3	0.963	2.320	1.438	0.349	0.016	0.219	LSM3 homolog, U6 small nuclear RNA and mRNA degradation associa	LSM3
14257	NCAPD3	1.238	2.319	1.291	0.380	0.013	0.283	non-SMC condensin II complex subunit D3	NCAPD3
7184	GEMIN6	0.775	2.317	1.361	0.051	0.034	0.114	gem nuclear organelle associated protein 6	GEMIN6
6880	FSTL3	1.559	2.314	1.926	0.373	0.047	0.209	follistatin like 3	FSTL3
22317	TRIM59	0.626	2.313	1.184	0.121	0.022	0.553	tripartite motif containing 59	TRIM59
20951	SVIP	0.788	2.312	1.344	0.418	0.005	0.267	small VCP/p97-interacting protein	SVIP
5619	EMC9	1.355	2.311	2.207	0.113	0.003	0.164	ER membrane protein complex subunit 9	EMC9
2766	CCBE1	0.566	2.311	1.485	0.062	0.003	0.148	collagen and calcium binding EGF domains 1	CCBE1
17975	RIBC2	1.070	2.310	1.303	0.353	0.126	0.001	RIB43A domain with coiled-coils 2	RIBC2
3131	CDC25B	0.592	2.308	1.391	0.163	0.064	0.247	cell division cycle 25B	CDC25B
7880	H2AFV	0.936	2.305	1.393	0.491	0.098	0.118	H2A histone family member V	H2AFV
6384	FBF1	0.721	2.303	1.198	0.217	0.017	0.467	Fas binding factor 1	FBF1
18150	RNF5	0.333	2.301	0.753	0.246	0.176	0.715	ring finger protein 5	RNF5
5376	ECE2	1.512	2.298	2.424	0.117	0.035	0.287	endothelin converting enzyme 2	ECE2
14125	MZT2B	0.646	2.297	1.832	0.141	0.010	0.124	mitotic spindle organizing protein 2B	MZT2B
5356	E2F8	1.030	2.296	1.290	0.370	0.000	0.096	E2F transcription factor 8	E2F8
23478	XRCC2	1.071	2.295	1.221	0.070	0.001	0.181	X-ray repair complementing defective repair in Chinese hamster cells	XRCC2
12793	LSM5	0.937	2.283	1.425	0.527	0.032	0.076	LSM5 homolog, U6 small nuclear RNA and mRNA degradation associa	LSM5
1798	BOLA3	1.685	2.273	1.747	0.022	0.109	0.465	bolA family member 3	BOLA3
15011	ODC1	1.031	2.269	1.322	0.935	0.086	0.382	ornithine decarboxylase 1	ODC1
22924	UQCC2	1.226	2.267	1.938	0.522	0.030	0.264	ubiquinol-cytochrome c reductase complex assembly factor 2	UQCC2
4021	COX8A	1.138	2.266	1.983	0.012	0.015	0.002	cytochrome c oxidase subunit 8A	COX8A
8302	HNRNPA1	0.619	2.266	1.603	0.267	0.066	0.413	heterogeneous nuclear ribonucleoprotein A1	HNRNPA1
4944	DKK1	0.844	2.265	1.509	0.665	0.507	0.664	dickkopf WNT signaling pathway inhibitor 1	DKK1
18563	SAE1	0.839	2.264	1.414	0.061	0.020	0.081	SUMO1 activating enzyme subunit 1	SAE1
3451	CGA	1.043	2.261	1.000	0.425	0.382	0.943	glycoprotein hormones, alpha polypeptide	CGA
3950	COMMD4	0.830	2.259	1.690	0.120	0.005	0.211	COMM domain containing 4	COMMD4
4330	CTPS1	1.282	2.257	1.578	0.401	0.069	0.047	CTP synthase 1	CTPS1
2241	C20orf27	0.500	2.252	1.457	0.078	0.032	0.138	chromosome 20 open reading frame 27	C20orf27
17590	RAN	0.991	2.251	1.737	0.946	0.006	0.029	RAN, member RAS oncogene family	RAN
20234	SNRPD1	1.152	2.250	1.839	0.479	0.005	0.245	small nuclear ribonucleoprotein D1 polypeptide	SNRPD1
4661	DCXR	0.658	2.249	1.765	0.329	0.010	0.406	dicarbonyl/L-xylulose reductase	DCXR
8164	HIST1H2BL	0.908	2.245	0.908	0.425	0.423	0.424	histone cluster 1, H2bl	HIST1H2BL
11524	LOC10192723	0.640	2.239	0.703	0.423	0.202	0.502	uncharacterized LOC101927230	LOC101927230
13424	MGP	0.317	2.239	0.697	0.124	0.080	0.336	matrix Gla protein	MGP
17093	PRR7	0.743	2.237	2.400	0.375	0.036	0.057	proline rich 7 (synaptic)	PRR7
5556	EIF5A	1.206	2.228	2.125	0.085	0.017	0.043	eukaryotic translation initiation factor 5A	EIF5A
5285	DUSP1	2.459	2.225	1.466	0.080	0.070	0.327	dual specificity phosphatase 1	DUSP1
3780	CMC2	1.412	2.224	1.415	0.105	0.021	0.031	C-x9-C motif containing 2	CMC2
6814	FOXQ1	0.413	2.221	1.293	0.439	0.283	0.785	forkhead box Q1	FOXQ1
13908	MTHFD1	0.922	2.221	1.515	0.694	0.046	0.147	methylenetetrahydrofolate dehydrogenase, cyclohydrolase and form	MTHFD1
16743	PPIH	0.932	2.218	1.447	0.360	0.001	0.178	peptidylprolyl isomerase H	PPIH
5142	DOK1	1.097	2.217	1.667	0.844	0.092	0.495	docking protein 1	DOK1
21546	TIMM10	0.946	2.216	1.492	0.828	0.021	0.465	translocase of inner mitochondrial membrane 10 homolog (yeast)	TIMM10
19854	SNHG8	0.344	2.214	1.100	0.104	0.062	0.830	small nucleolar RNA host gene 8	SNHG8
7210	GGCT	0.790	2.214	2.008	0.461	0.004	0.439	gamma-glutamylcyclotransferase	GGCT
1801	BOP1	1.073	2.214	2.370	0.847	0.056	0.254	block of proliferation 1	BOP1
5239	DSCC1	0.661	2.212	0.965	0.014	0.007	0.815	DNA replication and sister chromatid cohesion 1	DSCC1
8865	IMPDH2	0.377	2.201	1.426	0.209	0.047	0.407	IMP (inosine 5'-monophosphate) dehydrogenase 2	IMPDH2
8306	HNRNPA2B1	1.122	2.201	1.724	0.196	0.078	0.147	heterogeneous nuclear ribonucleoprotein A2/B1	HNRNPA2B1
21531	TICRR	0.881	2.199	1.308	0.738	0.048	0.484	TOPBP1 interacting checkpoint and replication regulator	TICRR
12835	LXN	0.504	2.198	3.154	0.439	0.086	0.047	latexin	LXN
8325	HNRNPM	1.039	2.191	2.213	0.803	0.076	0.273	heterogeneous nuclear ribonucleoprotein M	HNRNPM
1295	ASPHD1	1.055	2.190	2.274	0.879	0.014	0.404	aspartate beta-hydroxylase domain containing 1	ASPHD1
20231	SNRPB	1.227	2.189	1.762	0.152	0.002	0.147	small nuclear ribonucleoprotein polypeptides B and B1	SNRPB
14386	NEDD8	0.787	2.186	1.653	0.160	0.013	0.322	neural precursor cell expressed, developmentally down-regulated 8	NEDD8
21435	TGFB1	0.693	2.181	1.987	0.230	0.064	0.206	transforming growth factor, beta 1	TGFB1
4582	DBF4	1.056	2.178	1.248	0.882	0.047	0.493	DBF4 zinc finger	DBF4
21465	THAP10	1.060	2.178	1.265	0.755	0.031	0.468	THAP domain containing 10	THAP10
1645	BCL2L12	1.000	2.177	1.500	1.000	0.015	0.373	BCL2 like 12	BCL2L12
8274	HMGN3	0.855	2.177	1.840	0.372	0.060	0.314	high mobility group nucleosomal binding domain 3	HMGN3
4557	DANCR	0.472	2.174	1.732	0.116	0.062	0.296	differentiation antagonizing non-protein coding RNA	DANCR
14411	NELFE	0.815	2.171	1.563	0.191	0.001	0.275	negative elongation factor complex member E	NELFE
14415	NEMP1	0.892	2.165	0.874	0.323	0.010	0.570	nuclear envelope integral membrane protein 1	NEMP1
21497	THOC3	0.779	2.163	1.159	0.042	0.011	0.140	THO complex 3	THOC3
2584	CALM2	0.765	2.161	1.055	0.057	0.012	0.407	calmodulin 2 (phosphorylase kinase, delta)	CALM2
875	ANXA2	0.496	2.160	1.708	0.031	0.018	0.207	annexin A2	ANXA2
7768	GSTO2	0.941	2.159	3.103	0.773	0.010	0.070	glutathione S-transferase omega 2	GSTO2
3995	COTL1	1.205	2.156	2.392	0.693	0.161	0.354	coactosin like F-actin binding protein 1	COTL1
22649	TWIST1	0.989	2.155	2.053	0.966	0.044	0.144	twist family bHLH transcription factor 1	TWIST1
6803	FOXO1	0.868	2.153	0.860	0.352	0.449	0.390	forkhead box O1	FOXO1
5216	DRAP1	0.850	2.150	1.717	0.474	0.005	0.123	DR1 associated protein 1	DRAP1
18361	RPS2	0.385	2.149	1.324	0.042	0.008	0.422	ribosomal protein S2	RPS2
13741	MRPL23	0.714	2.147	2.019	0.152	0.009	0.190	mitochondrial ribosomal protein L23	MRPL23
22155	TPM2	0.540	2.146	1.306	0.361	0.150	0.523	tropomyosin 2 (beta)	TPM2
17948	RHNO1	0.923	2.145	1.352	0.447	0.056	0.025	RAD9-HUS1-RAD1 interacting nuclear orphan 1	RHNO1
17586	RAMP1	2.227	2.144	2.160	0.091	0.056	0.559	receptor activity modifying protein 1	RAMP1
599	ALDH16A1	0.897	2.142	1.449	0.650	0.038	0.332	aldehyde dehydrogenase 16 family member A1	ALDH16A1
14059	MYL6	0.870	2.142	1.926	0.732	0.070	0.254	myosin light chain 6	MYL6
17779	RCCD1	0.914	2.142	1.321	0.625	0.071	0.172	RCC1 domain containing 1	RCCD1
21851	TMEM256-PLS	0.445	2.139	1.154	0.004	0.099	0.741	TMEM256-PLSCR3 readthrough (NMD candidate)	TMEM256-PLSCR3
14769	NR4A1	0.712	2.135	1.023	0.029	0.020	0.960	nuclear receptor subfamily 4 group A member 1	NR4A1
3867	CNTROB	0.913	2.133	1.290	0.366	0.002	0.092	centrobin, centriole duplication and spindle assembly protein	CNTROB
1434	ATP6V0E2	0.561	2.131	1.157	0.068	0.330	0.793	ATPase H+ transporting V0 subunit e2	ATP6V0E2
19095	SH3RF3	0.516	2.131	1.219	0.149	0.058	0.413	SH3 domain containing ring finger 3	SH3RF3
4774	DEFB124	1.000	2.129	1.000	0.714	0.423	0.943	defensin beta 124	DEFB124
4370	CUL4B	1.601	2.128	2.412	0.265	0.187	0.414	cullin 4B	CUL4B
7255	GINS3	0.943	2.127	1.111	0.401	0.015	0.338	GINS complex subunit 3	GINS3
1488	AURKAIP1	0.971	2.125	1.747	0.868	0.000	0.328	aurora kinase A interacting protein 1	AURKAIP1

5667	ENPP1	1.048	2.122	1.467	0.911	0.322	0.546	ectonucleotide pyrophosphatase/phosphodiesterase 1	ENPP1
21453	TGM2	7.475	2.122	4.522	0.193	0.332	0.346	transglutaminase 2	TGM2
1992	C11orf98	1.108	2.121	1.753	0.483	0.017	0.250	chromosome 11 open reading frame 98	C11orf98
8261	HMGB1	1.060	2.119	1.596	0.660	0.067	0.251	high mobility group box 1	HMGB1
13421	MGLL	1.204	2.118	1.320	0.788	0.205	0.582	monoglyceride lipase	MGLL
8849	ILF2	0.920	2.117	1.457	0.322	0.011	0.032	interleukin enhancer binding factor 2	ILF2
22683	TYRO3	0.709	2.116	1.060	0.230	0.020	0.823	TYRO3 protein tyrosine kinase	TYRO3
21313	TDP1	0.927	2.115	1.286	0.633	0.016	0.050	tyrosyl-DNA phosphodiesterase 1	TDP1
4073	CPT1A	0.668	2.112	1.166	0.085	0.024	0.425	carnitine palmitoyltransferase 1A	CPT1A
14422	NETO2	0.756	2.110	1.159	0.362	0.073	0.639	neuropilin and tolloid like 2	NETO2
12790	LSM2	0.640	2.106	1.553	0.134	0.015	0.145	LSM2 homolog, U6 small nuclear RNA and mRNA degradation associa	LSM2
617	ALDH7A1	0.463	2.106	1.130	0.021	0.061	0.155	aldehyde dehydrogenase 7 family member A1	ALDH7A1
12578	LOC730101	0.120	2.105	1.026	0.005	0.062	0.823	uncharacterized LOC730101	LOC730101
7172	GDNF	1.641	2.104	0.953	0.523	0.178	0.782	glial cell derived neurotrophic factor	GDNF
2355	C5orf34	1.034	2.103	1.006	0.738	0.005	0.922	chromosome 5 open reading frame 34	C5orf34
14305	NDE1	0.559	2.100	1.055	0.056	0.095	0.661	nudE neurodevelopment protein 1	NDE1
8704	IGF2	0.660	2.100	0.248	0.779	0.638	0.500	insulin like growth factor 2	IGF2
3786	CMSS1	1.118	2.099	1.379	0.517	0.021	0.452	cms1 ribosomal small subunit homolog (yeast)	CMSS1
1202	ARPC5L	1.000	2.099	1.583	0.999	0.015	0.145	actin related protein 2/3 complex subunit 5-like	ARPC5L
16272	PIN1	1.032	2.099	1.372	0.843	0.058	0.112	peptidylprolyl cis/trans isomerase, NIMA-interacting 1	PIN1
14799	NRIP3	1.386	2.097	1.527	0.473	0.074	0.117	nuclear receptor interacting protein 3	NRIP3
4016	COX7A2	1.330	2.096	1.938	0.086	0.000	0.280	cytochrome c oxidase subunit 7A2	COX7A2
3970	COPS6	0.875	2.096	1.932	0.260	0.005	0.053	COP9 signalosome subunit 6	COPS6
17293	PTMS	0.568	2.095	1.701	0.064	0.094	0.246	parathymosin	PTMS
8943	IPO11-LRRC70	1.000	2.095	1.998	0.900	0.003	0.029	IPO11-LRRC70 readthrough	IPO11-LRRC70
7638	GPSM2	0.544	2.095	1.077	0.258	0.100	0.905	G-protein signaling modulator 2	GPSM2
8257	HMG20B	0.632	2.093	1.244	0.021	0.080	0.380	high mobility group 20B	HMG20B
6929	FXYD5	0.823	2.089	1.674	0.428	0.025	0.191	FXYD domain containing ion transport regulator 5	FXYD5
16573	POLA1	0.805	2.089	1.060	0.130	0.007	0.616	polymerase (DNA) alpha 1, catalytic subunit	POLA1
23782	ZMYND10	0.613	2.088	1.550	0.292	0.000	0.216	zinc finger MYND-type containing 10	ZMYND10
3159	CDCA4	0.952	2.086	1.067	0.852	0.050	0.815	cell division cycle associated 4	CDCA4
8612	IDH2	0.875	2.078	1.399	0.825	0.177	0.316	isocitrate dehydrogenase (NADP(+)) 2, mitochondrial	IDH2
8844	IL7R	5.402	2.077	2.303	0.018	0.222	0.102	interleukin 7 receptor	IL7R
18139	RNF26	0.939	2.077	1.400	0.576	0.009	0.036	ring finger protein 26	RNF26
19462	SLC35F2	0.911	2.074	1.610	0.453	0.024	0.346	solute carrier family 35 member F2	SLC35F2
2470	C9orf24	1.026	2.073	1.272	0.845	0.046	0.137	chromosome 9 open reading frame 24	C9orf24
8310	HNRNPC	0.843	2.072	1.692	0.021	0.058	0.132	heterogeneous nuclear ribonucleoprotein C (C1/C2)	HNRNPC
6598	FIBCD1	0.920	2.072	1.755	0.405	0.419	0.477	fibrinogen C domain containing 1	FIBCD1
5922	EZR	0.797	2.072	1.340	0.337	0.032	0.330	ezrin	EZR
1656	BCL7C	0.593	2.072	1.625	0.236	0.047	0.332	B-cell CLL/lymphoma 7C	BCL7C
18698	SCG5	0.667	2.071	0.951	0.255	0.227	0.874	secretogranin V	SCG5
9875	LAMC2	1.154	2.071	11.100	0.364	0.219	0.324	laminin subunit gamma 2	LAMC2
2342	C4orf46	0.711	2.071	0.852	0.435	0.042	0.693	chromosome 4 open reading frame 46	C4orf46
1584	BANF1	0.790	2.070	1.593	0.112	0.004	0.180	barrier to autointegration factor 1	BANF1
4814	DEPDC7	0.660	2.069	1.260	0.226	0.214	0.524	DEP domain containing 7	DEPDC7
1627	BCAS4	0.703	2.069	1.241	0.258	0.058	0.152	breast carcinoma amplified sequence 4	BCAS4
9283	KCNMA1	0.598	2.068	2.050	0.129	0.088	0.007	potassium calcium-activated channel subfamily M alpha 1	KCNMA1
2459	C9orf142	0.737	2.065	1.374	0.293	0.043	0.220	chromosome 9 open reading frame 142	C9orf142
18603	SAPCD1-AS1	0.740	2.064	1.181	0.406	0.097	0.446	SAPCD1 antisense RNA 1	SAPCD1-AS1
6586	FGL2	0.762	2.062	1.311	0.503	0.425	0.616	fibrinogen like 2	FGL2
15672	PAM16	1.544	2.059	1.965	0.047	0.028	0.249	presequence translocase-associated motor 16 homolog (S. cerevisiae	PAM16
9545	KLF17	1.467	2.058	1.786	0.292	0.060	0.195	Kruppel-like factor 17	KLF17
14186	NANS	0.831	2.051	2.109	0.247	0.038	0.019	N-acetylneuraminate synthase	NANS
14593	NME2	0.543	2.048	1.527	0.085	0.016	0.335	NME/NM23 nucleoside diphosphate kinase 2	NME2
15729	PARP2	0.961	2.043	0.908	0.733	0.016	0.490	poly(ADP-ribose) polymerase 2	PARP2
9661	KRBOX1	1.025	2.042	1.829	0.867	0.016	0.009	KRAB box domain containing 1	KRBOX1
10268	LINC00467	0.844	2.042	1.863	0.648	0.157	0.391	long intergenic non-protein coding RNA 467	LINC00467
9657	KPTN	0.527	2.041	1.289	0.061	0.005	0.336	kaptin (actin binding protein)	KPTN
13729	MRPL12	1.181	2.040	2.002	0.438	0.115	0.195	mitochondrial ribosomal protein L12	MRPL12
19753	SMIM4	1.019	2.038	1.847	0.952	0.083	0.239	small integral membrane protein 4	SMIM4
13694	MRAP2	0.431	2.038	2.488	0.044	0.128	0.044	melanocortin 2 receptor accessory protein 2	MRAP2
18211	ROMO1	0.904	2.037	2.059	0.689	0.020	0.334	reactive oxygen species modulator 1	ROMO1
17196	PSMC3	1.023	2.036	1.600	0.795	0.008	0.236	proteasome 26S subunit, ATPase 3	PSMC3
17473	RAB30-AS1	1.410	2.036	1.834	0.437	0.194	0.309	RAB30 antisense RNA 1 (head to head)	RAB30-AS1
23509	YIF1B	1.096	2.033	1.753	0.588	0.093	0.007	Yip1 interacting factor homolog B, membrane trafficking protein	YIF1B
20626	SRSF2	1.062	2.032	1.840	0.751	0.048	0.022	serine/arginine-rich splicing factor 2	SRSF2
8252	HMBS	1.445	2.031	2.233	0.143	0.006	0.143	hydroxymethylbilane synthase	HMBS
5637	EMP2	0.664	2.030	0.978	0.118	0.055	0.928	epithelial membrane protein 2	EMP2
7219	GGT5	2.231	2.029	3.825	0.366	0.120	0.417	gamma-glutamyltransferase 5	GGT5
511	AHCY	0.595	2.029	1.422	0.215	0.016	0.386	adenosylhomocysteinase	AHCY
16584	POLE3	0.773	2.029	1.044	0.066	0.007	0.674	polymerase (DNA) epsilon 3, accessory subunit	POLE3
3180	CDH24	0.637	2.027	1.097	0.112	0.040	0.239	cadherin 24	CDH24
14931	NUP37	0.977	2.027	1.676	0.620	0.006	0.121	nucleoporin 37kDa	NUP37
3948	COMMD3	1.220	2.026	1.401	0.336	0.118	0.132	COMM domain containing 3	COMMD3
20233	SNRPC	1.010	2.026	1.815	0.903	0.001	0.127	small nuclear ribonucleoprotein polypeptide C	SNRPC
23308	WDR54	0.914	2.025	1.625	0.486	0.011	0.295	WD repeat domain 54	WDR54
14367	NDUFS6	0.998	2.022	1.769	0.993	0.010	0.355	NADH:ubiquinone oxidoreductase subunit S6	NDUFS6
16088	PFN1	0.918	2.021	1.815	0.598	0.020	0.162	profilin 1	PFN1
2797	CCDC134	1.438	2.021	2.036	0.079	0.002	0.261	coiled-coil domain containing 134	CCDC134
3444	CFL1	0.912	2.021	1.880	0.509	0.017	0.010	cofilin 1	CFL1
8309	HNRNPAB	1.214	2.020	1.373	0.208	0.024	0.216	heterogeneous nuclear ribonucleoprotein A/B	HNRNPAB
6871	FSD1	0.671	2.019	1.692	0.001	0.025	0.096	fibronectin type III and SPRY domain containing 1	FSD1
3682	CLEC11A	0.677	2.019	2.406	0.321	0.120	0.415	C-type lectin domain family 11 member A	CLEC11A
15722	PARP1	0.707	2.018	1.286	0.039	0.038	0.150	poly (ADP-ribose) polymerase 1	PARP1
16649	POP5	0.811	2.018	1.448	0.441	0.007	0.232	POP5 homolog, ribonuclease P/MRP subunit	POP5
14786	NREP	0.492	2.016	2.100	0.140	0.148	0.004	neuronal regeneration related protein	NREP
24210	ZNF695	0.923	2.016	1.429	0.331	0.008	0.296	zinc finger protein 695	ZNF695
14369	NDUFS8	1.185	2.015	1.704	0.181	0.115	0.071	NADH:ubiquinone oxidoreductase core subunit S8	NDUFS8
9973	LDHB	0.723	2.014	1.314	0.039	0.004	0.305	lactate dehydrogenase B	LDHB
2409	C7orf50	0.764	2.014	1.525	0.521	0.063	0.315	chromosome 7 open reading frame 50	C7orf50
16509	PMM1	0.534	2.013	1.300	0.051	0.022	0.206	phosphomannomutase 1	PMM1

20230	SNRPA1	1.713	2.013	2.182	0.033	0.057	0.010	small nuclear ribonucleoprotein polypeptide A'	SNRPA1
1589	BARD1	0.994	2.013	1.144	0.962	0.090	0.437	BRCA1 associated RING domain 1	BARD1
8516	HSPB11	1.424	2.011	1.467	0.054	0.003	0.069	heat shock protein family B (small) member 11	HSPB11
2629	CAPN12	0.786	2.010	1.600	0.431	0.081	0.242	calpain 12	CAPN12
18260	RPL13AP20	0.668	2.009	0.915	0.067	0.036	0.764	ribosomal protein L13a pseudogene 20	RPL13AP20
18400	RPSAP58	0.454	2.008	1.426	0.085	0.086	0.461	ribosomal protein SA pseudogene 58	RPSAP58
6075	FAM166A	0.908	2.007	1.260	0.436	0.011	0.100	family with sequence similarity 166 member A	FAM166A
14497	NHP2	0.991	2.007	1.887	0.926	0.023	0.115	NHP2 ribonucleoprotein	NHP2
14124	MZT2A	0.502	2.007	1.459	0.147	0.060	0.289	mitotic spindle organizing protein 2A	MZT2A
23273	WDHD1	1.168	2.006	1.116	0.613	0.055	0.620	WD repeat and HMG-box DNA binding protein 1	WDHD1
3162	CDCA7L	0.405	2.006	1.016	0.182	0.103	0.964	cell division cycle associated 7 like	CDCA7L
24234	ZNF714	0.886	2.002	1.035	0.194	0.213	0.864	zinc finger protein 714	ZNF714
6277	FAM72C	0.984	2.001	1.170	0.692	0.157	0.093	family with sequence similarity 72 member C	FAM72C
15013	ODF2	0.914	2.000	1.290	0.761	0.027	0.519	outer dense fiber of sperm tails 2	ODF2
5295	DUSP2	1.034	2.000	1.208	0.718	0.001	0.370	dual specificity phosphatase 2	DUSP2
13770	MRPL51	0.962	1.996	1.917	0.652	0.001	0.155	mitochondrial ribosomal protein L51	MRPL51
13728	MRPL11	0.800	1.996	1.812	0.217	0.012	0.268	mitochondrial ribosomal protein L11	MRPL11
21829	TMEM238	1.031	1.996	1.429	0.921	0.286	0.439	transmembrane protein 238	TMEM238
21109	TARBP2	0.816	1.994	1.605	0.196	0.015	0.275	TAR (HIV-1) RNA binding protein 2	TARBP2
23128	VIM	0.260	1.992	1.045	0.176	0.027	0.941	vimentin	VIM
2075	C16orf95	0.807	1.990	1.689	0.274	0.022	0.156	chromosome 16 open reading frame 95	C16orf95
1666	BCRP2	0.996	1.989	2.024	0.984	0.013	0.329	breakpoint cluster region pseudogene 2	BCRP2
8762	IL11	54.109	1.988	2.169	0.340	0.519	0.427	interleukin 11	IL11
7512	GPATCH4	1.116	1.988	2.011	0.260	0.010	0.250	G-patch domain containing 4	GPATCH4
12353	LOC388849	0.466	1.987	1.609	0.372	0.015	0.322	.	LOC388849
13753	MRPL37	0.803	1.985	1.746	0.069	0.011	0.045	mitochondrial ribosomal protein L37	MRPL37
8465	HSD17B10	1.220	1.980	1.867	0.209	0.023	0.303	hydroxysteroid (17-beta) dehydrogenase 10	HSD17B10
9772	KRTAP2-3	0.554	1.979	2.064	0.243	0.026	0.551	keratin associated protein 2-3	KRTAP2-3
7494	GOT2	0.945	1.979	1.600	0.384	0.009	0.080	glutamic-oxaloacetic transaminase 2	GOT2
5992	FAM110A	0.819	1.977	1.599	0.495	0.163	0.278	family with sequence similarity 110 member A	FAM110A
22088	TOMM40	1.499	1.977	1.582	0.003	0.161	0.008	translocase of outer mitochondrial membrane 40	TOMM40
1091	ARHGDIB	0.612	1.976	1.924	0.209	0.013	0.220	Rho GDP dissociation inhibitor beta	ARHGDIB
8178	HIST1H4A	0.869	1.976	1.117	0.424	0.293	0.718	histone cluster 1, H4a	HIST1H4A
12172	LOC10302129	0.719	1.975	1.158	0.739	0.217	0.904	uncharacterized LOC103021295	LOC103021295
1700	BFSP1	0.718	1.974	1.020	0.046	0.047	0.946	beaded filament structural protein 1	BFSP1
17550	RAD51C	0.795	1.973	1.048	0.431	0.022	0.899	RAD51 paralog C	RAD51C
21348	TELO2	0.941	1.973	1.553	0.717	0.017	0.235	telomere maintenance 2	TELO2
12724	LRRC45	0.655	1.973	1.118	0.273	0.010	0.729	leucine rich repeat containing 45	LRRC45
7111	GBAP1	0.723	1.971	1.252	0.346	0.042	0.351	glucosylceramidase beta pseudogene 1	GBAP1
3070	CD320	0.851	1.969	2.119	0.538	0.018	0.247	CD320 molecule	CD320
14335	NDUFA7	1.556	1.967	2.527	0.372	0.119	0.035	NADH:ubiquinone oxidoreductase subunit A7	NDUFA7
18550	S1PR3	1.923	1.964	1.975	0.176	0.054	0.247	sphingosine-1-phosphate receptor 3	S1PR3
20239	SNRPF	0.948	1.963	1.543	0.345	0.008	0.177	small nuclear ribonucleoprotein polypeptide F	SNRPF
2596	CAMK1	0.790	1.963	1.289	0.073	0.017	0.289	calcium/calmodulin dependent protein kinase I	CAMK1
5868	EVI2B	0.523	1.962	0.493	0.107	0.078	0.082	ecotropic viral integration site 2B	EVI2B
20955	SWI5	0.788	1.961	1.522	0.229	0.034	0.352	SWI5 homologous recombination repair protein	SWI5
23482	XRCC6	0.875	1.958	1.433	0.324	0.000	0.254	X-ray repair complementing defective repair in Chinese hamster cells	XRCC6
2927	CCHCR1	1.070	1.958	1.398	0.822	0.022	0.470	coiled-coil alpha-helical rod protein 1	CCHCR1
8526	HSPD1	1.102	1.958	1.596	0.570	0.010	0.214	heat shock protein family D (Hsp60) member 1	HSPD1
16757	PPM1G	0.969	1.957	1.650	0.823	0.001	0.404	protein phosphatase, Mg2+/Mn2+ dependent 1G	PPM1G
9559	KLHDC4	0.868	1.957	1.542	0.591	0.004	0.485	kelch domain containing 4	KLHDC4
17186	PSMB3	1.060	1.956	1.853	0.725	0.004	0.215	proteasome subunit beta 3	PSMB3
3255	CDRT4	0.843	1.955	1.476	0.859	0.297	0.538	CMT1A duplicated region transcript 4	CDRT4
2976	CCNE1	1.310	1.955	1.345	0.237	0.207	0.209	cyclin E1	CCNE1
18363	RPS21	0.544	1.953	1.331	0.033	0.011	0.370	ribosomal protein S21	RPS21
1077	ARHGAP33	0.672	1.952	0.968	0.330	0.048	0.914	Rho GTPase activating protein 33	ARHGAP33
7145	GCNT1	0.415	1.951	0.713	0.004	0.074	0.291	glucosaminyl (N-acetyl) transferase 1, core 2	GCNT1
23660	ZDHHC12	0.803	1.951	1.382	0.462	0.087	0.457	zinc finger DHHC-type containing 12	ZDHHC12
3237	CDKN2AIPNL	1.095	1.951	1.281	0.534	0.071	0.055	CDKN2A interacting protein N-terminal like	CDKN2AIPNL
8123	HINT2	1.066	1.949	1.988	0.482	0.065	0.163	histidine triad nucleotide binding protein 2	HINT2
13517	MIS18BP1	0.990	1.949	1.035	0.973	0.132	0.883	MIS18 binding protein 1	MIS18BP1
13772	MRPL53	0.763	1.949	1.978	0.172	0.032	0.211	mitochondrial ribosomal protein L53	MRPL53
20238	SNRPE	0.880	1.947	1.512	0.553	0.029	0.201	small nuclear ribonucleoprotein polypeptide E	SNRPE
14892	NUDT15	1.127	1.947	1.574	0.313	0.050	0.009	nudix hydrolase 15	NUDT15
3624	CKAP5	0.902	1.947	1.098	0.637	0.106	0.711	cytoskeleton associated protein 5	CKAP5
15740	PARVB	0.862	1.945	1.831	0.438	0.007	0.218	parvin beta	PARVB
993	APOLD1	1.253	1.944	1.259	0.209	0.085	0.328	apolipoprotein L domain containing 1	APOLD1
2815	CCDC150	0.835	1.944	0.980	0.011	0.024	0.482	coiled-coil domain containing 150	CCDC150
5907	EXOSC9	1.580	1.943	1.840	0.105	0.032	0.406	exosome component 9	EXOSC9
14919	NUP107	1.057	1.943	1.112	0.603	0.019	0.324	nucleoporin 107kDa	NUP107
12852	LYAR	1.406	1.942	1.503	0.017	0.119	0.050	Ly1 antibody reactive	LYAR
22218	TRAP1	0.927	1.942	1.533	0.626	0.101	0.062	TNF receptor associated protein 1	TRAP1
14282	NCLN	1.242	1.941	1.481	0.270	0.050	0.124	nicalin	NCLN
5956	FABP4	0.193	1.939	0.813	0.445	0.413	0.874	fatty acid binding protein 4	FABP4
8683	IFT122	0.728	1.939	1.428	0.075	0.061	0.411	intraflagellar transport 122	IFT122
20240	SNRPG	1.443	1.937	1.699	0.187	0.032	0.046	small nuclear ribonucleoprotein polypeptide G	SNRPG
25	AAMDC	0.601	1.936	1.337	0.251	0.217	0.620	adipogenesis associated, Mth938 domain containing	AAMDC
7506	GPAT2	0.764	1.936	1.390	0.280	0.062	0.117	glycerol-3-phosphate acyltransferase 2, mitochondrial	GPAT2
18303	RPL35	0.692	1.936	1.448	0.003	0.018	0.320	ribosomal protein L35	RPL35
6600	FIBP	0.797	1.936	1.644	0.148	0.007	0.123	FGF1 intracellular binding protein	FIBP
6446	FBXO43	1.045	1.935	1.314	0.036	0.006	0.129	F-box protein 43	FBXO43
16719	PPDPF	0.615	1.935	1.293	0.394	0.119	0.454	pancreatic progenitor cell differentiation and proliferation factor	PPDPF
23573	ZBTB16	0.728	1.935	1.345	0.365	0.187	0.403	zinc finger and BTB domain containing 16	ZBTB16
18322	RPL8	0.520	1.935	1.438	0.144	0.024	0.464	ribosomal protein L8	RPL8
15607	PA2G4	1.110	1.933	1.462	0.417	0.086	0.074	proliferation-associated 2G4	PA2G4
264	ACYP1	2.155	1.932	1.816	0.018	0.103	0.462	acylphosphatase 1	ACYP1
20938	SUV39H2	1.011	1.932	1.195	0.935	0.024	0.120	suppressor of variegation 3-9 homolog 2	SUV39H2
1849	BRIP1	1.057	1.929	1.164	0.609	0.021	0.258	BRCA1 interacting protein C-terminal helicase 1	BRIP1
20117	SNORD22	9.471	1.928	15.005	0.423	0.425	0.423	small nucleolar RNA, C/D box 22	SNORD22
14753	NR1H3	0.516	1.927	1.277	0.019	0.043	0.659	nuclear receptor subfamily 1 group H member 3	NR1H3
2814	CCDC15	0.966	1.926	1.166	0.625	0.107	0.278	coiled-coil domain containing 15	CCDC15

7927	HAS1	1.333	1.926	1.368	0.398	0.102	0.510	hyaluronan synthase 1	HAS1
3334	CENPL	0.883	1.926	1.098	0.637	0.015	0.787	centromere protein L	CENPL
14366	NDUFS5	0.978	1.924	1.708	0.903	0.166	0.149	NADH:ubiquinone oxidoreductase subunit S5	NDUFS5
7935	HAUS4	0.491	1.923	1.065	0.033	0.018	0.557	HAUS augmin like complex subunit 4	HAUS4
4450	CYFIP2	0.914	1.923	1.093	0.679	0.269	0.396	cytoplasmic FMR1 interacting protein 2	CYFIP2
19046	SGOL1-AS1	0.975	1.923	1.456	0.867	0.173	0.055	.	SGOL1-AS1
14904	NUDT4P1	1.002	1.923	1.001	0.161	0.423	0.317	nudix hydrolase 4 pseudogene 1	NUDT4P1
1535	B3GNTL1	0.870	1.922	1.305	0.052	0.005	0.146	UDP-GlcNAc:betaGal beta-1,3-N-acetylglucosaminyltransferase-like 1	B3GNTL1
1999	C12orf45	0.675	1.922	1.205	0.291	0.004	0.511	chromosome 12 open reading frame 45	C12orf45
21180	TBC1D31	0.942	1.922	1.102	0.691	0.048	0.391	TBC1 domain family member 31	TBC1D31
17996	RIN1	0.443	1.920	1.518	0.006	0.100	0.035	Ras and Rab interactor 1	RIN1
5450	EFHD2	0.921	1.920	1.915	0.750	0.070	0.135	EF-hand domain family member D2	EFHD2
14839	NT5C	0.787	1.919	1.598	0.068	0.066	0.184	5', 3'-nucleotidase, cytosolic	NT5C
13771	MRPL52	1.075	1.919	1.917	0.754	0.093	0.134	mitochondrial ribosomal protein L52	MRPL52
6150	FAM207A	0.982	1.918	2.034	0.920	0.007	0.378	family with sequence similarity 207 member A	FAM207A
22605	TUBA4A	1.245	1.917	1.241	0.104	0.241	0.410	tubulin alpha 4a	TUBA4A
16075	PFAS	1.066	1.916	1.626	0.599	0.023	0.158	phosphoribosylformylglycinamidine synthase	PFAS
3481	CHCHD6	0.686	1.915	1.247	0.145	0.029	0.290	coiled-coil-helix-coiled-coil-helix domain containing 6	CHCHD6
862	ANP32B	0.687	1.914	1.307	0.196	0.119	0.533	acidic nuclear phosphoprotein 32 family member B	ANP32B
3320	CEMIP	0.315	1.912	0.733	0.091	0.456	0.185	cell migration inducing hyaluronan binding protein	CEMIP
9035	ISYNA1	0.399	1.911	1.247	0.123	0.209	0.393	inositol-3-phosphate synthase 1	ISYNA1
21593	TLE6	1.004	1.910	2.629	0.982	0.271	0.306	transducin like enhancer of split 6	TLE6
21390	TEX30	1.374	1.909	1.183	0.227	0.089	0.270	testis expressed 30	TEX30
7936	HAUS5	0.759	1.909	1.098	0.110	0.040	0.479	HAUS augmin like complex subunit 5	HAUS5
22276	TRIM28	0.812	1.906	1.369	0.215	0.021	0.166	tripartite motif containing 28	TRIM28
12120	LOC10272409	1.150	1.905	3.265	0.424	0.039	0.003	uncharacterized LOC102724094	LOC102724094
20597	SRM	1.216	1.905	2.233	0.139	0.192	0.008	spermidine synthase	SRM
2110	C18orf54	0.850	1.904	1.026	0.370	0.018	0.871	chromosome 18 open reading frame 54	C18orf54
6698	FLOT1	0.798	1.904	1.792	0.247	0.026	0.152	flotillin 1	FLOT1
4654	DCTPP1	0.725	1.903	1.254	0.180	0.072	0.257	dCTP pyrophosphatase 1	DCTPP1
16585	POLE4	0.666	1.900	1.759	0.283	0.013	0.271	polymerase (DNA) epsilon 4, accessory subunit	POLE4
13805	MRPS7	1.112	1.899	1.935	0.369	0.028	0.120	mitochondrial ribosomal protein S7	MRPS7
13800	MRPS34	0.754	1.899	1.452	0.010	0.022	0.111	mitochondrial ribosomal protein S34	MRPS34
16122	PGM2	0.874	1.897	1.140	0.684	0.033	0.713	phosphoglucomutase 2	PGM2
22628	TUFM	0.788	1.896	1.492	0.152	0.003	0.267	Tu translation elongation factor, mitochondrial	TUFM
23143	VMO1	3.578	1.896	21.835	0.315	0.437	0.427	vitelline membrane outer layer 1 homolog	VMO1
12818	LTV1	1.042	1.896	1.421	0.695	0.004	0.393	LTV1 ribosome biogenesis factor	LTV1
2411	C7orf55-LUC7	0.752	1.896	0.920	0.339	0.204	0.756	C7orf55-LUC7L2 readthrough	C7orf55-LUC7L2
12736	LRRC59	1.152	1.896	1.778	0.263	0.007	0.026	leucine rich repeat containing 59	LRRC59
15743	PASK	0.823	1.895	0.934	0.382	0.129	0.722	PAS domain containing serine/threonine kinase	PASK
14854	NTHL1	0.895	1.893	1.873	0.533	0.013	0.173	nth-like DNA glycosylase 1	NTHL1
13325	METRN	0.702	1.890	1.455	0.519	0.132	0.303	meteorin, glial cell differentiation regulator	METRN
13889	MTBP	0.946	1.890	0.989	0.766	0.004	0.962	MDM2 binding protein	MTBP
19201	SKA2	1.253	1.890	1.489	0.002	0.136	0.000	spindle and kinetochore associated complex subunit 2	SKA2
18365	RPS24	0.479	1.889	1.321	0.036	0.041	0.387	ribosomal protein S24	RPS24
21154	TBC1D1	0.808	1.889	1.231	0.602	0.188	0.476	TBC1 domain family member 1	TBC1D1
614	ALDH4A1	0.457	1.888	1.033	0.008	0.172	0.835	aldehyde dehydrogenase 4 family member A1	ALDH4A1
6593	FHL2	0.772	1.887	1.674	0.415	0.085	0.013	four and a half LIM domains 2	FHL2
3589	CHURC1-FNTB	0.764	1.885	1.002	0.202	0.019	0.995	CHURC1-FNTB readthrough	CHURC1-FNTB
6626	FKBP3	1.068	1.885	1.467	0.342	0.009	0.011	FK506 binding protein 3	FKBP3
1417	ATP5L	0.894	1.884	1.405	0.438	0.018	0.424	ATP synthase, H+ transporting, mitochondrial Fo complex subunit G	ATP5L
239	ACTN4	0.804	1.882	1.319	0.113	0.012	0.042	actinin alpha 4	ACTN4
3161	CDCA7	0.807	1.881	1.470	0.362	0.043	0.182	cell division cycle associated 7	CDCA7
17312	PTPN22	0.986	1.879	1.605	0.976	0.353	0.367	protein tyrosine phosphatase, non-receptor type 22	PTPN22
2789	CCDC124	0.934	1.878	1.616	0.505	0.012	0.374	coiled-coil domain containing 124	CCDC124
8094	HHIP-AS1	0.697	1.878	0.952	0.609	0.105	0.949	HHIP antisense RNA 1	HHIP-AS1
22935	UQCRQ	1.025	1.878	2.018	0.877	0.016	0.155	ubiquinol-cytochrome c reductase complex III subunit VII	UQCRQ
2065	C16orf74	0.457	1.878	0.757	0.121	0.102	0.555	chromosome 16 open reading frame 74	C16orf74
2981	CCNH	1.410	1.877	1.214	0.113	0.034	0.342	cyclin H	CCNH
22711	UBALD2	1.271	1.876	1.418	0.661	0.323	0.434	UBA like domain containing 2	UBALD2
16519	PMS2P2	0.799	1.876	1.004	0.657	0.152	0.992	PMS1 homolog 2, mismatch repair system component pseudogene 2	PMS2P2
19642	SLIRP	1.406	1.875	1.771	0.014	0.017	0.012	SRA stem-loop interacting RNA binding protein	SLIRP
13745	MRPL28	0.891	1.875	2.009	0.560	0.011	0.289	mitochondrial ribosomal protein L28	MRPL28
5018	DNA2	0.782	1.875	1.004	0.110	0.011	0.958	DNA replication helicase/nuclease 2	DNA2
14370	NDUFV1	0.698	1.871	1.743	0.339	0.014	0.438	NADH dehydrogenase (ubiquinone) flavoprotein 1, 51kDa	NDUFV1
9324	KCTD17	1.032	1.870	1.909	0.802	0.012	0.272	potassium channel tetramerization domain containing 17	KCTD17
13422	MGME1	0.936	1.870	1.127	0.846	0.099	0.581	mitochondrial genome maintenance exonuclease 1	MGME1
21571	TIPIN	1.196	1.869	1.523	0.158	0.003	0.142	TIMELESS interacting protein	TIPIN
7292	GLA	1.342	1.869	1.136	0.216	0.041	0.543	galactosidase alpha	GLA
14134	NAA10	0.830	1.868	1.519	0.248	0.076	0.139	N(alpha)-acetyltransferase 10, NatA catalytic subunit	NAA10
7541	GPN1	1.035	1.868	1.259	0.841	0.012	0.299	GPN-loop GTPase 1	GPN1
18592	SAMM50	0.698	1.868	1.292	0.080	0.050	0.131	SAMM50 sorting and assembly machinery component	SAMM50
8897	INO80E	0.785	1.867	1.334	0.238	0.024	0.250	INO80 complex subunit E	INO80E
8271	HMGN1	0.889	1.866	1.283	0.557	0.073	0.069	high mobility group nucleosome binding domain 1	HMGN1
16744	PPIL1	1.526	1.866	1.528	0.126	0.095	0.127	peptidylprolyl isomerase like 1	PPIL1
23504	YDJC	0.595	1.866	1.177	0.187	0.037	0.479	YdjC homolog (bacterial)	YDJC
17281	PTGS1	0.547	1.866	1.969	0.219	0.520	0.502	prostaglandin-endoperoxide synthase 1	PTGS1
18319	RPL7	0.349	1.863	1.023	0.039	0.067	0.950	ribosomal protein L7	RPL7
13265	MED27	0.776	1.863	1.442	0.292	0.033	0.115	mediator complex subunit 27	MED27
16142	PHB	1.037	1.862	1.625	0.252	0.002	0.140	prohibitin	PHB
8945	IPO4	0.917	1.861	1.575	0.283	0.065	0.009	importin 4	IPO4
14261	NCAPH2	1.010	1.861	1.432	0.939	0.036	0.218	non-SMC condensin II complex subunit H2	NCAPH2
17658	RASSF1	0.805	1.861	1.132	0.518	0.154	0.626	Ras association (RalGDS/AF-6) domain family member 1	RASSF1
2899	CCDC77	1.070	1.860	1.163	0.217	0.051	0.103	coiled-coil domain containing 77	CCDC77
18435	RRP9	1.309	1.860	1.951	0.387	0.049	0.407	ribosomal RNA processing 9, small subunit (SSU) processome compor	RRP9
7925	HARS	0.811	1.860	1.441	0.007	0.030	0.265	histidyl-tRNA synthetase	HARS
2559	CACYBP	0.730	1.860	0.912	0.145	0.008	0.706	calcyclin binding protein	CACYBP
15638	PAF1	0.835	1.859	1.426	0.165	0.019	0.407	PAF1 homolog, Paf1/RNA polymerase II complex component	PAF1
17777	RCC1	0.776	1.859	1.080	0.108	0.028	0.401	regulator of chromosome condensation 1	RCC1
3021	CCT7	1.006	1.858	1.548	0.967	0.003	0.281	chaperonin containing TCP1 subunit 7	CCT7
14882	NUDC	1.029	1.856	1.559	0.885	0.012	0.415	nudC nuclear distribution protein	NUDC

19224	SLAMF9	0.937	1.855	1.305	0.324	0.149	0.032	SLAM family member 9	SLAMF9
3945	COMMD1	0.694	1.854	1.443	0.039	0.015	0.395	copper metabolism domain containing 1	COMMD1
18323	RPL9	0.451	1.854	1.297	0.072	0.054	0.577	ribosomal protein L9	RPL9
12473	LOC642846	1.152	1.854	1.352	0.242	0.009	0.307	DEAD/H (Asp-Glu-Ala-Asp/His) box polypeptide 11-like	LOC642846
18306	RPL36A	0.481	1.852	1.150	0.024	0.022	0.607	ribosomal protein L36a	RPL36A
8292	HN1L	0.819	1.851	1.097	0.032	0.001	0.268	hematological and neurological expressed 1-like	HN1L
1844	BRI3BP	0.932	1.851	0.938	0.297	0.089	0.845	BRI3 binding protein	BRI3BP
3374	CEP72	1.006	1.851	1.140	0.948	0.050	0.551	centrosomal protein 72	CEP72
5902	EXOSC4	0.974	1.851	1.709	0.912	0.076	0.399	exosome component 4	EXOSC4
8425	HRAS	0.703	1.850	1.325	0.190	0.032	0.305	Harvey rat sarcoma viral oncogene homolog	HRAS
4448	CYCSP52	0.811	1.850	0.811	0.423	0.001	0.423	cytochrome c, somatic pseudogene 52	CYCSP52
21719	TMEM141	0.873	1.849	1.841	0.474	0.005	0.192	transmembrane protein 141	TMEM141
22008	TNFRSF12A	1.533	1.847	1.226	0.442	0.329	0.637	tumor necrosis factor receptor superfamily member 12A	TNFRSF12A
3016	CCT5	1.286	1.846	1.527	0.199	0.018	0.003	chaperonin containing TCP1 subunit 5	CCT5
23539	YWHAH	0.791	1.846	1.272	0.009	0.045	0.446	tyrosine 3-monooxygenase/tryptophan 5-monooxygenase activation	YWHAH
18289	RPL26L1	1.079	1.845	1.512	0.767	0.038	0.268	ribosomal protein L26 like 1	RPL26L1
20795	STK32A	1.041	1.845	2.861	0.904	0.464	0.477	serine/threonine kinase 32A	STK32A
18253	RPL10A	0.434	1.845	1.126	0.026	0.017	0.586	ribosomal protein L10a	RPL10A
720	ANAPC11	0.888	1.845	1.419	0.296	0.004	0.108	anaphase promoting complex subunit 11	ANAPC11
6257	FAM65B	2.129	1.845	3.131	0.222	0.562	0.466	family with sequence similarity 65 member B	FAM65B
6652	FLJ22447	1.365	1.845	2.420	0.515	0.415	0.075	uncharacterized LOC400221	FLJ22447
16614	POLR2L	0.591	1.844	1.711	0.180	0.054	0.159	polymerase (RNA) II subunit L	POLR2L
17530	RABL6	0.757	1.843	1.371	0.263	0.000	0.388	RAB, member RAS oncogene family-like 6	RABL6
21478	THBS1	2.448	1.842	1.509	0.187	0.211	0.355	thrombospondin 1	THBS1
1406	ATP5E	0.794	1.842	1.567	0.001	0.003	0.233	ATP synthase, H+ transporting, mitochondrial F1 complex, epsilon sul	ATP5E
21256	TCEB2	0.732	1.841	1.770	0.277	0.043	0.425	transcription elongation factor B subunit 2	TCEB2
16845	PPP5C	0.802	1.841	1.032	0.416	0.002	0.900	protein phosphatase 5 catalytic subunit	PPP5C
5539	EIF4A1	1.240	1.841	2.135	0.018	0.014	0.162	eukaryotic translation initiation factor 4A1	EIF4A1
9907	LAS1L	0.938	1.841	1.359	0.695	0.009	0.426	LAS1-like, ribosome biogenesis factor	LAS1L
22005	TNFRSF10D	0.654	1.839	1.071	0.350	0.007	0.828	tumor necrosis factor receptor superfamily member 10d	TNFRSF10D
13855	MSRB1	0.848	1.839	1.253	0.131	0.011	0.022	methionine sulfoxide reductase B1	MSRB1
14350	NDUFB2	0.889	1.839	1.597	0.089	0.054	0.042	NADH:ubiquinone oxidoreductase subunit B2	NDUFB2
21428	TFPT	0.901	1.839	1.746	0.487	0.036	0.397	TCF3 (E2A) fusion partner (in childhood Leukemia)	TFPT
16054	PET100	0.864	1.839	1.418	0.345	0.019	0.465	PET100 homolog	PET100
19957	SNORA8	1.579	1.838	0.667	0.574	0.507	0.772	small nucleolar RNA, H/ACA box 8	SNORA8
20627	SRSF3	0.982	1.837	1.579	0.908	0.032	0.081	serine/arginine-rich splicing factor 3	SRSF3
9667	KRI1	0.821	1.837	1.592	0.472	0.020	0.460	KRI1 homolog	KRI1
14142	NAA38	0.935	1.837	1.492	0.611	0.010	0.301	N(alpha)-acetyltransferase 38, NatC auxiliary subunit	NAA38
14993	OAZ3	0.798	1.837	1.604	0.475	0.020	0.185	ornithine decarboxylase antizyme 3	OAZ3
22156	TPM3	0.787	1.836	1.406	0.212	0.010	0.161	tropomyosin 3	TPM3
20309	SORD	0.729	1.836	1.361	0.359	0.092	0.121	sorbitol dehydrogenase	SORD
1961	C11orf31	0.695	1.835	1.484	0.048	0.208	0.015	chromosome 11 open reading frame 31	C11orf31
20660	SSRP1	1.004	1.835	1.623	0.967	0.009	0.265	structure specific recognition protein 1	SSRP1
5383	ECHS1	0.860	1.833	1.449	0.127	0.020	0.018	enoyl-CoA hydratase, short chain, 1, mitochondrial	ECHS1
21509	THRIL	1.028	1.832	0.742	0.857	0.065	0.393	TNF and HNRNPL related immunoregulatory long non-coding RNA	THRIL
1151	ARL2	0.518	1.831	1.277	0.044	0.054	0.103	ADP ribosylation factor like GTPase 2	ARL2
15597	P3H2	2.662	1.830	2.582	0.009	0.148	0.245	prolyl 3-hydroxylase 2	P3H2
15565	OXCT1	0.610	1.830	0.838	0.466	0.112	0.745	3-oxoacid CoA-transferase 1	OXCT1
8634	IFI27L2	0.747	1.829	1.664	0.169	0.014	0.303	interferon alpha inducible protein 27 like 2	IFI27L2
877	ANXA2P2	0.499	1.828	1.580	0.111	0.056	0.114	annexin A2 pseudogene 2	ANXA2P2
3014	CCT3	0.878	1.827	1.461	0.034	0.011	0.096	chaperonin containing TCP1 subunit 3	CCT3
3234	CDKN2A	0.604	1.827	1.238	0.234	0.063	0.445	cyclin-dependent kinase inhibitor 2A	CDKN2A
6730	FN3KRP	0.753	1.826	1.103	0.279	0.095	0.690	fructosamine 3 kinase related protein	FN3KRP
944	APEX2	0.988	1.825	1.424	0.939	0.055	0.173	apurinic/apyrimidinic endodeoxyribonuclease 2	APEX2
22481	TSSC1	1.235	1.825	1.696	0.360	0.041	0.087	tumor suppressing subtransferable candidate 1	TSSC1
5768	ERCC2	0.919	1.825	1.762	0.839	0.136	0.294	excision repair cross-complementation group 2	ERCC2
15720	PARM1	1.773	1.825	1.042	0.333	0.531	0.752	prostate androgen-regulated mucin-like protein 1	PARM1
7181	GEMIN2	0.751	1.822	1.029	0.123	0.035	0.833	gem nuclear organelle associated protein 2	GEMIN2
2799	CCDC137	0.988	1.821	1.515	0.857	0.050	0.174	coiled-coil domain containing 137	CCDC137
4166	CRYBB2	0.607	1.821	1.164	0.420	0.448	0.850	crystallin beta B2	CRYBB2
22665	TXNDC17	0.853	1.821	1.499	0.183	0.008	0.015	thioredoxin domain containing 17	TXNDC17
13226	MDH2	0.986	1.820	1.795	0.856	0.009	0.145	malate dehydrogenase 2	MDH2
16300	PITPNC1	1.131	1.820	1.674	0.747	0.001	0.251	phosphatidylinositol transfer protein, cytoplasmic 1	PITPNC1
16625	POLR3K	1.185	1.820	1.099	0.185	0.123	0.610	polymerase (RNA) III subunit K	POLR3K
21556	TIMM50	0.986	1.820	1.515	0.927	0.034	0.162	translocase of inner mitochondrial membrane 50	TIMM50
14940	NUP93	1.036	1.818	1.376	0.846	0.028	0.076	nucleoporin 93kDa	NUP93
13571	MMD	1.690	1.818	2.001	0.040	0.352	0.390	monocyte to macrophage differentiation associated	MMD
18295	RPL3	0.360	1.817	1.063	0.035	0.090	0.833	ribosomal protein L3	RPL3
22930	UQCRC1	1.377	1.816	2.132	0.155	0.011	0.298	ubiquinol-cytochrome c reductase core protein I	UQCRC1
8307	HNRNPA3	0.991	1.815	1.645	0.967	0.134	0.306	heterogeneous nuclear ribonucleoprotein A3	HNRNPA3
4931	DIXDC1	0.680	1.815	0.966	0.266	0.416	0.860	DIX domain containing 1	DIXDC1
6366	FARSB	1.105	1.815	1.578	0.521	0.024	0.049	phenylalanyl-tRNA synthetase beta subunit	FARSB
17520	RABEPK	0.708	1.815	1.303	0.312	0.011	0.364	Rab9 effector protein with kelch motifs	RABEPK
4650	DCTN3	0.706	1.814	1.598	0.107	0.061	0.065	dynactin subunit 3	DCTN3
13237	MEA1	1.045	1.813	1.342	0.648	0.070	0.032	male-enhanced antigen 1	MEA1
17207	PSMD2	1.129	1.813	1.298	0.143	0.004	0.031	proteasome 26S subunit, non-ATPase 2	PSMD2
20772	STEAP1B	1.114	1.813	1.279	0.800	0.045	0.621	STEAP family member 1B	STEAP1B
9859	LAGE3	0.815	1.813	1.461	0.520	0.012	0.365	L antigen family member 3	LAGE3
9055	ITGAE	0.819	1.812	1.278	0.148	0.015	0.363	integrin subunit alpha E	ITGAE
19834	SNF8	0.840	1.812	1.628	0.185	0.003	0.163	SNF8, ESCRT-II complex subunit	SNF8
4125	CRIP2	0.344	1.812	1.239	0.018	0.029	0.594	cysteine rich protein 2	CRIP2
19281	SLC17A9	2.196	1.811	2.080	0.038	0.224	0.135	solute carrier family 17 member 9	SLC17A9
5906	EXOSC8	1.168	1.811	1.187	0.453	0.039	0.225	exosome component 8	EXOSC8
7543	GPN3	1.160	1.811	1.020	0.279	0.015	0.850	GPN-loop GTPase 3	GPN3
21636	TMA7	0.773	1.811	1.351	0.160	0.026	0.250	translation machinery associated 7 homolog	TMA7
13279	MEF2BNB	1.022	1.811	1.602	0.938	0.103	0.130	.	MEF2BNB
5379	ECH1	0.873	1.810	1.337	0.209	0.010	0.161	enoyl-CoA hydratase 1, peroxisomal	ECH1
16529	PNKP	0.811	1.809	1.542	0.353	0.037	0.274	polynucleotide kinase 3'-phosphatase	PNKP
20675	SSX2IP	0.428	1.809	1.061	0.269	0.048	0.931	synovial sarcoma, X breakpoint 2 interacting protein	SSX2IP
1415	ATP5J2	1.155	1.809	1.877	0.329	0.010	0.048	ATP synthase, H+ transporting, mitochondrial Fo complex subunit F2	ATP5J2
8151	HIST1H2AM	1.284	1.809	1.478	0.526	0.220	0.375	histone cluster 1, H2am	HIST1H2AM

16916	PRDX2	1.060	1.808	1.575	0.549	0.060	0.222	peroxiredoxin 2	PRDX2
11176	LOC10013398	0.824	1.808	1.301	0.376	0.020	0.312	uncharacterized LOC100133985	LOC100133985
20548	SPSB2	0.682	1.805	1.202	0.004	0.110	0.456	splA/ryanodine receptor domain and SOCS box containing 2	SPSB2
12550	LOC728554	0.846	1.805	1.235	0.322	0.075	0.024	THO complex 3 pseudogene	LOC728554
14881	NUCKS1	0.801	1.805	1.079	0.203	0.176	0.364	nuclear casein kinase and cyclin-dependent kinase substrate 1	NUCKS1
412	ADORA1	0.407	1.805	1.058	0.038	0.014	0.780	adenosine A1 receptor	ADORA1
13665	MPHOSPH6	1.472	1.804	1.270	0.234	0.057	0.240	M-phase phosphoprotein 6	MPHOSPH6
13754	MRPL38	0.965	1.803	1.836	0.878	0.008	0.209	mitochondrial ribosomal protein L38	MRPL38
1412	ATP5H	1.107	1.803	1.338	0.291	0.058	0.008	ATP synthase, H+ transporting, mitochondrial Fo complex subunit D	ATP5H
20235	SNRPD2	0.736	1.802	1.391	0.022	0.008	0.431	small nuclear ribonucleoprotein D2 polypeptide	SNRPD2
3215	CDK5	0.892	1.802	1.664	0.527	0.171	0.020	cyclin-dependent kinase 5	CDK5
18375	RPS3	0.407	1.802	1.070	0.122	0.021	0.863	ribosomal protein S3	RPS3
10976	LINC01604	0.488	1.802	1.662	0.011	0.274	0.339	.	LINC01604
8320	HNRNPH3	1.149	1.801	1.468	0.134	0.048	0.195	heterogeneous nuclear ribonucleoprotein H3	HNRNPH3
17236	PSTPIP2	1.035	1.799	1.349	0.891	0.178	0.428	proline-serine-threonine phosphatase interacting protein 2	PSTPIP2
20631	SRSF7	1.008	1.799	1.287	0.954	0.044	0.054	serine/arginine-rich splicing factor 7	SRSF7
12908	MAD1L1	0.721	1.799	1.118	0.195	0.012	0.772	MAD1 mitotic arrest deficient like 1	MAD1L1
13991	MUTYH	0.882	1.797	1.295	0.640	0.061	0.420	mutY DNA glycosylase	MUTYH
18397	RPS9	0.449	1.797	1.201	0.253	0.100	0.787	ribosomal protein S9	RPS9
7752	GSS	1.071	1.797	1.724	0.681	0.022	0.026	glutathione synthetase	GSS
2974	CCND3	0.997	1.796	1.303	0.981	0.093	0.099	cyclin D3	CCND3
16900	PRDM1	1.563	1.796	1.131	0.144	0.033	0.263	PR domain 1	PRDM1
20507	SPOCD1	0.900	1.796	2.586	0.161	0.002	0.009	SPOC domain containing 1	SPOCD1
866	ANPEP	1.838	1.795	2.921	0.231	0.104	0.015	alanyl aminopeptidase, membrane	ANPEP
12780	LRWD1	1.161	1.793	1.388	0.480	0.113	0.120	leucine-rich repeats and WD repeat domain containing 1	LRWD1
8527	HSPE1	1.316	1.793	1.409	0.174	0.139	0.135	heat shock protein family E (Hsp10) member 1	HSPE1
20621	SRRT	0.873	1.792	1.243	0.395	0.025	0.484	serrate, RNA effector molecule	SRRT
10537	LINC00982	0.719	1.792	1.556	0.288	0.185	0.489	long intergenic non-protein coding RNA 982	LINC00982
22933	UQCRH	0.703	1.792	1.369	0.143	0.020	0.525	ubiquinol-cytochrome c reductase hinge protein	UQCRH
158	ACD	0.689	1.791	1.039	0.079	0.002	0.738	adrenocortical dysplasia homolog	ACD
17199	PSMC5	0.920	1.791	1.650	0.445	0.005	0.305	proteasome 26S subunit, ATPase 5	PSMC5
1924	BZW2	0.696	1.791	1.588	0.010	0.033	0.004	basic leucine zipper and W2 domains 2	BZW2
22694	U2AF2	0.973	1.790	1.402	0.783	0.027	0.033	U2 small nuclear RNA auxiliary factor 2	U2AF2
16608	POLR2I	0.876	1.790	1.516	0.134	0.083	0.411	polymerase (RNA) II subunit I	POLR2I
3468	CHAC2	1.025	1.790	1.067	0.881	0.130	0.703	ChaC cation transport regulator homolog 2	CHAC2
8122	HINT1	0.676	1.788	1.059	0.046	0.030	0.822	histidine triad nucleotide binding protein 1	HINT1
9097	ITPRIP	3.408	1.788	2.969	0.195	0.177	0.272	inositol 1,4,5-trisphosphate receptor interacting protein	ITPRIP
18558	SAAL1	0.829	1.786	1.099	0.031	0.001	0.093	serum amyloid A like 1	SAAL1
18370	RPS27A	0.515	1.785	1.198	0.084	0.032	0.489	ribosomal protein S27a	RPS27A
2150	C1QL1	0.893	1.785	1.384	0.734	0.333	0.002	complement component 1, q subcomponent-like 1	C1QL1
17955	RHOC	0.790	1.784	1.597	0.226	0.028	0.072	ras homolog family member C	RHOC
6756	FOSL1	1.677	1.784	1.557	0.469	0.274	0.489	FOS like 1, AP-1 trancription factor subunit	FOSL1
16730	PPIA	0.819	1.783	1.433	0.343	0.020	0.093	peptidylprolyl isomerase A	PPIA
2359	C5orf46	1.163	1.781	1.299	0.423	0.490	0.422	chromosome 5 open reading frame 46	C5orf46
18376	RPS3A	0.393	1.781	1.065	0.094	0.092	0.852	ribosomal protein S3A	RPS3A
5418	EEF1B2	0.548	1.780	1.319	0.006	0.009	0.184	eukaryotic translation elongation factor 1 beta 2	EEF1B2
8861	IMPA2	1.363	1.779	1.798	0.389	0.116	0.249	inositol monophosphatase 2	IMPA2
13138	MAZ	0.784	1.778	1.431	0.121	0.004	0.045	MYC associated zinc finger protein	MAZ
7372	GMPPB	1.193	1.778	1.287	0.445	0.035	0.388	GDP-mannose pyrophosphorylase B	GMPPB
23338	WDR91	1.102	1.777	1.215	0.651	0.120	0.497	WD repeat domain 91	WDR91
8778	IL17D	0.529	1.776	0.791	0.228	0.453	0.700	interleukin 17D	IL17D
19682	SMAD9	0.490	1.776	0.951	0.053	0.441	0.798	SMAD family member 9	SMAD9
5423	EEF1G	0.384	1.776	1.057	0.097	0.068	0.879	eukaryotic translation elongation factor 1 gamma	EEF1G
14930	NUP35	1.680	1.775	1.521	0.128	0.038	0.065	nucleoporin 35kDa	NUP35
19730	SMG9	0.622	1.775	1.153	0.067	0.210	0.157	SMG9 nonsense mediated mRNA decay factor	SMG9
14621	NOC2L	1.252	1.775	1.945	0.278	0.003	0.300	NOC2 like nucleolar associated transcriptional repressor	NOC2L
22059	TNPO2	0.888	1.775	1.247	0.096	0.011	0.381	transportin 2	TNPO2
14938	NUP85	0.986	1.775	1.148	0.946	0.027	0.460	nucleoporin 85	NUP85
1413	ATP5I	0.969	1.773	1.799	0.389	0.001	0.180	ATP synthase, H+ transporting, mitochondrial Fo complex subunit E	ATP5I
2344	C4orf48	0.791	1.770	1.760	0.274	0.009	0.372	chromosome 4 open reading frame 48	C4orf48
13052	MAP4K4	0.481	1.769	1.167	0.060	0.241	0.662	mitogen-activated protein kinase kinase kinase kinase 4	MAP4K4
22956	USMG5	1.205	1.769	1.526	0.316	0.070	0.033	up-regulated during skeletal muscle growth 5 homolog (mouse)	USMG5
18404	RPUSD1	0.870	1.769	1.561	0.213	0.007	0.288	RNA pseudouridylate synthase domain containing 1	RPUSD1
14032	MYEOV2	0.737	1.769	1.425	0.159	0.039	0.283	.	MYEOV2
959	APOA1BP	0.859	1.768	1.334	0.598	0.033	0.412	.	APOA1BP
7091	GATA2	0.959	1.767	1.226	0.911	0.211	0.665	GATA binding protein 2	GATA2
6009	FAM122B	1.110	1.766	1.071	0.078	0.044	0.226	family with sequence similarity 122B	FAM122B
1317	ATAD3B	1.098	1.765	1.623	0.780	0.041	0.382	ATPase family, AAA domain containing 3B	ATAD3B
22490	TSTA3	0.828	1.765	1.705	0.476	0.034	0.325	tissue specific transplantation antigen P35B	TSTA3
4525	CYR61	1.127	1.764	1.133	0.825	0.298	0.344	cysteine rich angiogenic inducer 61	CYR61
14011	MYBL1	0.588	1.764	0.723	0.283	0.043	0.597	MYB proto-oncogene like 1	MYBL1
18343	RPS10	0.546	1.763	1.275	0.049	0.008	0.383	ribosomal protein S10	RPS10
19924	SNORA61	9.571	1.763	1.000	0.422	0.725	0.943	small nucleolar RNA, H/ACA box 61	SNORA61
22778	UBL7-AS1	0.898	1.762	1.001	0.380	0.149	0.992	UBL7 antisense RNA 1 (head to head)	UBL7-AS1
13779	MRPS12	0.984	1.762	1.820	0.883	0.126	0.298	mitochondrial ribosomal protein S12	MRPS12
19026	SFXN2	0.963	1.762	1.205	0.880	0.051	0.470	sideroflexin 2	SFXN2
9496	KIF7	0.843	1.760	1.697	0.750	0.195	0.498	kinesin family member 7	KIF7
19096	SH3RF3-AS1	0.609	1.760	1.362	0.085	0.054	0.282	SH3RF3 antisense RNA 1	SH3RF3-AS1
17269	PTGES	42.277	1.758	9.200	0.163	0.255	0.393	prostaglandin E synthase	PTGES
3022	CCT8	0.927	1.758	1.295	0.614	0.040	0.381	chaperonin containing TCP1 subunit 8	CCT8
24116	ZNF580	0.640	1.757	1.617	0.126	0.080	0.199	zinc finger protein 580	ZNF580
22543	TTF2	0.847	1.756	1.010	0.293	0.003	0.966	transcription termination factor, RNA polymerase II	TTF2
3015	CCT4	0.793	1.755	1.283	0.004	0.005	0.059	chaperonin containing TCP1 subunit 4	CCT4
14966	NXN	0.715	1.755	1.534	0.167	0.123	0.019	nucleoredoxin	NXN
14759	NR2C2AP	0.844	1.755	1.262	0.034	0.023	0.194	nuclear receptor 2C2 associated protein	NR2C2AP
8093	HHIP	0.869	1.753	0.894	0.492	0.118	0.606	hedgehog interacting protein	HHIP
17188	PSMB5	1.027	1.753	1.529	0.854	0.007	0.280	proteasome subunit beta 5	PSMB5
8136	HIST1H1C	3.598	1.753	2.234	0.113	0.404	0.100	histone cluster 1, H1c	HIST1H1C
7030	GALE	1.369	1.752	1.431	0.548	0.285	0.506	UDP-galactose-4-epimerase	GALE
18885	SEPHS1	0.773	1.752	1.060	0.225	0.033	0.557	selenophosphate synthetase 1	SEPHS1
22105	TOPBP1	0.874	1.750	0.886	0.538	0.031	0.673	topoisomerase (DNA) II binding protein 1	TOPBP1

23032	USP5	0.934	1.750	1.395	0.425	0.035	0.050	ubiquitin specific peptidase 5	USP5
8335	HOMER3	0.604	1.748	1.136	0.171	0.018	0.619	homer scaffolding protein 3	HOMER3
7000	GADD45GIP1	0.861	1.748	1.673	0.379	0.077	0.292	GADD45G interacting protein 1	GADD45GIP1
1981	C11orf80	0.395	1.748	0.820	0.003	0.062	0.092	chromosome 11 open reading frame 80	C11orf80
8109	HIF1A	2.100	1.748	2.317	0.321	0.260	0.119	hypoxia inducible factor 1 alpha subunit	HIF1A
19916	SNORA57	0.311	1.748	1.015	0.544	0.437	0.990	small nucleolar RNA, H/ACA box 57	SNORA57
6168	FAM216A	1.020	1.748	1.350	0.914	0.247	0.351	family with sequence similarity 216 member A	FAM216A
23296	WDR4	0.955	1.748	1.491	0.820	0.077	0.251	WD repeat domain 4	WDR4
5606	ELP6	0.864	1.748	1.284	0.213	0.004	0.438	elongator acetyltransferase complex subunit 6	ELP6
9185	KAZALD1	0.627	1.748	1.120	0.478	0.076	0.830	Kazal type serine peptidase inhibitor domain 1	KAZALD1
17404	PYCRL	1.077	1.747	1.876	0.793	0.106	0.243	pyrroline-5-carboxylate reductase-like	PYCRL
7761	GSTM1	0.535	1.747	1.150	0.638	0.658	0.919	glutathione S-transferase mu 1	GSTM1
3717	CLIC3	0.685	1.746	0.865	0.679	0.562	0.838	chloride intracellular channel 3	CLIC3
7702	GRK6	1.281	1.746	1.260	0.106	0.004	0.385	G protein-coupled receptor kinase 6	GRK6
2882	CCDC61	1.112	1.746	1.702	0.736	0.045	0.306	coiled-coil domain containing 61	CCDC61
2767	CCBL1	0.813	1.746	1.054	0.322	0.023	0.799	.	CCBL1
17362	PUS1	1.061	1.745	1.606	0.586	0.057	0.056	pseudouridylate synthase 1	PUS1
23064	UXT	0.804	1.745	1.260	0.472	0.005	0.580	ubiquitously expressed prefoldin like chaperone	UXT
6498	FCRLB	0.947	1.744	1.262	0.673	0.018	0.207	Fc receptor like B	FCRLB
17554	RAD54B	0.871	1.743	1.012	0.383	0.006	0.965	RAD54 homolog B (S. cerevisiae)	RAD54B
17189	PSMB6	1.222	1.743	1.976	0.250	0.021	0.067	proteasome subunit beta 6	PSMB6
8884	INHBA	8.268	1.743	2.364	0.020	0.068	0.327	inhibin beta A	INHBA
4932	DKC1	1.071	1.743	1.334	0.585	0.005	0.309	dyskerin pseudouridine synthase 1	DKC1
20422	SPATA33	0.830	1.742	1.009	0.470	0.103	0.965	spermatogenesis associated 33	SPATA33
2738	CBR3	1.365	1.741	1.319	0.564	0.181	0.454	carbonyl reductase 3	CBR3
19120	SHISA3	0.633	1.741	1.064	0.488	0.150	0.894	shisa family member 3	SHISA3
18380	RPS5	0.466	1.741	1.139	0.144	0.034	0.727	ribosomal protein S5	RPS5
23500	YBX1	0.704	1.740	1.221	0.221	0.019	0.457	Y-box binding protein 1	YBX1
14338	NDUFAB1	1.156	1.740	1.495	0.374	0.157	0.015	NADH:ubiquinone oxidoreductase subunit AB1	NDUFAB1
8714	IGFBP3	0.748	1.740	2.009	0.618	0.551	0.295	insulin-like growth factor binding protein 3	IGFBP3
1576	BAIAP2	0.664	1.739	1.297	0.419	0.060	0.611	BAI1 associated protein 2	BAIAP2
3136	CDC37	0.786	1.739	1.471	0.182	0.018	0.301	cell division cycle 37	CDC37
14651	NOP58	1.115	1.738	1.449	0.370	0.024	0.164	NOP58 ribonucleoprotein	NOP58
12	AAAS	0.868	1.738	1.309	0.518	0.002	0.367	aladin WD repeat nucleoporin	AAAS
7810	GTF3C6	1.070	1.738	1.535	0.389	0.015	0.024	general transcription factor IIIC subunit 6	GTF3C6
18315	RPL4	0.458	1.737	1.102	0.103	0.085	0.790	ribosomal protein L4	RPL4
1353	ATIC	1.022	1.737	1.455	0.786	0.004	0.079	5-aminoimidazole-4-carboxamide ribonucleotide formyltransferase/I	ATIC
4687	DDX11	1.104	1.736	1.387	0.700	0.100	0.362	DEAD/H-box helicase 11	DDX11
2214	C1orf35	0.624	1.736	1.244	0.193	0.071	0.559	chromosome 1 open reading frame 35	C1orf35
13468	MINOS1	0.995	1.736	1.529	0.967	0.018	0.229	mitochondrial inner membrane organizing system 1	MINOS1
10100	LIN7B	1.102	1.736	1.306	0.703	0.041	0.403	lin-7 homolog B, crumbs cell polarity complex component	LIN7B
18274	RPL21P28	0.511	1.735	1.024	0.122	0.055	0.935	ribosomal protein L21 pseudogene 28	RPL21P28
1148	ARL16	0.654	1.734	1.178	0.012	0.171	0.178	ADP ribosylation factor like GTPase 16	ARL16
2874	CCDC51	0.575	1.734	1.104	0.031	0.049	0.751	coiled-coil domain containing 51	CCDC51
6523	FES	0.530	1.733	1.447	0.006	0.002	0.262	FES proto-oncogene, tyrosine kinase	FES
18610	SARNP	0.889	1.733	1.650	0.203	0.031	0.286	SAP domain containing ribonucleoprotein	SARNP
21282	TCHP	0.908	1.733	1.390	0.390	0.005	0.455	trichoplein keratin filament binding	TCHP
6345	FANCB	0.952	1.731	1.075	0.193	0.085	0.044	Fanconi anemia complementation group B	FANCB
24129	ZNF593	0.931	1.731	1.995	0.785	0.053	0.362	zinc finger protein 593	ZNF593
8413	HPRT1	1.152	1.731	1.420	0.208	0.038	0.063	hypoxanthine phosphoribosyltransferase 1	HPRT1
3013	CCT2	1.064	1.731	1.257	0.536	0.023	0.114	chaperonin containing TCP1 subunit 2	CCT2
20652	SSNA1	1.069	1.731	1.526	0.563	0.041	0.086	Sjogren syndrome nuclear autoantigen 1	SSNA1
22697	UAP1	1.603	1.730	1.752	0.045	0.109	0.133	UDP-N-acetylglucosamine pyrophosphorylase 1	UAP1
13810	MRTO4	1.031	1.728	1.506	0.857	0.054	0.395	MRT4 homolog, ribosome maturation factor	MRTO4
13756	MRPL4	1.100	1.728	1.769	0.500	0.038	0.091	mitochondrial ribosomal protein L4	MRPL4
14649	NOP2	1.156	1.728	1.525	0.114	0.153	0.013	NOP2 nucleolar protein	NOP2
13084	MAPRE1	1.073	1.728	1.162	0.342	0.038	0.255	microtubule associated protein RP/EB family member 1	MAPRE1
6950	G2E3	1.081	1.728	0.955	0.407	0.084	0.583	G2/M-phase specific E3 ubiquitin protein ligase	G2E3
16003	PDRG1	0.906	1.728	1.376	0.153	0.195	0.028	p53 and DNA damage regulated 1	PDRG1
8833	IL37	1.000	1.726	1.040	0.714	0.034	0.424	interleukin 37	IL37
19700	SMC1A	1.131	1.726	1.099	0.600	0.042	0.570	structural maintenance of chromosomes 1A	SMC1A
9401	KIAA0586	0.893	1.726	1.186	0.692	0.050	0.589	KIAA0586	KIAA0586
5371	EBNA1BP2	1.187	1.725	1.340	0.043	0.096	0.014	EBNA1 binding protein 2	EBNA1BP2
23477	XRCC1	0.963	1.725	1.117	0.578	0.000	0.211	X-ray repair complementing defective repair in Chinese hamster cells	XRCC1
18373	RPS29	0.563	1.725	1.205	0.074	0.085	0.385	ribosomal protein S29	RPS29
8859	IMP4	1.038	1.724	1.886	0.805	0.035	0.222	IMP4 homolog, U3 small nucleolar ribonucleoprotein	IMP4
17254	PTDSS1	0.609	1.723	0.947	0.121	0.046	0.799	phosphatidylserine synthase 1	PTDSS1
23116	VEPH1	0.774	1.723	1.122	0.369	0.357	0.748	ventricular zone expressed PH domain containing 1	VEPH1
5178	DPP3	1.052	1.723	1.427	0.842	0.121	0.215	dipeptidyl peptidase 3	DPP3
16824	PPP2R4	0.747	1.722	1.588	0.107	0.017	0.201	protein phosphatase 2A regulatory subunit 4	PPP2R4
18367	RPS26	0.766	1.722	1.451	0.609	0.109	0.499	ribosomal protein S26	RPS26
17693	RBL1	0.949	1.721	0.916	0.641	0.016	0.767	retinoblastoma-like 1	RBL1
22219	TRAPPC1	0.695	1.721	1.657	0.005	0.030	0.029	trafficking protein particle complex 1	TRAPPC1
18256	RPL12	0.433	1.720	1.038	0.005	0.028	0.674	ribosomal protein L12	RPL12
9233	KCNG1	1.122	1.720	1.268	0.686	0.069	0.423	potassium voltage-gated channel modifier subfamily G member 1	KCNG1
18233	RPA2	0.909	1.720	0.816	0.583	0.111	0.162	replication protein A2	RPA2
19055	SGTA	0.955	1.719	1.559	0.774	0.025	0.279	small glutamine rich tetratricopeptide repeat containing alpha	SGTA
17349	PTS	1.684	1.718	1.075	0.026	0.085	0.496	6-pyruvoyltetrahydropterin synthase	PTS
12609	LOXL4	0.991	1.718	0.746	0.982	0.185	0.389	lysyl oxidase like 4	LOXL4
16217	PIDD1	0.592	1.718	1.325	0.207	0.018	0.365	p53-induced death domain protein 1	PIDD1
641	ALKBH2	0.719	1.717	1.142	0.261	0.063	0.534	alkB homolog 2, alpha-ketoglutarate-dependent dioxygenase	ALKBH2
21062	TAF10	0.920	1.717	1.870	0.454	0.016	0.119	TATA-box binding protein associated factor 10	TAF10
18292	RPL28	0.701	1.717	1.267	0.362	0.148	0.604	ribosomal protein L28	RPL28
10429	LINC00707	1.331	1.717	1.501	0.267	0.114	0.148	long intergenic non-protein coding RNA 707	LINC00707
1003	APRT	0.790	1.716	1.666	0.564	0.038	0.274	adenine phosphoribosyltransferase	APRT
20932	SUSD2	0.451	1.716	1.478	0.367	0.264	0.582	sushi domain containing 2	SUSD2
7931	HAT1	1.311	1.716	1.093	0.338	0.033	0.755	histone acetyltransferase 1	HAT1
883	ANXA6	0.660	1.716	1.451	0.081	0.047	0.083	annexin A6	ANXA6
17279	PTGR1	0.864	1.716	1.442	0.350	0.071	0.235	prostaglandin reductase 1	PTGR1
4792	DEK	1.071	1.715	1.005	0.626	0.087	0.968	DEK proto-oncogene	DEK
1556	BABAM1	0.910	1.715	1.709	0.581	0.014	0.207	BRISC and BRCA1 A complex member 1	BABAM1

15922	PDAP1	0.939	1.715	1.471	0.010	0.006	0.233	PDGFA associated protein 1	PDAP1
17332	PTPRJ	1.679	1.714	1.614	0.105	0.143	0.007	protein tyrosine phosphatase, receptor type J	PTPRJ
14595	NME4	0.704	1.714	1.543	0.133	0.119	0.275	NME/NM23 nucleoside diphosphate kinase 4	NME4
20734	STAG3L5P	0.626	1.714	1.038	0.280	0.143	0.902	stromal antigen 3-like 5 pseudogene	STAG3L5P
14876	NUBP2	0.793	1.713	1.557	0.464	0.037	0.351	nucleotide binding protein 2	NUBP2
7882	H2AFY	0.896	1.713	1.155	0.561	0.006	0.406	H2A histone family member Y	H2AFY
17532	RAC2	0.968	1.713	1.382	0.903	0.339	0.200	ras-related C3 botulinum toxin substrate 2 (rho family, small GTP bind	RAC2
21477	THBD	0.950	1.712	1.418	0.943	0.600	0.602	thrombomodulin	THBD
21201	TBCD	0.648	1.712	1.143	0.006	0.060	0.424	tubulin folding cofactor D	TBCD
22086	TOMM22	0.923	1.711	1.802	0.385	0.012	0.093	translocase of outer mitochondrial membrane 22	TOMM22
18291	RPL27A	0.538	1.711	1.302	0.052	0.017	0.400	ribosomal protein L27a	RPL27A
5900	EXOSC2	1.073	1.710	1.493	0.642	0.013	0.401	exosome component 2	EXOSC2
18357	RPS18	0.403	1.709	0.925	0.026	0.047	0.514	ribosomal protein S18	RPS18
21289	TCOF1	1.081	1.708	1.309	0.404	0.008	0.427	treacle ribosome biogenesis factor 1	TCOF1
728	ANAPC7	0.955	1.708	1.366	0.689	0.032	0.147	anaphase promoting complex subunit 7	ANAPC7
13739	MRPL21	0.953	1.708	1.386	0.867	0.035	0.405	mitochondrial ribosomal protein L21	MRPL21
18396	RPS8	0.430	1.707	1.088	0.029	0.046	0.564	ribosomal protein S8	RPS8
2011	C12orf75	0.295	1.707	0.603	0.003	0.007	0.372	chromosome 12 open reading frame 75	C12orf75
17206	PSMD14	1.394	1.707	1.248	0.139	0.038	0.265	proteasome 26S subunit, non-ATPase 14	PSMD14
2136	C19orf70	0.831	1.706	1.776	0.269	0.051	0.279	chromosome 19 open reading frame 70	C19orf70
14161	NACC1	1.127	1.706	1.519	0.385	0.021	0.006	nucleus accumbens associated 1	NACC1
13766	MRPL47	0.957	1.706	1.226	0.579	0.002	0.378	mitochondrial ribosomal protein L47	MRPL47
1920	BYSL	1.265	1.705	1.641	0.100	0.067	0.105	bystin like	BYSL
6093	FAM173B	0.797	1.705	0.985	0.111	0.013	0.802	family with sequence similarity 173 member B	FAM173B
7903	HADHA	0.547	1.705	1.167	0.003	0.078	0.531	hydroxyacyl-CoA dehydrogenase/3-ketoacyl-CoA thiolase/enoyl-CoA	HADHA
14329	NDUFA3	1.237	1.703	1.944	0.199	0.019	0.254	NADH:ubiquinone oxidoreductase subunit A3	NDUFA3
17235	PSTPIP1	0.748	1.703	0.870	0.019	0.312	0.038	proline-serine-threonine phosphatase interacting protein 1	PSTPIP1
4374	CUTA	0.824	1.702	1.337	0.163	0.010	0.252	cutA divalent cation tolerance homolog (E. coli)	CUTA
17538	RAD18	1.023	1.702	0.996	0.857	0.009	0.981	RAD18, E3 ubiquitin protein ligase	RAD18
19119	SHISA2	1.372	1.700	1.188	0.040	0.006	0.012	shisa family member 2	SHISA2
5532	EIF3I	0.877	1.700	1.572	0.340	0.009	0.133	eukaryotic translation initiation factor 3 subunit I	EIF3I
3138	CDC37L1-AS1	0.686	1.700	1.039	0.261	0.083	0.958	CDC37L1 antisense RNA 1 (head to head)	CDC37L1-AS1
16279	PINX1	0.817	1.700	1.244	0.325	0.145	0.338	PIN2/TERF1 interacting, telomerase inhibitor 1	PINX1
21796	TMEM204	0.451	1.700	1.338	0.170	0.060	0.449	transmembrane protein 204	TMEM204
21211	TBL3	0.934	1.700	1.689	0.804	0.001	0.353	transducin beta like 3	TBL3
6628	FKBP5	0.411	1.699	0.902	0.214	0.097	0.823	FK506 binding protein 5	FKBP5
14855	NTM	0.389	1.697	0.778	0.304	0.201	0.705	neurotrimin	NTM
7031	GALK1	0.817	1.697	1.869	0.443	0.063	0.031	galactokinase 1	GALK1
9569	KLHL13	2.418	1.696	1.713	0.410	0.358	0.289	kelch like family member 13	KLHL13
18406	RPUSD3	0.900	1.696	1.548	0.348	0.063	0.024	RNA pseudouridylate synthase domain containing 3	RPUSD3
3613	CISD3	0.994	1.695	1.747	0.963	0.043	0.073	CDGSH iron sulfur domain 3	CISD3
13738	MRPL20	0.993	1.695	1.742	0.906	0.004	0.175	mitochondrial ribosomal protein L20	MRPL20
3494	CHEK1	1.049	1.695	1.276	0.898	0.092	0.414	checkpoint kinase 1	CHEK1
5310	DUT	0.902	1.695	1.322	0.399	0.013	0.272	deoxyuridine triphosphatase	DUT
16812	PPP2CA	1.150	1.694	1.435	0.234	0.006	0.035	protein phosphatase 2 catalytic subunit alpha	PPP2CA
22681	TYMSOS	0.486	1.693	1.019	0.032	0.053	0.866	TYMS opposite strand	TYMSOS
21198	TBCB	0.763	1.693	1.385	0.045	0.010	0.018	tubulin folding cofactor B	TBCB
7529	GPD1L	0.539	1.693	1.055	0.092	0.034	0.786	glycerol-3-phosphate dehydrogenase 1-like	GPD1L
32	AARSD1	0.717	1.692	1.386	0.103	0.108	0.407	alanyl-tRNA synthetase domain containing 1	AARSD1
14328	NDUFA2	1.030	1.692	1.715	0.812	0.002	0.248	NADH:ubiquinone oxidoreductase subunit A2	NDUFA2
1993	C12orf10	0.636	1.692	1.266	0.065	0.039	0.392	chromosome 12 open reading frame 10	C12orf10
8340	HOPX	0.495	1.692	1.097	0.564	0.564	0.921	HOP homeobox	HOPX
14230	NBL1	0.441	1.692	1.234	0.159	0.206	0.599	neuroblastoma 1, DAN family BMP antagonist	NBL1
22692	U2AF1	1.016	1.691	1.516	0.886	0.013	0.191	U2 small nuclear RNA auxiliary factor 1	U2AF1
5103	DNLZ	0.856	1.691	1.615	0.432	0.187	0.087	DNL-type zinc finger	DNLZ
23970	ZNF385D	0.905	1.691	1.782	0.820	0.138	0.322	zinc finger protein 385D	ZNF385D
18320	RPL7A	0.408	1.691	0.949	0.033	0.032	0.678	ribosomal protein L7a	RPL7A
3205	CDK16	1.018	1.690	1.552	0.449	0.001	0.000	cyclin-dependent kinase 16	CDK16
23337	WDR90	1.058	1.690	0.961	0.783	0.047	0.855	WD repeat domain 90	WDR90
21029	SYTL3	1.136	1.690	0.996	0.291	0.283	0.956	synaptotagmin like 3	SYTL3
16609	POLR2J	0.870	1.689	1.354	0.491	0.091	0.075	polymerase (RNA) II subunit J	POLR2J
21066	TAF15	1.051	1.688	1.849	0.706	0.095	0.444	TATA-box binding protein associated factor 15	TAF15
1154	ARL3	0.932	1.688	1.210	0.683	0.097	0.375	ADP ribosylation factor like GTPase 3	ARL3
19835	SNHG1	0.794	1.688	1.046	0.438	0.178	0.796	small nucleolar RNA host gene 1	SNHG1
14948	NUTF2	0.711	1.688	1.072	0.329	0.028	0.843	nuclear transport factor 2	NUTF2
21291	TCP1	0.777	1.687	0.990	0.114	0.018	0.925	t-complex 1	TCP1
752	ANKH	0.731	1.687	1.433	0.312	0.385	0.637	ANKH inorganic pyrophosphate transport regulator	ANKH
16303	PITPNM3	0.762	1.686	1.526	0.190	0.125	0.050	PITPNM family member 3	PITPNM3
1403	ATP5B	1.285	1.685	1.669	0.219	0.027	0.110	ATP synthase, H+ transporting, mitochondrial F1 complex, beta polyp	ATP5B
16130	PGP	0.999	1.685	0.960	0.997	0.113	0.591	phosphoglycolate phosphatase	PGP
4075	CPT1C	0.709	1.685	1.591	0.304	0.166	0.030	carnitine palmitoyltransferase 1C	CPT1C
19693	SMARCB1	0.843	1.685	1.460	0.082	0.007	0.021	SWI/SNF related, matrix associated, actin dependent regulator of chr	SMARCB1
1410	ATP5G2	0.725	1.685	1.440	0.014	0.000	0.082	ATP synthase, H+ transporting, mitochondrial Fo complex subunit C2	ATP5G2
4225	CSPG4	0.410	1.684	1.356	0.002	0.030	0.600	chondroitin sulfate proteoglycan 4	CSPG4
19849	SNHG3	1.012	1.684	1.436	0.976	0.132	0.360	small nucleolar RNA host gene 3	SNHG3
17224	PSMG3	0.835	1.684	1.503	0.309	0.042	0.111	proteasome assembly chaperone 3	PSMG3
20287	SOCS2	0.685	1.684	1.661	0.370	0.255	0.225	suppressor of cytokine signaling 2	SOCS2
23079	VARS	1.225	1.683	2.032	0.124	0.001	0.071	valyl-tRNA synthetase	VARS
17723	RBM42	0.915	1.683	1.388	0.463	0.029	0.050	RNA binding motif protein 42	RBM42
19851	SNHG5	0.434	1.683	0.731	0.362	0.656	0.681	small nucleolar RNA host gene 5	SNHG5
3967	COPS3	0.774	1.683	1.177	0.048	0.017	0.154	COP9 signalosome subunit 3	COPS3
4666	DDB2	0.301	1.682	0.830	0.000	0.004	0.336	damage specific DNA binding protein 2	DDB2
18351	RPS15	0.659	1.681	1.373	0.183	0.020	0.457	ribosomal protein S15	RPS15
4224	CSNK2B	0.817	1.681	1.489	0.307	0.006	0.345	casein kinase 2 beta	CSNK2B
2640	CAPNS1	0.621	1.681	1.553	0.023	0.000	0.035	calpain small subunit 1	CAPNS1
13464	MILR1	1.969	1.680	2.682	0.324	0.222	0.266	mast cell immunoglobulin like receptor 1	MILR1
7586	GPR183	1.588	1.679	1.126	0.102	0.096	0.392	G protein-coupled receptor 183	GPR183
5536	EIF3K	0.673	1.679	1.379	0.219	0.031	0.477	eukaryotic translation initiation factor 3 subunit K	EIF3K
23281	WDR18	0.971	1.679	1.886	0.738	0.110	0.136	WD repeat domain 18	WDR18
7759	GSTCD	1.068	1.679	0.902	0.303	0.066	0.557	glutathione S-transferase C-terminal domain containing	GSTCD
5537	EIF3L	0.371	1.678	0.924	0.039	0.054	0.714	eukaryotic translation initiation factor 3 subunit L	EIF3L

23095	VCAN	0.819	1.678	1.652	0.733	0.353	0.146	versican	VCAN
8495	HSP90AA1	0.755	1.677	0.955	0.104	0.058	0.833	heat shock protein 90kDa alpha family class A member 1	HSP90AA1
10593	LINC01063	0.846	1.676	1.355	0.424	0.000	0.574	long intergenic non-protein coding RNA 1063	LINC01063
23191	VPS9D1-AS1	0.904	1.676	1.676	0.711	0.204	0.153	VPS9D1 antisense RNA 1	VPS9D1-AS1
10946	LINC01564	0.542	1.676	1.166	0.199	0.141	0.549	long intergenic non-protein coding RNA 1564	LINC01564
18311	RPL38	0.647	1.675	1.226	0.029	0.004	0.415	ribosomal protein L38	RPL38
15036	OLA1	0.781	1.675	1.061	0.118	0.002	0.808	Obg-like ATPase 1	OLA1
20661	SSSCA1	1.005	1.674	1.633	0.984	0.022	0.300	Sjogren syndrome/scleroderma autoantigen 1	SSSCA1
5624	EMG1	1.223	1.674	1.798	0.466	0.012	0.319	EMG1 N1-specific pseudouridine methyltransferase	EMG1
5346	DZIP1	0.921	1.674	1.130	0.520	0.098	0.490	DAZ interacting zinc finger protein 1	DZIP1
2148	C1QBP	1.231	1.673	1.742	0.081	0.057	0.109	complement component 1, q subcomponent binding protein	C1QBP
8580	HYPK	1.432	1.673	1.539	0.096	0.057	0.375	huntingtin interacting protein K	HYPK
2027	C14orf2	0.863	1.672	1.548	0.162	0.000	0.150	chromosome 14 open reading frame 2	C14orf2
6506	FDXR	0.330	1.672	1.316	0.094	0.111	0.302	ferredoxin reductase	FDXR
18269	RPL18	0.464	1.672	1.120	0.114	0.014	0.769	ribosomal protein L18	RPL18
18778	SDF2L1	1.140	1.672	1.788	0.502	0.041	0.414	stromal cell derived factor 2 like 1	SDF2L1
8875	INE2	0.570	1.672	1.246	0.114	0.203	0.287	inactivation escape 2 (non-protein coding)	INE2
3605	CINP	0.813	1.672	1.645	0.269	0.040	0.287	cyclin-dependent kinase 2 interacting protein	CINP
12754	LRRC8C	0.624	1.672	0.928	0.441	0.053	0.876	leucine-rich repeat containing 8 family member C	LRRC8C
3870	COA4	1.276	1.670	1.531	0.140	0.098	0.139	cytochrome c oxidase assembly factor 4 homolog	COA4
6800	FOXN3-AS1	0.759	1.669	1.077	0.243	0.096	0.706	FOXN3 antisense RNA 1	FOXN3-AS1
13219	MDC1	0.972	1.669	1.065	0.699	0.073	0.661	mediator of DNA damage checkpoint 1	MDC1
2801	CCDC14	0.865	1.668	0.961	0.064	0.108	0.799	coiled-coil domain containing 14	CCDC14
12983	MALSU1	0.900	1.668	1.136	0.338	0.047	0.036	mitochondrial assembly of ribosomal large subunit 1	MALSU1
23745	ZGRF1	1.038	1.668	1.123	0.651	0.099	0.278	zinc finger GRF-type containing 1	ZGRF1
20436	SPATC1L	0.578	1.668	1.325	0.150	0.076	0.429	spermatogenesis and centriole associated 1-like	SPATC1L
12795	LSM7	0.864	1.668	1.584	0.510	0.083	0.254	LSM7 homolog, U6 small nuclear RNA and mRNA degradation associa	LSM7
14610	NMT2	0.814	1.668	1.306	0.549	0.285	0.482	N-myristoyltransferase 2	NMT2
3480	CHCHD5	0.783	1.666	1.541	0.180	0.006	0.335	coiled-coil-helix-coiled-coil-helix domain containing 5	CHCHD5
22948	USB1	1.334	1.666	1.399	0.062	0.066	0.032	U6 snRNA biogenesis phosphodiesterase 1	USB1
13840	MSH5-SAPCD1	0.828	1.666	1.036	0.307	0.036	0.741	MSH5-SAPCD1 readthrough (NMD candidate)	MSH5-SAPCD1
13836	MSH2	0.866	1.666	0.949	0.460	0.038	0.769	mutS homolog 2	MSH2
7793	GTF2H4	0.888	1.665	1.532	0.496	0.002	0.102	general transcription factor IIH subunit 4	GTF2H4
1923	BZW1	0.915	1.665	1.394	0.736	0.001	0.215	basic leucine zipper and W2 domains 1	BZW1
15491	ORMDL2	1.200	1.665	1.512	0.642	0.162	0.058	ORMDL sphingolipid biosynthesis regulator 2	ORMDL2
9396	KIAA0391	1.080	1.665	1.255	0.320	0.131	0.472	KIAA0391	KIAA0391
18209	ROGDI	0.562	1.664	1.134	0.030	0.070	0.619	rogdi homolog	ROGDI
646	ALKBH6	1.246	1.664	1.703	0.468	0.200	0.071	alkB homolog 6	ALKBH6
19840	SNHG16	1.093	1.664	1.483	0.745	0.084	0.279	small nucleolar RNA host gene 16	SNHG16
3986	CORO1A	0.844	1.664	1.358	0.292	0.023	0.058	coronin 1A	CORO1A
18290	RPL27	0.580	1.664	1.129	0.015	0.074	0.223	ribosomal protein L27	RPL27
5756	ERAL1	0.944	1.663	1.391	0.750	0.041	0.116	Era like 12S mitochondrial rRNA chaperone 1	ERAL1
21683	TMEM107	0.941	1.661	1.381	0.838	0.112	0.109	transmembrane protein 107	TMEM107
15789	PCBD1	0.740	1.661	1.059	0.168	0.125	0.902	pterin-4 alpha-carbinolamine dehydratase 1	PCBD1
18310	RPL37A	0.507	1.661	1.041	0.058	0.027	0.887	ribosomal protein L37a	RPL37A
8170	HIST1H3C	0.706	1.660	0.835	0.003	0.021	0.012	histone cluster 1, H3c	HIST1H3C
22947	UROS	0.649	1.660	1.308	0.003	0.038	0.012	uroporphyrinogen III synthase	UROS
970	APOBEC3C	0.499	1.660	1.347	0.199	0.235	0.357	apolipoprotein B mRNA editing enzyme catalytic subunit 3C	APOBEC3C
15052	OMD	0.142	1.660	0.640	0.001	0.145	0.059	osteomodulin	OMD
7632	GPRIN1	0.922	1.659	1.033	0.699	0.075	0.727	G protein regulated inducer of neurite outgrowth 1	GPRIN1
13350	METTL5	0.906	1.659	1.300	0.667	0.087	0.021	methyltransferase like 5	METTL5
16577	POLD2	0.971	1.659	1.374	0.835	0.033	0.151	polymerase (DNA) delta 2, accessory subunit	POLD2
16960	PRKAR1B	0.534	1.659	1.240	0.028	0.145	0.035	protein kinase cAMP-dependent type I regulatory subunit beta	PRKAR1B
23635	ZC3HC1	0.850	1.659	1.326	0.188	0.004	0.113	zinc finger C3HC-type containing 1	ZC3HC1
18381	RPS6	0.424	1.659	0.958	0.039	0.047	0.770	ribosomal protein S6	RPS6
13781	MRPS15	0.941	1.659	1.409	0.352	0.012	0.019	mitochondrial ribosomal protein S15	MRPS15
13715	MRM1	1.020	1.658	1.310	0.788	0.124	0.314	mitochondrial rRNA methyltransferase 1	MRM1
9563	KLHDC8B	0.636	1.658	1.263	0.244	0.085	0.467	kelch domain containing 8B	KLHDC8B
18436	RRS1	1.185	1.658	1.319	0.376	0.127	0.225	ribosome biogenesis regulator homolog	RRS1
16604	POLR2E	0.861	1.658	1.382	0.093	0.013	0.036	polymerase (RNA) II subunit E	POLR2E
6035	FAM136A	0.757	1.658	1.109	0.023	0.005	0.380	family with sequence similarity 136 member A	FAM136A
2828	CCDC167	1.016	1.658	1.390	0.883	0.015	0.297	coiled-coil domain containing 167	CCDC167
2604	CAMK2N2	0.846	1.658	0.949	0.416	0.165	0.877	calcium/calmodulin dependent protein kinase II inhibitor 2	CAMK2N2
14281	NCL	0.899	1.658	1.489	0.727	0.111	0.544	nucleolin	NCL
4446	CYC1	1.363	1.658	1.461	0.113	0.233	0.007	cytochrome c1	CYC1
2662	CARHSP1	0.630	1.657	1.247	0.273	0.385	0.674	calcium regulated heat stable protein 1	CARHSP1
16717	PPCDC	0.739	1.657	1.175	0.146	0.287	0.203	phosphopantothenoylcysteine decarboxylase	PPCDC
21759	TMEM177	0.799	1.657	1.233	0.285	0.241	0.208	transmembrane protein 177	TMEM177
909	AP2M1	0.829	1.657	1.459	0.164	0.032	0.134	adaptor related protein complex 2 mu 1 subunit	AP2M1
3017	CCT6A	0.894	1.656	1.175	0.369	0.020	0.192	chaperonin containing TCP1 subunit 6A	CCT6A
3407	CETN3	1.233	1.656	1.354	0.332	0.071	0.081	centrin, EF-hand protein, 3	CETN3
950	APITD1-CORT	0.861	1.656	1.296	0.596	0.075	0.305	APITD1-CORT readthrough	APITD1-CORT
1419	ATP5O	0.848	1.656	1.259	0.002	0.067	0.051	ATP synthase, H+ transporting, mitochondrial F1 complex, O subunit	ATP5O
5844	ETFA	0.907	1.654	1.322	0.416	0.011	0.085	electron transfer flavoprotein alpha subunit	ETFA
3322	CEND1	2.193	1.654	1.899	0.070	0.509	0.171	cell cycle exit and neuronal differentiation 1	CEND1
2512	CABLES1	1.400	1.654	1.221	0.497	0.187	0.746	Cdk5 and Abl enzyme substrate 1	CABLES1
11643	LOC10192778	1.287	1.654	1.833	0.447	0.444	0.230	uncharacterized LOC101927780	LOC101927780
14717	NPM1	0.595	1.654	1.048	0.036	0.007	0.730	nucleophosmin (nucleolar phosphoprotein B23, numatrin)	NPM1
9961	LCORL	1.091	1.654	1.077	0.771	0.005	0.849	ligand dependent nuclear receptor corepressor like	LCORL
17053	PRPS1	0.803	1.653	1.073	0.398	0.012	0.775	phosphoribosyl pyrophosphate synthetase 1	PRPS1
13544	MLF2	1.111	1.653	1.513	0.476	0.004	0.246	myeloid leukemia factor 2	MLF2
23302	WDR46	1.048	1.653	1.733	0.805	0.043	0.396	WD repeat domain 46	WDR46
2129	C19orf53	0.813	1.653	1.345	0.114	0.105	0.241	chromosome 19 open reading frame 53	C19orf53
3477	CHCHD2	0.868	1.653	1.344	0.328	0.033	0.085	coiled-coil-helix-coiled-coil-helix domain containing 2	CHCHD2
7132	GCDH	0.873	1.652	1.640	0.204	0.009	0.242	glutaryl-CoA dehydrogenase	GCDH
22090	TOMM5	1.187	1.652	1.304	0.313	0.130	0.435	translocase of outer mitochondrial membrane 5	TOMM5
16650	POP7	1.112	1.651	1.242	0.565	0.087	0.084	POP7 homolog, ribonuclease P/MRP subunit	POP7
7615	GPR75-ASB3	1.228	1.651	1.854	0.298	0.523	0.420	GPR75-ASB3 readthrough	GPR75-ASB3
14219	NAV2	0.830	1.650	1.221	0.571	0.167	0.415	neuron navigator 2	NAV2
14347	NDUFB1	0.921	1.650	1.396	0.124	0.083	0.468	NADH:ubiquinone oxidoreductase subunit B1	NDUFB1
133	ACAA2	0.756	1.650	1.498	0.259	0.077	0.134	acetyl-CoA acyltransferase 2	ACAA2

23481	XRCC5	1.004	1.649	1.171	0.940	0.008	0.088	X-ray repair complementing defective repair in Chinese hamster cells	XRCC5
14110	MYPN	3.115	1.649	2.432	0.015	0.516	0.055	myopalladin	MYPN
10996	LIPE	0.884	1.648	1.069	0.519	0.043	0.553	lipase E, hormone sensitive type	LIPE
4008	COX5A	1.053	1.648	1.265	0.683	0.120	0.165	cytochrome c oxidase subunit 5A	COX5A
20815	STOML2	1.103	1.646	1.749	0.322	0.029	0.082	stomatin like 2	STOML2
12750	LRRC75A-AS1	0.364	1.646	0.963	0.079	0.120	0.900	LRRC75A antisense RNA 1	LRRC75A-AS1
8125	HIP1	0.638	1.646	0.963	0.044	0.291	0.725	huntingtin interacting protein 1	HIP1
8315	HNRNPD	1.039	1.646	1.590	0.894	0.132	0.408	heterogeneous nuclear ribonucleoprotein D	HNRNPD
16513	PMPCA	0.824	1.646	1.704	0.510	0.026	0.395	peptidase, mitochondrial processing alpha subunit	PMPCA
23479	XRCC3	0.878	1.645	1.033	0.309	0.093	0.770	X-ray repair complementing defective repair in Chinese hamster cells	XRCC3
12615	LPAR4	1.562	1.645	1.263	0.010	0.149	0.265	lysophosphatidic acid receptor 4	LPAR4
4578	DAZAP1	1.313	1.645	1.909	0.194	0.003	0.124	DAZ associated protein 1	DAZAP1
4900	DIAPH3-AS1	0.573	1.645	1.253	0.423	0.160	0.739	DIAPH3 antisense RNA 1	DIAPH3-AS1
22703	UBA52	0.589	1.644	1.305	0.038	0.006	0.319	ubiquitin A-52 residue ribosomal protein fusion product 1	UBA52
22172	TPRKB	1.238	1.644	1.005	0.518	0.157	0.990	TP53RK binding protein	TPRKB
18431	RRP36	0.915	1.644	1.275	0.606	0.027	0.225	ribosomal RNA processing 36	RRP36
5515	EIF2B3	0.910	1.643	1.321	0.159	0.014	0.040	eukaryotic translation initiation factor 2B subunit gamma	EIF2B3
18074	RNF126	1.307	1.642	1.360	0.019	0.037	0.015	ring finger protein 126	RNF126
18299	RPL32	0.551	1.642	1.299	0.088	0.016	0.327	ribosomal protein L32	RPL32
22230	TRAPPC5	0.805	1.642	1.531	0.326	0.016	0.364	trafficking protein particle complex 5	TRAPPC5
18354	RPS16	0.531	1.642	1.120	0.091	0.040	0.735	ribosomal protein S16	RPS16
12048	LOC10246708	0.660	1.641	0.906	0.311	0.423	0.754	uncharacterized LOC102467080	LOC102467080
8633	IFI27L1	1.184	1.641	1.180	0.676	0.304	0.431	interferon alpha inducible protein 27 like 1	IFI27L1
5950	FAAP20	0.712	1.641	1.680	0.365	0.033	0.373	Fanconi anemia core complex associated protein 20	FAAP20
7225	GGTLC2	1.104	1.641	1.636	0.754	0.269	0.537	gamma-glutamyltransferase light chain 2	GGTLC2
3340	CENPT	0.808	1.641	1.037	0.226	0.140	0.883	centromere protein T	CENPT
17170	PSIP1	0.714	1.640	0.913	0.011	0.070	0.088	PC4 and SFRS1 interacting protein 1	PSIP1
1284	ASL	1.302	1.640	2.254	0.235	0.234	0.178	argininosuccinate lyase	ASL
5124	DOC2A	0.926	1.640	1.684	0.637	0.026	0.173	double C2 domain alpha	DOC2A
6948	FZR1	0.996	1.639	1.318	0.974	0.038	0.245	fizzy/cell division cycle 20 related 1	FZR1
20473	SPHK1	1.081	1.639	3.161	0.880	0.280	0.409	sphingosine kinase 1	SPHK1
24351	ZNF93	0.949	1.639	0.843	0.816	0.060	0.578	zinc finger protein 93	ZNF93
17741	RBMX	0.851	1.638	1.260	0.051	0.001	0.015	RNA binding motif protein, X-linked	RBMX
20928	SURF2	0.663	1.638	1.223	0.009	0.018	0.500	surfeit 2	SURF2
10756	LINC01296	1.022	1.638	1.347	0.891	0.358	0.423	long intergenic non-protein coding RNA 1296	LINC01296
17180	PSMA7	1.171	1.638	1.669	0.481	0.020	0.304	proteasome subunit alpha 7	PSMA7
8600	ICMT	0.873	1.638	0.936	0.572	0.048	0.856	isoprenylcysteine carboxyl methyltransferase	ICMT
18336	RPP40	0.882	1.638	1.505	0.462	0.198	0.145	ribonuclease P/MRP subunit p40	RPP40
20640	SSBP1	1.035	1.637	1.473	0.689	0.078	0.056	single stranded DNA binding protein 1	SSBP1
11064	LOC10012800	1.024	1.637	1.516	0.522	0.137	0.102	uncharacterized LOC100128006	LOC100128006
2218	C1orf53	1.058	1.636	1.079	0.537	0.115	0.803	chromosome 1 open reading frame 53	C1orf53
1485	AUNIP	0.823	1.636	0.903	0.068	0.090	0.283	aurora kinase A and ninein interacting protein	AUNIP
6504	FDX1L	0.773	1.636	1.344	0.152	0.159	0.109	ferredoxin 1-like	FDX1L
21561	TIMP1	1.646	1.635	2.493	0.146	0.433	0.372	TIMP metallopeptidase inhibitor 1	TIMP1
5863	EVA1C	1.347	1.634	1.639	0.190	0.004	0.469	eva-1 homolog C (C. elegans)	EVA1C
14156	NABP2	0.946	1.634	1.425	0.544	0.119	0.062	nucleic acid binding protein 2	NABP2
3868	COA1	1.011	1.634	1.341	0.951	0.026	0.099	cytochrome c oxidase assembly factor 1 homolog	COA1
14327	NDUFA13	0.964	1.633	1.782	0.900	0.050	0.424	NADH:ubiquinone oxidoreductase subunit A13	NDUFA13
21255	TCEB1	1.195	1.633	1.203	0.300	0.033	0.236	transcription elongation factor B subunit 1	TCEB1
16044	PEMT	1.070	1.633	1.538	0.647	0.150	0.086	phosphatidylethanolamine N-methyltransferase	PEMT
8575	HYI	0.701	1.633	1.047	0.031	0.069	0.697	hydroxypyruvate isomerase (putative)	HYI
8157	HIST1H2BE	1.118	1.633	1.000	0.423	0.377	0.943	histone cluster 1, H2be	HIST1H2BE
21088	TAGLN2	1.591	1.632	2.269	0.020	0.023	0.042	transgelin 2	TAGLN2
13730	MRPL13	1.262	1.632	1.557	0.095	0.005	0.087	mitochondrial ribosomal protein L13	MRPL13
4630	DCK	1.009	1.632	0.902	0.966	0.023	0.795	deoxycytidine kinase	DCK
949	APITD1	1.307	1.632	1.335	0.238	0.083	0.011	apoptosis-inducing, TAF9-like domain 1	APITD1
1525	B3GAT3	1.002	1.632	1.528	0.984	0.217	0.214	beta-1,3-glucuronyltransferase 3	B3GAT3
13667	MPHOSPH9	1.075	1.631	1.179	0.557	0.001	0.590	M-phase phosphoprotein 9	MPHOSPH9
19354	SLC25A3	0.617	1.631	1.379	0.010	0.014	0.262	solute carrier family 25 member 3	SLC25A3
22226	TRAPPC2L	0.597	1.631	1.324	0.149	0.001	0.563	trafficking protein particle complex 2-like	TRAPPC2L
13419	MGC72080	1.238	1.629	2.541	0.186	0.012	0.042	MGC72080 pseudogene	MGC72080
18347	RPS12	0.484	1.629	1.187	0.226	0.123	0.764	ribosomal protein S12	RPS12
13545	MLH1	0.985	1.629	1.058	0.878	0.048	0.573	mutL homolog 1	MLH1
21769	TMEM183B	0.736	1.628	1.373	0.010	0.000	0.015	transmembrane protein 183B	TMEM183B
4720	DDX49	0.725	1.628	1.274	0.169	0.017	0.469	DEAD-box helicase 49	DDX49
22147	TPGS2	0.848	1.627	1.323	0.187	0.152	0.114	tubulin polyglutamylase complex subunit 2	TPGS2
4718	DDX46	0.917	1.627	1.238	0.467	0.193	0.547	DEAD-box helicase 46	DDX46
155	ACBD7	0.899	1.626	1.309	0.416	0.068	0.281	acyl-CoA binding domain containing 7	ACBD7
14313	NDOR1	1.005	1.626	1.163	0.975	0.059	0.429	NADPH dependent diflavin oxidoreductase 1	NDOR1
14560	NLE1	0.634	1.626	1.201	0.058	0.065	0.153	notchless homolog 1 (Drosophila)	NLE1
6765	FOXC2-AS1	0.830	1.626	1.170	0.679	0.114	0.707	FOXC2 antisense RNA 1	FOXC2-AS1
14060	MYL6B	0.756	1.626	1.565	0.203	0.028	0.500	myosin light chain 6B	MYL6B
23454	XKR5	0.940	1.626	1.209	0.479	0.050	0.105	XK related 5	XKR5
16006	PDSS1	0.981	1.625	1.042	0.904	0.252	0.884	prenyl (decaprenyl) diphosphate synthase, subunit 1	PDSS1
6737	FNDC4	0.427	1.625	1.023	0.173	0.029	0.943	fibronectin type III domain containing 4	FNDC4
2909	CCDC85B	0.569	1.625	1.235	0.171	0.308	0.460	coiled-coil domain containing 85B	CCDC85B
2650	CAPZB	0.894	1.625	1.601	0.398	0.046	0.179	capping actin protein of muscle Z-line beta subunit	CAPZB
3376	CEP78	0.922	1.625	0.830	0.500	0.102	0.395	centrosomal protein 78	CEP78
19771	SMPD4	1.059	1.624	1.404	0.613	0.034	0.018	sphingomyelin phosphodiesterase 4	SMPD4
21720	TMEM143	0.711	1.624	1.169	0.034	0.105	0.353	transmembrane protein 143	TMEM143
18327	RPLP2	0.580	1.623	1.330	0.114	0.059	0.493	ribosomal protein lateral stalk subunit P2	RPLP2
6606	FIGNL1	1.121	1.623	0.885	0.720	0.113	0.634	fidgetin like 1	FIGNL1
1834	BRD8	1.004	1.622	1.169	0.957	0.017	0.141	bromodomain containing 8	BRD8
6580	FGFR3	1.055	1.621	1.088	0.516	0.027	0.276	fibroblast growth factor receptor 3	FGFR3
4862	DHPS	0.997	1.621	1.294	0.988	0.009	0.446	deoxyhypusine synthase	DHPS
22489	TST	0.828	1.621	1.159	0.029	0.016	0.427	thiosulfate sulfurtransferase	TST
1177	ARMC6	0.843	1.621	1.444	0.459	0.107	0.226	armadillo repeat containing 6	ARMC6
1208	ARRB2	0.783	1.621	1.198	0.359	0.268	0.560	arrestin beta 2	ARRB2
14657	NOSIP	1.071	1.621	1.792	0.768	0.030	0.386	nitric oxide synthase interacting protein	NOSIP
11224	LOC10028891	0.319	1.620	0.892	0.116	0.120	0.707	uncharacterized LOC100288911	LOC100288911
13778	MRPS11	1.113	1.620	1.357	0.398	0.157	0.065	mitochondrial ribosomal protein S11	MRPS11

11343	LOC10050684	1.191	1.620	1.525	0.403	0.005	0.289	uncharacterized LOC100506844	LOC100506844
23093	VBP1	1.102	1.620	1.191	0.456	0.029	0.393	von Hippel-Lindau binding protein 1	VBP1
18318	RPL6	0.562	1.619	1.126	0.039	0.058	0.417	ribosomal protein L6	RPL6
17292	PTMA	1.054	1.619	1.835	0.828	0.095	0.323	prothymosin, alpha	PTMA
18372	RPS28	0.582	1.618	1.286	0.092	0.016	0.468	ribosomal protein S28	RPS28
5141	DOHH	1.362	1.617	1.656	0.202	0.007	0.260	deoxyhypusine hydroxylase/monooxygenase	DOHH
1464	ATPIF1	0.865	1.617	1.266	0.420	0.114	0.156	ATPase inhibitory factor 1	ATPIF1
15611	PABPC1L	0.586	1.617	1.089	0.568	0.235	0.897	poly(A) binding protein cytoplasmic 1 like	PABPC1L
6817	FOXRED1	1.034	1.617	1.394	0.778	0.169	0.154	FAD-dependent oxidoreductase domain containing 1	FOXRED1
7338	GLT8D2	0.396	1.616	1.104	0.063	0.388	0.600	glycosyltransferase 8 domain containing 2	GLT8D2
8614	IDH3B	0.775	1.615	1.427	0.020	0.018	0.300	isocitrate dehydrogenase 3 (NAD(+)) beta	IDH3B
6250	FAM57B	1.016	1.615	1.027	0.426	0.081	0.425	family with sequence similarity 57 member B	FAM57B
18316	RPL41	0.616	1.615	1.098	0.103	0.049	0.705	ribosomal protein L41	RPL41
19070	SH3BGR	0.603	1.615	0.741	0.033	0.045	0.005	SH3 domain binding glutamate rich protein	SH3BGR
18394	RPS7	0.593	1.615	1.024	0.068	0.067	0.938	ribosomal protein S7	RPS7
24288	ZNF789	0.743	1.615	0.837	0.267	0.059	0.449	zinc finger protein 789	ZNF789
1301	ASRGL1	0.894	1.614	1.164	0.193	0.015	0.266	asparaginase like 1	ASRGL1
7672	GRIA1	0.269	1.614	0.340	0.106	0.568	0.118	glutamate ionotropic receptor AMPA type subunit 1	GRIA1
2346	C5	0.653	1.614	1.108	0.057	0.008	0.509	complement component 5	C5
3351	CEP131	0.844	1.614	1.224	0.559	0.053	0.574	centrosomal protein 131	CEP131
15555	OVCA2	0.848	1.612	1.316	0.450	0.173	0.095	ovarian tumor suppressor candidate 2	OVCA2
14623	NOC4L	1.108	1.612	1.756	0.650	0.038	0.323	nucleolar complex associated 4 homolog	NOC4L
7767	GSTO1	1.165	1.612	1.655	0.695	0.153	0.318	glutathione S-transferase omega 1	GSTO1
3379	CEP85	1.045	1.611	1.020	0.811	0.066	0.906	centrosomal protein 85	CEP85
22659	TXN2	0.572	1.611	1.327	0.078	0.064	0.435	thioredoxin 2	TXN2
6896	FTSJ3	0.846	1.611	1.272	0.040	0.014	0.539	FtsJ homolog 3	FTSJ3
5283	DUS3L	0.928	1.611	1.282	0.431	0.056	0.487	dihydrouridine synthase 3 like	DUS3L
2118	C19orf25	0.847	1.610	1.355	0.601	0.045	0.489	chromosome 19 open reading frame 25	C19orf25
20929	SURF4	1.776	1.610	1.816	0.011	0.093	0.223	surfeit 4	SURF4
4365	CUEDC2	0.849	1.610	1.564	0.124	0.023	0.403	CUE domain containing 2	CUEDC2
9339	KDELC2	0.955	1.610	1.018	0.893	0.274	0.927	KDEL motif containing 2	KDELC2
16827	PPP2R5C	0.777	1.610	1.104	0.260	0.092	0.533	protein phosphatase 2 regulatory subunit B', gamma	PPP2R5C
3619	CITED4	0.989	1.610	0.847	0.938	0.102	0.133	Cbp/p300 interacting transactivator with Glu/Asp rich carboxy-termi	CITED4
4359	CTU2	1.029	1.609	1.507	0.755	0.075	0.094	cytosolic thiouridylase subunit 2	CTU2
435	ADSL	0.813	1.608	1.278	0.390	0.019	0.350	adenylosuccinate lyase	ADSL
7815	GTPBP4	1.470	1.608	1.588	0.004	0.036	0.252	GTP binding protein 4	GTPBP4
22926	UQCR10	1.204	1.607	1.611	0.389	0.148	0.002	ubiquinol-cytochrome c reductase, complex III subunit X	UQCR10
1319	ATAD5	0.890	1.607	0.935	0.502	0.070	0.631	ATPase family, AAA domain containing 5	ATAD5
18474	RTKN2	0.813	1.607	0.948	0.031	0.027	0.684	rhotekin 2	RTKN2
11901	LOC10192908	0.693	1.607	1.062	0.177	0.080	0.703	uncharacterized LOC101929089	LOC101929089
18262	RPL13AP5	0.494	1.607	1.107	0.174	0.099	0.822	ribosomal protein L13a pseudogene 5	RPL13AP5
15604	P4HA3	3.034	1.607	2.222	0.268	0.412	0.536	prolyl 4-hydroxylase subunit alpha 3	P4HA3
18683	SCARNA6	0.349	1.607	0.349	0.051	0.423	0.051	small Cajal body-specific RNA 6	SCARNA6
4014	COX6C	0.874	1.607	1.362	0.054	0.002	0.061	cytochrome c oxidase subunit 6C	COX6C
22277	TRIM29	0.875	1.606	0.991	0.423	0.348	0.982	tripartite motif containing 29	TRIM29
8682	IFRD2	1.168	1.606	1.362	0.253	0.253	0.067	interferon-related developmental regulator 2	IFRD2
18096	RNF152	1.125	1.606	1.339	0.132	0.132	0.390	ring finger protein 152	RNF152
20826	STRA6	1.064	1.606	1.122	0.043	0.094	0.009	stimulated by retinoic acid 6	STRA6
21550	TIMM17B	1.262	1.606	1.607	0.308	0.129	0.293	translocase of inner mitochondrial membrane 17 homolog B (yeast)	TIMM17B
10097	LIN52	0.977	1.606	0.926	0.903	0.070	0.799	lin-52 DREAM MuvB core complex component	LIN52
52	ABCA6	0.530	1.606	1.297	0.051	0.389	0.527	ATP binding cassette subfamily A member 6	ABCA6
18293	RPL29	0.509	1.606	1.033	0.030	0.065	0.914	ribosomal protein L29	RPL29
11122	LOC10013041	1.304	1.605	3.490	0.199	0.451	0.404	uncharacterized LOC100130417	LOC100130417
5156	DPCD	0.669	1.604	1.727	0.422	0.081	0.415	deleted in primary ciliary dyskinesia homolog (mouse)	DPCD
18324	RPLP0	0.469	1.604	1.238	0.101	0.046	0.493	ribosomal protein lateral stalk subunit P0	RPLP0
14650	NOP56	1.162	1.604	1.264	0.170	0.268	0.086	NOP56 ribonucleoprotein	NOP56
9065	ITGB3	0.998	1.604	1.504	0.996	0.184	0.564	integrin subunit beta 3	ITGB3
10349	LINC00601	0.985	1.602	1.035	0.890	0.080	0.675	long intergenic non-protein coding RNA 601	LINC00601
22672	TXNL4A	1.027	1.602	1.472	0.801	0.002	0.117	thioredoxin like 4A	TXNL4A
1467	ATRIP	0.970	1.602	1.189	0.814	0.074	0.402	ATR interacting protein	ATRIP
2670	CASC10	0.580	1.602	1.230	0.056	0.432	0.714	cancer susceptibility candidate 10	CASC10
16766	PPP1CA	1.015	1.600	1.597	0.749	0.016	0.107	protein phosphatase 1 catalytic subunit alpha	PPP1CA
8503	HSPA14	1.040	1.600	1.068	0.554	0.001	0.519	heat shock protein family A (Hsp70) member 14	HSPA14
23169	VPS29	0.813	1.600	1.196	0.150	0.034	0.122	VPS29 retromer complex component	VPS29
22912	UPF3B	0.840	1.600	1.187	0.175	0.016	0.652	UPF3 regulator of nonsense transcripts homolog B (yeast)	UPF3B
5916	EYA2	0.965	1.600	1.739	0.874	0.089	0.381	EYA transcriptional coactivator and phosphatase 2	EYA2
7904	HADHB	0.668	1.600	1.349	0.011	0.062	0.206	hydroxyacyl-CoA dehydrogenase/3-ketoacyl-CoA thiolase/enoyl-CoA	HADHB
18283	RPL23AP7	0.722	1.600	1.329	0.131	0.052	0.041	ribosomal protein L23a pseudogene 7	RPL23AP7
19423	SLC2A8	0.944	1.600	0.974	0.567	0.007	0.807	solute carrier family 2 member 8	SLC2A8
1059	ARHGAP19	0.881	1.600	0.972	0.082	0.054	0.643	Rho GTPase activating protein 19	ARHGAP19
8009	HDGFRP2	0.784	1.600	1.326	0.070	0.000	0.350	hepatoma-derived growth factor-related protein 2	HDGFRP2
24287	ZNF788	0.839	1.599	0.909	0.202	0.030	0.203	zinc finger family member 788	ZNF788
4015	COX7A1	0.614	1.598	1.058	0.065	0.076	0.756	cytochrome c oxidase subunit 7A1	COX7A1
14330	NDUFA4	0.954	1.598	1.365	0.857	0.212	0.490	NDUFA4, mitochondrial complex associated	NDUFA4
17033	PROSER3	0.891	1.598	1.091	0.570	0.025	0.578	proline and serine rich 3	PROSER3
21737	TMEM158	5.123	1.598	1.597	0.016	0.149	0.133	transmembrane protein 158 (gene/pseudogene)	TMEM158
18511	RWDD2B	0.883	1.598	1.521	0.000	0.000	0.019	RWD domain containing 2B	RWDD2B
13317	MESP1	0.779	1.597	1.247	0.478	0.136	0.396	mesoderm posterior bHLH transcription factor 1	MESP1
15617	PABPC4	0.389	1.597	0.955	0.091	0.092	0.858	poly(A) binding protein cytoplasmic 4	PABPC4
17539	RAD21	1.221	1.597	1.275	0.413	0.183	0.176	RAD21 cohesin complex component	RAD21
16242	PIGX	1.086	1.596	1.028	0.253	0.025	0.741	phosphatidylinositol glycan anchor biosynthesis class X	PIGX
13755	MRPL39	1.191	1.596	1.088	0.432	0.070	0.716	mitochondrial ribosomal protein L39	MRPL39
1090	ARHGDIA	0.783	1.595	1.337	0.013	0.004	0.029	Rho GDP dissociation inhibitor alpha	ARHGDIA
14325	NDUFA11	0.927	1.595	1.794	0.753	0.000	0.312	NADH:ubiquinone oxidoreductase subunit A11	NDUFA11
9112	JADE1	0.634	1.594	0.946	0.246	0.069	0.829	jade family PHD finger 1	JADE1
139	ACAD9	0.880	1.594	1.280	0.168	0.015	0.087	acyl-CoA dehydrogenase family member 9	ACAD9
14688	NPDC1	0.526	1.594	1.100	0.163	0.180	0.667	neural proliferation, differentiation and control, 1	NPDC1
16621	POLR3F	0.895	1.594	1.162	0.053	0.014	0.382	polymerase (RNA) III subunit F	POLR3F
370	ADGRE5	0.889	1.594	0.994	0.780	0.237	0.985	adhesion G protein-coupled receptor E5	ADGRE5
21415	TFDP1	0.859	1.594	1.123	0.408	0.010	0.632	transcription factor Dp-1	TFDP1
4683	DDT	1.057	1.594	1.510	0.609	0.105	0.004	D-dopachrome tautomerase	DDT

3155	CDC7	1.549	1.594	1.430	0.255	0.111	0.051	cell division cycle 7	CDC7
7594	GPR3	1.099	1.593	0.993	0.462	0.066	0.848	G protein-coupled receptor 3	GPR3
18663	SCARF2	0.397	1.593	0.812	0.055	0.251	0.185	scavenger receptor class F member 2	SCARF2
22010	TNFRSF13C	0.898	1.592	1.239	0.423	0.014	0.441	tumor necrosis factor receptor superfamily member 13C	TNFRSF13C
22958	USP1	0.975	1.592	0.894	0.777	0.014	0.231	ubiquitin specific peptidase 1	USP1
4107	CREB3L4	0.573	1.592	1.155	0.141	0.057	0.544	cAMP responsive element binding protein 3-like 4	CREB3L4
18267	RPL17	0.549	1.592	1.047	0.045	0.049	0.700	ribosomal protein L17	RPL17
18349	RPS14	0.461	1.590	1.022	0.026	0.072	0.951	ribosomal protein S14	RPS14
20917	SUPT16H	0.823	1.590	1.190	0.285	0.134	0.565	SPT16 homolog, facilitates chromatin remodeling subunit	SUPT16H
3843	CNRIP1	0.809	1.589	1.271	0.554	0.073	0.390	cannabinoid receptor interacting protein 1	CNRIP1
9270	KCNK12	0.784	1.589	0.910	0.470	0.427	0.774	potassium two pore domain channel subfamily K member 12	KCNK12
20841	STUB1	0.831	1.589	1.309	0.221	0.054	0.215	STIP1 homology and U-box containing protein 1	STUB1
20782	STIP1	1.213	1.589	1.268	0.001	0.011	0.130	stress induced phosphoprotein 1	STIP1
96	ABHD11	0.816	1.589	0.907	0.079	0.184	0.219	abhydrolase domain containing 11	ABHD11
8135	HIST1H1B	1.006	1.589	1.136	0.952	0.079	0.427	histone cluster 1, H1b	HIST1H1B
15968	PDF	0.877	1.588	1.151	0.262	0.143	0.300	peptide deformylase (mitochondrial)	PDF
23535	YWHAE	1.013	1.588	1.423	0.908	0.016	0.085	tyrosine 3-monooxygenase/tryptophan 5-monooxygenase activation	YWHAE
22946	UROD	0.594	1.588	1.516	0.065	0.009	0.073	uroporphyrinogen decarboxylase	UROD
21553	TIMM23	1.190	1.588	1.549	0.191	0.017	0.064	translocase of inner mitochondrial membrane 23	TIMM23
11461	LOC10192694	1.258	1.587	1.044	0.494	0.107	0.914	uncharacterized LOC101926940	LOC101926940
24249	ZNF736	0.623	1.587	0.826	0.035	0.063	0.244	zinc finger protein 736	ZNF736
18138	RNF25	0.931	1.587	1.288	0.585	0.076	0.208	ring finger protein 25	RNF25
22289	TRIM39-RPP2	0.811	1.587	1.602	0.486	0.516	0.089	TRIM39-RPP21 readthrough	TRIM39-RPP21
7397	GNB2L1	0.435	1.586	0.976	0.031	0.008	0.892	.	GNB2L1
19782	SMUG1	1.039	1.586	1.542	0.550	0.070	0.162	single-strand-selective monofunctional uracil-DNA glycosylase 1	SMUG1
18532	S100A16	0.498	1.584	1.274	0.036	0.062	0.104	S100 calcium binding protein A16	S100A16
6029	FAM133DP	0.561	1.584	1.123	0.446	0.034	0.817	family with sequence similarity 133 member D, pseudogene	FAM133DP
4742	DEF8	0.727	1.584	1.161	0.004	0.023	0.390	differentially expressed in FDCP 8 homolog (mouse)	DEF8
18317	RPL5	0.430	1.583	0.953	0.020	0.086	0.782	ribosomal protein L5	RPL5
17356	PUF60	1.017	1.583	1.602	0.899	0.007	0.290	poly(U) binding splicing factor 60KDa	PUF60
3866	CNTRL	0.891	1.583	1.079	0.040	0.102	0.073	centriolin	CNTRL
4663	DDAH1	0.553	1.583	0.900	0.151	0.147	0.764	dimethylarginine dimethylaminohydrolase 1	DDAH1
37	AATF	0.978	1.582	1.302	0.921	0.041	0.557	apoptosis antagonizing transcription factor	AATF
10119	LINC00116	0.895	1.582	1.385	0.318	0.001	0.112	long intergenic non-protein coding RNA 116	LINC00116
1402	ATP5A1	0.810	1.582	1.435	0.308	0.025	0.267	ATP synthase, H+ transporting, mitochondrial F1 complex, alpha subu	ATP5A1
18305	RPL36	0.667	1.581	1.204	0.030	0.041	0.091	ribosomal protein L36	RPL36
13733	MRPL16	0.925	1.581	1.492	0.549	0.069	0.014	mitochondrial ribosomal protein L16	MRPL16
2635	CAPN5	0.623	1.580	1.181	0.321	0.056	0.704	calpain 5	CAPN5
3373	CEP70	0.843	1.579	1.063	0.422	0.103	0.661	centrosomal protein 70	CEP70
17886	RFXANK	0.976	1.579	1.786	0.884	0.003	0.157	regulatory factor X associated ankyrin containing protein	RFXANK
22949	USE1	0.720	1.579	1.479	0.123	0.110	0.282	unconventional SNARE in the ER 1	USE1
20853	STX1A	1.343	1.579	1.290	0.438	0.145	0.403	syntaxin 1A	STX1A
5767	ERCC1	0.966	1.578	1.391	0.830	0.020	0.202	excision repair cross-complementation group 1	ERCC1
5173	DPM2	0.932	1.578	1.351	0.719	0.234	0.049	dolichyl-phosphate mannosyltransferase polypeptide 2, regulatory su	DPM2
19335	SLC25A14	1.094	1.578	1.425	0.569	0.012	0.164	solute carrier family 25 member 14	SLC25A14
14501	NHSL1	0.923	1.578	1.038	0.620	0.368	0.897	NHS like 1	NHSL1
332	ADAT1	1.087	1.578	1.390	0.263	0.127	0.346	adenosine deaminase, tRNA specific 1	ADAT1
1988	C11orf94	1.000	1.577	1.130	0.714	0.000	0.423	chromosome 11 open reading frame 94	C11orf94
12636	LPXN	1.193	1.576	1.316	0.732	0.347	0.438	leupaxin	LPXN
262	ACY1	1.081	1.575	2.075	0.114	0.000	0.230	aminoacylase 1	ACY1
18265	RPL14	0.504	1.575	0.978	0.033	0.042	0.900	ribosomal protein L14	RPL14
5530	EIF3G	0.728	1.575	1.319	0.188	0.002	0.471	eukaryotic translation initiation factor 3 subunit G	EIF3G
13687	MPV17L2	1.330	1.575	1.104	0.540	0.340	0.732	MPV17 mitochondrial inner membrane protein like 2	MPV17L2
22529	TTC39C	1.129	1.575	1.499	0.629	0.115	0.237	tetratricopeptide repeat domain 39C	TTC39C
7375	GMPS	0.938	1.575	1.050	0.172	0.008	0.584	guanine monophosphate synthase	GMPS
6275	FAM72A	0.991	1.574	1.341	0.878	0.012	0.026	family with sequence similarity 72 member A	FAM72A
7803	GTF3A	0.994	1.574	1.412	0.945	0.073	0.289	general transcription factor IIIA	GTF3A
5514	EIF2B2	1.237	1.574	1.794	0.415	0.042	0.190	eukaryotic translation initiation factor 2B subunit beta	EIF2B2
18348	RPS13	0.543	1.574	0.977	0.010	0.030	0.617	ribosomal protein S13	RPS13
1852	BRMS1	0.939	1.574	1.511	0.459	0.019	0.386	breast cancer metastasis suppressor 1	BRMS1
7154	GCSH	0.700	1.574	1.020	0.167	0.037	0.912	glycine cleavage system protein H	GCSH
18872	SEMA7A	0.483	1.574	0.870	0.083	0.063	0.784	semaphorin 7A (John Milton Hagen blood group)	SEMA7A
17158	PSENEN	0.982	1.573	1.466	0.908	0.057	0.375	presenilin enhancer gamma secretase subunit	PSENEN
14349	NDUFB11	0.995	1.573	1.623	0.983	0.047	0.343	NADH:ubiquinone oxidoreductase subunit B11	NDUFB11
7775	GSTZ1	1.151	1.573	1.970	0.647	0.155	0.342	glutathione S-transferase zeta 1	GSTZ1
22911	UPF3A	0.826	1.572	1.176	0.055	0.023	0.673	UPF3 regulator of nonsense transcripts homolog A (yeast)	UPF3A
13144	MBD3	0.967	1.572	1.431	0.834	0.071	0.307	methyl-CpG binding domain protein 3	MBD3
941	APEH	0.963	1.572	1.458	0.527	0.011	0.100	acylaminoacyl-peptide hydrolase	APEH
18717	SCLT1	1.055	1.572	1.056	0.721	0.081	0.689	sodium channel and clathrin linker 1	SCLT1
5398	EDC4	0.879	1.571	1.268	0.252	0.053	0.067	enhancer of mRNA decapping 4	EDC4
14357	NDUFB8	0.983	1.571	1.735	0.879	0.000	0.305	NADH:ubiquinone oxidoreductase subunit B8	NDUFB8
15932	PDCD5	2.441	1.571	1.542	0.034	0.108	0.001	programmed cell death 5	PDCD5
4605	DCAF15	1.188	1.571	1.051	0.055	0.081	0.089	DDB1 and CUL4 associated factor 15	DCAF15
18052	RNASEH2C	0.881	1.570	1.290	0.187	0.042	0.504	ribonuclease H2 subunit C	RNASEH2C
19365	SLC25A39	0.823	1.570	1.343	0.330	0.166	0.093	solute carrier family 25 member 39	SLC25A39
4018	COX7B	1.527	1.570	1.643	0.044	0.085	0.181	cytochrome c oxidase subunit 7B	COX7B
15799	PCCB	1.016	1.569	1.735	0.920	0.034	0.033	propionyl-CoA carboxylase beta subunit	PCCB
4383	CWF19L1	0.791	1.569	1.005	0.208	0.014	0.974	CWF19-like 1, cell cycle control (S. pombe)	CWF19L1
14936	NUP62	1.159	1.569	1.381	0.622	0.207	0.083	nucleoporin 62kDa	NUP62
18312	RPL39	0.604	1.569	1.320	0.213	0.146	0.611	ribosomal protein L39	RPL39
2502	CA5B	0.528	1.569	1.077	0.170	0.391	0.791	carbonic anhydrase 5B	CA5B
1387	ATP1B3	1.448	1.569	1.494	0.271	0.108	0.289	ATPase Na+/K+ transporting subunit beta 3	ATP1B3
16306	PITX1	0.738	1.568	1.419	0.617	0.636	0.608	paired like homeodomain 1	PITX1
1363	ATOX1	1.092	1.568	1.766	0.634	0.076	0.177	antioxidant 1 copper chaperone	ATOX1
1551	B9D2	1.138	1.568	1.497	0.553	0.262	0.261	B9 protein domain 2	B9D2
18352	RPS15A	0.520	1.568	0.971	0.021	0.064	0.535	ribosomal protein S15a	RPS15A
16080	PFDN6	1.626	1.568	1.788	0.084	0.091	0.220	prefoldin subunit 6	PFDN6
13271	MED4-AS1	0.626	1.568	1.028	0.200	0.121	0.910	MED4 antisense RNA 1	MED4-AS1
1630	BCCIP	1.317	1.567	1.601	0.092	0.013	0.160	BRCA2 and CDKN1A interacting protein	BCCIP
14933	NUP50	1.252	1.567	1.269	0.354	0.187	0.391	nucleoporin 50kDa	NUP50
3366	CEP41	0.893	1.567	1.246	0.160	0.009	0.003	centrosomal protein 41	CEP41

18878	SENP3	1.054	1.567	1.303	0.694	0.068	0.232	SUMO1/sentrin/SMT3 specific peptidase 3	SENP3
18359	RPS19	0.497	1.566	1.160	0.110	0.087	0.634	ribosomal protein S19	RPS19
19501	SLC39A4	0.527	1.566	1.179	0.335	0.111	0.756	solute carrier family 39 member 4	SLC39A4
1405	ATP5D	0.814	1.566	1.551	0.601	0.091	0.436	ATP synthase, H+ transporting, mitochondrial F1 complex, delta subu	ATP5D
19001	SF3B5	0.951	1.566	1.594	0.675	0.011	0.221	splicing factor 3b subunit 5	SF3B5
20595	SRI	0.870	1.566	1.940	0.394	0.100	0.080	sorcin	SRI
12839	LY6G5C	0.948	1.565	1.269	0.433	0.028	0.526	lymphocyte antigen 6 complex, locus G5C	LY6G5C
16852	PPT1	1.095	1.565	1.444	0.143	0.002	0.038	palmitoyl-protein thioesterase 1	PPT1
1506	AXL	1.046	1.564	1.874	0.790	0.095	0.008	AXL receptor tyrosine kinase	AXL
5857	ETV4	1.621	1.564	1.048	0.044	0.281	0.753	ETS variant 4	ETV4
13864	MSTO1	1.209	1.564	1.518	0.062	0.016	0.079	misato 1, mitochondrial distribution and morphology regulator	MSTO1
17151	PSCA	0.693	1.563	1.551	0.457	0.465	0.573	prostate stem cell antigen	PSCA
1199	ARPC4	0.650	1.563	1.196	0.030	0.027	0.125	actin related protein 2/3 complex subunit 4	ARPC4
22154	TPM1	1.911	1.563	1.458	0.284	0.309	0.256	tropomyosin 1 (alpha)	TPM1
1741	BLMH	0.524	1.563	1.021	0.045	0.106	0.947	bleomycin hydrolase	BLMH
14348	NDUFB10	0.923	1.562	1.300	0.158	0.029	0.138	NADH:ubiquinone oxidoreductase subunit B10	NDUFB10
14467	NFKBIB	1.175	1.562	1.361	0.637	0.157	0.458	NFKB inhibitor beta	NFKBIB
8855	IMMP1L	0.977	1.562	1.490	0.836	0.040	0.210	inner mitochondrial membrane peptidase subunit 1	IMMP1L
18362	RPS20	0.518	1.561	1.090	0.095	0.065	0.747	ribosomal protein S20	RPS20
14079	MYO19	1.158	1.561	1.396	0.383	0.095	0.035	myosin XIX	MYO19
22611	TUBB2A	0.729	1.561	1.098	0.450	0.440	0.673	tubulin beta 2A class IIa	TUBB2A
14190	NAP1L4	0.952	1.559	1.090	0.707	0.017	0.489	nucleosome assembly protein 1 like 4	NAP1L4
18255	RPL11	0.496	1.559	1.109	0.083	0.088	0.783	ribosomal protein L11	RPL11
7492	GOT1	1.146	1.558	1.304	0.402	0.116	0.055	glutamic-oxaloacetic transaminase 1	GOT1
13403	MGAT5B	1.300	1.558	1.130	0.032	0.192	0.411	mannosyl (alpha-1,6-)-glycoprotein beta-1,6-N-acetyl-glucosaminyltra	MGAT5B
11011	LKAAEAR1	0.904	1.558	1.034	0.361	0.021	0.884	LKAAEAR motif containing 1	LKAAEAR1
14352	NDUFB3	1.236	1.557	1.441	0.436	0.136	0.203	NADH:ubiquinone oxidoreductase subunit B3	NDUFB3
5969	FAH	1.161	1.557	1.733	0.508	0.001	0.144	fumarylacetoacetate hydrolase (fumarylacetoacetase)	FAH
19697	SMARCD2	0.665	1.557	1.648	0.183	0.034	0.210	SWI/SNF related, matrix associated, actin dependent regulator of chr	SMARCD2
17716	RBM3	0.519	1.557	1.468	0.200	0.230	0.281	RNA binding motif (RNP1, RRM) protein 3	RBM3
4738	DECR2	0.756	1.557	1.979	0.544	0.177	0.253	2,4-dienoyl-CoA reductase 2, peroxisomal	DECR2
4404	CXCL9	1.264	1.556	1.000	0.026	0.506	0.943	C-X-C motif chemokine ligand 9	CXCL9
21552	TIMM22	0.807	1.556	1.377	0.239	0.085	0.222	translocase of inner mitochondrial membrane 22 homolog (yeast)	TIMM22
17209	PSMD4	1.025	1.556	1.673	0.783	0.006	0.254	proteasome 26S subunit, non-ATPase 4	PSMD4
23314	WDR61	0.853	1.556	1.297	0.410	0.059	0.283	WD repeat domain 61	WDR61
5419	EEF1D	0.612	1.556	1.385	0.439	0.219	0.672	eukaryotic translation elongation factor 1 delta	EEF1D
6876	FSIP1	1.126	1.555	1.208	0.394	0.211	0.213	fibrous sheath interacting protein 1	FSIP1
483	AGMAT	0.948	1.555	1.286	0.469	0.008	0.025	agmatinase	AGMAT
8435	HRCT1	0.622	1.554	1.685	0.619	0.580	0.564	histidine rich carboxyl terminus 1	HRCT1
6365	FARSA	1.379	1.554	1.517	0.033	0.131	0.106	phenylalanyl-tRNA synthetase alpha subunit	FARSA
20872	STYXL1	0.938	1.554	1.775	0.862	0.115	0.411	serine/threonine/tyrosine interacting-like 1	STYXL1
17533	RAC3	0.359	1.554	0.987	0.034	0.231	0.974	ras-related C3 botulinum toxin substrate 3 (rho family, small GTP binc	RAC3
21303	TCTEX1D2	1.003	1.554	1.169	0.987	0.171	0.221	Tctex1 domain containing 2	TCTEX1D2
13765	MRPL46	1.237	1.554	1.351	0.239	0.252	0.041	mitochondrial ribosomal protein L46	MRPL46
18377	RPS4X	0.434	1.554	0.971	0.003	0.105	0.274	ribosomal protein S4, X-linked	RPS4X
13783	MRPS17	1.062	1.553	1.532	0.431	0.065	0.219	mitochondrial ribosomal protein S17	MRPS17
8603	ICT1	1.108	1.553	1.238	0.169	0.042	0.222	immature colon carcinoma transcript 1	ICT1
17045	PRPF4	1.015	1.552	1.121	0.592	0.016	0.524	pre-mRNA processing factor 4	PRPF4
21197	TBCA	1.073	1.552	1.365	0.588	0.048	0.351	tubulin folding cofactor A	TBCA
7191	GFER	0.832	1.551	1.342	0.354	0.068	0.414	growth factor, augmenter of liver regeneration	GFER
5529	EIF3F	0.301	1.551	0.796	0.012	0.049	0.148	eukaryotic translation initiation factor 3 subunit F	EIF3F
19073	SH3BGRL3	0.724	1.551	1.807	0.430	0.215	0.195	SH3 domain binding glutamate rich protein like 3	SH3BGRL3
5621	EMD	0.828	1.551	1.367	0.317	0.019	0.088	emerin	EMD
13671	MPND	0.664	1.551	1.332	0.023	0.044	0.380	MPN domain containing	MPND
1822	BRAT1	0.848	1.551	1.256	0.101	0.005	0.399	BRCA1 associated ATM activator 1	BRAT1
14857	NTMT1	1.067	1.551	1.734	0.751	0.038	0.367	N-terminal Xaa-Pro-Lys N-methyltransferase 1	NTMT1
7889	H3F3AP4	0.916	1.550	1.375	0.312	0.017	0.189	H3 histone, family 3A, pseudogene 4	H3F3AP4
18445	RSG1	0.643	1.550	1.219	0.070	0.073	0.196	REM2 and RAB like small GTPase 1	RSG1
23534	YWHAB	0.745	1.549	1.245	0.013	0.008	0.120	tyrosine 3-monooxygenase/tryptophan 5-monooxygenase activation	YWHAB
17419	QARS	0.552	1.549	1.226	0.032	0.021	0.556	glutaminyl-tRNA synthetase	QARS
1472	ATXN10	1.008	1.549	1.253	0.708	0.080	0.078	ataxin 10	ATXN10
12156	LOC10272471	0.802	1.549	1.055	0.002	0.423	0.522	uncharacterized LOC102724719	LOC102724719
87	ABCF2	1.071	1.549	1.274	0.654	0.134	0.129	ATP binding cassette subfamily F member 2	ABCF2
14356	NDUFB7	0.957	1.549	1.833	0.835	0.058	0.298	NADH:ubiquinone oxidoreductase subunit B7	NDUFB7
22927	UQCR11	0.979	1.548	1.737	0.859	0.022	0.272	ubiquinol-cytochrome c reductase, complex III subunit XI	UQCR11
1743	BLOC1S1	1.223	1.548	1.787	0.087	0.007	0.230	biogenesis of lysosomal organelles complex 1 subunit 1	BLOC1S1
18803	SEC11C	2.007	1.548	0.940	0.187	0.518	0.853	SEC11 homolog C, signal peptidase complex subunit	SEC11C
6393	FBN2	1.764	1.548	3.203	0.561	0.584	0.132	fibrillin 2	FBN2
7502	GPAA1	0.767	1.548	1.439	0.333	0.078	0.355	glycosylphosphatidylinositol anchor attachment 1	GPAA1
11220	LOC10028879	0.582	1.548	1.158	0.566	0.364	0.820	uncharacterized LOC100288798	LOC100288798
21742	TMEM161B-A	1.050	1.548	1.639	0.579	0.034	0.156	TMEM161B antisense RNA 1	TMEM161B-AS1
5117	DNMT3B	0.861	1.548	0.960	0.505	0.105	0.798	DNA (cytosine-5-)-methyltransferase 3 beta	DNMT3B
2923	CCDC96	0.713	1.547	0.878	0.298	0.042	0.702	coiled-coil domain containing 96	CCDC96
13872	MT1E	4.832	1.547	3.013	0.112	0.242	0.015	metallothionein 1E	MT1E
109	ABHD17C	1.131	1.547	1.240	0.147	0.390	0.601	abhydrolase domain containing 17C	ABHD17C
7623	GPR89A	0.872	1.546	1.180	0.415	0.132	0.077	G protein-coupled receptor 89A	GPR89A
903	AP1S1	0.647	1.546	1.403	0.117	0.116	0.101	adaptor related protein complex 1 sigma 1 subunit	AP1S1
3387	CERK	0.641	1.545	0.797	0.289	0.047	0.512	ceramide kinase	CERK
17652	RASIP1	1.237	1.545	1.398	0.070	0.001	0.358	Ras interacting protein 1	RASIP1
3324	CENPB	0.869	1.545	1.164	0.205	0.023	0.254	centromere protein B	CENPB
7179	GDPGP1	0.686	1.545	0.823	0.227	0.009	0.475	GDP-D-glucose phosphorylase 1	GDPGP1
18503	RUSC1	0.661	1.545	1.082	0.006	0.012	0.549	RUN and SH3 domain containing 1	RUSC1
19800	SNAPC1	1.979	1.545	2.055	0.159	0.437	0.403	small nuclear RNA activating complex polypeptide 1	SNAPC1
16699	PPA2	0.826	1.544	1.139	0.129	0.060	0.494	pyrophosphatase (inorganic) 2	PPA2
5355	E2F7	0.909	1.544	1.060	0.580	0.019	0.753	E2F transcription factor 7	E2F7
1163	ARL6IP4	0.750	1.544	1.323	0.377	0.095	0.565	ADP ribosylation factor like GTPase 6 interacting protein 4	ARL6IP4
1668	BCS1L	0.834	1.543	1.343	0.133	0.294	0.042	BCS1 homolog, ubiquinol-cytochrome c reductase complex chaperon	BCS1L
21551	TIMM21	0.941	1.543	0.857	0.184	0.089	0.092	translocase of inner mitochondrial membrane 21	TIMM21
22928	UQCRB	0.747	1.543	1.173	0.118	0.038	0.667	ubiquinol-cytochrome c reductase binding protein	UQCRB
18279	RPL23A	0.660	1.542	1.081	0.150	0.020	0.739	ribosomal protein L23a	RPL23A
13744	MRPL27	1.253	1.542	1.580	0.332	0.095	0.289	mitochondrial ribosomal protein L27	MRPL27

1234	AS3MT	0.746	1.542	1.067	0.671	0.494	0.857	arsenite methyltransferase	AS3MT
20605	SRP9	0.910	1.542	1.205	0.386	0.061	0.241	signal recognition particle 9kDa	SRP9
21548	TIMM13	0.748	1.542	1.355	0.262	0.124	0.545	translocase of inner mitochondrial membrane 13	TIMM13
17401	PYCARD-AS1	0.458	1.542	1.325	0.027	0.156	0.080	PYCARD antisense RNA 1	PYCARD-AS1
1306	ASTN2	0.506	1.541	1.036	0.024	0.044	0.610	astrotactin 2	ASTN2
16575	POLB	0.897	1.541	1.268	0.227	0.108	0.037	polymerase (DNA) beta	POLB
4711	DDX39B	1.003	1.541	1.677	0.988	0.055	0.127	DEAD-box helicase 39B	DDX39B
229	ACTL6A	0.972	1.540	0.991	0.743	0.040	0.965	actin like 6A	ACTL6A
12974	MAGOHB	1.311	1.540	1.261	0.125	0.026	0.171	mago homolog B, exon junction complex core component	MAGOHB
2117	C19orf24	1.023	1.540	1.512	0.944	0.340	0.143	chromosome 19 open reading frame 24	C19orf24
16053	PES1	0.898	1.540	1.417	0.450	0.085	0.550	pescadillo ribosomal biogenesis factor 1	PES1
7395	GNB1L	0.848	1.540	1.311	0.371	0.186	0.257	G protein subunit beta 1 like	GNB1L
18911	SERBP1	0.789	1.540	1.280	0.092	0.057	0.260	SERPINE1 mRNA binding protein 1	SERBP1
2053	C16orf13	0.819	1.539	1.404	0.409	0.147	0.297	chromosome 16 open reading frame 13	C16orf13
7077	GAS2L1	1.024	1.539	1.185	0.793	0.037	0.148	growth arrest specific 2 like 1	GAS2L1
22352	TRMT1	0.870	1.539	1.539	0.253	0.094	0.361	tRNA methyltransferase 1	TRMT1
10123	LINC00160	2.119	1.539	1.312	0.288	0.527	0.422	long intergenic non-protein coding RNA 160	LINC00160
3244	CDO1	0.884	1.538	1.557	0.805	0.370	0.598	cysteine dioxygenase type 1	CDO1
6497	FCRLA	7.033	1.538	2.393	0.385	0.585	0.001	Fc receptor like A	FCRLA
5799	ERLIN1	1.259	1.538	1.323	0.168	0.075	0.126	ER lipid raft associated 1	ERLIN1
18750	SCRIB	0.889	1.537	1.181	0.265	0.010	0.119	scribbled planar cell polarity protein	SCRIB
3342	CENPV	1.042	1.537	0.876	0.927	0.335	0.161	centromere protein V	CENPV
1411	ATP5G3	1.163	1.537	1.327	0.239	0.032	0.024	ATP synthase, H+ transporting, mitochondrial Fo complex subunit C3	ATP5G3
5951	FAAP24	0.803	1.537	0.820	0.530	0.244	0.388	Fanconi anemia core complex associated protein 24	FAAP24
13774	MRPL55	0.959	1.536	1.774	0.558	0.082	0.395	mitochondrial ribosomal protein L55	MRPL55
16922	PRELID1	1.205	1.536	1.634	0.170	0.037	0.033	PRELI domain containing 1	PRELID1
11034	LMNA	0.499	1.535	1.386	0.024	0.012	0.075	lamin A/C	LMNA
18232	RPA1	0.661	1.535	0.873	0.072	0.081	0.226	replication protein A1	RPA1
16518	PMS2P1	0.874	1.535	1.340	0.698	0.060	0.387	PMS1 homolog 2, mismatch repair system component pseudogene 1	PMS2P1
23664	ZDHHC16	1.039	1.535	1.419	0.811	0.089	0.081	zinc finger DHHC-type containing 16	ZDHHC16
15609	PAAF1	0.840	1.534	1.259	0.475	0.032	0.534	proteasomal ATPase associated factor 1	PAAF1
14187	NAP1L1	0.437	1.534	0.995	0.017	0.183	0.971	nucleosome assembly protein 1 like 1	NAP1L1
548	AK6	1.137	1.534	1.162	0.406	0.014	0.501	adenylate kinase 6	AK6
3745	CLPP	1.201	1.534	1.801	0.333	0.081	0.046	caseinolytic mitochondrial matrix peptidase proteolytic subunit	CLPP
22776	UBL5	0.988	1.534	1.489	0.829	0.069	0.091	ubiquitin like 5	UBL5
22788	UBQLN4	1.028	1.534	1.242	0.572	0.061	0.379	ubiquilin 4	UBQLN4
15718	PARK7	0.876	1.534	1.179	0.041	0.020	0.126	Parkinsonism associated deglycase	PARK7
8329	HNRNPUL1	0.760	1.533	1.121	0.010	0.033	0.029	heterogeneous nuclear ribonucleoprotein U like 1	HNRNPUL1
5971	FAHD2A	0.754	1.533	1.182	0.434	0.257	0.524	fumarylacetoacetate hydrolase domain containing 2A	FAHD2A
16100	PGAM5	1.070	1.532	1.187	0.423	0.013	0.305	PGAM family member 5, mitochondrial serine/threonine protein pho	PGAM5
1952	C10orf90	1.554	1.532	1.369	0.011	0.254	0.113	chromosome 10 open reading frame 90	C10orf90
13327	METTL1	1.297	1.532	1.428	0.144	0.196	0.387	methyltransferase like 1	METTL1
8166	HIST1H2BN	1.020	1.532	1.231	0.944	0.121	0.366	histone cluster 1, H2bn	HIST1H2BN
18947	SERPINB6	0.857	1.532	1.325	0.273	0.033	0.433	serpin family B member 6	SERPINB6
18287	RPL24	0.535	1.531	1.033	0.072	0.061	0.915	ribosomal protein L24	RPL24
15871	PCDHGC4	0.738	1.530	0.953	0.133	0.093	0.820	protocadherin gamma subfamily C, 4	PCDHGC4
18304	RPL35A	0.567	1.530	0.889	0.022	0.123	0.385	ribosomal protein L35a	RPL35A
21875	TMEM41A	0.704	1.530	0.918	0.012	0.227	0.166	transmembrane protein 41A	TMEM41A
14355	NDUFB6	1.306	1.530	1.362	0.302	0.038	0.324	NADH:ubiquinone oxidoreductase subunit B6	NDUFB6
2123	C19orf43	0.746	1.529	1.325	0.303	0.045	0.600	chromosome 19 open reading frame 43	C19orf43
2520	CABYR	0.956	1.529	1.235	0.826	0.105	0.219	calcium binding tyrosine phosphorylation regulated	CABYR
14939	NUP88	1.378	1.529	1.108	0.073	0.010	0.522	nucleoporin 88	NUP88
16239	PIGU	1.179	1.529	1.153	0.180	0.092	0.212	phosphatidylinositol glycan anchor biosynthesis class U	PIGU
4726	DDX54	0.928	1.529	1.428	0.383	0.001	0.331	DEAD-box helicase 54	DDX54
22480	TSR3	0.764	1.529	1.644	0.159	0.004	0.327	TSR3, acp transferase ribosome maturation factor	TSR3
13605	MMS22L	0.922	1.528	0.904	0.625	0.027	0.600	MMS22 like, DNA repair protein	MMS22L
12992	MAMSTR	0.547	1.527	1.424	0.045	0.048	0.270	MEF2 activating motif and SAP domain containing transcriptional reg	MAMSTR
1620	BCAP31	0.883	1.527	1.326	0.327	0.017	0.274	B-cell receptor-associated protein 31	BCAP31
4012	COX6B1	0.983	1.527	1.350	0.889	0.072	0.153	cytochrome c oxidase subunit 6B1	COX6B1
3942	COLGALT1	1.071	1.526	1.408	0.292	0.083	0.021	collagen beta(1-O)galactosyltransferase 1	COLGALT1
24386	ZSCAN16	0.797	1.526	0.980	0.279	0.158	0.877	zinc finger and SCAN domain containing 16	ZSCAN16
19855	SNHG9	1.077	1.526	1.398	0.782	0.013	0.393	small nucleolar RNA host gene 9	SNHG9
11758	LOC10192832	0.845	1.525	0.934	0.423	0.037	0.710	uncharacterized LOC101928324	LOC101928324
19419	SLC2A4RG	0.528	1.525	1.016	0.224	0.199	0.965	SLC2A4 regulator	SLC2A4RG
18983	SETD8	0.832	1.524	1.083	0.393	0.008	0.801	.	SETD8
17640	RASD2	0.920	1.523	1.086	0.157	0.171	0.669	RASD family member 2	RASD2
9671	KRT10	0.981	1.523	1.223	0.851	0.127	0.154	keratin 10	KRT10
11645	LOC10192779	0.891	1.523	1.903	0.476	0.417	0.198	uncharacterized LOC101927795	LOC101927795
16043	PELP1	1.050	1.523	1.507	0.766	0.000	0.382	proline, glutamate and leucine rich protein 1	PELP1
1599	BAX	0.673	1.522	1.597	0.025	0.043	0.268	BCL2-associated X protein	BAX
17785	RCN3	1.241	1.522	1.861	0.420	0.014	0.269	reticulocalbin 3	RCN3
22078	TOE1	1.064	1.522	1.083	0.679	0.149	0.607	target of EGR1, member 1 (nuclear)	TOE1
14880	NUCB2	1.694	1.522	1.600	0.005	0.055	0.015	nucleobindin 2	NUCB2
18612	SARS2	0.826	1.521	1.503	0.370	0.066	0.345	seryl-tRNA synthetase 2, mitochondrial	SARS2
22747	UBE2N	1.261	1.521	1.233	0.024	0.017	0.300	ubiquitin conjugating enzyme E2 N	UBE2N
15929	PDCD2L	0.712	1.521	1.076	0.057	0.103	0.401	programmed cell death 2-like	PDCD2L
13673	MPP1	0.777	1.520	1.331	0.137	0.049	0.050	membrane palmitoylated protein 1	MPP1
908	AP2B1	0.779	1.520	1.065	0.393	0.059	0.828	adaptor related protein complex 2 beta 1 subunit	AP2B1
4391	CXCL1	2395.465	1.520	25.809	0.224	0.489	0.361	C-X-C motif chemokine ligand 1	CXCL1
13799	MRPS33	0.815	1.520	1.181	0.062	0.097	0.017	mitochondrial ribosomal protein S33	MRPS33
16034	PECR	0.861	1.519	0.974	0.196	0.032	0.913	peroxisomal trans-2-enoyl-CoA reductase	PECR
19332	SLC25A11	1.308	1.519	1.481	0.067	0.093	0.157	solute carrier family 25 member 11	SLC25A11
6764	FOXC2	0.791	1.518	1.108	0.477	0.275	0.718	forkhead box C2 (MFH-1, mesenchyme forkhead 1)	FOXC2
9104	IVNS1ABP	1.788	1.518	1.279	0.271	0.392	0.314	influenza virus NS1A binding protein	IVNS1ABP
14901	NUDT22	0.933	1.518	1.620	0.395	0.158	0.055	nudix hydrolase 22	NUDT22
3761	CLTA	0.650	1.518	1.111	0.003	0.005	0.423	clathrin light chain A	CLTA
10766	LINC01311	0.923	1.518	1.392	0.755	0.252	0.606	long intergenic non-protein coding RNA 1311	LINC01311
14252	NCAM1	0.775	1.517	1.470	0.019	0.255	0.002	neural cell adhesion molecule 1	NCAM1
20278	SNX5	0.552	1.517	1.109	0.049	0.027	0.523	sorting nexin 5	SNX5
16797	PPP1R35	1.035	1.517	1.578	0.595	0.011	0.010	protein phosphatase 1 regulatory subunit 35	PPP1R35
21097	TANGO6	0.804	1.517	1.205	0.059	0.000	0.060	transport and golgi organization 6 homolog	TANGO6

19007	SFPQ	1.148	1.516	1.335	0.038	0.001	0.021	splicing factor proline/glutamine-rich	SFPQ
12452	LOC541473	0.427	1.516	0.594	0.254	0.544	0.373	FK506 binding protein 6, 36kDa pseudogene	LOC541473
19345	SLC25A22	0.991	1.516	1.419	0.961	0.085	0.128	solute carrier family 25 member 22	SLC25A22
7785	GTF2F1	0.698	1.515	1.058	0.030	0.048	0.815	general transcription factor IIF subunit 1	GTF2F1
6228	FAM43A	0.511	1.515	0.892	0.067	0.533	0.612	family with sequence similarity 43 member A	FAM43A
4635	DCLRE1B	0.951	1.514	1.033	0.825	0.007	0.919	DNA cross-link repair 1B	DCLRE1B
1058	ARHGAP18	0.586	1.514	0.710	0.537	0.133	0.678	Rho GTPase activating protein 18	ARHGAP18
64	ABCB8	1.006	1.514	1.556	0.975	0.075	0.166	ATP binding cassette subfamily B member 8	ABCB8
18432	RRP7A	0.897	1.514	1.271	0.445	0.024	0.384	ribosomal RNA processing 7 homolog A	RRP7A
943	APEX1	0.667	1.514	1.245	0.039	0.010	0.184	apurinic/apyrimidinic endodeoxyribonuclease 1	APEX1
13758	MRPL41	0.911	1.514	1.429	0.608	0.040	0.329	mitochondrial ribosomal protein L41	MRPL41
1116	ARHGEF39	1.044	1.514	1.148	0.840	0.118	0.493	Rho guanine nucleotide exchange factor 39	ARHGEF39
10873	LINC01483	1.162	1.513	1.000	0.423	0.421	0.943	long intergenic non-protein coding RNA 1483	LINC01483
18360	RPS19BP1	0.768	1.513	1.223	0.255	0.050	0.542	ribosomal protein S19 binding protein 1	RPS19BP1
6818	FOXRED2	0.685	1.513	0.821	0.306	0.172	0.472	FAD dependent oxidoreductase domain containing 2	FOXRED2
22650	TWIST2	1.604	1.512	1.575	0.187	0.213	0.166	twist family bHLH transcription factor 2	TWIST2
16648	POP4	0.942	1.511	1.221	0.838	0.228	0.273	POP4 homolog, ribonuclease P/MRP subunit	POP4
5139	DOCK9-AS1	1.000	1.511	1.129	0.714	0.188	0.422	DOCK9 antisense RNA 1	DOCK9-AS1
9731	KRT86	1.207	1.511	1.498	0.398	0.094	0.256	keratin 86	KRT86
16036	PEG10	0.527	1.510	1.719	0.336	0.054	0.578	paternally expressed 10	PEG10
3198	CDK10	0.762	1.510	1.401	0.089	0.115	0.213	cyclin-dependent kinase 10	CDK10
4063	CPS1	0.743	1.510	1.158	0.201	0.042	0.519	carbamoyl-phosphate synthase 1	CPS1
5088	DNAJC8	0.826	1.510	1.120	0.100	0.037	0.094	DnaJ heat shock protein family (Hsp40) member C8	DNAJC8
18276	RPL22	0.481	1.510	1.015	0.085	0.162	0.961	ribosomal protein L22	RPL22
21779	TMEM19	0.742	1.510	0.754	0.147	0.128	0.182	transmembrane protein 19	TMEM19
3817	CNKSR3	0.468	1.509	1.056	0.036	0.187	0.821	CNKSR family member 3	CNKSR3
14922	NUP155	1.094	1.509	1.006	0.638	0.019	0.983	nucleoporin 155kDa	NUP155
14899	NUDT2	0.796	1.509	1.375	0.206	0.151	0.253	nudix hydrolase 2	NUDT2
16588	POLH	0.556	1.509	0.874	0.100	0.068	0.439	polymerase (DNA) eta	POLH
15917	PCYOX1L	0.886	1.509	1.052	0.045	0.003	0.338	prenylcysteine oxidase 1 like	PCYOX1L
17272	PTGES3	0.915	1.509	1.074	0.316	0.021	0.435	prostaglandin E synthase 3	PTGES3
272	ADAM12	1.196	1.508	1.370	0.762	0.534	0.088	ADAM metallopeptidase domain 12	ADAM12
2236	C20orf196	1.182	1.508	1.372	0.190	0.010	0.465	chromosome 20 open reading frame 196	C20orf196
18469	RTEL1	1.126	1.508	1.342	0.110	0.203	0.047	regulator of telomere elongation helicase 1	RTEL1
21558	TIMM8B	0.976	1.508	1.491	0.924	0.159	0.247	translocase of inner mitochondrial membrane 8 homolog B (yeast)	TIMM8B
22604	TUBA3FP	0.750	1.508	1.009	0.167	0.221	0.941	tubulin alpha 3f pseudogene	TUBA3FP
18489	RTTN	1.728	1.508	0.969	0.292	0.229	0.900	rotatin	RTTN
9171	KARS	0.930	1.508	1.347	0.016	0.058	0.015	lysyl-tRNA synthetase	KARS
23326	WDR77	1.090	1.507	1.458	0.218	0.000	0.157	WD repeat domain 77	WDR77
1019	AQP7P3	1.020	1.507	2.397	0.950	0.245	0.036	aquaporin 7 pseudogene 3	AQP7P3
11145	LOC10013131	0.977	1.507	1.090	0.939	0.200	0.756	uncharacterized LOC100131315	LOC100131315
5653	ENHO	0.662	1.507	0.897	0.078	0.291	0.419	energy homeostasis associated	ENHO
8114	HIGD1A	1.242	1.507	1.253	0.248	0.143	0.277	HIG1 hypoxia inducible domain family member 1A	HIGD1A
475	AGBL5	0.659	1.506	1.181	0.055	0.052	0.253	ATP/GTP binding protein-like 5	AGBL5
13295	MEIG1	1.122	1.506	0.989	0.594	0.183	0.899	meiosis/spermiogenesis associated 1	MEIG1
18602	SAPCD1	0.797	1.506	0.798	0.423	0.083	0.424	suppressor APC domain containing 1	SAPCD1
5734	EPN1	0.761	1.506	1.478	0.111	0.035	0.217	epsin 1	EPN1
5266	DTNBP1	0.661	1.506	1.115	0.026	0.017	0.147	dystrobrevin binding protein 1	DTNBP1
897	AP1B1	1.021	1.505	1.517	0.921	0.022	0.097	adaptor related protein complex 1 beta 1 subunit	AP1B1
9087	ITPK1	0.599	1.505	1.007	0.218	0.196	0.974	inositol-tetrakisphosphate 1-kinase	ITPK1
12972	MAGOH	1.142	1.504	1.436	0.072	0.009	0.055	mago homolog, exon junction complex core component	MAGOH
23108	VDAC3	1.104	1.504	1.264	0.471	0.113	0.389	voltage dependent anion channel 3	VDAC3
5225	DRG1	1.024	1.503	1.350	0.879	0.048	0.291	developmentally regulated GTP binding protein 1	DRG1
3962	COPE	1.221	1.503	1.688	0.345	0.083	0.084	coatomer protein complex, subunit epsilon	COPE
5780	ERGIC1	1.107	1.503	1.234	0.325	0.021	0.015	endoplasmic reticulum-golgi intermediate compartment 1	ERGIC1
7106	GATSL2	0.733	1.503	0.905	0.218	0.229	0.597	GATS protein-like 2	GATSL2
9140	JMJD7-PLA2G	1.326	1.503	1.965	0.073	0.002	0.295	JMJD7-PLA2G4B readthrough	JMJD7-PLA2G4B
12690	LRRC20	0.504	1.503	0.862	0.041	0.119	0.369	leucine rich repeat containing 20	LRRC20
13788	MRPS21	0.940	1.503	1.441	0.185	0.005	0.334	mitochondrial ribosomal protein S21	MRPS21
21557	TIMM8A	1.140	1.503	1.275	0.013	0.002	0.468	translocase of inner mitochondrial membrane 8 homolog A (yeast)	TIMM8A
19853	SNHG7	0.304	1.503	0.720	0.049	0.260	0.202	small nucleolar RNA host gene 7	SNHG7
1725	BIRC2	1.230	1.502	0.939	0.084	0.369	0.304	baculoviral IAP repeat containing 2	BIRC2
2451	C9orf114	0.908	1.502	1.366	0.404	0.009	0.390	chromosome 9 open reading frame 114	C9orf114
19127	SHKBP1	0.917	1.501	1.704	0.740	0.178	0.064	SH3KBP1 binding protein 1	SHKBP1
17294	PTN	0.338	1.501	0.720	0.347	0.276	0.651	pleiotrophin	PTN
8159	HIST1H2BG	1.000	1.501	1.376	0.714	0.239	0.159	histone cluster 1, H2bg	HIST1H2BG
16443	PLEKHJ1	0.835	1.501	1.248	0.435	0.169	0.432	pleckstrin homology domain containing J1	PLEKHJ1
20963	SYCE3	0.895	1.500	0.988	0.423	0.423	0.937	synaptonemal complex central element protein 3	SYCE3
8507	HSPA2	1.378	1.500	1.220	0.568	0.560	0.214	heat shock protein family A (Hsp70) member 2	HSPA2
4839	DGCR6L	0.630	1.500	1.253	0.188	0.016	0.497	DiGeorge syndrome critical region gene 6-like	DGCR6L
